# Influence of Ring Strain on the Formation of Rearrangement vs Cyclization Isotwistane Products in the Acyl Radical Reaction of Bicyclo[2.2.2]octanone

**DOI:** 10.1021/acs.orglett.3c02374

**Published:** 2023-09-22

**Authors:** Chih-Ming Chen, Sheng-Kuo Lin, Chi-Tien Hsieh, Julakanti Satyanarayana Reddy, Yi Ning Teoh, Mu-Jeng Cheng, Hsing-Pang Hsieh

**Affiliations:** † Institute of Biotechnology and Pharmaceutical Research, National Health Research Institutes, Miaoli County 350, Taiwan, ROC; ‡ Department of Chemistry, National Tsing Hua University, Hsinchu 300, Taiwan, ROC; § Department of Chemistry, National Cheng Kung University, Tainan 701, Taiwan, ROC; ∥ Biomedical Translation Research Center, Academia Sinica, Taipei City 115, Taiwan, ROC

## Abstract

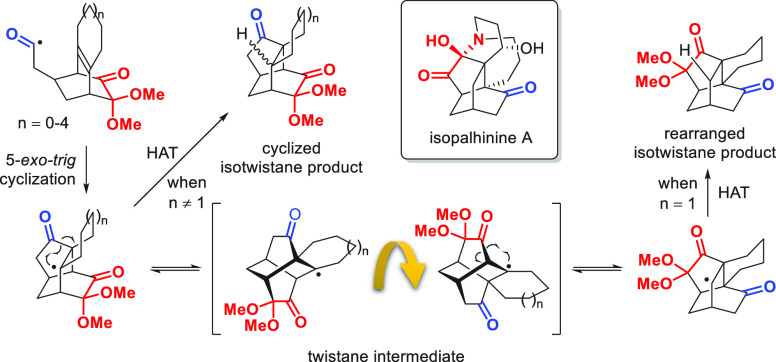

An acyl radical reaction of bicyclo[2.2.2]octenone to yield either rearranged or cyclized isotwistane products is described. The influence of ring strain on the reaction was demonstrated by alternating the sizes of the fused ring in the starting material. DFT calculations showed that the reaction is under thermodynamic control and proceeds via a 5-*exo*-*trig* cyclization intermediate, which undergoes either hydrogen-atom transfer (HAT) to give a cyclized product or rearrangement via a twistane intermediate to give a rearranged product.

The isotwistane motif, depicted in Scheme 1, is a rigid skeleton commonly found in various natural products, including the palhinines.^1,2^ Various strategies have been applied to its synthesis, such as Diels–Alder reactions, aldol reactions, radical cyclization, alkylation, and cascade reactions.^3^ In recent work, we reported the first use of a thiol-mediated acyl radical cyclization to construct the isotwistane skeleton from bicyclo[2.2.2]octenol **1** (Scheme 1),^4,5^ which resulted in intermediate **2** needed for the biomimetic total synthesis of the palhinines. Our original plan was to complete the synthesis via isotwistane intermediate **4**; however, when bicyclo[2.2.2]octanone **3** was reacted under thiol-mediated radical conditions, unexpected rearrangement product **5** was obtained instead of the desired cyclized product **4**. A plausible rearrangement mechanism to account for this result entails the formation of cyclopropoxy intermediate **B** through a cascade 6-*endo*-*trig*/3-*endo*-*trig* cyclization from acyl radical **A**, followed by ring opening and hydrogen-atom transfer (HAT) to yield rearrangement isotwistane product **5**.^6^

**Scheme 1:**
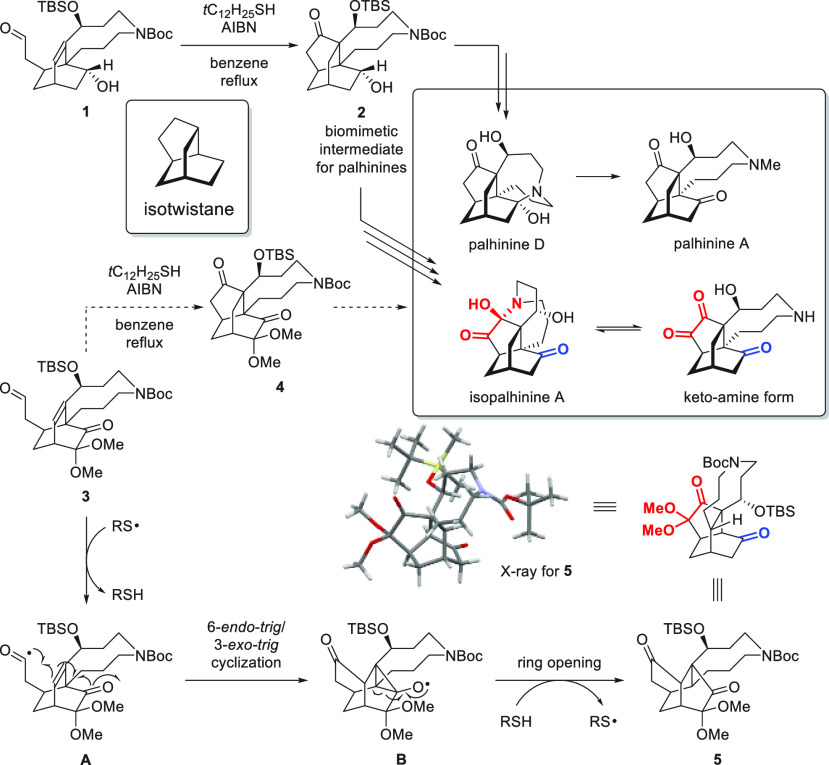
Previous Work of Total Synthesis of Palhinines and the Unexpected Rearrangement Product **5** Was Obtained from Bicyclo[2.2.2]octenone **3**

Although the rearrangement product **5** was unexpected, the locations of its oxygen containing functional groups are consistent with those of the isotwistane skeleton of isopalhinine A, which inspired us to develop a more atom-economic synthetic strategy toward the palhinines via the possible rearrangement intermediate **6** (Scheme 2). Accordingly, we synthesized bicyclo[2.2.2]octenone **7**, hoping to then obtain rearranged product **6** by an acyl radical reaction. However, when bicyclo[2.2.2]octenones **7a** and **7b** were treated with *t*C_12_H_25_SH/AIBN, the direct cyclized products **8a** and **8b**(7) were obtained through 5-*exo*-*trig* cyclization instead. Modifying the functional groups on C3 or C6 did not affect the outcome of the reaction. Previous synthetic studies of the palhinines^8^ concluded that the twisted structure of the isotwistane moiety may strain the 9-membered fused ring at its bridgeheads (C4 and C12).

**Scheme 2:**
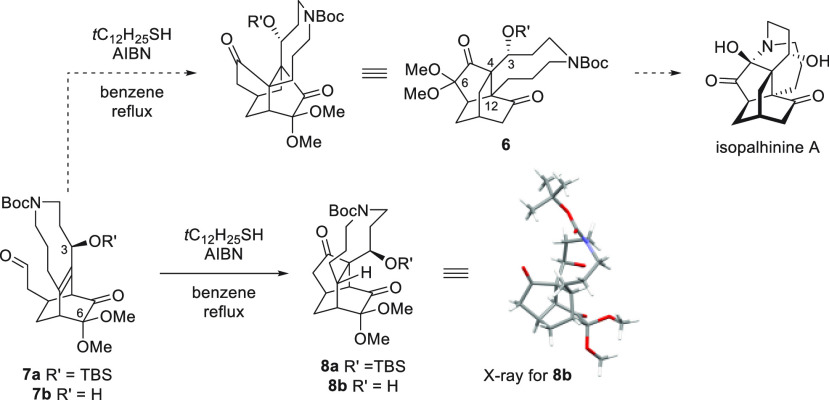
Our Failure To Obtain Rearrangement Product **6** from Bicyclo[2.2.2]octenone **7**

In addition, a synthesis of a tricyclo[m.2.2.0]skeleton^9^ via intramolecular Diels–Alder reactions of masked *ortho*-benzoquinones (MOBs)^10^ by Liao also reported the effect of ring strain on the yields of tricyclic products, with the yields of 7-membered ring products being significantly lower than those incorporating 6- or 5-membered fused rings. We hypothesized that a smaller 6- and 5-membered fused ring would be less strained at the bridgeheads than a 9-membered ring, and therefore attempted to reduce the fused ring size of the radical precursor to promote the formation of rearrangement product.

To investigate this hypothesis, 8-, 7-, 6-, and 5-membered fused ring precursors **9a**–**d** were synthesized (Scheme 3) and subjected to the rearrangement reaction conditions. The 8-, 7-, and 6-membered precursors **9a**–**c** were obtained from 3,4-dimethoxylbenzaldehyde **10**,^11^ to which carbon chains of various lengths were appended using the Wittig reaction to give a selection of alkene products, which were hydrogenated to give carboxylic acids **11a**–**c**. Friedel–Crafts cyclization reactions of **11a**–**c** yielded the requisite 8-, 7-, and 6-membered fused rings **12a**–**c**, which underwent demethylation and Clemmensen reduction to yield the desired 2-methoxyphenols **13a**–**c**. The 5-membered precursor **9d** was obtained from 5-indanol **14**,^12^ which led to desired 2-methoxyphenol **13d** by one-pot bromination and an Ullman-type coupling. As depicted in Scheme 3b, MOB intermediates **15a**–**d** were obtained by oxidative dearomatization of **13a**–**d** followed by a Diels–Alder reaction with acrolein to yield bicyclo[2.2.2]octenones **16a**–**d**. Finally, acyl radical precursors **12a**–**d** were obtained by the one-carbon elongation of **16a**–**d** via Wittig reactions and acidic hydrolysis.

**Scheme 3:**
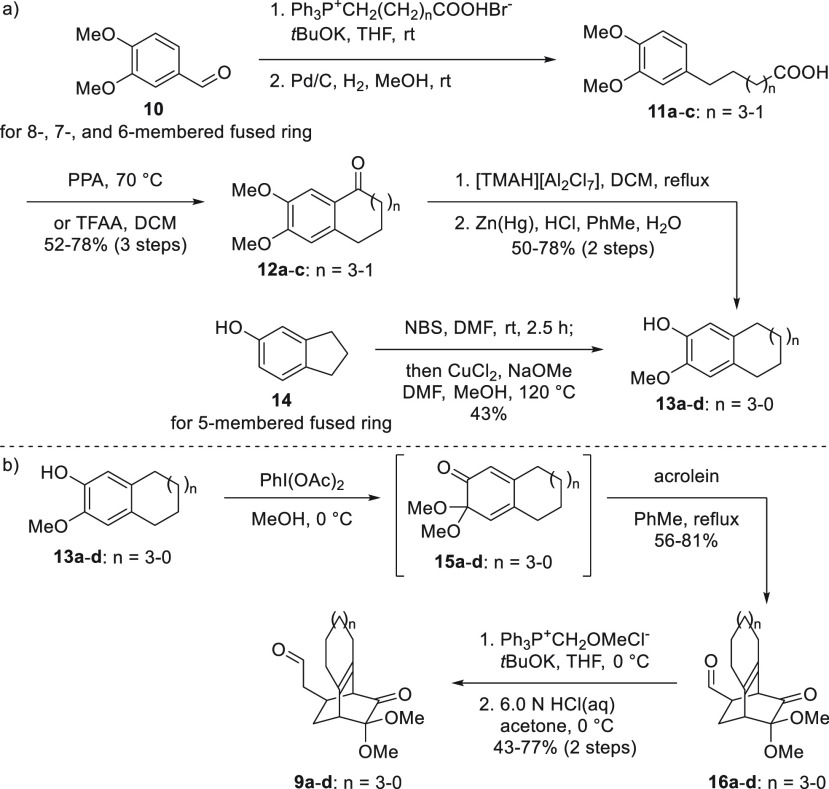
Syntheses of Radical Rearrangement Reaction Precursors **9a**–**d** with Reducing Size of Fused Ring

Acyl radical precursors **9a**–**d** were individually subjected to the thiol-mediated acyl radical reaction using *t*BuSH/AIBN; the results are presented in Table 1. As expected, reaction of the eight-membered fused ring **9a** under these conditions yielded the cyclized product **17a** in 54% yield, without any of the corresponding rearrangement product **18a** (entry 1). For the seven-membered fused ring **9b**, the desired rearrangement product **18b** was obtained in 10% yield (entry 2), with the major products of this reaction being a mixture of the cyclization products **17b** and **17b′**, a related alkene side product, in a ratio of 4:3 and a total yield of 60%. For the six-membered fused ring **9c**, the corresponding rearrangement product **18c** was obtained as a single product in 69% yield (entry 3), the structure of which was confirmed by X-ray crystallography.^13^ Surprisingly, the five-membered fused ring precursor **9d** did not yield predicted rearrangement product **18d**, but instead produced cyclization product **17d** as a single product in 53% yield.

**Table 1:**
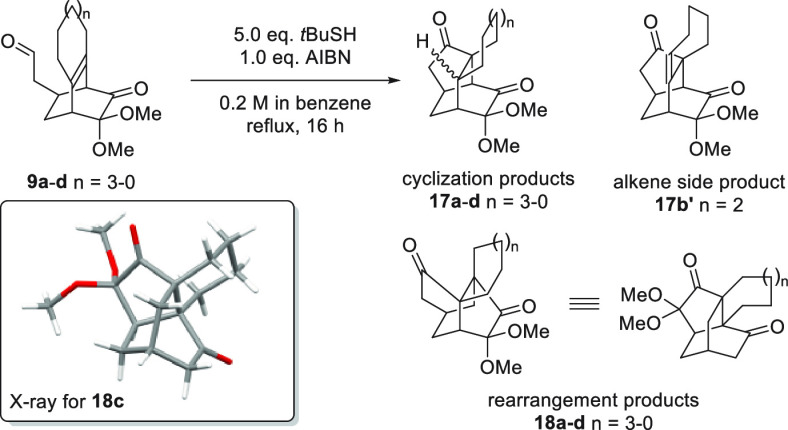

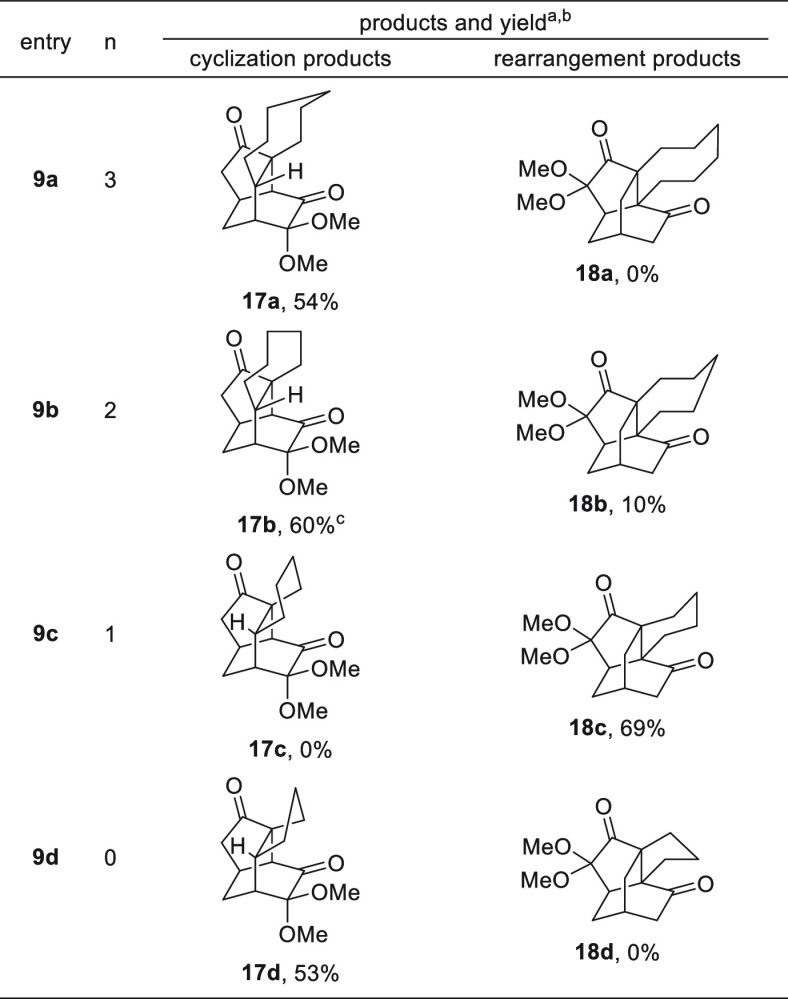
Results of Thiol-Mediated Acyl Radical Reaction with Different Ring Size of Fused Rings

a The strucure of products were confirmed by NMR spectra, **18c** was fur-ther confirmed by X-ray crystallography.

b The yield is isolated yield.

c A mixture with alkene side product **17b**′, the ratio 4:3 (**17b**:**17b**′) was determined by 1H-NMR spectrum.

To further confirm the effect of ring strain on this cyclization/rearrangement process, we synthesized compound **19** (Scheme 4) bearing two methyl groups in place of the fused ring on the bridged alkene to eliminate its influence on the reaction. Both rearrangement (**20**) and cyclization (**21**) products were obtained in a ratio of approximately 1:1. This result is further evidence that the ring strain of the fused ring governs the outcome of the thiol-mediated acyl radical reaction of a bicyclo[2.2.2]octenone skeleton to give either a rearranged or cyclized major product.

**Scheme 4:**
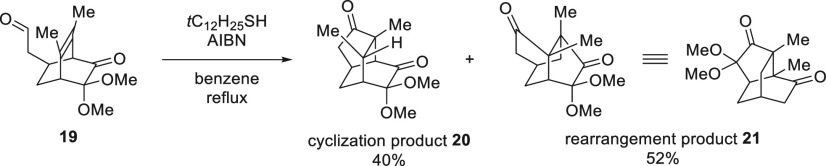
Model Study of Thiol-Mediated Acyl Radical Reaction without Fused Ring

Density functional theory (DFT) calculations were conducted to elucidate the reaction mechanism and rationalize the experimentally observed product selectivity. For reactant **19** lacking a fused ring, two possible pathways were identified, each of which leads to a different product (Scheme 5). Intermediate **19C** is first formed by hydrogen abstraction of **19** using *t*BuSH/AIBN. We assumed that this step is facile and spontaneous, and therefore, its energetics were not computed. In the next step, the newly formed acyl radical couples with one carbon of the bridged alkene to form a C–C bond (**19C** → **19D**). The reaction bifurcates at **19D**. In the cyclization pathway (depicted in red), **19D** abstracts a hydrogen from *t*BuSH to form cyclized product **20**. In the rearrangement pathway, **19D** forms a twistane intermediated **19E** via 3-membered ring rearrangement with the ketone on the 5-membered ring, followed by another 3-membered ring rearrangement with the other ketone and hydrogen abstraction to form the rearranged product **21** (**19D** → **19E** → **19F** → **21**, colored in blue).

**Scheme 5:**
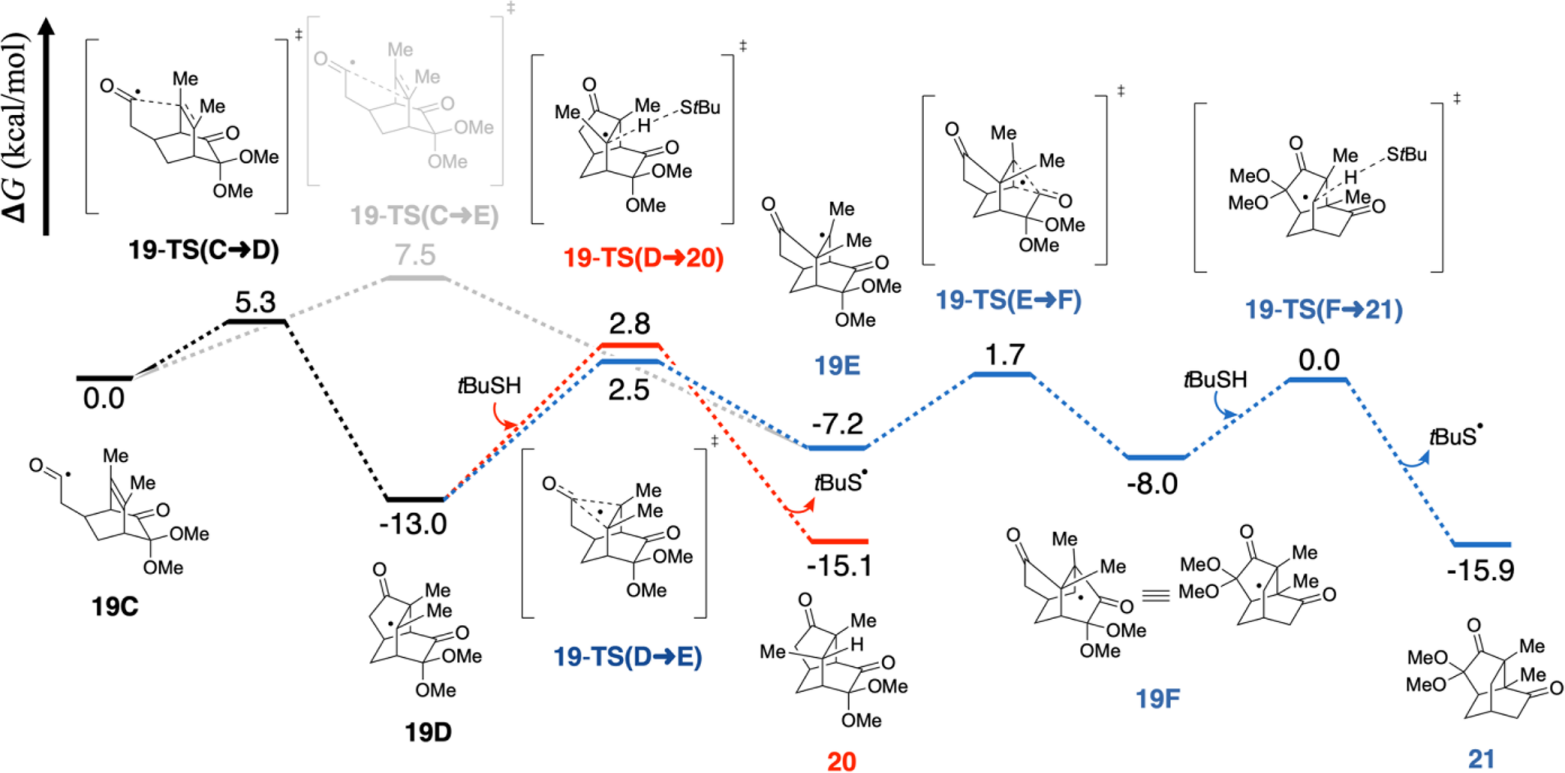
Calculated Free Energy Profile for **19** The red line indicates the cyclization pathway, the blue line indicates the rearrangement pathway, and the gray line indicates the 6-*endo*-*trig* cyclization pathway.

Starting from **19D**, where the two pathways branch, we found that the rate-determining step (RDS) for the cyclization pathway is **19D** → **20**, and **19D** → **19E** for the rearrangement pathway. It should be noted that the radical intermediate **19E** can be generated directly from **19C** and undergo a 6-*endo*-*trig* cyclization (**19C** → **19E**). However, this pathway has a kinetic barrier that is 2.2 kcal/mol higher than that for 5-*exo*-*trig* cyclization (**19C** → **19D**), suggesting it to be disfavored.

Gibbs free energy surfaces were calculated for **9a**–**d** (see Supporting Information); the RDSs for the two pathways are the same as **19**. The kinetic barriers of the RDS and reaction free energies for both pathways are summarized in Table 2 and compared to the observed product selectivity. Based on the kinetics, one would predict **9c** and **9d** to lead to the cyclization product, **9a** and **9b** to the rearrangement product, and **19** to both. This prediction is inconsistent with our previous experimental observations. In contrast, a prediction based on thermodynamics (**9a** and **9d** lead to the cyclization product, **9c** to the rearrangement product, **19** and **9b** to both products) is more consistent with our experiments, and suggests that, under the reaction conditions, all the kinetic barriers can be surmounted. Knowing that product selectivity is under thermodynamic control, the selectivity of the reaction can be predicted by comparing the relative energy levels of two potential products.

**Table 2:**
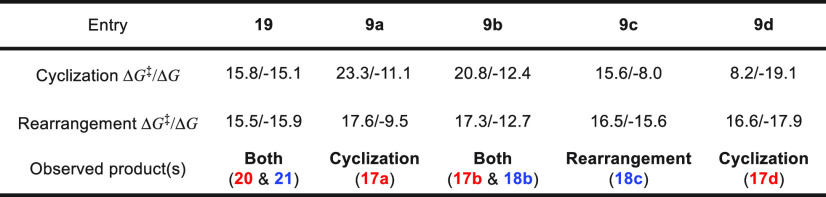
Calculated Overall Δ*G*^‡^ and Δ*G* for **19** and **9a**–**d** for Both Pathways

To further understand why starting material **9c** led to the rearrangement product, a hypothetical homodesmotic reaction, as proposed by previous studies,^14^ was used to calculate the ring strain energy. Products **20** and **21**, both lacking fused rings, were selected as reference products due to their lack of ring strain and comparable thermodynamic stabilities. A key requirement for the homodesmotic reaction is that equal numbers of C, CH, CH_2_, and CH_3_ components are on both sides of the equation. Therefore, in the calculation, the fused ring product **17** or **18** was reacted with ethane, removing the CH_2_ component from the fused ring, and yielding propane and the fused ring free product **20** or **21** (Table 3). Taking cyclization product **17c** as an example (where *n* equals one), four targeted CH_2_ units (orange) were in the fused ring of **17c**, while four CH_2_ units were also present in four units of propane; ten CH_3_ units (green) were in 5 units of ethane, while two CH_3_ units were in **20**, and eight CH_3_ units were in propane. The negative values of the reaction enthalpies (−Δ*H*) in these homodesmotic reactions were used to estimate the ring strain energies. The calculated ring strain energies explain why six-membered fused ring entry **9c** favored rearrangement product **18c**. This preference can be attributed to the minimal ring strain, likely resulting from the chair conformation of the six-membered fused ring.^15^

**Table 3:**
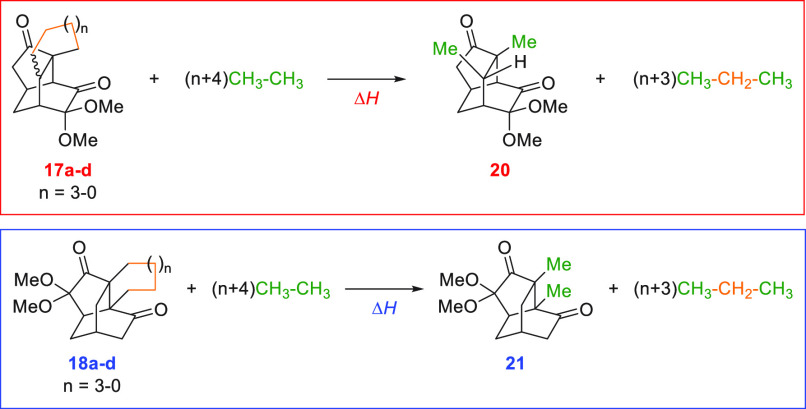

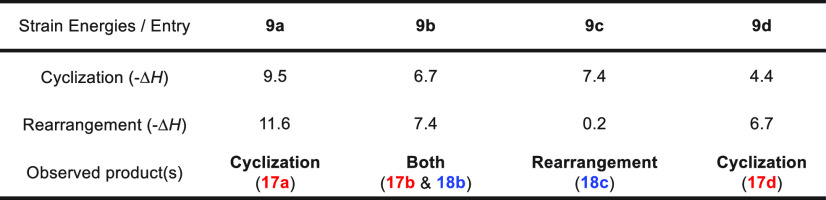
Calculated Strain Energies^a^

a The general formula for calculating ring strain energy is based on a homodesmotic reaction. The orange and green colors indicate the CH_2_ and CH_3_ units, respectively, which remain equal on both sides of the equation.

In summary, we have demonstrated the effect of ring strain on an acyl radical reaction of a bicyclo[2.2.2]octenone skeleton leading to the formation of cyclized or rearranged isotwistane products. Based on these results and those of DFT calculations, the proposed rearrangement mechanism involves a two-step 3-membered ring rearrangement (**D** → **E** → **F**) proceeding via twistane intermediate **E**. Currently, we are exploring these pathways pursuant to a total synthesis of isopalhinine A and related natural products based on isotwistane or similar skeletons. We are also investigating the effect of other substituents on the outcome of this acyl radical reaction; the results will be published in due course.

## Data Availability

The data underlying this study are available in the published article and its Supporting Information.

## Supporting Information Available

General procedures, experimental procedures, X-ray crystal data for **5**, **8b**, and **18c**, characterization data for new compounds, and computational details including computational levels, free energy profiles for **9a**–**d**, energetics, and optimized structures. (PDF)
