# Supplementary material for: Influence of Ring Strain on the Formation of Rearrangement vs Cyclization Isotwistane Products in the Acyl Radical Reaction of Bicyclo[2.2.2]octanone
Source: Org Lett. 2023 Sep 22;25(43):7757–62. doi: 10.1021/acs.orglett.3c02374 (PMC10630961; doi:10.1021/acs.orglett.3c02374)
Supplement: Supplementary file 1 — ol3c02374_si_001.pdf [file ol3c02374_si_001.pdf]

Supporting Information

Influence of Ring Strain on the Formation of Rearrangement vs Cyclization Isotwistane Products in the Acyl Radical Reaction of Bicyclo[2.2.2]octanone

Chih-Ming Chen,[a,‡] Sheng-Kuo Lin,[a,b,‡] Chi-Tien Hsieh,[c,‡] Julakanti Satyanarayana Reddy,[a] Yi Ning Teoh,[a,b] Mu-Jeng Cheng,[c,*] Hsing-Pang Hsieh[a,b,d,*]

a. Institute of Biotechnology and Pharmaceutical Research, National Health Research Institutes, Miaoli County 350, Taiwan, ROC.

b. Department of Chemistry, National Tsing Hua University, Hsinchu 300, Taiwan, ROC.

c. Department of Chemistry, National Cheng Kung University, Tainan 701, Taiwan, ROC.

d. Biomedical Translation Research Center, Academia Sinica, Taipei City 115, Taiwan, ROC.

Email: hphsieh@nhri.edu.tw; mjcheng@mail.ncku.edu.tw

Table of Contents

1. General Procedures.......2
2. Experimental Procedures.......3
2.1 The syntheses of 2-methoxyphenols 13a-b with different fused ring.......3
2.2 The syntheses of acyl radical precursors 9a-d.......9
2.3 The thiol-mediated acyl radical reaction of radical precursors 9a-d.......15
2.4 The synthesis of dimethyl model study........19
3. Computational Details.......24
4. References.......29
5. Coordinates of Optimized Structures.......31
6. X-Ray Crystal Data.......69
7. ^{1}H- and ^{13}C-NMR Spectra.......75

1. General Procedures

Unless otherwise mentioned, reagents were obtained from commercial sources and used without further purification. High temperature reactions were heated in oil baths. All moisture- or oxygen-sensitive reactions were performed under positive pressure of anhydrous argon or nitrogen in anhydrous solvents, which were dried prior to use following standard procedures. Thin layer chromatography (TLC) was performed using Merck 5554 DC-Alufolien Kieselgel 60 F254. Flash column chromatography was performed using 230–400 mesh silica gel from Merck Art.9385 Kieselgel 60H. Except as otherwise indicated, yields were calculated after flash column chromatography. ^{1}H-NMR and ^{13}C-NMR were measured by using Varian Mercury-300 MHz, Varian Mercury-400 MHz, Bruker AVANCE NEO-400 MHz and Bruker AVANCE NEO-600 MHz spectrometers. Chemical shifts are reported as δ values in ppm, and calibrated using residual undeuterated solvent ($CDCl_3$ (7.27 ppm) or CD_3OD (3.31 ppm)) as internal reference for ^{1}H NMR and the deuterated solvent ($CDCl_3$ (77.00 ppm) or CD_3OD (49.00 ppm)) as internal standard for ^{13}C NMR. Coupling constants are reported in Hz; multiplicities are indicated as follows: s (singlet); d (doublet); t (triplet); m (multiplet). Infrared (IR) spectra were recorded on a Perkin Elmer FT-IR Spectrometer Spectrum RXI and are reported in wavenumbers (cm^{-1}). High resolution mass spectra (HRMS) were recorded using a VARIAN 901-MS/TOF. Melting points were determined with a Krüss Optronic KSP1N melting point meter. X-ray diffraction analysis was measured on a Bruker D8 Dual Single Crystal X-ray Diffractometer (D8 Venture *IuS* 3.0 Dual source) with monochromatic MoKα radiation.

2. Experimental Procedures

2.1 The syntheses of 2-methoxyphenols 13a-b with different fused ring

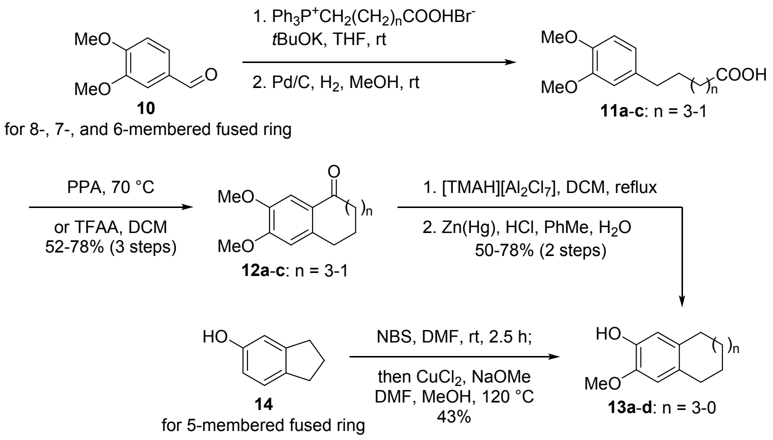

Compounds **13a-d** were prepared via modified literature procedures.[1,2]

For 8-membered fused ring compound 12a

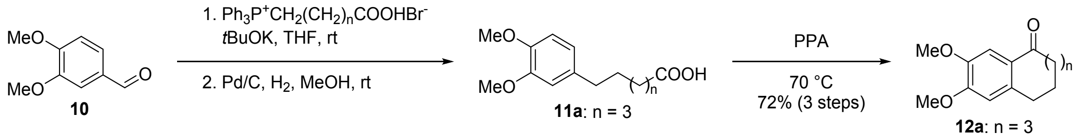

1.0 M *t*BuOK in THF (18.1 mL, 18.10 mmol, 3.0 eq.) was added to a two-neck round-bottom flask charged with (4-carboxybutyl)triphenylphosphonium bromide (3.201 g, 7.22 mmol, 1.2 eq.) at 0 ℃. After stirring at 0 ℃ for 10 min, a solution of 3,4-dimethoxylbenzaldehyde **10** (1.000 g, 6.02 mmol) in THF (42.1 mL) was added. The reaction mixture was warmed to room temperature and stirred for overnight, then cooled to 0 ℃, diluted with water and carefully acidified to pH 2 by the portionwise addition of 2.0 N HCl. The mixture was then extracted with EtOAc and the combined organic extracts were dried over $NaSO_4$, filtered, and concentrated *in vacuo* to afford E/Z mixture of the crude olefin.

10% Pd on carbon (0.064 g, 0.06 mmol, 1 mol%) was added to a solution of the crude olefin in MeOH (60 mL). The flask was evacuated under vacuum and H_2 gas was introduced via balloon. After stirring at room temperature for 7 h, the reaction mixture was filtered through Celite® and the filtrate was concentrated *in vacuo* to afford crude carboxylic acid **11a**.

A mixture of crude carboxylic acid **11a** and polyphosphoric acid (113.3 g) was heated and stirred at 70 ℃ for 3 h. The reaction mixture was then diluted with water and extracted with DCM. The combined organic extracts were dried over $NaSO_4$, filtered, and concentrated *in vacuo*. The crude residue was purified by silica gel column chromatography (10% to 33% EtOAc/hexane) to afford **12a** as white solid (1.061 g, 72% yield over 3 steps).

Mp: 106.3-107.5 °C.

^{1}H NMR (300 MHz, $CDCl_3$): δ 7.61 (s, 1H), 6.64 (s, 1H), 3.94 (s, 3H), 3.93 (s, 3H), 3.16 (t, J = 6.6 Hz, 2H), 3.04 (t, J = 6.9 Hz, 2H), 1.93-1.82 (m, 2H), 1.80 (tt, J = 6.6, 6.6 Hz, 2H), 1.51-1.39 (m, 2H).

^{13}C NMR (75 MHz, $CDCl_3$): δ 202.3, 152.3, 147.1, 135.2, 131.8, 113.8, 111.5, 55.8, 55.8, 42.4, 35.0, 27.5, 24.4, 22.6.

IR (neat): 2931, 2852, 1654, 1596, 1568, 1512, 1464, 1446, 1399, 1353, 1308, 1262, 1218, 1182, 1148, 1124, 1073, 1045 cm^{-1}.

HRMS (ESI): calculated for $C_{14}H_{18}NaO_3$ 257.1148 [M+Na]$^+$, found 257.1152.

For 7-membered fused ring compound 12b

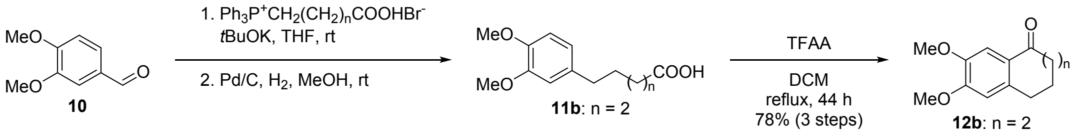

1.0 M *t*BuOK in THF (36.0 mL, 36.00 mmol, 3.0 equiv.) was added to a two-neck round-bottom flask charged with (3-carboxypropyl)triphenylphosphonium bromide (6.203 g, 14.45 mmol, 1.2 equiv.) at 0 °C. After stirring at 0 °C for 10 min, a solution of 3,4-dimethoxylbenzaldehyde **10** (2.000 g, 12.04 mmol) in THF (84 mL) was added. The reaction mixture was warmed to room temperature and stirred for 40 min, then cooled to 0 °C, diluted with water and carefully acidified to pH 2 by the portion-wise addition of 6.0 N HCl. The mixture was then extracted with DCM and the combined organic extracts were dried over $NaSO_4$, filtered, and concentrated *in vacuo* to afford E/Z mixture of the crude olefin.

10% Pd on carbon (0.128 g, 0.12 mmol, 1 mol%) was added to a solution of the crude olefin in MeOH (60 mL). The flask was evacuated under vacuum and H_2 gas was introduced via balloon. After stirring at room temperature for 16 h, the reaction mixture was filtered through Celite® and the filtrate was concentrated *in vacuo* to afford crude carboxylic acid **11b**.

A mixture of crude carboxylic acid **11b**, trifluoroacetic anhydride (3.0 mL, 21.67 mmol, 1.8 eq.) and DCM (60 mL) was heated to reflux and stirred for 23 h. A second portion of trifluoroacetic anhydride (1.5 mL, 10.84 mmol, 0.9 eq.) was added and the reaction was stirred for 6 h. A third portion of trifluoroacetic anhydride (1.5 mL, 10.84 mmol, 0.9 eq.) was added and the reaction was stirred for 9 h. The final portion of trifluoroacetic anhydride (1.5 mL, 10.84 mmol, 0.9 eq.) was added and the reaction was stirred for 6 h. The reaction mixture was cooled to 0 °C, then diluted with 5.0 M NaOH (18 mL) in MeOH (90 mL) to quench and hydrolyze side product, enol trifluoroacetate, to regenerate desire ketone product. The majority of the solvent was then removed *in vacuo*, and the residue was diluted with water (100 mL) and extracted with DCM (3 x

100 mL). The combined organic extracts were dried over $NaSO_4$, filtered, and concentrated *in vacuo*. The crude residue was purified by silica gel column chromatography (0% to 50% EtOAc/hexane) to afford **12b** as white solid (2.062 g, 78% yield over 3 steps). The spectroscopic data was identical to the one reported in the literature.[3]

For 6-membered fused ring compound 12c

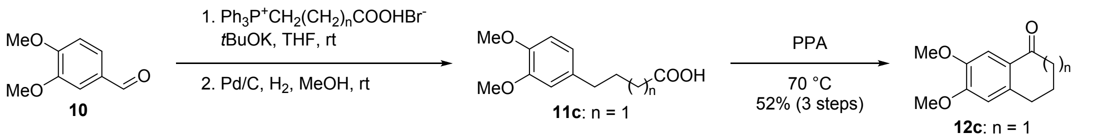

1.0 M *t*BuOK in THF (15.1 mL, 15.10 mmol, 2.5 eq.) was added to a solution of 3,4-dimethoxylbenzaldehyde **10** (1.000 g, 6.02 mmol) and (2-carboxyethyl)triphenyl-phosphonium bromide (2.749 g, 6.62 mmol, 1.1 eq.) in THF (45.1 mL) at -78 °C. After stirring at -78 °C for 1 h, the reaction mixture was warmed to room temperature and stirred overnight. The majority of the solvent was then removed *in vacuo*, and the residue was diluted with water and extracted with DCM. The aqueous phase was cooled to 0 °C and carefully acidified to pH 2 by the portion-wise addition of 6.0 N HCl, then extracted with DCM. The combined organic extracts were dried over $NaSO_4$, filtered, and concentrated *in vacuo* to afford E/Z mixture of the crude olefin.

10% Pd on carbon (0.064 g, 0.06 mmol, 1 mol%) was added to a solution of the crude olefin in MeOH (30.1 mL). The flask was evacuated under vacuum and H_2 gas was introduced via balloon. After stirring at room temperature for 4 h, the reaction mixture was filtered through Celite® and the filtrate was concentrated *in vacuo* to afford crude carboxylic acid **11c**.

A mixture of crude carboxylic acid **11c** and polyphosphoric acid (41.2 g) was heated and stirred at 70 °C for 2 h. The reaction mixture was then diluted with water and extracted with DCM. The combined organic extracts were dried over $NaSO_4$, filtered, and concentrated *in vacuo*. The crude residue was purified by silica gel column chromatography (0% to 33% EtOAc/hexane) to afford **12c** as white solid (0.650 g, 52% yield over 3 steps). The spectroscopic data was identical to the one reported in the literature.[3]

For 2-methoxyphenol 13a with 8-membered fused ring

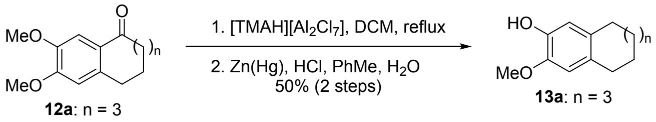

A mixture of trimethylamine hydrochloride (0.736 g, 7.70 mmol, 2.2 eq.) and $AlCl_3$ (2.053 g, 15.40 mmol, 4.4 eq.) in DCM (15.4 mL) was stirred at room

temperature for 2 h to provide 0.5 M of [TMAH][Al_2Cl_7] in DCM. The above solution was added to a flask charged with **12a** (0.821 g, 3.50 mmol) and the reaction was heated to reflux and stirred for 10 h. The reaction mixture was cooled to 0 °C, diluted with water and extracted with DCM. The combined organic extracts were dried over $NaSO_4$, filtered, and concentrated *in vacuo*. The crude residue was redissolved by EtOAc and extracted by 2.0 M NaOH. The EtOAc layer was dried over $NaSO_4$, filtered, concentrated *in vacuo*, then purified by silica gel column chromatography (0% to 33% EtOAc/hexane) to recover the starting material **12a** as white solid (0.203 g, 25% recovery yield). The basic aqueous phase was cooled to 0 °C, carefully acidified to pH 2 by the portionwise addition of 6.0 N HCl, then extracted with DCM. The combined organic extracts were dried over $NaSO_4$, filtered, and concentrated *in vacuo* to provide crude phenol as a mixture (0.552 g).

0.2 N HCl (8.4 mL) was added to a mixture of zinc dust (1.969 g, 30.12 mmol, 12.0 eq.) and $HgCl_2$ (0.204 g, 0.75 mmol, 0.3 eq.) at 0 °C. The mixture was stirred at room temperature for 10 min and the supernatant liquid was decanted to provide amalgamated zinc. Amalgamated zinc, 10.0 N HCl (8.4 mL) and toluene (4.2 mL) were added to a flask charged with the mixture of the crude phenol (0.552 g, 2.51 mmol). The reaction was heated to reflux and stirred for 6 h. The reaction mixture was cooled to room temperature, diluted with water and extracted with DCM. The combined organic extracts were dried over $NaSO_4$, filtered, and concentrated *in vacuo*. The crude residue was purified by silica gel column chromatography (0% to 10% EtOAc/hexane) to afford 2-methoxyphenol **13a** as white solid (0.364 g, 50% yield over 2 steps).

Mp: 69.3-71.7 °C.
^{1}H NMR (300 MHz, $CDCl_3$): δ 6.68 (s, 1H), 6.60 (s, 1H), 5.40 (s, 1H), 3.87 (s, 3H), 2.75-2.60 (m, 4H), 1.72-1.56 (m, 4H), 1.42-1.28 (m, 4H).
^{13}C NMR (75 MHz, $CDCl_3$): δ 144.6, 143.4, 133.7, 132.3, 115.0, 111.4, 55.7, 32.2, 32.0, 31.8, 31.4, 25.7 (2C).
IR (neat): 3546, 3436, 2922, 2847, 1623, 1594, 1510, 1466, 1450, 1361, 1285, 1251, 1230, 1211, 1193, 1173, 1163, 1108, 1075, 1042, 1010, 957 cm^{-1}.
HRMS (ESI): calculated for $C_{13}H_{17}O_2$ 205.1234 $[M-H]^-$, found 205.1229.

For 2-methoxyphenol 13b with 7-membered fused ring

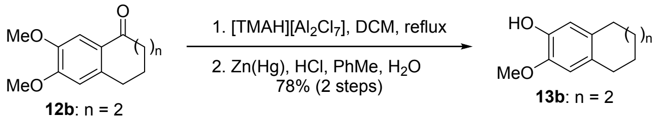

A mixture of trimethylamine hydrochloride (4.837 g, 50.61 mmol, 2.5 eq.) and $AlCl_3$ (13.500 g, 101.24 mmol, 5.0 eq.) in DCM (100 mL) was stirred at room

temperature for 2 h to provide 0.5 M of [TMAH][Al_2Cl_7] in DCM. 0.5 M of [TMAH][Al_2Cl_7] in DCM (80 mL) was added to a flask charged with **12b** (4.398 g, 19.97 mmol) and the reaction was heated to reflux and stirred for 12 h. A second portion of 0.5 M of [TMAH][Al_2Cl_7] in DCM (20 mL) was then added and the reaction was continued to stirred for 17 h. The reaction mixture was cooled to 0 °C, diluted with water and extracted with DCM. The combined organic extracts were dried over $NaSO_4$, filtered, and concentrated *in vacuo* to provide crude phenol as a mixture.

0.2 N HCl (67 mL) was added to a mixture of zinc dust (15.668 g, 239.64 mmol, 12.0 equiv.) and $HgCl_2$ (1.626 g, 5.99 mmol, 0.3 equiv.) at 0 °C. The mixture was stirred at room temperature for 5 min and the supernatant liquid was decanted to provide amalgamated zinc. Amalgamated zinc, 10.0 N HCl (67 mL) and toluene (33 mL) were added to a flask charged with the mixture of the crude phenol. The reaction was heated to reflux and stirred for 6 h. The reaction mixture was cooled to room temperature, diluted with water and extracted with DCM. The combined organic extracts were dried over $NaSO_4$, filtered, and concentrated *in vacuo*. The crude residue was purified by silica gel column chromatography (0% to 15% EtOAc/hexane) to afford 2-methoxyphenol **13b** as white solid (3.00 g, 78% yield over 2 steps).

Mp: 102.6-104.5 °C.
^{1}H NMR (600 MHz, $CDCl_3$): δ 6.70 (s, 1H), 6.64 (s, 1H), 5.37 (s, 1H), 3.86 (s, 3H), 2.74-2.70 (m, 2H), 2.70-2.66 (m, 2H), 1.85-1.77 (m, 2H), 1.66-1.58 (m, 4H).
^{13}C NMR (150 MHz, $CDCl_3$): δ 143.9, 142.9, 136.3, 135.0, 115.6, 112.2, 56.0, 36.4, 36.0, 32.7, 28.6, 28.5.
IR (neat): 3538, 3053, 2912, 2844, 2686, 1618, 1595, 1509, 1449, 1357, 1335, 1325, 1287, 1263, 1235, 1208, 1192, 1167, 1128, 1102, 1083, 1012, 964 cm^{-1}.
HRMS (ESI): calculated for $C_{12}H_{17}O_2$ 193.1223 $[M+H]^+$, found 193.1230.

For 2-methoxyphenol 13c with 6-membered fused ring

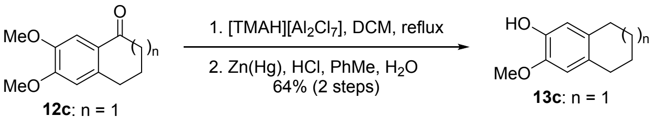

A mixture of trimethylamine hydrochloride (0.868 g, 9.08 mmol, 2.5 eq.) and $AlCl_3$ (2.412 g, 18.16 mmol, 5.0 eq.) in DCM (18.2 mL) was stirred at room temperature for 2 h to provide 0.5 M of [TMAH][Al_2Cl_7] in DCM. To a flask charged with **12c** (0.748 g, 3.63 mmol) was added 0.5 M of [TMAH][Al_2Cl_7] in DCM (18.2 mL) and the reaction was heated to reflux and stirred for 12 h. The reaction mixture was cooled to 0 °C, diluted with water and extracted with DCM. The combined organic extracts were dried over $NaSO_4$, filtered, and concentrated *in vacuo*. The crude residue

was redissolved by EtOAc and extracted by 2.0 M NaOH. The organic layer was discarded and the aqueous phase was cooled to 0 °C and carefully acidified to pH 2 with portionwise addition of 6.0 N HCl. It was then extracted with DCM and the combined organic extracts were dried over $NaSO_4$, filtered, and concentrated *in vacuo* to provide crude phenol as a mixture.

0.2 N HCl (11.2 mL) was added to a mixture of zinc dust (2.636 g, 40.32 mmol, 12.0 equiv.) and $HgCl_2$ (0.274 g, 1.01 mmol, 0.3 equiv.) at 0 °C. The mixture was stirred at room temperature for 5 min and the supernatant liquid was decanted to provide amalgamated zinc. Amalgamated zinc, 10.0 N HCl (11.2 mL) and toluene (5.6 mL) were added to a flask charged with the mixture of the crude phenol. The reaction was heated to reflux and stirred for 8 h. The reaction mixture was cooled to room temperature, diluted with water and extracted with DCM. The combined organic extracts were dried over $NaSO_4$, filtered, and concentrated *in vacuo*. The crude residue was purified by silica gel column chromatography (0% to 15% EtOAc/hexane) to afford 2-methoxyphenol **13c** as white solid (0.413 g, 64% yield over 2 steps). The spectroscopic data was identical to the one reported in the literature.[4]

For 2-methoxyphenol 13d with 5-membered fused ring

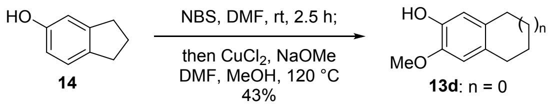

NBS (2.653 g, 14.91 mmol, 1.0 eq.) was slowly added to a solution of 5-indanol **14** (2.000 g, 14.91 mmol) in DMF (30 mL) at 0 °C. The reaction mixture was warmed to room temperature and stirred for 2.5 h. $CuCl_2$ (1.002 g, 7.45 mmol, 0.5 eq.) and 5.4 M NaOMe in MeOH (27.6 mL, 149.10 mmol, 10.0 eq.) were then added and the mixture was heated to 120 °C. After stirring for 4.5 h, the reaction mixture was cooled to 0 °C and diluted with water (90 mL), then carefully acidified to pH 2 by the portion-wise addition of 6 N HCl (27 mL) and extracted with Et_2O (4 x 90 mL). The combined organic extracts were dried over $NaSO_4$, filtered, and concentrated in vacuo. The crude residue was purified by silica gel column chromatography (0% to 10% EtOAc/hexane) to afford of 2-methoxyphenol **13d** as white solid (1.044 g, 43% yield). The spectroscopic data was identical to the one reported in the literature.[5]

2.2 The syntheses of acyl radical precursors 9a-d

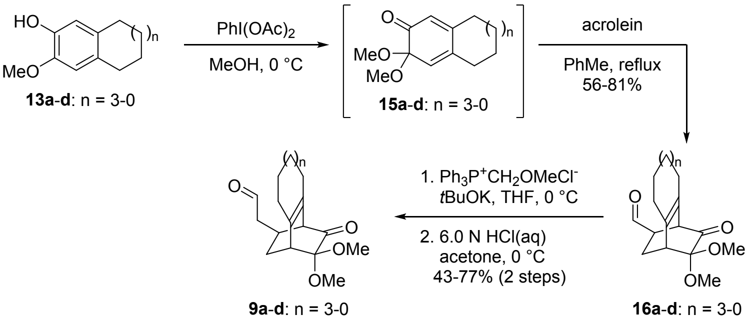

For Diels-Alder adduct 16a with 8-membered fused ring

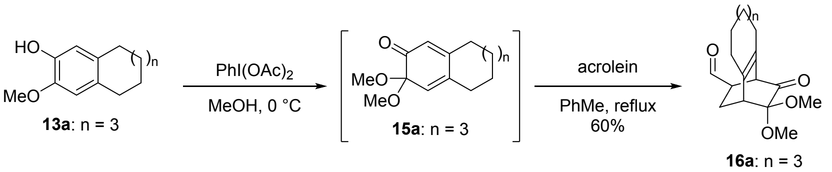

PhI(OAc)$_2$ (0.709 g, 2.20 mmol, 1.3 eq.) was added to a solution of 2-methoxyphenol **13a** (0.349 g, 1.69 mmol) in methanol (8.5 mL) at 0 °C. After stirring at 0 °C for 10 min, acrolein (2.3 mL, 34.42 mmol, 20.4 eq.) and toluene (84.5 mL) was added. The reaction was heated to reflux and stirred for 6 h. The reaction mixture was cooled to room temperature, and the solvent was removed *in vacuo*. The crude residue was purified by silica gel column chromatography (0% to 25% EtOAc/hexane) to afford Diels-Alder adduct **16a** as colorless oil (0.299 g, 60% yield).

^{1}H NMR (300 MHz, CDCl$_3$): δ 9.57 (s, 1H), 3.37 (s, 3H), 3.35 (d, *J* = 1.8 Hz, 1H), 3.32 (s, 3H), 3.03-2.92 (m, 2H), 2.45-2.20 (m, 3H), 2.20-2.04 (m, 2H), 1.78 (ddd, *J* = 12.9, 6.0, 2.7 Hz, 1H), 1.72-1.40 (m, 6H), 1.40-1.24 (m, 2H).
^{13}C NMR (75 MHz, CDCl$_3$): δ 200.1, 200.0, 142.0, 129.0, 94.5, 53.3, 49.9, 49.8, 46.4, 44.3, 31.4, 30.2, 28.2, 28.0, 25.9, 25.0, 21.8.
IR (neat): 3452, 2927, 2851, 2714, 1732, 1463, 1454, 1390, 1359, 1314, 1251, 1190, 1136, 1127, 1096, 1066, 1049, 996, 979, 958 cm^{-1}.
HRMS (ESI): calculated for $C_{17}H_{24}NaO_4$ 315.1567 $[M+Na]^+$, found 315.1574.

For Diels-Alder adduct 16b with 7-membered fused ring

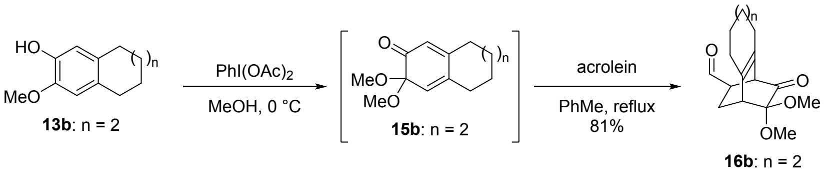

PhI(OAc)$_2$ (1.105 g, 3.43 mmol, 1.2 equiv.) was added to a solution of **13b** (0.549 g, 2.86 mmol) in methanol (14.3 mL) at 0 °C. After stirring at 0 °C for 15 min, acrolein

(3.8 mL, 56.87 mmol, 19.9 equiv.) and toluene (143 mL) was added. The reaction was heated to reflux and stirred for 6 h. The reaction mixture was cooled to room temperature, and the solvent was removed *in vacuo*. The crude residue was purified by silica gel column chromatography (0% to 25% EtOAc/hexane) to afford Diels-Alder adduct **16b** as colorless oil (0.641 g, 81% yield).

^{1}H NMR (400 MHz, $CDCl_3$): δ 9.56 (d, *J* = 0.8 Hz, 1H), 3.35 (s, 3H), 3.33 (s, 3H), 3.30 (d, *J* = 2.4 Hz, 1H), 2.98-2.92 (m, 1H), 2.94 (t, *J* = 2.8 Hz, 1H), 2.37-2.32 (m, 2H), 2.30-2.14 (m, 2H), 2.10 (ddd, *J* = 12.8, 10.0, 2.8 Hz, 1H), 1.81 (ddd, *J* = 12.8, 5.6, 2.8 Hz, 1H), 1.71-1.59 (m, 3H), 1.56-1.42 (m, 3H).
^{13}C NMR (100 MHz, $CDCl_3$): δ 200.5, 200.0, 143.8, 131.2, 94.6, 55.1, 50.2, 49.9, 46.7, 45.9, 34.4, 33.9, 30.4, 26.5 (2C), 21.3.
IR (neat): 3630, 3531, 3449, 2923, 2851, 2836, 2714, 1738, 1732, 1448, 1391, 1362, 1313, 1277, 1253, 1206, 1139, 1108, 1078, 1051, 1002, 982, 964, 909 cm^{-1}.
HRMS (ESI): calculated for $C_{16}H_{22}NaO_4$ 301.1410 $[M+Na]^+$, found 301.1416.

For Diels-Alder adduct 16c with 6-membered fused ring

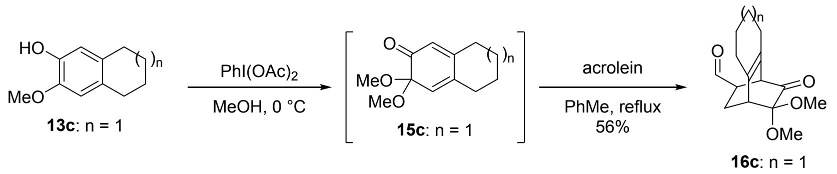

PhI(OAc)$_2$ (0.973 g, 3.02 mmol, 1.3 equiv.) at 0 °C was added to a solution of **13c** (0.413 g, 2.32 mmol) in methanol (11.6 mL). After stirring at 0 °C for 4 min, acrolein (3.3 mL, 49.39 mmol, 21.3 equiv.) and toluene (116 mL) was added. The reaction was heated to reflux and stirred for 6 h, then cooled to room temperature, and the solvent was removed *in vacuo*. The crude residue was purified by silica gel column chromatography (0% to 33% EtOAc/hexane) to afford Diels-Alder adduct **16c** as colorless oil (0.340 g, 56% yield).

^{1}H NMR (300 MHz, $CDCl_3$): δ 9.55 (s, 1H), 3.35 (s, 3H), 3.33 (s, 3H), 3.25 (d, *J* = 1.8 Hz, 1H), 3.04-2.94 (m, 1H), 2.87 (dd, *J* = 2.7, 2.7 Hz, 1H), 2.20-2.06 (m, 3H), 2.06-1.88 (m, 2H), 1.79 (ddd, *J* = 12.9, 5.7, 2.7 Hz, 1H), 1.72-1.50 (m, 4H).
^{13}C NMR (75 MHz, $CDCl_3$): δ 200.1, 199.8, 139.9, 127.3, 95.0, 52.1, 50.3, 49.9, 47.0, 42.4, 27.7, 27.4, 22.4, 22.2, 21.5.
IR (neat): 3450, 2941, 2858, 2834, 2714, 1730, 1669, 1450, 1437, 1390, 1310, 1264, 1251, 1206, 1179, 1158, 1135, 1094, 1054, 1002, 978, 956, 920, 899 cm^{-1}.
HRMS (ESI): calculated for $C_{15}H_{21}O_4$ 265.1434 $[M+H]^+$, found 265.1443.

For Diels-Alder adduct 16d with 5-membered fused ring

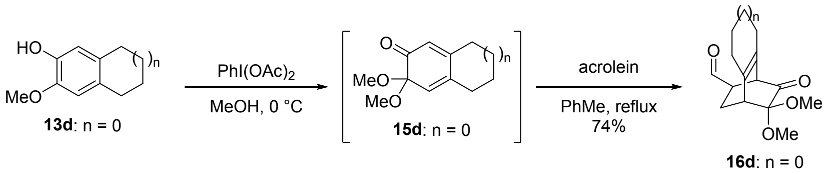

PhI(OAc)$_2$ (2.754 g, 8.55 mmol, 1.3 equiv.) was added to a solution of **13d** (1.080 g, 6.58 mmol) in methanol (32.9 mL) at 0 °C. After stirring at 0 °C for 15 min, acrolein (4.4 mL, 65.85 mmol, 10.0 equiv.) and toluene (329 mL) were added. The reaction was heated to reflux and stirred for 6 h. The reaction mixture was cooled to room temperature, and the solvent was removed *in vacuo*. The crude residue was purified by silica gel column chromatography (0% to 15% EtOAc/hexane) to afford Diels-Alder adduct **16d** as colorless oil (1.212 g, 74% yield).

^{1}H NMR (600 MHz, CDCl$_3$): δ 9.52 (d, *J* = 0.6 Hz, 1H), 3.59 (d, *J* = 1.8 Hz, 1H), 3.35 (s, 3H), 3.32 (s, 3H), 3.23 (dd, *J* = 3.0, 3.0 Hz, 1H), 3.04 (dddd, *J* = 9.6, 5.4, 1.8, 0.6 Hz, 1H), 2.50-2.45 (m, 2H), 2.45-2.40 (m, 1H), 2.37-2.30 (m, 1H), 2.19 (ddd, *J* = 12.6, 9.6, 3.0 Hz, 1H), 2.06-1.98 (m, 1H), 1.95-1.87 (m, 1H), 1.75 (ddd, *J* = 12.6, 5.4, 3.0 Hz, 1H).
^{13}C NMR (150 MHz, CDCl$_3$): δ 199.6, 199.2, 145.7, 133.7, 95.0, 50.1, 49.9, 48.7, 47.2, 38.5, 33.1, 32.9, 23.3, 21.8.
IR (neat): 3448, 2952, 2847, 2716, 1729, 1450, 1389, 1310, 1196, 1130, 1103, 1052, 999, 981, 950, 894 cm^{-1}.
HRMS (ESI): calculated for $C_{14}H_{18}NaO_4$ 273.1097 $[M+Na]^+$, found 273.1113.

For 8-membered fused ring acyl radical precursor 9a

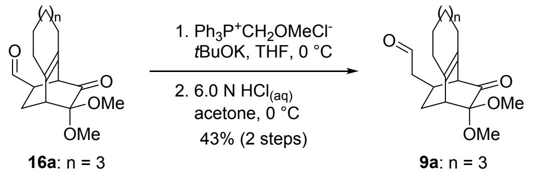

1.0 M *t*BuOK in THF (3.1 mL, 3.10 mmol, 3.0 eq.) was added to a two-neck round-bottom flask charged with (methoxymethyl)triphenylphosphonium chloride (1.224 g, 3.57 mmol, 3.5 eq.) at 0 °C. After stirring at 0 °C for 10 min, a solution of Diels-Alder adduct **16a** (0.299 g, 1.02 mmol) in THF (7.1 mL) was added. The reaction mixture was warmed to room temperature and stirred for 1 h, then diluted with water and extracted with DCM. The combined organic extracts were dried over NaSO$_4$, filtered, and concentrated *in vacuo*. The crude residue was purified by silica gel column chromatography (0% to 10% EtOAc/hexane) to afford a E/Z mixture of methyl enol ether intermediate (0.157 g, 48% yield).

6.0 N HCl (1.6 mL) was added dropwise to a solution of methyl enol ether intermediate (0.157 g, 0.49 mmol) in acetone (4.9 mL) at 0 ℃. The reaction mixture was warmed to room temperature and stirred for 25 min, then diluted with water and extracted with DCM. The combined organic extracts were dried over $NaSO_4$, filtered, and concentrated *in vacuo*. The crude residue was purified by silica gel column chromatography (0% to 15% EtOAc/hexane) to afford aldehyde **9a** as colorless oil (0.135 g, 90% yield).

^{1}H NMR (300 MHz, $CDCl_3$): δ 9.73 (t, J = 1.5 Hz, 1H), 3.36 (s, 3H), 3.31 (s, 3H), 2.82 (dd, J = 2.7, 2.7 Hz, 1H), 2.80 (d, J = 1.5 Hz, 1H), 2.74-2.61 (m, 1H), 2.48-2.34 (m, 4H), 2.32 (ddd, J = 12.3, 9.0, 2.7 Hz, 1H), 2.24-2.13 (m, 1H), 2.02 (ddd, J = 13.2, 4.2, 4.2 Hz, 1H), 1.70-1.44 (m, 6H), 1.44-1.30 (m, 2H), 0.90 (ddd, J = 12.3, 6.0, 2.7 Hz, 1H).
^{13}C NMR (75 MHz, $CDCl_3$): δ 201.4, 200.7, 141.2, 128.8, 93.9, 58.1, 49.8, 49.7, 49.7, 44.5, 31.5, 30.6, 28.3, 28.2, 28.0, 27.8, 26.1, 24.9.
IR (neat): 3446, 2926, 2850, 2721, 1731, 1558, 1540, 1506, 1463, 1447, 1388, 1359, 1257, 1209, 1163, 1136, 1126, 1108, 1070, 1049, 1010, 988, 936 cm^{-1}.
HRMS (ESI): calculated for $C_{18}H_{26}NaO_4$ 329.1723 $[M+Na]^+$, found 329.1731.

For 7-membered fused ring acyl radical precursor 9b

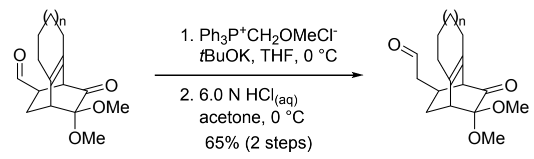

1.0 M *t*BuOK in THF (6.9 mL, 6.90 mmol, 3.0 equiv.) was added to a two-neck round-bottom flask charged with (methoxymethyl)triphenylphosphonium chloride (2.763 g, 8.06 mmol, 3.5 equiv.) at 0 ℃. After stirring at 0 ℃ for 7 min, a solution of Diels-Alder adduct **16b** (0.641 g, 2.30 mmol) in THF (16.1 mL) was added. The reaction mixture was warmed to room temperature and stirred for 50 min, then diluted with water and extracted with DCM. The combined organic extracts were dried over $NaSO_4$, filtered, and concentrated *in vacuo*. The crude residue was purified by silica gel column chromatography (0% to 10% EtOAc/hexane) to afford a E/Z mixture of methyl enol ether intermediate (0.587 g, 83% yield).

6.0 N HCl (3.4 mL) was added dropwise to a solution of methyl enol ether intermediate (0.311 g, 1.02 mmol) in acetone (10.2 mL) at 0 ℃. The reaction mixture was warmed to room temperature and stirred for 40 min, then quenched with sat. $NaHCO_3$ (30mL) and extracted with DCM. The combined organic extracts were dried over $NaSO_4$, filtered, and concentrated *in vacuo*. The crude residue was purified by

silica gel column chromatography (0% to 25% EtOAc/hexane) to afford aldehyde **9b** as colorless oil (0.233 g, 78% yield).

^{1}H NMR (400 MHz, $CDCl_3$): δ 9.68 (t, *J* = 1.6 Hz, 1H), 3.28 (s, 3H), 3.26 (s, 3H), 2.76 (t, *J* = 2.8 Hz, 1H), 2.72 (d, *J* = 2.0 Hz, 1H), 2.63-2.54 (m, 1H), 2.41-2.27 (m, 4H), 2.24 (ddd, *J* = 12.4, 9.2, 2.8 Hz, 1H), 2.20-2.09 (m, 2H), 1.71-1.57 (m, 3H), 1.57-1.47 (m, 3H), 0.87 (ddd, *J* = 12.4, 5.2, 2.8 Hz, 1H).
^{13}C NMR (75 MHz, $CDCl_3$): δ 201.6, 200.6, 142.9, 130.9, 93.9, 60.0, 49.9, 49.7, 49.7, 45.9, 34.3, 34.2, 30.3, 27.9, 27.7, 26.6, 26.5.
IR (neat): 3443, 2924, 2851, 2835, 2722, 1732, 1448, 1406, 1388, 1361, 1316, 1277, 1260, 1211, 1193, 1140, 1111, 1078, 1051, 1012, 990, 963, 947, 907 cm^{-1}.
HRMS (ESI): calculated for $C_{17}H_{24}NaO_4$ 315.1567 $[M+Na]^+$, found 315.1581.

For 6-membered fused ring acyl radical precursor 9c

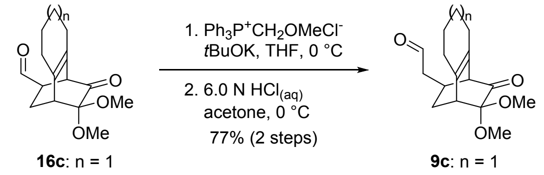

1.0 M *t*BuOK in THF (4.0 mL, 4.00 mmol, 3.0 equiv.) was added to a two-neck round-bottom flask charged with (methoxymethyl)triphenylphosphonium chloride (1.584 g, 4.62 mmol, 3.5 equiv.) at 0 °C. After stirring at 0 °C for 15 min, a solution of Diels-Alder adduct **16c** (0.349 g, 1.32 mmol) in THF (9.2 mL) was added. The reaction mixture was warmed to room temperature and stirred for 20 min, then diluted with water and extracted with DCM. The combined organic extracts were dried over $NaSO_4$, filtered, and concentrated *in vacuo*. The crude residue was purified by silica gel column chromatography (0% to 15% EtOAc/hexane) to afford a E/Z mixture of methyl enol ether intermediate (0.331 g, 86% yield).

6.0 N HCl (7.2 mL) was added dropwise to a solution of methyl enol ether intermediate (0.631 g, 2.16 mmol) in acetone (21.6 mL) at 0 °C. The reaction mixture was warmed to room temperature and stirred for 20 min, then diluted with water and extracted with DCM. The combined organic extracts were dried over $NaSO_4$, filtered, and concentrated *in vacuo*. The crude residue was purified by silica gel column chromatography (0% to 30% EtOAc/hexane) to afford aldehyde **9c** as colorless oil (0.538 g, 90% yield).

^{1}H NMR (300 MHz, $CDCl_3$): δ 9.75-9.70 (m, 1H), 3.33 (s, 3H), 3.32 (s, 3H), 2.75 (dd, *J* = 2.4, 2.4 Hz, 1H), 2.73-2.63 (m, 2H), 2.46-2.26 (m, 3H), 2.18-2.01 (m, 3H), 2.00-1.87 (m, 1H), 1.78-1.59 (m, 4H), 0.92 (ddd, *J* = 12.9, 5.4, 2.4 Hz, 1H).

^{13}C NMR (75 MHz, $CDCl_3$): δ 201.5, 200.8, 139.2, 127.3, 94.5, 57.0, 50.2, 49.9, 49.6, 42.6, 28.7, 28.3, 28.2, 27.7, 22.7, 22.5.
HRMS (ESI): calculated for $C_{16}H_{22}NaO_4$ 301.1410 $[M+Na]^+$, found 301.1410.

For 5-membered fused ring acyl radical precursor 9d

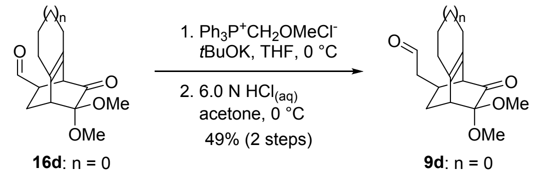

1.0 M *t*BuOK in THF (14.0 mL, 14.00 mmol, 3.0 equiv.) was added to a two-neck round-bottom flask charged with (methoxymethyl)triphenylphosphonium chloride (5.591 g, 16.31 mmol, 3.5 equiv.) at 0 °C. After stirring at 0 °C for 15 min, a solution of Diels-Alder adduct **16d** (1.167 g, 4.66 mmol) in THF (32.6 mL) was added. The reaction mixture was warmed to room temperature and stirred for 38 min, then diluted with brine and extracted with DCM. The combined organic extracts were dried over $NaSO_4$, filtered, and concentrated *in vacuo*. The crude residue was purified by silica gel column chromatography (0% to 10% EtOAc/hexane) to afford a E/Z mixture of methyl enol ether intermediate (0.847 g, 65% yield).

6.0 N HCl (0.9 mL) was added to a solution of enol ether S1d (0.077 g, 0.28 mmol) in acetone (2.8 mL) at 0 °C. The reaction mixture was warmed to room temperature and stirred for 25 min, then quenched with sat. $NaHCO_3$ (15mL) and extracted with EtOAc. The combined organic extracts were dried over $NaSO_4$, filtered, and concentrated *in vacuo*. The crude residue was purified by silica gel column chromatography (0% to 25% EtOAc/hexane) to afford aldehyde **9d** as colorless oil (0.055 g, 75% yield).

^{1}H NMR (400 MHz, $CDCl_3$): δ 9.71 (t, J = 1.6 Hz, 1H), 3.34 (s, 3H), 3.31 (s, 3H), 3.10 (t, J = 2.8 Hz, 1H), 3.07 (d, J = 2.0 Hz, 1H), 2.79-2.70 (m, 1H), 2.56-2.41 (m, 3H), 2.41-2.24 (m, 4H), 2.13-2.02 (m, 1H), 2.02-1.92 (m, 1H), 0.88 (ddd, J = 12.8, 5.2, 2.8 Hz, 1H).
^{13}C NMR (100 MHz, $CDCl_3$): δ 200.7, 200.5, 145.1, 133.8, 94.4, 53.5, 50.0 (2C), 49.6, 38.6, 33.6, 33.1, 28.9, 28.4, 23.4.
IR (neat): 3442, 2950, 2894, 2845, 2724, 1731, 1448, 1406, 1388, 1361, 1208, 1194, 1169, 1149, 1132, 1108, 1049, 1007, 988, 904 cm^{-1}.
HRMS (ESI): calculated for $C_{15}H_{20}NaO_4$ 287.1254 $[M+Na]^+$, found 287.1255.

2.3 The thiol-mediated acyl radical reaction of radical precursors 9a-d

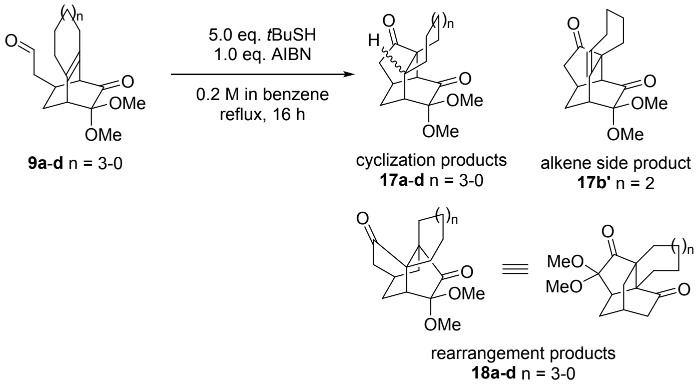

General procedure for the thiol-mediated acyl reaction

A solution of aldehyde **9a-d** (1.0 eq.), *t*BuSH (5.0 eq.), and AIBN (1.0 eq.) in benzene (2.0 mL) was heated to reflux and stirred under argon atmosphere for 16 h. The reaction mixture was cooled to room temperature, and the solvent was removed *in vacuo*. The crude residue was purified by silica gel column chromatography (0% to 25% EtOAc/hexane) to afford cyclized product **17a-d** and/or rearranged product **18a-d**.

Thiol-mediated acyl radical reaction of precursor 9a with 8-membered fused ring

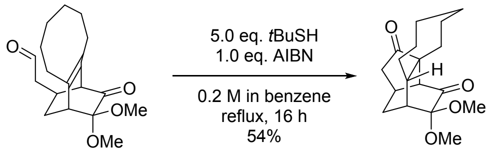

9a **17a**

The above general procedure was followed when aldehyde **9a** (0.119 g, 0.39 mmol) reacted with *t*BuSH (0.22 mL, 1.95 mmol, 5.0 eq.) and AIBN (0.064 g, 0.39 mmol, 1.0 eq.) in benzene (2.0 mL) to afford cyclized product **17a** as white solid (64 mg, 54% yield).

Mp: 73.2-74.6 ℃.

^{1}H NMR (300 MHz, $CDCl_3$): δ 3.37 (s, 3H), 3.35 (s, 3H), 2.74-2.61 (m, 2H), 2.46-2.32 (m, 1H), 2.32-2.25 (m, 2H), 2.21 (d, J = 3.9 Hz, 1H), 2.09-1.94 (m, 2H), 1.94-1.68 (m, 4H), 1.68-1.44 (m, 5H), 1.42-1.19 (m, 3H).

^{13}C NMR (75 MHz, $CDCl_3$): δ 218.7 (C), 207.0 (C), 96.2 (C), 61.2 (CH), 57.0 (C), 49.8 (CH_3), 49.7 (CH_3), 47.9 (CH_2), 41.5 (CH), 36.8 (CH_2), 35.6 (CH), 31.9 (CH_2), 30.0 (CH), 29.7(CH_2), 26.3 (CH_2), 26.1 (CH_2), 22.6 (CH_2), 22.4 (CH_2).

IR (neat): 3446, 2922, 2873, 2852, 1738, 1732, 1469, 1462, 1456, 1410, 1366, 1337, 1311, 1282, 1233, 1210, 1191, 1170, 1132, 1110, 1077, 1058, 1050, 1014, 988, 976, 944 cm^{-1}.

HRMS (ESI): calculated for $C_{18}H_{26}NaO_4$ 329.1723 [M+Na]$^+$, found 329.1740.

Thiol-mediated acyl radical reaction of precursor 9b with 7-membered fused ring

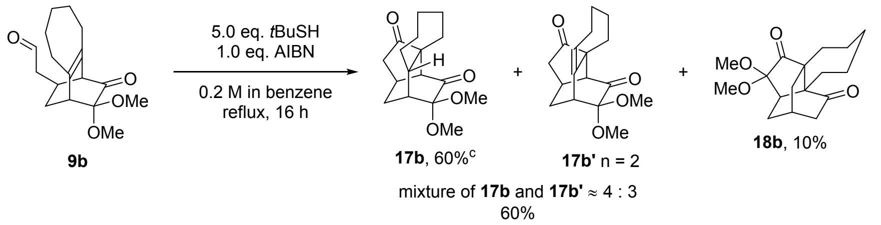

The above general procedure was followed when aldehyde **9b** (0.233g, 0.80 mmol) reacted with *t*BuSH (0.45 mL, 3.98 mmol, 5.0 eq.) and AIBN (0.131 g, 0.80 mmol, 1.0 eq.) in benzene (4.0 mL) to afford a mixture of cyclized product **17b** and related alkene side product **17b'** (4:3) as white solid (139 mg, 60% yield), and rearranged product **18b** as white solid (24 mg, 10% yield).

For **17b**:

^{1}H NMR (300 MHz, $CDCl_3$): δ 3.28 (s, 3H), 3.23 (s, 3H), 2.78-2.57 (m, 1H), 2.57-2.16 (m, 6H), 1.98-1.68 (m, 4H), 1.68-1.36 (m, 4H), 1.36-0.94 (m, 3H).

^{13}C NMR (100 MHz, $CDCl_3$): δ 219.8 (C), 205.6 (C), 95.6 (C), 59.2 (CH), 55.7 (C), 49.5 (CH_3), 48.8 (CH_3), 48.0 (CH_2), 46.8 (CH), 40.1 (CH), 33.8 (CH_2), 32.7 (CH_2), 31.3 (CH_2), 31.0 (CH_2), 30.8 (CH_2), 29.3 (CH), 23.2 (CH_2).

HRMS (ESI): calculated for $C_{17}H_{24}NaO_4$ 315.1567 [M+Na]$^+$, found 315.1564.

For **17b'**:

^{1}H NMR (300 MHz, $CDCl_3$): δ 5.95 (dd, J = 9.3, 4.8 Hz, 1H), 3.33 (s, 3H), 3.30 (s, 3H), 2.79-2.61 (m, 2H), 2.61-2.46 (m, 1H), 2.46-2.18 (m, 5H), 2.08-1.94 (m, 1H), 1.79-1.53 (m, 3H), 1.53-1.34 (m, 2H), 1.33-0.99 (m, 1H).

^{13}C NMR (100 MHz, $CDCl_3$): δ 210.8 (C), 206.5 (C), 134.4 (C), 133.7 (CH), 95.5 (C), 59.2 (CH), 58.4 (C), 49.6 (CH_3), 49.5 (CH_3), 46.5 (CH_2), 45.7 (CH), 31.3 (CH_2), 30.3 (CH), 29.9 (CH_2), 26.5 (CH_2), 26.1 (CH_2), 24.6 (CH_2).

HRMS (ESI): calculated for $C_{17}H_{22}NaO_4$ 313.1410 [M+Na]$^+$, found 313.1416.

For **18b**:

^{1}H NMR (400 MHz, $CDCl_3$): δ 3.36 (s, 3H), 3.31 (s, 3H), 2.39 (ddd, J = 18.0, 4.0, 2.4 Hz, 1H), 2.24 (d, J = 10.0 Hz, 1H), 2.21-2.02 (m, 5H), 1.98 (ddd, J = 14.4, 3.2, 3.2 Hz, 1H), 1.77-1.46 (m, 7H), 1.38-1.25 (m, 3H).

^{13}C NMR (100 MHz, $CDCl_3$): δ 214.0 (C), 211.5 (C), 101.3 (C), 56.0 (C), 54.9 (C), 50.3 (CH_3), 50.1 (CH_3), 47.8 (CH), 47.4 (CH_2), 41.9 (CH_2), 35.8 (CH_2), 29.2 (CH_2), 26.9 (CH_2), 26.9 (CH), 26.5 (CH_2), 22.5 (CH_2), 22.0 (CH_2).
IR (neat): 3484, 3408, 2927, 2869, 1749, 1714, 1461, 1409, 1343, 1261, 1206, 1193, 1147, 1117, 1069, 1055, 1035, 1025, 1003, 948, 934, 915, 866 cm^{-1}.
HRMS (ESI): calculated for $C_{17}H_{24}NaO_4$ 315.1567 $[M+Na]^+$, found 315.1578.

Thiol-mediated acyl radical reaction of precursor 9c with 6-membered fused ring

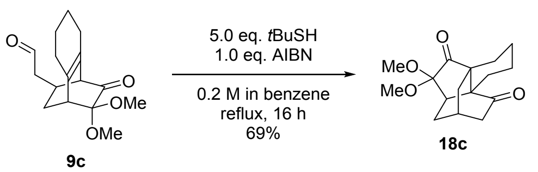

The above general procedure was followed when aldehyde **9c** (0.517g, 1.86 mmol) reacted with *t*BuSH (1.05 mL, 9.30 mmol, 5.0 eq.) and AIBN (0.305 g, 1.86 mmol, 1.0 eq.) in benzene (9.3 mL) to afford rearranged product **18c** as white solid (357 mg, 69% yield).

Mp: 103.6-105.0 ℃.
^{1}H NMR (600 MHz, $CDCl_3$): δ 3.39 (s, 3H), 3.35 (s, 3H), 2.38 (ddd, J = 18.0, 3.0, 3.0 Hz, 1H), 2.28-2.22 (m, 2H), 2.22-2.13 (m, 4H), 1.93 (ddd, J = 14.4, 3.0, 3.0 Hz, 1H), 1.67-1.61 (m, 1H), 1.58-1.54 (m, 1H), 1.54-1.41 (m, 3H), 1.26-1.19 (m, 1H), 1.10-0.99 (m, 2H).
^{13}C NMR (75 MHz, $CDCl_3$): δ 213.5 (C), 210.1 (C), 101.4 (C), 51.7 (C), 50.6 (C), 50.2 (CH_3, CH_3), 46.6 (CH_2), 44.5 (CH), 38.8 (CH_2), 32.2 (CH_2), 27.4 (CH_2), 26.6 (CH), 25.5 (CH_2), 22.5 (CH_2), 22.3 (CH_2).
IR (neat): 3488, 3415, 2936, 2869, 2838, 1751, 1716, 1463, 1453, 1407, 1344, 1283, 1241, 1213, 1179, 1149, 1130, 1107, 1078, 1066, 1050, 1039, 984, 942, 933, 920, 890, 874, 850 cm^{-1}.
HRMS (ESI): calculated for $C_{16}H_{22}NaO_4$ 301.1410 $[M+Na]^+$, found 301.1421.

Thiol-mediated acyl radical reaction of precursor 9d with 5-membered fused ring

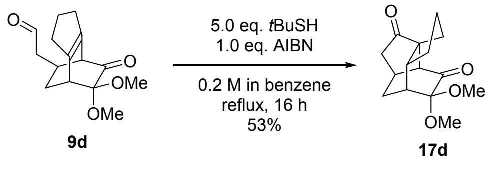

The above general procedure was followed when aldehyde **9d** (0.055g, 0.21 mmol) reacted with *t*BuSH (0.12 mL, 1.05 mmol, 5.0 eq.) and AIBN (0.034 g, 0.21 mmol, 1.0 eq.) in benzene (1.1 mL) to afford cyclized product **17d** as white solid (29 mg, 53%

yield).

Mp: 95.5-97.0 ℃.

^{1}H NMR (300 MHz, $CDCl_3$): δ 3.33 (s, 3H), 3.27 (s, 3H), 2.80-2.71 (m, 1H), 2.56 (ddd, *J* = 13.2, 10.2, 3.0 Hz, 1H), 2.45 (d, *J* = 3.6 Hz, 1H), 2.41 (dt, *J* = 3.0, 3.0 Hz, 1H), 2.30 (d, *J* = 3.3 Hz, 2H), 2.12 (td, *J* = 8.4, 3.0 Hz, 1H), 2.06-1.89 (m, 2H), 1.83-1.69 (m, 1H), 1.67-1.48 (m, 2H), 1.36-1.23 (m, 1H), 1.19 (ddd, *J* = 13.2, 3.0, 2.1 Hz, 1H).

^{13}C NMR (75 MHz, $CDCl_3$): δ 217.3 (C), 205.9 (C), 95.2 (C), 61.6 (C), 55.7 (CH), 49.2 (CH_3), 49.1 (CH_3), 46.7 (CH_2), 46.6 (CH), 36.4 (CH), 31.7 (CH_2), 31.0 (CH_2), 30.1 (CH_2), 29.7 (CH), 26.4 (CH_2).

IR (neat): 3546, 3447, 2948, 2882, 2835, 1738, 1732, 1636, 1456, 1410, 1368, 1338, 1318, 1285, 1267, 1182, 1131, 1092, 1062, 1046, 987, 967, 930 cm^{-1}.

HRMS (ESI): calculated for $C_{15}H_{20}NaO_4$ 287.1254 $[M+Na]^+$, found 287.1270.

2.4 The synthesis of dimethyl model study.

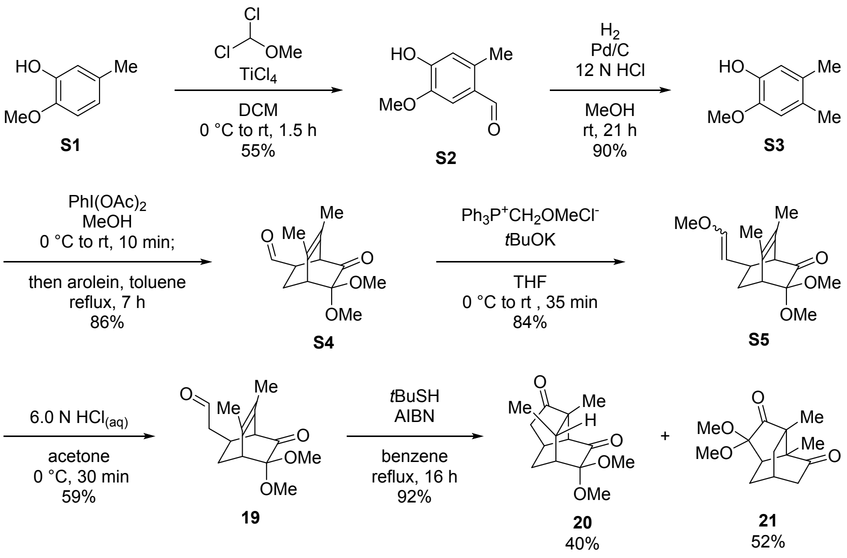

For compound S2

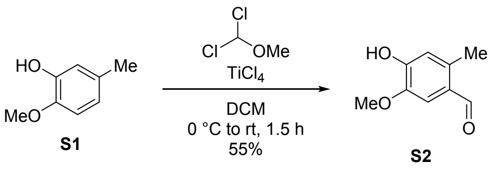

Titanium (IV) chloride (8.0 mL, 72.80 mmol, 2.0 equiv.) and dichloromethyl methyl ether (5.2 mL, 57.50 mmol, 1.6 equiv.) were added to a solution of 2-methoxy-5-methylphenol **S1** (5.0 g, 36.19 mmol) in dichloromethane at 0 ℃. The reaction mixture was then warmed to room temperature and stirred for 1.5 h. After the reaction has finished, the mixture was poured into ice water with precipitate forming. The precipitate was then collected through filtration and washed by EtOAc to afford light brown solid **S2** (3.323 g, 55% yield). The spectroscopic data was identical to the one reported in the literature[6].

For compound S3

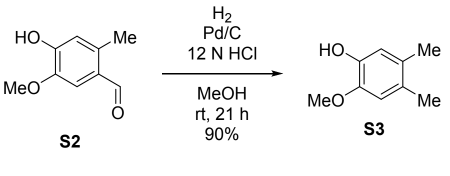

10% Pd on carbon (1.058 g, 0.99 mmol, 5 mol%) and 12 N HCl (0.05 mL, 0.60 mmol, 3 mol%) were added to a solution of the **S2** (3.305 g, 19.89 mmol) in MeOH (199 mL). The flask was evacuated under vacuum and H_2 gas was introduced via balloon. After stirring at room temperature for 21 h, the reaction mixture was filtered through Celite® and the filtrate was concentrated *in vacuo*. The crude residue was purified by silica gel column chromatography (20% EtOAc/hexane) to afford **S3** as

white solid (2.736 g, 90% yield).

^{1}H NMR (400 MHz, $CDCl_3$): δ 6.73 (s, 1H), 6.66 (s, 1H), 3.86 (s, 3H), 2.20 (s, 3H), 2.17 (s, 3H).
^{13}C NMR (100 MHz, $CDCl_3$): δ 144.3, 143.3, 129.1, 127.5, 116.0, 112.6, 56.1, 19.3, 19.0.
IR (neat): 3390, 2962, 2934, 2848, 1619, 1520, 1460, 1370, 1354, 1285, 1272, 1228, 1176, 1096, 1013, 994 cm^{-1}.
HRMS (ESI): calculated for $C_9H_{12}NaO_2$ 175.0730 $[M+Na]^+$, found 175.0734.

For compound S4

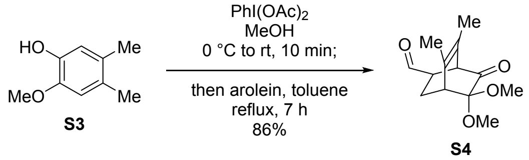

PhI(OAc)$_2$ (7.442 g, 23.11 mmol, 1.3 equiv.) was added to a solution of **S3** (2.705 g, 17.77 mmol) in methanol (89 mL) at 0 °C. After stirring at room temperature for 10 min, acrolein (23.8 mL, 356.19 mmol, 20 equiv.) and toluene (592 mL) were added. The reaction was heated to reflux and stirred for 7 h. The reaction mixture was cooled to room temperature, and the solvent was removed *in vacuo*. The crude residue was purified by silica gel column chromatography (hexane to 33% EtOAc/hexane) to afford Diels-Alder adduct **S4** as colorless oil (3.660 g, 86% yield).

^{1}H NMR (400 MHz, $CDCl_3$): δ 9.56 (d, J = 0.8 Hz, 1H), 3.36 (s, 3H), 3.33 (s, 3H), 3.28 (d, J = 2.0 Hz, 1H), 3.01 - 2.94 (m, 1H), 2.91 (dd, J = 2.8, 2.8 Hz, 1H), 2.13 (ddd, J = 12.8, 10.0, 2.8 Hz, 1H), 1.82 (q, J = 0.8 Hz, 3H), 1.78 (ddd, J = 12.8, 6.0, 2.8 Hz, 1H), 1.74 (q, J = 0.8 Hz, 3H).
^{13}C NMR (100 MHz, $CDCl_3$): δ 200.2, 199.7, 136.7, 124.6, 94.8, 53.2, 50.2, 49.7, 46.8, 43.8, 21.3, 17.0, 16.8.
IR (neat): 3458, 2976, 2948, 2837, 1738, 1732, 1462, 1454, 1445, 1380, 1318, 1254, 1219, 1193, 1138, 1088, 1056, 1049, 970, 874, 868 cm^{-1}.
HRMS (ESI): calculated for $C_{13}H_{18}NaO_4$ 261.1097 $[M+Na]^+$, found 261.1106.

For compound S5

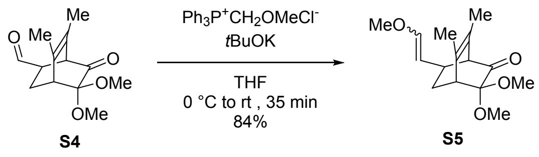

1.0 M *t*BuOK in THF (36 mL, 36.00 mmol, 3.0 equiv.) was added to a two-neck round-bottom flask charged with (methoxymethyl)triphenylphosphonium chloride (14.552 g, 42.45 mmol, 3.5 equiv.) at 0 ℃. After stirring at 0 ℃ for 10 min, a solution of Diels-Alder adduct **S4** (2.890 g, 12.13 mmol) in THF (85 mL) was added. The reaction mixture was warmed to room temperature and stirred for 35 min. It was then diluted with water and extracted with DCM. The combined organic extracts were dried over $NaSO_4$, filtered, and concentrated *in vacuo*. The crude residue was purified by silica gel column chromatography (0% to 25% EtOAc/hexane) to afford E/Z mixture (≈ 1.6:1.0) of enol ether **S5** as colorless oil (2.711 g, 84% yield).

^{1}H NMR (400 MHz, $CDCl_3$, *E*/*Z*-mixutures (1.6:1.0)): for ***E*-isomer** δ 6.33 (d, *J* = 12.4 Hz, 1H), 4.47 (dd, *J* = 12.4, 9.6 Hz, 1H), 3.48 (s, 3H), 3.34 (s, 3H), 3.31 (s, 3H), 2.77 - 2.74 (m, 1H), 2.72 (d, *J* = 2.0 Hz, 1H), 2.71 - 2.64 (m, 1H), 2.20 (ddd, *J* = 12.4, 9.2, 2.8, 1H), 1.86 (q, *J* = 0.8 Hz, 3H), 1.77 (q, *J* = 0.8 Hz, 3H), 1.07 (ddd, *J* = 12.4, 5.6, 2.8, 1H); for ***Z*-isomer** δ 5.80 (dd, *J* = 6.0, 0.8 Hz, 1H), 4.08 (dd, *J* = 9.2, 6.0 Hz, 1H), 3.58 (s, 3H), 3.33 (s, 3H), 3.32 (s, 3H), 3.27 - 3.18 (m, 1H), 2.78 (d, *J* = 2.0 Hz, 1H), 2.77 - 2.74 (m, 1H), 2.27 (ddd, *J* = 12.4, 9.2, 2.8, 1H), 1.84 (q, *J* = 0.8 Hz, 3H), 1.75 (q, *J* = 0.8 Hz, 3H), 1.01 (ddd, *J* = 12.4, 5.2, 2.8, 1H).
^{13}C NMR (100 MHz, $CDCl_3$): δ 202.2, 147.2, 145.7, 135.4, 135.1, 125.2, 124.8, 110.0, 106.2, 94.5, 94.5, 61.2, 59.5, 59.5, 56.0, 50.2, 50.1, 49.8, 49.7, 44.4, 44.3, 33.9, 29.6, 29.4, 29.4, 17.8, 17.1, 17.0.
IR (neat): 3626, 3446, 2947, 2857, 2834, 2042, 1738, 1731, 1668, 1660, 1651, 1462, 1454, 1392, 1335, 1312, 1262, 1248, 1229, 1207, 1133, 1112, 1096, 1046, 1003, 985, 964, 936 cm^{-1}.
HRMS (ESI): calculated for $C_{15}H_{22}NaO_4$ 289.1410 $[M+Na]^+$, found 289.1418.

For compound 19

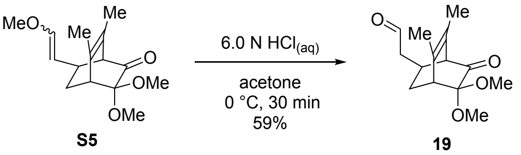

6.0 N HCl was added dropwisely to a solution of enol ether **S5** (0.800 g, 3.00 mmol) in acetone at 0 ℃. The reaction mixture was warmed to room temperature and stirred for 30 min. It was then diluted with water and extracted with DCM. The combined organic extracts were dried over $NaSO_4$, filtered, and concentrated *in vacuo*. The crude residue was purified by silica gel column chromatography (25% EtOAc/hexane) to afford aldehyde **19** as colorless oil (0.450 g, 59% yield).

^{1}H NMR (400 MHz, $CDCl_3$): δ 9.72 (t, *J* = 1.6 Hz, 1H), 3.34 (s, 3H), 3.31 (s, 3H), 2.78 (dd, *J* = 2.8, 2.8 Hz, 1H), 2.75 (d, *J* = 1.6 Hz, 1H), 2.72 - 2.63 (m, 1H), 2.39 (ddd, *J* = 16.4, 6.4, 1.6 Hz, 1H), 2.36 - 2.26 (m, 2H), 0.92 (ddd, *J* = 12.8, 5.6, 2.8 Hz, 1H).
^{13}C NMR (100 MHz, $CDCl_3$): δ 201.5, 200.6, 135.9, 124.3, 94.2, 58.2, 50.1, 49.7, 49.4, 44.0, 28.5, 27.8, 17.5, 17.0.
IR (neat): 3445, 2947, 2836, 2727, 1738, 1732, 1715, 1462, 1455, 1445, 1384, 1353, 1305, 1281, 1261, 1245, 1218, 1194, 1139, 1109, 1050, 986, 906, 871 cm^{-1}.
HRMS (ESI): calculated for $C_{14}H_{20}NaO_4$ 275.1254 $[M+Na]^+$, found 275.1263.

Thiol-mediated acyl radical reaction of precursor 19 with dimethyl group

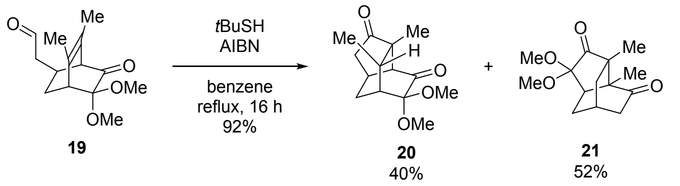

A solution of aldehyde **19** (0.44 g, 1.74 mmol), *t*BuSH (0.99 mL, 8.72 mmol, 5.0 equiv.), and AIBN (0.29 g, 1.74 mmol, 1.0 equiv.) in benzene (8.8 mL) was heated to reflux and stirred under argon atmosphere for 16 h. The reaction mixture was cooled to room temperature, and the solvent was removed *in vacuo*. The crude residue was purified by silica gel column chromatography (0% to 15% EtOAc/hexane) to afford cyclized product **20** (0.18 g, 40% yield), and rearranged product **21** as white solid (0.23 g, 52% yield).

For cyclized product **20**:
^{1}H NMR (400 MHz, $CDCl_3$): δ 3.34 (s, 3H), 3.29 (s, 3H), 2.77 - 2.71 (m, 1H), 2.47 - 2.28 (m, 4H), 2.23 - 2.20 (m, 1H), 1.96 (qd, *J* = 5.7, 2.1 Hz, 1H), 1.35 (ddd, *J* = 13.6, 3.6, 1.2 Hz, 1H), 1.14 (d, *J* = 7.6 Hz, 3H), 1.01 (s, 3H).
^{13}C NMR (75 MHz, $CDCl_3$): δ 216.8 (C), 206.2 (C), 95.9 (C), 57.8 (CH), 52.8 (C), 49.5 (CH_3), 49.4 (CH_3), 46.2 (CH_2), 40.1 (CH), 36.4 (CH), 32.3 (CH_2), 30.5 (CH), 16.1 (CH_3), 14.9 (CH_3).
IR (neat) 2951, 2937, 2873,2837, 1729, 1464, 1404, 1384, 1334, 1285, 1272, 1256, 1225, 1198, 1176, 1133, 1120, 1093, 1085, 1044, 1024, 1007, 954 cm^{-1}.
HRMS (ESI): calculated for $C_{14}H_{20}NaO_4$ 275.1254 $[M+Na]^+$, found 275.1259.

For rearranged product **21**:
Mp 51.4-55.1 °C.
^{1}H NMR (300 MHz, $CDCl_3$): δ 3.35 (s, 3H), 3.33 (s, 3H), 2.41 - 2.13 (m, 5H), 1.98 (dd, *J* = 14.7, 3.3 Hz, 1H), 1.74 (ddd, *J* = 12.0, 3.0, 3.0 Hz, 1H), 1.50 (dd, *J* = 14.7, 3.0 Hz, 1H), 1.06 (s, 3H), 0.95 (s, 3H).

^{13}C NMR (75 MHz, $CDCl_3$): δ 213.2 (C), 211.0 (C), 101.7 (C), 52.4 (C), 50.7 (C), 50.5 (CH_3), 50.3 (CH_3), 46.2 (CH_2), 45.4 (CH), 39.4 (CH_2), 27.6 (CH_2), 26.7 (CH), 18.3 (CH_3), 15.7 (CH_3).

IR (neat) 2973, 2942, 2873, 2836, 1754, 1722, 1715, 1462, 1454, 1407, 1387, 1343, 1268, 1218, 1205, 1134, 1116,1102,1059, 1030, 987, 960, 929, 907 cm^{-1}.

HRMS (ESI): calculated for $C_{14}H_{20}NaO_4$ 275.1254 $[M+Na]^+$, found 275.1263.

3. Computational Details

All calculations were performed using the B3LYP[7] functional in combination with Grimme's D3 dispersion correction with BJ-damping.[8] The double-ζ quality Pople basis set 6-31G**[9] was employed for geometry optimization and vibrational frequency calculations. To avoid errors in vibrational entropies caused by small frequencies, any frequencies below 50 cm^{-1} were treated as 50 cm^{-1} for thermal corrections.[10] The solvation free energies (ΔG_{sol}) in benzene were estimated using CPCM[11] implicit solvation model, performing single point calculation under the same computational level as the geometry optimization with gas-phase optimized structures. For more accurate electronic energies, the triple-ζ quality basis set 6-311++G**[12] was used to calculate the single point energy using the optimized structure.

Since molecules are treated as ideal gases when calculating entropies, the entropy values are often overestimated. Therefore, we employed a procedure proposed by Wertz[13] and Cooper and Ziegler[14] to estimate the entropy in solution.

The entropy in solution (S_s) can be calculated using the following equations:

$$\Delta S_1 = Rln(V^s_{m,liq}/V_{m,gas})$$

$$\Delta S_2 = Rln(V^{\circ}_m/V^s_{m,liq})$$

$$\alpha = \frac{S^{\circ,s}_{liq} - \left(S^{\circ,s}_{gas} + \Delta S_1\right)}{\left(S^{\circ,s}_{gas} + \Delta S_1\right)}$$

$$\Delta S_{sol} = \left[\Delta S_1 + \alpha\left(S_g + \Delta S_1\right) + \Delta S_2\right]$$

$$= \left[\left(-11.16\; cal/mol\cdot K\right) - 0.22\left(S_g - 11.16\; cal/mol\cdot K\right) + 4.81\; cal/mol\cdot K\right]$$

$$S_s = S_g + \Delta S_{sol}$$

In the above equations, S_g and ΔS_{sol} represent the gas-phase entopy of the solute and the solvation entopy, repectively. $S^{o,s}_{liq}$, $S^{o,s}_{gas}$ denote the standard entropies of the solvent in the liquid and gas phase, respectively. For benzene, their values are 173.4 and 269.2 $J/mol^{-1}K^{-1}$, respectively. $V^s_{m,liq}$ is the molar volume of benzene, which is 0.08907 L/mol. $V_{m,gas}$ represents the molar volume of the ideal gas at 298 K (24.45 L/mol). V^o_m denotes the molar volume of the solution under standard conditions (1 L/mol).

The Gibbs free energies can be expressed by the following equation:

$$G = E_{elec} + \Delta G_{sol} + ZPE + 4RT + H_{vib} - TS_s$$

where E_{elec}, ΔG_{sol}, ZPE, and H_{vib} represent the electronic energy, solvation free energy, zero-point energy, and enthalpy contribution from vibration, respectively. The temperature, T, is set to 298.15 K.

Free Energy Profiles for 9a-d

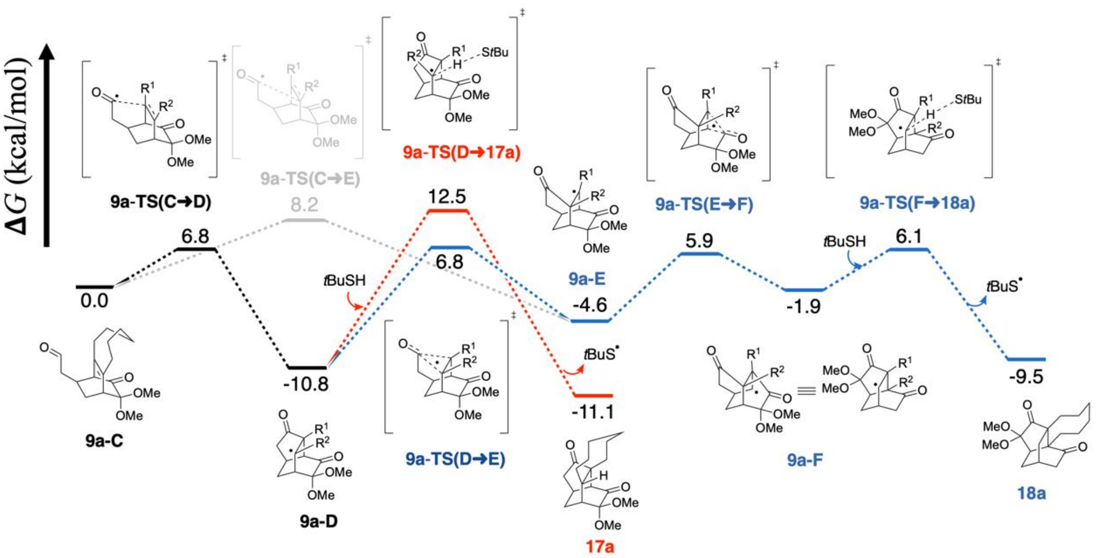

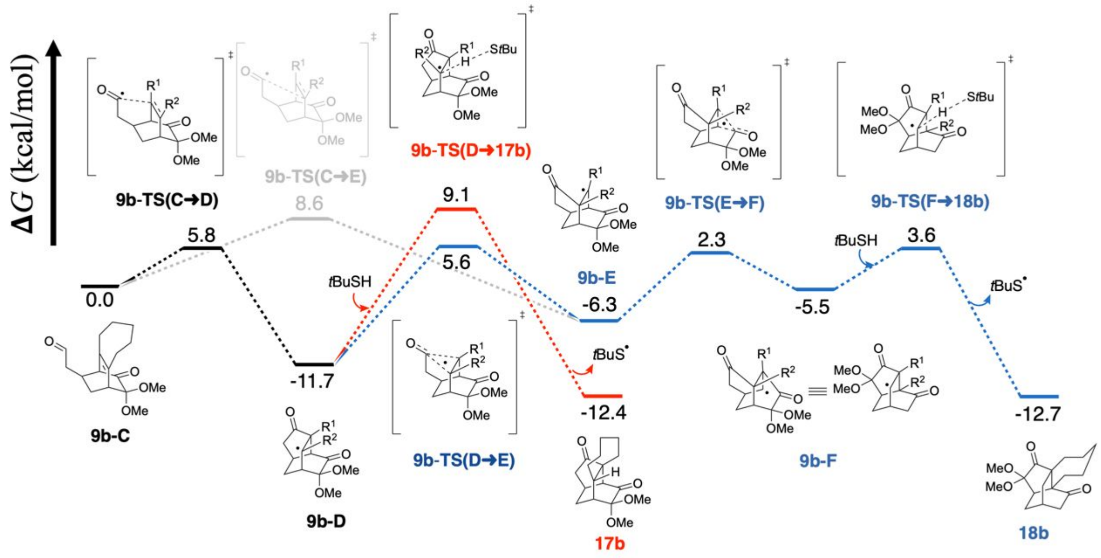

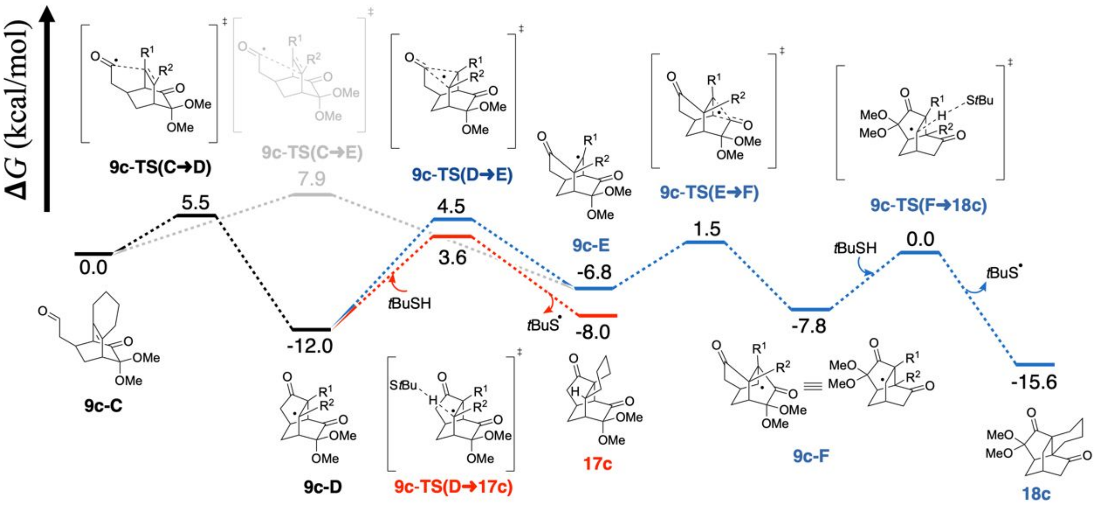
ΔG (kcal/mol)
9c-TS(C→D)
9c-TS(C→E)
9c-TS(D→E)
9c-E
9c-TS(E→F)
9c-TS(F→18c)
7.9
5.5
4.5
3.6
1.5
0.0
0.0
-6.8
-8.0
-7.8
-12.0
-15.6
tBuSH
tBuS•
tBuSH
tBuS•
9c-C
9c-D
9c-TS(D→17c)
17c
9c-F
18c

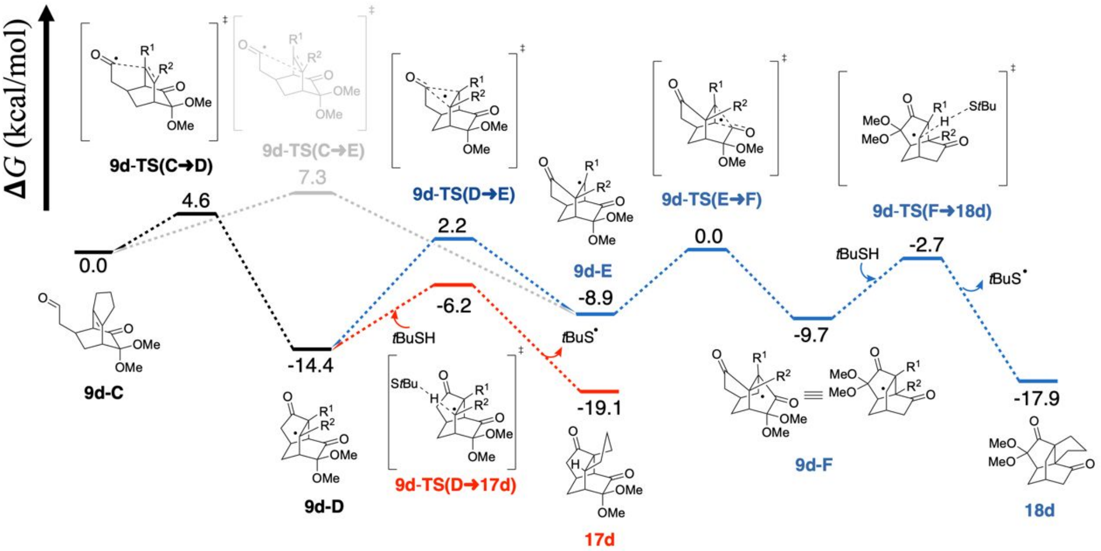
ΔG (kcal/mol)
9d-TS(C→D)
9d-TS(C→E)
9d-TS(D→E)
9d-E
9d-TS(E→F)
9d-TS(F→18d)
7.3
4.6
2.2
0.0
0.0
-2.7
-6.2
-8.9
-9.7
-14.4
-19.1
-17.9
tBuSH
tBuS•
tBuSH
tBuS•
9d-C
9d-D
9d-TS(D→17d)
17d
9d-F
18d

Table S1. Energy components contributing to the Gibbs free energies for each calculated species. At least two possible conformations were calculated for every fused-ring species, including their intermediates and products (entries **9a-d**). The most stable local minima and saddle points were considered. All values are given in Hartree units.

**Species**	E_{elec}	ΔG_{sol}	$ZPE+4RT+H_{vib}-TS_s$	**G**
*t*BuSH	-556.7438	-0.0019	0.1116	-556.6341
*t*BuS·	-556.0984	-0.0016	0.1017	-555.9982
19C	-846.0420	-0.0054	0.2787	-845.7687
19-TS(C->D)	-846.0344	-0.0054	0.2796	-845.7602
19D	-846.0659	-0.0059	0.2824	-845.7894
19-TS(C->E)	-846.0323	-0.0055	0.2810	-845.7568
19-TS(D->E)	-846.0416	-0.0059	0.2828	-845.7647
19E	-846.0568	-0.0059	0.2825	-845.7802
19-TS(E->F)	-846.0430	-0.0063	0.2833	-845.7660
19F	-846.0584	-0.0058	0.2827	-845.7815
19-TS(F->21)	-1402.8033	-0.0071	0.4076	-1402.4028
21	-846.7219	-0.0057	0.2976	-846.4300
19-TS(D->20)	-1402.8001	-0.0071	0.4088	-1402.3983
20	-846.7210	-0.0056	0.2980	-846.4286
9a-C	-1002.1465	-0.0051	0.3746	-1001.7770
9a-TS(C->D)	-1002.1358	-0.0051	0.3747	-1001.7662
9a-D	-1002.1660	-0.0056	0.3774	-1001.7942
9a-TS(C->E)	-1002.1349	-0.0052	0.3762	-1001.7639
9a-TS(D->E)	-1002.1390	-0.0055	0.3782	-1001.7662
9a-E	-1002.1568	-0.0056	0.3780	-1001.7844
9a-TS(E->F)	-1002.1385	-0.0062	0.3770	-1001.7676
9a-F	-1002.1509	-0.0057	0.3765	-1001.7801
9a-TS(F->18a)	-1558.8971	-0.0066	0.5022	-1558.4014
18a	-1002.8151	-0.0053	0.3922	-1002.4281
9a-TS(D->17a)	-1558.8896	-0.0057	0.5040	-1558.3913
17a	-1002.8177	-0.0051	0.3923	-1002.4306
9b-C	-962.8167	-0.0054	0.3440	-962.4781
9b-TS(C->D)	-962.8098	-0.0051	0.3460	-962.4689
9b-D	-962.8401	-0.0054	0.3489	-962.4967

9b-TS(C->E)	-962.8062	-0.0052	0.3471	-962.4644
9b-TS(D->E)	-962.8131	-0.0056	0.3494	-962.4693
9b-E	-962.8320	-0.0056	0.3494	-962.4881
9b-TS(E->F)	-962.8175	-0.0062	0.3492	-962.4745
9b-F	-962.8298	-0.0056	0.3486	-962.4868
9b-TS(F->18b)	-1519.5720	-0.0077	0.4731	-1519.1065
18b	-963.4922	-0.0055	0.3635	-963.1342
9b-TS(D->17b)	-1519.5675	-0.0057	0.4755	-1519.0977
17b	-963.4930	-0.0050	0.3642	-963.1338
9c-C	-923.4941	-0.0053	0.3160	-923.1834
9c-TS(C->D)	-923.4875	-0.0051	0.3180	-923.1747
9c-D	-923.5176	-0.0056	0.3206	-923.2026
9c-TS(C->E)	-923.4846	-0.0053	0.3190	-923.1709
9c-TS(D->E)	-923.4915	-0.0057	0.3209	-923.1762
9c-E	-923.5092	-0.0056	0.3206	-923.1943
9c-TS(E->F)	-923.4960	-0.0060	0.3209	-923.1811
9c-F	-923.5110	-0.0055	0.3206	-923.1959
9c-TS(F->18c)	-1480.2561	-0.0068	0.4453	-1479.8175
18c	-924.1744	-0.0053	0.3356	-923.8442
9c-TS(D->17c)	-1480.2525	-0.0058	0.4465	-1479.8118
17c	-924.1619	-0.0056	0.3354	-923.8321
9d-C	-884.1511	-0.0053	0.2872	-883.8692
9d-TS(C->D)	-884.1459	-0.0051	0.2892	-883.8618
9d-D	-884.1787	-0.0055	0.2920	-883.8922
9d-TS(C->E)	-884.1420	-0.0053	0.2898	-883.8575
9d-TS(D->E)	-884.1521	-0.0057	0.2921	-883.8657
9d-E	-884.1701	-0.0056	0.2923	-883.8834
9d-TS(E->F)	-884.1552	-0.0061	0.2921	-883.8692
9d-F	-884.1705	-0.0054	0.2914	-883.8846
9d-TS(F->18d)	-1440.9167	-0.0069	0.4160	-1440.5076
18d	-884.8345	-0.0054	0.3063	-884.5336
9d-TS(D->17d)	-1440.9252	-0.0058	0.4177	-1440.5133
17d	-884.8373	-0.0054	0.3072	-884.5355

4. References

(1) (a) Herdman, C. A.; Strecker, T. E.; Tanpure, R. P.; Chen, Z.; Winters, A.; Gerberich, J.; Liu, L.; Hamel, E.; Mason, R. P.; Chaplin, D. J.; Trawick, M. L.; Pinney, K. G. Synthesis and Biological Evaluation of Benzocyclooctene-Based and Indene-Based Anticancer Agents that Function as Inhibitors of Tubulin Polymerization. *Med. Chem. Comm.* **2016**, *7*, 2418-2427. (b) Tanpure, R. P.; George, C. S.; Strecker, T. E.; Devkota, L.; Tidmore, J. K.; Lin, C.-M.; Herdman, C. A.; MacDonough, M. T.; Sriram, M.; Chaplin, D. J.; Trawick, M. L.; Pinney, K. G. Synthesis of Structurally Diverse Benzosuberene Analogues and Their Biological Evaluation as Anti-Cancer Agents. *Bioorg. Med. Chem.* **2013**, *21*, 8019-8032.

(2) Michel, F.; Thomas, F.; Hamman, S.; Saint-Aman, E.; Bucher, C.; Pierre, J.-L. Galactose Oxidase Models: Solution Chemistry, and Phenoxyl Radical Generation Mediated by the Copper Status. *Chem. Eur. J.* **2004**, *10*, 4115-4125.

(3) Fillion, E.; Fishlock, D.; Wilsily, A.; Goll, J. M. Meldrum's Acids as Acylating Agents in the Catalytic Intramolecular Friedel-Crafts reaction. *J. Org. Chem.* **2005**, *70*, 1316-1327.

(4) Boger, D. L.; Coleman, R. S. Benzylic Hydroperoxide Rearrangement: Observations on a Viable and Convenient Alternative to the Baeyer-Villiger Rearrangement. *J. Org. Chem.* **1986**, *51*, 5436-5439.

(5) Ozaki, Y.; Oshio I.; Ohsuga Y.; Kaburagi S.; Sung Z.-Z.; Kim S.-W. A New Entry to the Synthesis of 1,2-Benzenediol Congeners. *Chem. Pharm. Bull.* **1991**, *39*, 1132-1136.

(6) Tanaka, M.; Okita, M.; Akamatsu, H.; Chiba, K.; Obaishi, H.; Nagakura, N.; Sakurai, H.; Yamatsu, I. Hydroxyindole Derivatives as Inhibitors of IL-1 Generation. II. Synthesis and Pharmacological Activities of (*E*)-3-(7-Hydroxy-6-methoxyindole-4-yl)-2-methylpropenoic Acid Derivatives. *Eur. J. Med. Chem.* **1996**, *31*, 187-198.

(7) (a) Becke, A. D. Density-Functional Exchange-Energy Approximation with Correct Asymptotic Behavior. *Phys. Rev. A* **1988**, *38*, 3098-3100. (b) Becke, A. D. Density-Functional Thermochemistry. III. The Role of Exact Exchange. *J. Chem. Phys.* **1993**, *98*, 5648-5652. (c) Lee, C.; Yang, W.; Parr, R. G. Development of the Colle-Salvetti Correlation-Energy Formula into a Functional of the Electron Density. *Phys. Rev. B* **1988**, *37*, 785-789.

(8) (a) Grimme, S.; Antony, J.; Ehrlich, S.; Krieg, S. A Consistent and Accurate *ab initio* Parametrization of Density Functional Dispersion Correction (DFT-D) for the 94 Elements H-Pu. *J. Chem. Phys.* **2010**, *132*, 154104. (b) Grimme, S.; Ehrlich, S.; Goerigk, L. Effect of the Damping Function in Dispersion Corrected Density

Functional Theory. *J. Comput. Chem.* **2011**, *32*, 1456-1465.

(9) (a) Hehre, W. J.; Ditchfield, R.; Pople, J. A. Self-Consistent Molecular Orbital Methods. XII. Further Extensions of Gaussian-Type Basis Sets for Use in Molecular Orbital Studies of Organic Molecules. *J. Chem. Phys.* **1972**, *56*, 2257-2261. (b) Francl, M. M.; Pietro, W. J.; Hehre, W. J.; Binkley, J. S.; Gordon, M. S.; DeFrees, D. J.; Pople, J. A. Self-Consistent Molecular Orbital Methods. XXIII. A Polarization-Type Basis Set for Second-Row Elements. *J. Chem. Phys.* **1982**, *77*, 3654-3665.

(10)(a) Keith, J. A.; Nielsen, R. J.; Oxgaard, J.; Goddard, W. A. Unraveling the Wacker Oxidation Mechanisms. *J. Am. Chem. Soc.* **2007**, *129*, 12342-12343. (b) Cramer, C. J. (2013). Essentials of Computational Chemistry: Theories and Models. John Wiley & Sons.

(11)(a) Barone, V.; Cossi. M. Quantum Calculation of Molecular Energies and Energy Gradients in Solution by a Conductor Solvent Model. *J. Phys. Chem. A* **1998**, *102*, 1995-2001. (b) Cossi, M.; Rega, N.; Scalmani, G.; Barone, V. Energies, Structures, and Electronic Properties of Molecules in Solution with the C-PCM Solvation Model. *J. Comp. Chem.* **2003**, *24*, 669-681.

(12)(a) Krishnan, R.; Binkley, J. S.; Seeger, R.; Pople, J. A. Self-Consistent Molecular Orbital Methods. XX. A Basis Set for Correlated Wave Functions. *J. Chem. Phys.* **1980**, *72*, 650-654. (b) Clark, T.; Chandrasekhar, J.; Spitznagel, G. W.; Schleyer, P. V. S. Efficient Diffuse Function-Augmented Basis Sets for Anion Calculations. III.† The 3-21+G Basis Set for First-Row Elements, Li–F. *J. Comput. Chem.* **1983**, *4*, 294-301.

(13)Wertz, D. H. Relationship between the Gas-Phase Entropies of Molecules and Their Entropies of Solvation in Water and 1-Octanol. *J. Am. Chem. Soc.* **1980**, *102*, 5316-5322.

(14)Cooper, J.; Ziegler, T. A Density Functional Study of S_N2 Substitution at Square-Planar Platinum(II) Complexes. *Inorganic Chemistry*, **2002**, *41*, 6614-6622.

5. Coordinates of Optimized Structures

15
tBuSH
C -0.044615 -0.259898 0.014390
C 0.476099 0.482808 1.249133
H 0.119622 1.515522 1.267129
H 0.130363 -0.020120 2.160599
H 1.570221 0.498452 1.270738
C -1.578192 -0.243061 -0.014507
H -1.964437 0.780232 -0.011666
H -1.964572 -0.757093 -0.899276
H -1.965719 -0.755291 0.872721
C 0.475889 -1.700645 -0.011380
H 0.119070 -2.232644 -0.896563
H 1.570003 -1.727296 -0.014484
H 0.130392 -2.238353 0.880094
S 0.488393 0.635745 -1.537142
H 1.816584 0.518612 -1.333648

14
tBuS-radical
C -0.060340 -0.247173 0.018979
C 0.468207 0.479551 1.263128
H 0.128288 1.518876 1.287652
H 0.098986 -0.017134 2.168692
H 1.560486 0.477037 1.285322
C -1.594911 -0.249627 -0.000337
H -1.991430 0.769690 -0.010375
H -1.976001 -0.772743 -0.880519
H -1.977265 -0.751091 0.897108
C 0.475179 -1.692434 -0.021332
H 0.116732 -2.228817 -0.904004
H 1.568200 -1.715793 -0.015106
H 0.117627 -2.223527 0.868930
S 0.589167 0.562038 -1.508232

37
19C
C -0.112529 -0.343930 0.134260
C 1.429681 -0.259556 0.109238
C 0.447281 2.121693 0.136448
C -0.716092 1.086797 0.118440
H -0.473569 -0.916027 -0.723138
H -0.417306 -0.880932 1.037846
H -1.281444 1.235285 -0.807706
C 1.866549 0.463581 -1.184083
C 1.295498 1.910040 -1.108419
H 0.062157 3.142577 0.146560
H 1.880857 -1.254229 0.136719
C 1.382104 1.829009 1.302857
C 1.901100 0.587235 1.280865
C -1.659842 1.304883 1.304661
H -2.466266 0.560201 1.322321
H -1.098164 1.198759 2.243167
C -2.285172 2.692758 1.351778
O -3.275058 3.018503 1.924082
O 1.536552 2.761584 -1.936076
O 3.272069 0.417561 -1.195650
O 1.303464 -0.067687 -2.366070
C 3.929500 1.137285 -2.243977
H 4.002326 2.202808 -2.010312
H 4.930217 0.705883 -2.319274
H 3.400150 1.021859 -3.192962
C 1.649052 -1.422225 -2.643374
H 2.725819 -1.586233 -2.539591
H 1.116077 -2.121847 -1.986580
H 1.348325 -1.609474 -3.675538
C 2.893325 -0.005595 2.231411
H 2.516891 -0.944475 2.656982
H 3.816358 -0.247955 1.691178
C 1.660000 2.930709 2.280189
H 2.123259 3.786489 1.773540

H 0.728442 3.301348 2.725903
H 3.145673 0.663247 3.056437
H 2.323934 2.618663 3.088679

37
19D
C -0.529687 -0.220941 0.235716
C 1.015274 -0.274652 0.163710
C 0.194468 2.200459 0.233493
C -0.986665 1.229328 0.556203
H -0.942016 -0.542782 -0.724894
H -0.892558 -0.912301 1.001592
H -1.875236 1.482462 -0.025608
C 1.437091 0.443341 -1.145833
C 0.850517 1.879267 -1.086786
H -0.130730 3.244170 0.226001
H 1.381275 -1.304013 0.146557
C 1.150974 1.920599 1.424665
C 1.616342 0.485826 1.306551
C -1.208098 1.469243 2.060442
H -1.852130 2.341645 2.229540
H -1.658450 0.626620 2.592401
C 0.173660 1.811008 2.616435
O 0.479280 1.934896 3.784114
O 0.955145 2.663394 -2.006965
O 2.849480 0.472985 -1.137300
O 0.919836 -0.131009 -2.320129
C 3.476434 1.148922 -2.233006
H 3.434251 2.234903 -2.110032
H 4.516338 0.816386 -2.229922
H 3.001476 0.886792 -3.181582
C 1.290078 -1.489255 -2.546389
H 2.353985 -1.649713 -2.346735
H 0.699196 -2.177511 -1.929003
H 1.081969 -1.692232 -3.598233
C 2.669363 -0.059505 2.201734
H 2.564016 0.330266 3.220680
H 2.629578 -1.152768 2.242211
C 2.263003 2.942477 1.637505
H 3.002488 2.880965 0.834584
H 1.852024 3.956467 1.659415
H 3.676279 0.213266 1.848645
H 2.761757 2.762505 2.592197

37
19E
C -0.576669 -0.293378 0.133034
C 0.960891 -0.356131 0.215280
C 0.150315 2.115235 0.300501
C -0.964154 1.089429 0.700386
H -0.893993 -0.417972 -0.904624
H -1.047430 -1.087011 0.721586
H -1.935150 1.426245 0.330106
C 1.450947 0.377300 -1.056883
C 0.781573 1.788482 -1.058799
H -0.200714 3.149351 0.296005
H 1.343047 -1.378590 0.236505
C 1.345099 1.881972 1.180870
C 1.458400 0.411668 1.508617
C -0.957795 0.926454 2.224768
H -1.070283 1.880637 2.753689
H -1.766641 0.271098 2.562928
C 0.382359 0.305773 2.626887
O 0.609849 -0.142190 3.731264
O 0.905902 2.576644 -1.972914
O 2.856992 0.477177 -0.989994
O 1.033324 -0.233325 -2.255249
C 3.496227 1.195091 -2.051327
H 3.397496 2.275938 -1.916103
H 4.549504 0.910823 -2.008801
H 3.071727 0.923655 -3.020507
C 1.484890 -1.571608 -2.448826
H 2.537973 -1.679718 -2.171579
H 0.884358 -2.287582 -1.873788
H 1.364667 -1.784959 -3.512381
C 2.810387 -0.072147 2.014680

H 2.736770 -1.117940 2.323798
H 3.561031 0.018010 1.230140
C 2.386737 2.901635 1.468093
H 2.055418 3.898671 1.164827
H 2.635850 2.935631 2.536875
H 3.324318 2.679993 0.937832
H 3.125795 0.497998 2.891345

37
19F
C -0.866402 0.042808 0.226932
C 0.651421 -0.275709 0.181097
C -0.104662 2.342542 0.775391
C -1.114266 1.292187 1.111951
H -1.238616 0.227147 -0.780673
H -1.420151 -0.807367 0.640217
H -2.136996 1.657000 0.987988
C 1.321564 0.332611 -1.079777
C 1.692937 1.772660 -0.638299
H -0.362706 3.325813 0.398726
H 0.835927 -1.351171 0.208181
C 1.341129 1.930049 0.852748
C 1.393202 0.448388 1.371044
C -0.848681 0.860343 2.566714
H -0.944720 1.697131 3.267495
H -1.543388 0.079431 2.897444
C 0.571198 0.320249 2.658392
O 1.017170 -0.165691 3.679039
O 2.184082 2.621771 -1.348267
O 2.487233 -0.427930 -1.323490
O 0.519854 0.429637 -2.228200
C 3.368997 0.081328 -2.331290
H 3.989196 0.895388 -1.944438
H 4.003022 -0.758043 -2.623275
H 2.809989 0.449349 -3.195108
C 0.163985 -0.823222 -2.812637
H 1.049465 -1.403559 -3.085909
H -0.456199 -1.426330 -2.137739
H -0.411521 -0.585201 -3.708311
C 2.812975 -0.058099 1.618043
H 2.799947 -1.137024 1.788646
H 3.466256 0.139785 0.769147
C 2.231397 2.948360 1.559794
H 2.140956 3.918879 1.065962
H 1.939851 3.058140 2.607958
H 3.227191 0.406001 2.514697
H 3.281309 2.648676 1.517357

38
21
C -0.862947 0.058960 0.210432
C 0.653072 -0.278111 0.177606
C -0.172463 2.410778 0.789037
C -1.126019 1.258585 1.135347
H -1.214612 0.285275 -0.797213
H -1.429502 -0.806763 0.568944
H -2.164200 1.591346 1.033855
C 1.343527 0.308467 -1.082224
C 1.737298 1.744163 -0.633002
H -0.412089 2.830888 -0.193396
H 0.825437 -1.355156 0.218589
C 1.329651 1.933389 0.825344
C 1.393280 0.455248 1.359885
C -0.839177 0.820872 2.571130
H -0.963001 1.642367 3.287401
H -1.503893 0.013313 2.898769
C 0.595358 0.328061 2.658585
O 1.071101 -0.116077 3.685448
O 2.299217 2.558217 -1.329966
O 2.484052 -0.485245 -1.326965
O 0.547138 0.438486 -2.232674
C 3.387646 0.001914 -2.326080
H 4.039736 0.784111 -1.926259
H 3.987721 -0.858352 -2.629128
H 2.846033 0.404806 -3.185334
C 0.153141 -0.799491 -2.824860

H 1.021094 -1.402621 -3.104715
H -0.481920 -1.388894 -2.151968
H -0.417657 -0.538668 -3.717204
C 2.822131 -0.037668 1.593766
H 2.823111 -1.118944 1.749903
H 3.473066 0.177194 0.746857
C 2.215064 2.944618 1.542126
H 2.129037 3.919703 1.055434
H 1.915093 3.048022 2.589494
H -0.295982 3.223293 1.512921
H 3.233895 0.420114 2.494420
H 3.266649 2.651551 1.511489

38
20
C -0.344443 -0.288742 0.377373
C 1.187767 -0.277598 0.242988
C 0.290837 2.163069 0.356743
C -0.878738 1.157733 0.590703
H -0.782950 -0.717184 -0.525924
H -0.642408 -0.930233 1.212189
H -1.716318 1.358940 -0.079922
C 1.549529 0.457385 -1.063789
C 1.030108 1.912239 -0.930723
H -0.066039 3.197251 0.358089
H 1.583918 -1.296377 0.192626
C 1.172867 1.879851 1.610242
C 1.851773 0.463781 1.425469
C -1.237661 1.436443 2.058023
H -1.898759 2.306908 2.152905
H -1.720804 0.603849 2.577172
C 0.098281 1.796310 2.706649
O 0.285380 2.009680 3.884124
O 1.233102 2.760934 -1.774735
O 2.951809 0.401721 -1.186540
O 0.898624 -0.051066 -2.209374
C 3.531450 1.081273 -2.305888
H 3.627828 2.153326 -2.115326
H 4.520312 0.637888 -2.441878
H 2.929599 0.938632 -3.206329
C 1.181343 -1.415730 -2.510994
H 2.252933 -1.624603 -2.438227
H 0.636324 -2.100373 -1.848837
H 0.847086 -1.576944 -3.537274
C 1.897912 -0.384695 2.699773
H 0.907018 -0.732653 3.007938
H 2.524799 -1.266463 2.535653
C 2.209980 2.950902 1.925465
H 2.943666 3.027076 1.117741
H 1.739516 3.929698 2.058084
H 2.884891 0.661920 1.129058
H 2.733191 2.705846 2.853508
H 2.313358 0.181574 3.537003

37
19-TS(C->D)
C -0.378115 -0.187892 0.403873
C 1.147850 -0.234621 0.189086
C 0.362105 2.228199 0.209221
C -0.849228 1.290010 0.514033
H -0.881432 -0.681975 -0.430416
H -0.630037 -0.738796 1.314531
H -1.649878 1.484445 -0.204331
C 1.453238 0.390530 -1.193677
C 0.962764 1.865927 -1.137621
H 0.062174 3.278147 0.199468
H 1.521963 -1.260750 0.214141
C 1.421109 1.934591 1.271689
C 1.854252 0.624564 1.218258
C -1.335607 1.621466 1.930435
H -1.849713 2.590988 1.941581
H -2.023128 0.864653 2.326842
C -0.117710 1.770030 2.828291
O 0.069727 1.368197 3.936793
O 1.086503 2.637355 -2.064949
O 2.846782 0.287468 -1.367475

O 0.738400 -0.182075 -2.266841
C 3.392741 0.904380 -2.537873
H 3.509704 1.983210 -2.402553
H 4.371124 0.443011 -2.688585
H 2.757205 0.727835 -3.409149
C 0.986933 -1.568596 -2.485286
H 2.058870 -1.787204 -2.467051
H 0.480513 -2.194648 -1.739454
H 0.583416 -1.800309 -3.472405
C 2.939155 0.023958 2.047093
H 2.571844 -0.853131 2.596797
H 3.756225 -0.322379 1.400954
C 2.218542 3.073814 1.855197
H 3.032046 3.364177 1.178482
H 1.583783 3.948239 2.018740
H 3.350428 0.726274 2.773855
H 2.659402 2.800557 2.816469

37
19-TS(C->E)
C -0.429488 -0.334501 0.225856
C 1.110461 -0.371423 0.140825
C 0.306298 2.084737 0.247014
C -0.840315 1.090606 0.654865
H -0.844756 -0.588482 -0.752103
H -0.791695 -1.068834 0.949805
H -1.776915 1.392233 0.178874
C 1.474628 0.325528 -1.195539
C 0.846429 1.752604 -1.141190
H -0.025355 3.123976 0.273277
H 1.497772 -1.392022 0.146405
C 1.475261 1.806076 1.166585
C 1.749648 0.451623 1.262670
C -0.982107 1.115544 2.179070
H -1.175089 2.121231 2.575867
H -1.820076 0.474578 2.480476
C 0.270814 0.535926 2.827483
O 0.690300 0.752224 3.933238
O 0.865353 2.519215 -2.079236
O 2.882153 0.361504 -1.264777
O 0.910171 -0.276864 -2.336557
C 3.441862 1.082212 -2.366805
H 3.414445 2.162050 -2.192239
H 4.478611 0.748869 -2.445399
H 2.905897 0.863223 -3.293622
C 1.295427 -1.630819 -2.560254
H 2.372288 -1.765077 -2.420175
H 0.756578 -2.319611 -1.897136
H 1.030671 -1.857991 -3.594299
C 2.941205 -0.101283 1.987151
H 2.795196 -1.158839 2.222731
H 3.827384 -0.014347 1.348430
C 2.149713 2.877102 1.960921
H 1.841017 3.870887 1.627767
H 1.899143 2.780854 3.028483
H 3.119839 0.430357 2.923820
H 3.240309 2.813686 1.882544

37
19-TS(D->E)
C -0.675972 -0.107354 0.113936
C 0.852351 -0.231993 0.220611
C 0.170614 2.277976 0.199355
C -1.036202 1.328343 0.548110
H -0.981210 -0.310623 -0.915640
H -1.178950 -0.830931 0.760934
H -1.954829 1.679845 0.074687
C 1.404925 0.437720 -1.066516
C 0.809923 1.877418 -1.118595
H -0.125375 3.328402 0.168038
H 1.181834 -1.271671 0.274328
C 1.216870 1.998769 1.278833
C 1.416553 0.554117 1.419622
C -1.093026 1.347171 2.073431
H -1.493870 2.288809 2.466761
H -1.700441 0.530460 2.476080

C 0.360783 1.245714 2.507394
O 0.753650 1.358943 3.698838
O 0.917512 2.598840 -2.085996
O 2.811960 0.481975 -0.955858
O 0.992128 -0.184811 -2.256942
C 3.509130 1.139313 -2.020224
H 3.437618 2.227585 -1.933691
H 4.551645 0.829877 -1.926657
H 3.113298 0.839378 -2.993338
C 1.386491 -1.547744 -2.400421
H 2.426594 -1.694728 -2.093666
H 0.740101 -2.218279 -1.820554
H 1.281868 -1.786592 -3.460052
C 2.647706 -0.017934 2.076260
H 2.900612 0.527796 2.984181
H 2.474422 -1.063635 2.349923
C 2.230963 3.040110 1.651243
H 3.043245 3.060618 0.915218
H 1.774772 4.033662 1.687197
H 3.483137 0.020210 1.372896
H 2.658403 2.823505 2.631739

37
19-TS(E->F)
C -0.479488 -0.360034 0.140232
C 1.056553 -0.424676 0.254978
C 0.078784 2.079730 0.424063
C -0.930750 0.991090 0.732295
H -0.778239 -0.442763 -0.904173
H -0.952501 -1.176389 0.695653
H -1.929551 1.268611 0.388483
C 1.658587 0.303711 -0.967325
C 1.508404 1.783121 -0.602811
H -0.249387 3.086892 0.195318
H 1.429857 -1.447559 0.324234
C 1.430962 1.878783 0.959421
C 1.545319 0.427788 1.497325
C -0.877662 0.818311 2.264456
H -1.057574 1.762233 2.792922
H -1.620692 0.098639 2.622874
C 0.515022 0.311189 2.634717
O 0.785526 -0.124726 3.734874
O 1.819133 2.751642 -1.337023
O 3.025003 -0.058132 -1.031195
O 1.016347 0.072019 -2.196656
C 3.817555 0.661557 -1.981958
H 4.042847 1.672625 -1.630561
H 4.741921 0.090730 -2.090569
H 3.306307 0.735124 -2.945317
C 1.114052 -1.266078 -2.678034
H 2.153408 -1.604467 -2.712305
H 0.536798 -1.964392 -2.057415
H 0.695264 -1.259102 -3.685586
C 2.933996 0.044229 1.994501
H 2.935822 -1.001612 2.310656
H 3.674346 0.172134 1.206294
C 2.189837 3.007931 1.613271
H 2.060452 3.920338 1.027061
H 1.824956 3.186103 2.630309
H 3.209822 0.642226 2.865653
H 3.258234 2.784645 1.664090

52
19-TS(F->21)
C -0.787444 -0.101959 0.146768
C 0.740843 -0.373886 0.159383
C -0.181044 2.270328 0.602363
C -1.115980 1.151231 0.998753
H -1.132600 0.045974 -0.876828
H -1.328935 -0.958785 0.561734
H -2.159290 1.442364 0.848363
C 1.420088 0.173197 -1.122892
C 1.711718 1.655335 -0.764102
H -0.450447 2.864855 -0.271043
H 0.958474 -1.438136 0.264594
C 1.306987 1.900456 0.701231

C 1.426496 0.456895 1.312089
C -0.845483 0.788811 2.463675
H -0.998697 1.643692 3.130892
H -1.498074 -0.019538 2.810951
C 0.598610 0.340004 2.596249
O 1.060416 -0.078370 3.639050
O 2.194281 2.473590 -1.511693
O 2.614912 -0.555660 -1.295059
O 0.641701 0.175613 -2.292320
C 3.504564 -0.072616 -2.309372
H 4.086360 0.783159 -1.954095
H 4.174495 -0.903704 -2.537199
H 2.955613 0.225651 -3.205828
C 0.333729 -1.122018 -2.803003
H 1.241356 -1.694857 -3.011116
H -0.291956 -1.694715 -2.107121
H -0.219588 -0.958115 -3.728669
C 2.869711 0.045023 1.601978
H 2.914491 -1.023674 1.822937
H 3.528519 0.240248 0.756750
C 2.169585 2.983805 1.347397
H 2.008236 3.938712 0.843578
H 1.918260 3.100026 2.404678
H -0.438497 3.383386 1.613264
S -0.668940 4.516825 2.566130
C -2.473583 4.827620 2.207367
C -2.781606 6.186361 2.854339
H -3.840368 6.429347 2.706708
H -2.586138 6.167370 3.930518
H -2.179963 6.982327 2.407940
C -2.711713 4.900144 0.694933
H -2.521950 3.937014 0.212558
H -3.756273 5.168827 0.496006
H -2.067367 5.650168 0.229890
C -3.345258 3.737264 2.841365
H -3.175054 3.677162 3.919121
H -4.406231 3.958074 2.670339
H -3.135185 2.755585 2.409327
H 3.229110 2.733262 1.266514
H 3.239441 0.573588 2.481978

52
19-TS(D->20)
C -0.314657 -0.268613 0.385246
C 1.135439 -0.304005 -0.158497
C 0.395852 2.160468 0.217139
C -0.727686 1.197335 0.707836
H -0.987652 -0.691385 -0.361280
H -0.389797 -0.893045 1.280222
H -1.687945 1.436502 0.247795
C 1.134901 0.442595 -1.514341
C 0.814026 1.927821 -1.211519
H 0.101176 3.207899 0.326874
H 1.471483 -1.331256 -0.321416
C 1.538368 1.807072 1.214789
C 2.042785 0.379789 0.868768
C -0.724280 1.468772 2.219266
H -1.274277 2.386116 2.464691
H -1.140233 0.664745 2.832634
C 0.746996 1.712346 2.545855
O 1.230917 1.828468 3.648427
O 0.912270 2.810193 -2.037876
O 2.385661 0.228721 -2.090928
O 0.082791 0.043568 -2.377755
C 2.698692 0.941455 -3.295012
H 3.049092 1.950358 -3.069782
H 3.499597 0.372435 -3.769792
H 1.827742 0.996709 -3.951701
C 0.142032 -1.313800 -2.813401
H 1.147272 -1.576858 -3.153627
H -0.160383 -2.010424 -2.020636
H -0.561096 -1.397541 -3.643600
C 2.456384 -0.518264 2.015261
H 1.580559 -0.980243 2.491259
H 3.097547 -1.323715 1.647626
C 2.641412 2.855148 1.320512

H 3.186563 2.938327 0.378830
H 2.215825 3.830815 1.572613
S 4.735602 0.460783 -0.502531
H 3.388230 0.563953 0.241120
C 6.080860 0.940247 0.701560
C 5.790491 0.455681 2.124001
H 6.655759 0.660416 2.766154
H 4.932214 0.975034 2.556189
H 5.593663 -0.618969 2.147060
C 6.301983 2.458328 0.687690
H 7.154664 2.720393 1.326962
H 6.510291 2.812413 -0.325198
H 5.426487 2.993541 1.060023
C 7.335929 0.232611 0.161502
H 7.547214 0.528116 -0.870709
H 8.202995 0.507413 0.774359
H 7.219936 -0.853801 0.190426
H 2.988475 0.029862 2.792256
H 3.340913 2.582673 2.113605

47
9a-C
C -1.872603 -1.435705 -0.100147
C -0.347584 -1.398994 -0.333111
C -1.239614 1.006044 -0.222280
C -2.418686 0.013129 -0.005673
H -2.359632 -1.971516 -0.917879
H -2.071504 -1.979618 0.826716
H -3.155402 0.182707 -0.797753
C -0.069871 -0.714960 -1.690399
C -0.606675 0.739655 -1.577496
H -1.580729 2.042565 -0.187171
H 0.072057 -2.407561 -0.344838
C -0.143865 0.718769 0.798341
C 0.321654 -0.547557 0.736478
C -3.117631 0.294535 1.333608
H -3.455631 1.336745 1.402685
H -3.997726 -0.355943 1.418570
C -2.253071 -0.039010 2.544657
O -2.074131 0.632772 3.512566
O -0.485041 1.562676 -2.459449
O 1.325985 -0.775600 -1.878558
O -0.776231 -1.267586 -2.779737
C 1.849653 -0.084602 -3.018303
H 1.963020 0.983855 -2.816290
H 2.828107 -0.529434 -3.212308
H 1.199938 -0.212509 -3.887243
C -0.489435 -2.637046 -3.052619
H 0.588880 -2.822268 -3.060637
H -0.962447 -3.305336 -2.321750
H -0.903468 -2.845320 -4.040582
C 1.411327 -1.231456 1.519242
H 0.947276 -2.092335 2.024030
H 2.097670 -1.671309 0.780584
C 0.365675 1.858150 1.642771
H -0.396127 2.644253 1.673510
H 0.492324 1.538983 2.677096
C 2.223500 -0.437335 2.549403
H 1.546984 0.057202 3.249408
H 2.781543 -1.162126 3.154399
C 1.682068 2.461920 1.104309
H 1.462062 3.021499 0.186318
H 2.049887 3.194455 1.834334
C 2.781437 1.440832 0.789801
H 3.654966 1.991237 0.418558
H 2.454820 0.802300 -0.037723
C 3.245830 0.554835 1.956569
H 4.105053 -0.026927 1.597892
H 3.628730 1.188020 2.767957

47
9a-D
C -1.959773 -1.442987 -0.516998
C -0.418678 -1.384646 -0.373022
C -1.388937 1.021814 -0.430279
C -2.552717 -0.016650 -0.343297

H -2.215527 -1.840662 -1.502085
H -2.374533 -2.122761 0.233081
H -3.312908 0.177433 -1.102517
C 0.125639 -0.654157 -1.624425
C -0.494704 0.768355 -1.618998
H -1.770909 2.044289 -0.489454
H 0.011558 -2.387787 -0.330684
C -0.659608 0.781856 0.927028
C -0.037872 -0.599687 0.856048
C -3.087644 0.218845 1.076816
H -3.754930 1.090082 1.108483
H -3.632487 -0.626108 1.506565
C -1.849462 0.565237 1.897831
O -1.774381 0.616171 3.107261
O -0.262948 1.584703 -2.486605
O 1.530230 -0.626780 -1.497172
O -0.279889 -1.224963 -2.847777
C 2.251550 0.047784 -2.535434
H 2.230458 1.132038 -2.395047
H 3.279201 -0.313661 -2.464259
H 1.836004 -0.186806 -3.517860
C 0.122653 -2.578293 -3.048569
H 1.169438 -2.727392 -2.767189
H -0.505572 -3.276214 -2.480804
H -0.001170 -2.779090 -4.114024
C 0.590218 -1.282077 2.028502
H -0.182287 -1.517887 2.780012
H 0.978324 -2.245971 1.678315
C 0.229194 1.949170 1.389484
H -0.338357 2.864919 1.185585
H 0.320410 1.897826 2.477509
C 1.728613 -0.537248 2.753793
H 1.311495 0.273247 3.358129
H 2.159644 -1.244965 3.471038
C 1.619398 2.047772 0.729713
H 1.612585 1.536529 -0.235823
H 1.821872 3.102510 0.512316
C 2.793431 1.512933 1.562221
H 3.720802 1.795608 1.047583
H 2.811994 2.037626 2.528126
C 2.834221 -0.000591 1.819006
H 3.812539 -0.231781 2.256510
H 2.790730 -0.528477 0.858885

47
9a-E
C -2.239495 -1.179181 -0.383896
C -0.706533 -1.294508 -0.262729
C -1.393005 1.197495 -0.384904
C -2.575487 0.257776 0.058879
H -2.543891 -1.378404 -1.413197
H -2.750716 -1.901968 0.259712
H -3.518571 0.613356 -0.362668
C -0.165419 -0.696501 -1.583573
C -0.749942 0.745721 -1.695772
H -1.700714 2.240987 -0.473347
H -0.374276 -2.328518 -0.153844
C -0.255475 0.997781 0.583648
C -0.192050 -0.462756 0.982099
C -2.608365 0.214400 1.590467
H -2.683094 1.210803 2.043643
H -3.456197 -0.371729 1.958565
C -1.311165 -0.441858 2.073490
O -1.139826 -0.814714 3.216110
O -0.596256 1.447179 -2.672394
O 1.242610 -0.696628 -1.532948
O -0.630770 -1.368102 -2.733630
C 1.917301 -0.094372 -2.644759
H 1.910191 0.996432 -2.567427
H 2.944715 -0.460359 -2.601115
H 1.450916 -0.385273 -3.588239
C -0.277987 -2.746611 -2.819157
H 0.766357 -2.907964 -2.535043
H -0.924839 -3.368970 -2.187981
H -0.420390 -3.035119 -3.862001
C 1.098895 -1.083484 1.546888

H 0.783849 -2.026617 2.004943
H 1.744745 -1.340414 0.704351
C 0.366182 2.144570 1.315808
H -0.277368 3.024844 1.198949
H 0.390030 1.926731 2.389244
C 1.897466 -0.280302 2.591453
H 1.218603 0.322349 3.196937
H 2.336979 -0.992874 3.298284
C 1.791373 2.530588 0.852335
H 1.712992 3.015274 -0.128663
H 2.180669 3.287477 1.545786
C 2.774513 1.363843 0.736995
H 3.728794 1.763777 0.371674
H 2.414017 0.679054 -0.035004
C 3.052863 0.567407 2.019448
H 3.885877 -0.112654 1.798007
H 3.419276 1.244919 2.802617

47
9a-F
C -2.380197 -1.030360 -0.567752
C -0.845099 -1.250570 -0.464394
C -1.811155 1.319192 0.009723
C -2.788945 0.219305 0.257234
H -2.665164 -0.892551 -1.610565
H -2.915386 -1.906044 -0.184507
H -3.811296 0.524791 0.021116
C -0.112065 -0.618105 -1.671464
C 0.080687 0.855720 -1.244664
H -2.087123 2.279898 -0.410344
H -0.597137 -2.311057 -0.392569
C -0.355249 1.003215 0.229163
C -0.268165 -0.461760 0.787734
C -2.648184 -0.171990 1.740961
H -2.768739 0.697467 2.398386
H -3.379327 -0.925591 2.051923
C -1.254062 -0.742906 1.932780
O -0.970848 -1.449878 2.880644
O 0.496300 1.743801 -1.957248
O 1.142087 -1.264627 -1.774524
O -0.798476 -0.632632 -2.895431
C 2.059317 -0.699119 -2.718508
H 2.547060 0.194287 -2.316910
H 2.806475 -1.472366 -2.906801
H 1.552111 -0.430712 -3.648458
C -0.992915 -1.932578 -3.451871
H -0.043286 -2.460040 -3.577137
H -1.656680 -2.546100 -2.829671
H -1.459943 -1.777698 -4.425521
C 1.139206 -0.925006 1.193559
H 1.099350 -2.015824 1.257877
H 1.831534 -0.706906 0.380609
C 0.384586 2.152506 0.937580
H -0.041277 3.072358 0.526126
H 0.106276 2.143645 1.996888
C 1.694429 -0.369438 2.531937
H 0.936112 0.213114 3.062638
H 1.899176 -1.218884 3.188692
C 1.926198 2.226428 0.779664
H 2.263371 1.565710 -0.024220
H 2.169735 3.235817 0.434388
C 2.743348 1.948860 2.050282
H 3.723961 2.429557 1.945164
H 2.255973 2.445924 2.900886
C 2.970772 0.473047 2.395325
H 3.529769 0.431162 3.338374
H 3.624710 0.018691 1.637923

48
18a
C -0.751832 0.074921 0.402030
C 0.779420 -0.172218 0.311585
C -0.156587 2.483049 0.744013
C -1.037649 1.335204 1.234937
H -1.169735 0.191940 -0.598583
H -1.239371 -0.789567 0.864688

H -2.093375 1.614601 1.155979
C 1.388269 0.370146 -1.005817
C 1.749311 1.837106 -0.650238
H -0.432487 2.772429 -0.275678
H 1.012898 -1.233152 0.411952
C 1.378329 2.103390 0.807585
C 1.538406 0.661610 1.437792
C -0.664754 1.041236 2.690212
H -0.647601 1.961463 3.289376
H -1.354435 0.347141 3.179548
C 0.719155 0.419727 2.704887
O 1.085675 -0.312795 3.604751
O 2.243452 2.629712 -1.420354
O 2.539293 -0.400854 -1.269215
O 0.530577 0.418039 -2.118977
C 3.386188 0.071272 -2.323165
H 4.040368 0.877238 -1.977392
H 3.989410 -0.786499 -2.627235
H 2.798613 0.437731 -3.168352
C 0.168981 -0.858715 -2.645984
H 1.048118 -1.418820 -2.975784
H -0.376828 -1.464135 -1.911917
H -0.481443 -0.658711 -3.498710
C 3.000514 0.168901 1.584807
H 2.988420 -0.917282 1.455355
H 3.571074 0.540083 0.733325
C 2.141218 3.322753 1.349520
H 1.838968 4.146574 0.694499
H 1.750544 3.569479 2.343733
C 3.768505 0.450423 2.895648
H 3.456115 -0.291368 3.631335
H 4.831529 0.265316 2.689409
C 3.680239 3.267450 1.386083
H 4.048790 2.531182 0.668647
H 4.071448 4.227465 1.033012
H -0.316997 3.366360 1.370339
C 4.258801 2.998900 2.782648
H 4.136835 3.907066 3.388402
H 5.340793 2.833972 2.696435
C 3.628093 1.830352 3.543170
H 4.088728 1.782248 4.537496
H 2.568499 2.038883 3.731757

48

17a

C -0.475679 -0.086051 0.152968
C 1.047771 -0.264508 0.070613
C 0.465848 2.260948 0.118930
C -0.823357 1.416624 0.344189
H -0.934074 -0.460416 -0.764639
H -0.880583 -0.678995 0.978387
H -1.617351 1.714717 -0.343427
C 1.539624 0.389527 -1.235931
C 1.195714 1.896970 -1.146093
H 0.239554 3.331035 0.092021
H 1.317459 -1.324566 0.050568
C 1.295045 1.910181 1.402856
C 1.771483 0.408128 1.262295
C -1.167592 1.766599 1.798488
H -1.766646 2.682279 1.867853
H -1.711060 0.984965 2.337894
C 0.182529 2.049784 2.457649
O 0.324185 2.405096 3.608746
O 1.517657 2.697594 -2.000206
O 2.929430 0.164811 -1.305645
O 0.873855 -0.064212 -2.395443
C 3.625050 0.750281 -2.411636
H 3.837893 1.807803 -2.234361
H 4.560438 0.193609 -2.501317
H 3.044759 0.659460 -3.332729
C 0.999449 -1.460105 -2.658006
H 2.034150 -1.794964 -2.537502
H 0.350253 -2.056955 -2.004949
H 0.687346 -1.605167 -3.693640
C 1.676106 -0.460127 2.526674
H 0.622842 -0.606868 2.801736

H 2.053492 -1.454752 2.253386
C 2.451297 2.905830 1.598680
H 3.040565 2.898330 0.671902
H 2.007402 3.906861 1.663590
C 2.432153 0.026953 3.769094
H 1.942916 0.915669 4.171077
H 2.318264 -0.746811 4.538712
C 3.421083 2.744072 2.783489
H 4.044549 3.645513 2.776478
H 2.861674 2.776129 3.721654
H 2.820419 0.449764 0.975933
C 4.352364 1.511834 2.735984
H 5.350993 1.815239 3.070724
H 4.487487 1.205491 1.692765
C 3.933103 0.303840 3.607118
H 4.438444 -0.596596 3.232789
H 4.327961 0.467969 4.617505

47
9a-TS(C->D)
C -0.497978 -0.161467 0.224174
C 1.038841 -0.209706 0.340279
C 0.274384 2.247714 0.246500
C -0.978028 1.314988 0.277605
H -0.809894 -0.625835 -0.714073
H -0.938916 -0.738340 1.042038
H -1.619464 1.536530 -0.579532
C 1.628539 0.437919 -0.935589
C 1.139183 1.912135 -0.953314
H -0.012694 3.299906 0.196981
H 1.395224 -1.238592 0.419506
C 1.088022 1.930383 1.505544
C 1.515310 0.617014 1.523828
C -1.738971 1.607933 1.577839
H -2.223510 2.590747 1.523452
H -2.507539 0.854427 1.787990
C -0.728324 1.677699 2.712092
O -0.745550 1.146444 3.783356
O 1.454552 2.702552 -1.817905
O 3.029275 0.327879 -0.824778
O 1.151907 -0.110631 -2.144658
C 3.804818 0.971163 -1.841921
H 3.904536 2.042807 -1.648220
H 4.788486 0.498064 -1.809051
H 3.353069 0.832245 -2.826866
C 1.436360 -1.495040 -2.332758
H 2.478915 -1.721878 -2.090673
H 0.779994 -2.130706 -1.724806
H 1.252009 -1.704733 -3.387697
C 2.286213 -0.071157 2.610656
H 1.570470 -0.377860 3.392131
H 2.687331 -0.999942 2.189234
C 1.698033 3.101063 2.247647
H 0.985687 3.929938 2.187038
H 1.796363 2.873760 3.311506
C 3.445773 0.691243 3.277252
H 3.056608 1.413207 4.000727
H 4.002126 -0.040445 3.873915
C 3.056179 3.556567 1.659137
H 3.057553 3.370641 0.578446
H 3.136334 4.644144 1.774363
C 4.305554 2.930381 2.293080
H 5.180399 3.338656 1.771216
H 4.385479 3.279697 3.332316
C 4.403695 1.396862 2.288385
H 5.436099 1.133564 2.545240
H 4.230219 1.018083 1.275075

47
9a-TS(C->E)
C -0.352549 -0.299780 0.212161
C 1.188008 -0.356710 0.220674
C 0.421217 2.100733 0.175223
C -0.763412 1.144886 0.561449
H -0.714485 -0.586854 -0.777452
H -0.766565 -1.001100 0.941431

H -1.670689 1.442104 0.029491
C 1.638237 0.269238 -1.123564
C 1.050710 1.712404 -1.156317
H 0.106150 3.144639 0.137032
H 1.555476 -1.382071 0.293952
C 1.524950 1.860021 1.187621
C 1.783958 0.500387 1.353302
C -0.973825 1.226586 2.075249
H -1.161971 2.248058 2.431437
H -1.837738 0.613833 2.361714
C 0.236129 0.638930 2.794106
O 0.564681 0.807083 3.938576
O 1.166664 2.451282 -2.110291
O 3.048553 0.255611 -1.137319
O 1.107071 -0.364555 -2.264433
C 3.677745 0.911047 -2.244514
H 3.700819 1.995225 -2.101577
H 4.697649 0.523133 -2.279269
H 3.156652 0.688400 -3.178266
C 1.453087 -1.739875 -2.409999
H 2.516898 -1.904103 -2.213749
H 0.860047 -2.378697 -1.743213
H 1.227598 -2.004624 -3.444373
C 2.919981 -0.166881 2.105654
H 2.487339 -1.041244 2.605822
H 3.596983 -0.566431 1.338152
C 2.101017 3.009479 1.960926
H 1.413821 3.860107 1.888056
H 2.144642 2.750407 3.022065
C 3.735676 0.633674 3.131779
H 3.062003 1.204660 3.770513
H 4.212855 -0.091348 3.801661
C 3.494625 3.466013 1.472916
H 3.374773 3.983572 0.512884
H 3.879462 4.210384 2.182166
C 4.514900 2.339306 1.288098
H 5.445251 2.786280 0.915649
H 4.163880 1.666934 0.499346
C 4.854492 1.513192 2.537196
H 5.689955 0.853581 2.267962
H 5.237118 2.176940 3.324120

47
9a-TS(D->E)
C -0.454758 -0.223513 0.049377
C 1.065835 -0.306581 0.251293
C 0.331267 2.176538 0.334991
C -0.884030 1.185871 0.499590
H -0.691331 -0.405106 -0.999959
H -0.971713 -0.979405 0.646256
H -1.755705 1.548560 -0.048137
C 1.668233 0.427743 -0.975261
C 1.139709 1.894295 -0.919636
H 0.010693 3.219880 0.338224
H 1.420287 -1.339107 0.275746
C 1.265683 1.863658 1.509988
C 1.531121 0.421388 1.540554
C -1.093007 1.107890 2.004537
H -1.519797 2.027263 2.423589
H -1.742031 0.275701 2.293362
C 0.311030 0.965388 2.572907
O 0.560462 0.945446 3.802597
O 1.410619 2.719087 -1.765774
O 3.069119 0.345167 -0.884127
O 1.214236 -0.066905 -2.216485
C 3.816544 1.032298 -1.897920
H 3.878645 2.102796 -1.686265
H 4.815545 0.593228 -1.876506
H 3.360414 0.890436 -2.879430
C 1.513050 -1.438787 -2.468385
H 2.545189 -1.677004 -2.194881
H 0.831944 -2.106127 -1.925361
H 1.375005 -1.589556 -3.540224
C 2.685368 -0.280597 2.251794
H 2.253180 -1.169302 2.727247
H 3.343919 -0.652000 1.457545

C 2.039992 2.979070 2.173509
H 1.408039 3.874801 2.141518
H 2.158654 2.734071 3.229412
C 3.529332 0.469342 3.298163
H 2.863345 1.025589 3.954543
H 3.990952 -0.292752 3.937343
C 3.407985 3.317443 1.560283
H 3.262300 3.737647 0.556854
H 3.845343 4.120425 2.168242
C 4.393167 2.148178 1.461915
H 5.347534 2.548706 1.096712
H 4.045423 1.453843 0.697207
C 4.671445 1.350951 2.744149
H 5.010736 2.026631 3.540872
H 5.525102 0.696334 2.525219

47
9a-TS(E->F)
C -0.553342 -0.340668 0.099902
C 0.980201 -0.481517 0.203170
C 0.118564 2.054744 0.458143
C -0.939404 1.011555 0.735716
H -0.860658 -0.375886 -0.944510
H -1.060778 -1.151743 0.632231
H -1.925061 1.347379 0.406301
C 1.610228 0.244597 -1.004794
C 1.543313 1.717788 -0.600386
H -0.160024 3.081185 0.250399
H 1.301118 -1.523273 0.247224
C 1.472892 1.778781 0.961916
C 1.518183 0.312752 1.473617
C -0.886197 0.790589 2.262166
H -0.970227 1.734730 2.814414
H -1.682704 0.129218 2.616530
C 0.458839 0.146310 2.580489
O 0.655426 -0.487581 3.597792
O 1.886691 2.687350 -1.313733
O 2.959778 -0.178619 -1.090412
O 0.949202 0.073341 -2.233423
C 3.765079 0.498157 -2.062670
H 4.016173 1.512042 -1.739624
H 4.673988 -0.098671 -2.160172
H 3.250921 0.559237 -3.025308
C 0.969096 -1.258800 -2.740883
H 1.986757 -1.657415 -2.780078
H 0.349926 -1.933645 -2.135089
H 0.554397 -1.207476 -3.748847
C 2.874039 -0.223130 1.949870
H 2.722595 -1.293329 2.118956
H 3.580816 -0.146304 1.123796
C 2.278411 2.902951 1.588511
H 1.881303 3.832356 1.172010
H 2.063830 2.935982 2.662909
C 3.475136 0.392036 3.240443
H 2.751599 1.038463 3.745781
H 3.653979 -0.424514 3.945455
C 3.806413 2.859060 1.335993
H 4.036293 2.107755 0.573961
H 4.100269 3.817784 0.898233
C 4.665886 2.612644 2.584722
H 5.674967 3.002179 2.401889
H 4.260597 3.210306 3.413301
C 4.795383 1.151853 3.033489
H 5.364221 1.140104 3.971494
H 5.407888 0.603429 2.304578

62
9a-TS(F->18a)
C -0.716056 -0.085875 0.245508
C 0.813946 -0.335518 0.174515
C -0.096726 2.286197 0.617114
C -1.006934 1.182243 1.086736
H -1.123518 0.035324 -0.758135
H -1.217179 -0.941195 0.711421
H -2.056718 1.470483 0.985699
C 1.424368 0.210525 -1.139930

C 1.745304 1.686032 -0.792725
H -0.396698 2.835991 -0.275864
H 1.045484 -1.396707 0.275133
C 1.404378 1.943452 0.689445
C 1.564528 0.496075 1.310284
C -0.656572 0.856529 2.544651
H -0.662125 1.761669 3.163638
H -1.352510 0.142007 2.993660
C 0.732141 0.249005 2.571330
O 1.099961 -0.475049 3.476594
O 2.182878 2.502235 -1.570407
O 2.596400 -0.531438 -1.389058
O 0.576276 0.229605 -2.261018
C 3.438314 -0.041117 -2.439430
H 4.058050 0.792766 -2.096173
H 4.076501 -0.879446 -2.724802
H 2.847480 0.292090 -3.296034
C 0.242353 -1.060074 -2.775472
H 1.136114 -1.616535 -3.069857
H -0.318452 -1.657511 -2.045941
H -0.385594 -0.881692 -3.649440
C 3.022947 -0.002696 1.468211
H 2.999639 -1.090334 1.351701
H 3.597271 0.352145 0.613285
C 2.195088 3.160237 1.211195
H 1.881479 3.990638 0.572622
H 1.843210 3.404059 2.217363
C 3.792359 0.284770 2.775983
H 3.460035 -0.433978 3.525586
H 4.850299 0.067772 2.574572
C 3.732438 3.074327 1.205505
H 4.072502 2.319915 0.492619
H 4.130864 4.021908 0.828517
H -0.374613 3.430813 1.573492
S -0.642273 4.596646 2.487935
C -2.481017 4.771736 2.223125
C -2.837139 6.151015 2.798346
H -3.916257 6.318938 2.702396
H -2.578092 6.218109 3.859094
H -2.315075 6.949003 2.264134
C -2.809514 4.727692 0.726519
H -2.582621 3.749370 0.293530
H -3.879483 4.915445 0.575753
H -2.245088 5.485152 0.177213
C -3.243492 3.678361 2.980701
H -3.009103 3.706207 4.047678
H -4.324414 3.822308 2.860090
H -2.995354 2.682463 2.605932
C 4.333202 2.817862 2.595814
H 4.244533 3.740645 3.184223
H 5.408804 2.623550 2.492539
C 3.687303 1.681310 3.392332
H 4.159805 1.647170 4.381521
H 2.634537 1.915464 3.589410

62
9a-TS(D->17a)
C -2.712114 -1.281102 0.096076
C -1.217815 -1.400598 -0.282799
C -1.884004 1.090190 -0.239470
C -3.125751 0.220016 0.095864
H -3.326689 -1.842721 -0.606399
H -2.869322 -1.725206 1.084083
H -3.946506 0.401467 -0.599462
C -1.056300 -0.796019 -1.703961
C -1.205875 0.733606 -1.534751
H -2.146776 2.150571 -0.270170
H -0.902460 -2.445584 -0.311903
C -0.958862 0.798598 0.994825
C -0.399554 -0.646454 0.792089
C -3.421323 0.691850 1.519299
H -3.864211 1.696492 1.529440
H -4.067226 0.035158 2.107218
C -2.028608 0.779545 2.137577
O -1.804993 0.805367 3.326751
O -0.814856 1.549374 -2.341657

O 0.153417 -1.238599 -2.222369
O -2.139533 -1.118340 -2.572899
C 0.592806 -0.658989 -3.459646
H 1.208058 0.220673 -3.265292
H 1.188421 -1.429017 -3.956006
H -0.256609 -0.385676 -4.087626
C -2.215016 -2.487544 -2.962775
H -1.273553 -2.830113 -3.400547
H -2.469764 -3.143596 -2.120406
H -3.009614 -2.544988 -3.708370
C -0.113289 -1.513175 2.013850
H -0.977899 -1.466608 2.690082
H -0.074072 -2.548031 1.656692
C 0.067074 1.945798 1.178466
H 0.836108 1.837313 0.408173
H -0.473707 2.862806 0.915585
C 1.153024 -1.296065 2.861538
H 1.211306 -2.176015 3.514481
H 2.028944 -1.341844 2.209311
C 0.729867 2.220506 2.537208
H 1.235744 3.185818 2.405066
H -0.041914 2.385977 3.293835
S 2.399148 -0.093877 -0.297653
H 0.982137 -0.468905 0.203296
C 3.262884 -1.740552 -0.545326
C 3.892674 -1.704510 -1.945063
H 4.478164 -2.617506 -2.110747
H 3.126533 -1.638935 -2.718965
H 4.564859 -0.848564 -2.056077
C 2.281442 -2.912255 -0.442602
H 2.817470 -3.847014 -0.647379
H 1.858826 -2.988949 0.561919
H 1.469896 -2.805833 -1.163024
C 4.366742 -1.872233 0.513250
H 3.950626 -1.901035 1.522963
H 4.931147 -2.799083 0.349532
H 5.066179 -1.033419 0.460101
C 1.774654 1.241322 3.088151
H 2.331713 1.797908 3.852017
H 2.499117 0.992518 2.305191
C 1.263472 -0.050827 3.753487
H 0.310270 0.137941 4.256492
H 1.981060 -0.310145 4.541559

44
9b-C
C -0.143035 -0.310466 0.129839
C 1.401154 -0.269596 0.130753
C 0.491729 2.137784 0.152299
C -0.701104 1.137855 0.129402
H -0.505058 -0.856733 -0.744041
H -0.478806 -0.854084 1.018448
H -1.265786 1.313248 -0.792317
C 1.880918 0.446595 -1.150935
C 1.341957 1.905122 -1.086648
H 0.133486 3.168388 0.161067
H 1.823967 -1.276505 0.159666
C 1.419063 1.822197 1.319807
C 1.876958 0.554802 1.315238
C -1.630098 1.380258 1.321170
H -2.457130 0.658648 1.343097
H -1.069576 1.258682 2.258449
C -2.227310 2.781539 1.365443
O -3.140020 3.152836 2.030964
O 1.610939 2.749045 -1.913810
O 3.285947 0.371745 -1.129962
O 1.334683 -0.072301 -2.345924
C 3.980699 1.078830 -2.162246
H 4.066029 2.143410 -1.927878
H 4.975555 0.630522 -2.212813
H 3.473004 0.971640 -3.124000
C 1.655324 -1.434617 -2.614429
H 2.724294 -1.624749 -2.478993
H 1.086118 -2.121342 -1.974604
H 1.380973 -1.614185 -3.655303
C 2.774651 -0.099816 2.332623

H 2.494587 -1.159920 2.386671
H 3.810386 -0.095108 1.961920
C 1.816204 2.944634 2.250000
H 1.710566 3.882982 1.693492
H 1.109580 3.026422 3.089951
C 2.695768 0.506783 3.740495
H 1.636414 0.650364 3.986903
H 3.080882 -0.216561 4.468332
C 3.257863 2.836239 2.770166
H 3.896761 2.560578 1.923047
H 3.598796 3.822658 3.104532
C 3.451203 1.831710 3.915562
H 4.524065 1.628053 4.021863
H 3.131874 2.288918 4.860530

44
9b-D
C -0.488795 -0.203542 0.282606
C 1.055035 -0.212000 0.235966
C 0.187853 2.234715 0.284054
C -0.988642 1.243487 0.540995
H -0.881862 -0.575104 -0.667039
H -0.839784 -0.874849 1.071814
H -1.843442 1.471873 -0.098874
C 1.482489 0.497718 -1.074497
C 0.937679 1.949182 -0.993320
H -0.159911 3.270829 0.265098
H 1.448487 -1.231754 0.234915
C 1.095602 1.984047 1.529261
C 1.626657 0.569397 1.387813
C -1.303798 1.498659 2.022741
H -1.962601 2.367786 2.145633
H -1.777142 0.658705 2.538591
C 0.036352 1.863487 2.656773
O 0.242624 2.025746 3.840874
O 1.138856 2.771873 -1.862703
O 2.891751 0.456795 -1.103894
O 0.914423 -0.042393 -2.245362
C 3.528924 1.127995 -2.198163
H 3.593028 2.204494 -2.018629
H 4.532651 0.702900 -2.262518
H 2.987289 0.957751 -3.131232
C 1.226838 -1.411600 -2.493612
H 2.292474 -1.609131 -2.343524
H 0.642301 -2.082910 -1.851922
H 0.963388 -1.601222 -3.535432
C 2.626829 -0.070398 2.296940
H 2.136849 -0.888774 2.853124
H 3.378563 -0.568522 1.664103
C 2.142134 3.097745 1.788065
H 1.871990 3.954798 1.161914
H 2.047512 3.426811 2.828566
C 3.322931 0.858545 3.297429
H 2.561472 1.362540 3.899201
H 3.910120 0.247731 3.992641
C 3.592550 2.689850 1.497098
H 3.617790 2.094108 0.579495
H 4.188676 3.588432 1.301964
C 4.244654 1.897902 2.644670
H 4.576129 2.596125 3.423566
H 5.149539 1.405453 2.266443

44
9b-E
C -0.567107 -0.230747 0.103636
C 0.963599 -0.370074 0.229993
C 0.280401 2.134806 0.277925
C -0.897453 1.176051 0.644074
H -0.862132 -0.349775 -0.940529
H -1.093179 -0.992765 0.687303
H -1.838091 1.560640 0.243065
C 1.527565 0.318692 -1.035629
C 0.980279 1.780439 -1.040017
H -0.015538 3.185525 0.256070
H 1.290757 -1.410589 0.275130
C 1.441699 1.855246 1.192718

C 1.469425 0.383610 1.534105
C -0.941859 1.029210 2.169783
H -0.980224 1.997472 2.684619
H -1.808274 0.448615 2.500376
C 0.330713 0.298218 2.599711
O 0.454135 -0.251939 3.673863
O 1.238788 2.580198 -1.917483
O 2.935463 0.277980 -0.967585
O 1.060370 -0.249889 -2.239357
C 3.644710 0.918942 -2.035189
H 3.669271 2.003311 -1.898957
H 4.659165 0.516654 -1.999601
H 3.184031 0.696221 -2.999874
C 1.380125 -1.626384 -2.427756
H 2.416545 -1.835726 -2.145985
H 0.710488 -2.278360 -1.852721
H 1.243316 -1.830290 -3.491165
C 2.763735 -0.220717 2.091529
H 2.495429 -1.202086 2.494710
H 3.459278 -0.384822 1.265491
C 2.417658 2.918230 1.579434
H 2.242292 3.775072 0.917752
H 2.205245 3.285956 2.598707
C 3.427453 0.612621 3.199859
H 2.648353 1.072320 3.815655
H 3.969743 -0.056697 3.876383
C 3.898690 2.500353 1.491023
H 4.029629 1.922461 0.572841
H 4.514289 3.401243 1.387892
C 4.401677 1.687396 2.693121
H 4.630387 2.366949 3.524091
H 5.352616 1.216427 2.414122

44
9b-F
C -0.887408 0.037537 0.207533
C 0.631394 -0.292161 0.177065
C -0.106332 2.325914 0.801799
C -1.139328 1.287916 1.093083
H -1.246302 0.224800 -0.804100
H -1.450892 -0.809480 0.613888
H -2.152327 1.670620 0.946130
C 1.307143 0.335336 -1.066755
C 1.643913 1.773689 -0.608538
H -0.341978 3.320494 0.440284
H 0.808669 -1.369088 0.193523
C 1.332097 1.896917 0.900096
C 1.358347 0.410018 1.399109
C -0.913490 0.840316 2.549583
H -0.927050 1.692353 3.239781
H -1.667659 0.124886 2.894297
C 0.450408 0.175301 2.620865
O 0.762612 -0.552983 3.542962
O 2.070715 2.654521 -1.323621
O 2.496023 -0.394176 -1.303455
O 0.521005 0.421051 -2.226413
C 3.369254 0.143580 -2.304004
H 3.963510 0.974225 -1.911485
H 4.029731 -0.675844 -2.593731
H 2.805045 0.497342 -3.170218
C 0.188629 -0.836781 -2.813627
H 1.084613 -1.417504 -3.049439
H -0.455220 -1.435161 -2.156795
H -0.354154 -0.607218 -3.731660
C 2.757769 -0.155744 1.693866
H 2.616594 -1.210549 1.944632
H 3.365460 -0.132156 0.788314
C 2.225985 2.946980 1.582307
H 1.978862 3.904310 1.112267
H 1.941522 3.034126 2.637523
C 3.488389 0.543077 2.855992
H 2.758324 0.856873 3.605719
H 4.120695 -0.190982 3.366986
C 3.736921 2.703684 1.433838
H 3.928811 2.346878 0.417986
H 4.253493 3.667526 1.500446

C 4.363127 1.739723 2.455437
H 5.314501 1.379436 2.044267
H 4.620044 2.291888 3.368160

45
18b
C -0.886548 0.082353 0.177628
C 0.627012 -0.288682 0.169544
C -0.170906 2.412444 0.851275
C -1.153800 1.268025 1.123282
H -1.208615 0.334146 -0.833672
H -1.478346 -0.778733 0.504838
H -2.182148 1.623213 1.000756
C 1.330189 0.306958 -1.072335
C 1.672686 1.746715 -0.612542
H -0.386362 2.901390 -0.104259
H 0.777111 -1.369367 0.203881
C 1.320876 1.902171 0.869969
C 1.355450 0.418585 1.386244
C -0.914411 0.784049 2.551818
H -0.955383 1.610969 3.272836
H -1.644390 0.036552 2.878947
C 0.465506 0.158522 2.613332
O 0.803061 -0.565437 3.530485
O 2.135253 2.604421 -1.330719
O 2.502058 -0.449564 -1.297381
O 0.558182 0.411473 -2.241062
C 3.398172 0.069283 -2.287403
H 4.010071 0.882479 -1.885122
H 4.040600 -0.765394 -2.574108
H 2.851506 0.440724 -3.157358
C 0.184249 -0.839860 -2.817414
H 1.059806 -1.462032 -3.022113
H -0.500635 -1.397709 -2.166615
H -0.326043 -0.602102 -3.751845
C 2.764599 -0.133604 1.671608
H 2.639084 -1.193762 1.907039
H 3.374270 -0.090997 0.768298
C 2.220025 2.947551 1.549309
H 1.975425 3.904376 1.075321
H 1.928133 3.039681 2.603106
C 3.487737 0.560651 2.841707
H 2.754310 0.870079 3.589470
H 4.118845 -0.174471 3.352556
C 3.734933 2.719307 1.420957
H 3.946285 2.367815 0.407429
H 4.239432 3.688988 1.496024
C 4.359871 1.761264 2.449107
H 5.317003 1.406878 2.046258
H 4.605717 2.315800 3.363451
H -0.283160 3.180939 1.624068

45
17b
C -0.114835 -0.352649 0.390820
C 1.363561 -0.205884 0.002000
C 0.294936 2.146694 0.265586
C -0.748780 1.049333 0.629755
H -0.654422 -0.876147 -0.400205
H -0.198890 -0.965464 1.293941
H -1.667123 1.163286 0.050635
C 1.439016 0.535116 -1.348915
C 0.878624 1.961723 -1.110666
H -0.143963 3.147096 0.324494
H 1.840202 -1.184329 -0.109118
C 1.354957 1.937321 1.397418
C 2.150137 0.625504 1.044306
C -0.959212 1.323980 2.121230
H -1.632072 2.175630 2.284759
H -1.352195 0.481289 2.696305
C 0.427934 1.731786 2.613961
O 0.721602 1.901243 3.778496
O 0.931052 2.838546 -1.948675
O 2.793316 0.540682 -1.731963
O 0.607776 -0.007470 -2.354717
C 3.128293 1.248931 -2.930665

H 3.231560 2.320783 -2.744039
H 4.083465 0.835438 -3.262203
H 2.368102 1.098809 -3.700735
C 0.904527 -1.353369 -2.721295
H 1.977201 -1.492369 -2.885503
H 0.563662 -2.067768 -1.961319
H 0.364677 -1.542000 -3.650709
C 2.624267 -0.210688 2.237175
H 1.770239 -0.675964 2.744830
H 3.209440 -1.037058 1.815505
C 2.315029 3.126644 1.547986
H 2.835921 3.236681 0.587702
H 1.723585 4.041578 1.678271
C 3.485365 0.543516 3.278023
H 2.854717 0.850212 4.115905
H 4.218686 -0.163066 3.682969
C 3.350178 3.057112 2.683115
H 4.005784 3.929033 2.566811
H 2.833173 3.177985 3.638969
H 3.049979 0.965038 0.526457
C 4.225757 1.794065 2.770512
H 4.719357 1.596541 1.809231
H 5.034659 2.010812 3.478286

44
9b-TS(C->D)
C -0.435659 -0.232112 0.332527
C 1.100269 -0.241626 0.212990
C 0.267844 2.199184 0.286863
C -0.938843 1.230688 0.481503
H -0.876152 -0.694012 -0.554413
H -0.733269 -0.830760 1.198291
H -1.700132 1.443345 -0.273656
C 1.484786 0.450676 -1.117173
C 0.953560 1.909717 -1.035065
H -0.053485 3.242523 0.305700
H 1.493149 -1.261111 0.218050
C 1.285959 1.889110 1.389294
C 1.728397 0.581564 1.319276
C -1.513882 1.498520 1.878700
H -2.075640 2.441499 1.885474
H -2.184663 0.700785 2.219939
C -0.360475 1.690079 2.847289
O -0.236858 1.314240 3.974646
O 1.116754 2.722654 -1.920235
O 2.891811 0.392395 -1.192815
O 0.867198 -0.093088 -2.261977
C 3.500870 1.073640 -2.294907
H 3.576859 2.147966 -2.105945
H 4.500229 0.644459 -2.394016
H 2.933136 0.918446 -3.215540
C 1.165567 -1.463883 -2.515059
H 2.237836 -1.660375 -2.420905
H 0.617560 -2.130718 -1.837146
H 0.846233 -1.662741 -3.539459
C 2.772489 -0.073121 2.174171
H 2.362859 -1.033777 2.521798
H 3.624781 -0.341794 1.529984
C 2.091813 3.042943 1.978164
H 1.828894 3.943455 1.412009
H 1.798184 3.242195 3.014442
C 3.246243 0.737190 3.384879
H 2.365595 1.083568 3.935681
H 3.788762 0.072285 4.066591
C 3.610262 2.815235 1.897655
H 3.839415 2.362412 0.926125
H 4.129507 3.780210 1.922457
C 4.150012 1.926328 3.031025
H 5.146583 1.561257 2.751948
H 4.286913 2.534308 3.934116

44
9b-TS(C->E)
C -0.458101 -0.338002 0.239832
C 1.080816 -0.376450 0.187660
C 0.280840 2.076747 0.257805

C -0.870915 1.090462 0.652774
H -0.854290 -0.596764 -0.744673
H -0.837917 -1.067662 0.959796
H -1.799881 1.392675 0.162520
C 1.473373 0.308441 -1.148461
C 0.855229 1.740031 -1.114638
H -0.049212 3.116898 0.271894
H 1.465755 -1.397950 0.210426
C 1.449677 1.813420 1.185955
C 1.715629 0.456007 1.313304
C -1.031673 1.120620 2.172592
H -1.222890 2.127306 2.567043
H -1.876001 0.485394 2.468583
C 0.207236 0.532141 2.839858
O 0.548210 0.682480 3.982540
O 0.916904 2.508804 -2.049936
O 2.883241 0.335301 -1.199537
O 0.924209 -0.298647 -2.294375
C 3.464072 1.041489 -2.300812
H 3.452524 2.122118 -2.130584
H 4.495867 0.690720 -2.368305
H 2.931876 0.826275 -3.230366
C 1.302654 -1.657385 -2.500634
H 2.376022 -1.798544 -2.341843
H 0.747834 -2.336375 -1.840670
H 1.053090 -1.891132 -3.536984
C 2.927224 -0.155373 1.981092
H 2.618875 -1.125835 2.388380
H 3.647306 -0.375599 1.180717
C 2.213401 2.940676 1.819148
H 1.869917 3.877840 1.368852
H 1.977209 3.014656 2.891908
C 3.599469 0.660932 3.092446
H 2.833281 0.982906 3.800105
H 4.259638 -0.009995 3.654370
C 3.735558 2.796408 1.612897
H 3.896280 2.422609 0.595797
H 4.205434 3.785485 1.658575
C 4.429620 1.864268 2.623547
H 4.708858 2.441972 3.513527
H 5.370678 1.513359 2.181510

44
9b-TS(D->E)
C -0.590853 -0.212428 0.059503
C 0.935649 -0.324632 0.187155
C 0.233210 2.169536 0.334022
C -0.974356 1.188392 0.573599
H -0.876712 -0.356090 -0.984602
H -1.094716 -0.979506 0.653470
H -1.881142 1.567548 0.098624
C 1.496811 0.426798 -1.049161
C 0.936508 1.880876 -0.982051
H -0.074967 3.216082 0.367933
H 1.274689 -1.362552 0.180606
C 1.252390 1.831330 1.426821
C 1.480002 0.385375 1.449333
C -1.074354 1.090249 2.090170
H -1.478424 2.001154 2.547633
H -1.696443 0.249544 2.412640
C 0.366226 0.948824 2.560247
O 0.697345 0.951358 3.772750
O 1.113295 2.692056 -1.865000
O 2.905026 0.406710 -0.967652
O 1.044933 -0.086427 -2.279829
C 3.610118 1.084309 -2.015676
H 3.589387 2.168436 -1.874252
H 4.638808 0.724008 -1.959046
H 3.180249 0.849396 -2.991682
C 1.376494 -1.451323 -2.527531
H 2.413185 -1.665261 -2.250209
H 0.709196 -2.133027 -1.985561
H 1.246073 -1.607996 -3.599540
C 2.708190 -0.287929 2.048343
H 2.361369 -1.192371 2.563686
H 3.310030 -0.624130 1.196612

C 2.214241 2.889415 1.925769
H 1.869885 3.855606 1.542019
H 2.155383 2.948423 3.017726
C 3.597049 0.518454 3.002458
H 2.973771 0.925064 3.799340
H 4.287345 -0.185585 3.482836
C 3.663593 2.636582 1.476381
H 3.642171 2.276268 0.445733
H 4.207145 3.588043 1.461487
C 4.435565 1.630718 2.355445
H 4.936332 2.174408 3.166218
H 5.235287 1.184608 1.750766

44
9b-TS(E->F)
C -0.504374 -0.356864 0.110866
C 1.032772 -0.439421 0.227696
C 0.068565 2.065742 0.475590
C -0.949183 0.979681 0.742631
H -0.801223 -0.404388 -0.936029
H -0.985237 -1.186389 0.639425
H -1.944572 1.275286 0.404156
C 1.643810 0.313019 -0.974270
C 1.516557 1.782370 -0.568776
H -0.247705 3.080246 0.263127
H 1.394023 -1.467927 0.274212
C 1.424020 1.839974 0.995454
C 1.527508 0.374696 1.498911
C -0.900792 0.759780 2.268935
H -1.031391 1.698173 2.821859
H -1.671349 0.063988 2.614773
C 0.467417 0.174233 2.603168
O 0.681810 -0.433548 3.632553
O 1.829245 2.763752 -1.279380
O 3.008747 -0.057882 -1.051692
O 0.998377 0.119486 -2.208121
C 3.794707 0.650559 -2.016825
H 4.008136 1.671702 -1.689228
H 4.724887 0.086980 -2.111247
H 3.284106 0.696999 -2.982163
C 1.073099 -1.209691 -2.718169
H 2.105727 -1.568214 -2.752475
H 0.477443 -1.909550 -2.117218
H 0.662283 -1.172278 -3.728330
C 2.903588 -0.088278 1.998892
H 2.765959 -1.114543 2.351636
H 3.594488 -0.129266 1.155586
C 2.198713 2.984134 1.628184
H 1.921309 3.885965 1.074961
H 1.865300 3.130181 2.663585
C 3.487894 0.756390 3.144205
H 2.676246 1.089118 3.797281
H 4.113615 0.114031 3.773293
C 3.721615 2.795220 1.556793
H 3.957617 2.332688 0.593078
H 4.205072 3.777911 1.540999
C 4.328176 1.961997 2.697725
H 5.321619 1.619841 2.381932
H 4.493341 2.605883 3.570858

59
9b-TS(F->18b)
C -0.684560 -0.623617 0.153278
C 0.870444 -0.588966 0.219225
C -0.558141 1.853321 0.490221
C -1.285012 0.592242 0.885816
H -1.018195 -0.619719 -0.884107
H -1.056704 -1.541480 0.619221
H -2.353732 0.680923 0.673290
C 1.481942 0.000431 -1.075438
C 1.492163 1.527554 -0.802327
H -0.982236 2.010859 -0.965692
H 1.284716 -1.581631 0.403695
C 0.968192 1.782507 0.630091
C 1.330543 0.423965 1.345184
C -1.027943 0.401286 2.389549

H -1.283392 1.301286 2.963598
H -1.602356 -0.428931 2.813917
C 0.450399 0.109649 2.568060
O 0.886006 -0.419191 3.572236
O 1.874987 2.367322 -1.579857
O 2.797948 -0.503270 -1.172999
O 0.760430 -0.203695 -2.258619
C 3.610196 0.060182 -2.210972
H 4.042081 1.016914 -1.902723
H 4.408467 -0.662814 -2.389651
H 3.030536 0.216153 -3.123563
C 0.695036 -1.559932 -2.698949
H 1.693512 -1.971840 -2.869862
H 0.169597 -2.197211 -1.977358
H 0.135430 -1.542772 -3.634591
C 2.810806 0.289096 1.742416
H 2.940248 -0.735141 2.102195
H 3.446558 0.385626 0.860714
C 1.522047 3.101407 1.198259
H 1.090415 3.894775 0.578130
H 1.127827 3.247551 2.211726
C 3.256570 1.270285 2.844020
H 2.421950 1.452152 3.524568
H 4.020880 0.783983 3.459517
C 3.051526 3.257762 1.179740
H 3.425946 2.870256 0.229615
H 3.284923 4.328122 1.161903
H -0.994913 2.785032 0.850930
S -1.625747 2.099252 -2.317131
C -2.622615 3.631307 -1.965098
C -1.690005 4.787487 -1.582607
H -2.270413 5.706661 -1.433835
H -1.153043 4.577715 -0.653048
H -0.948060 4.965049 -2.364423
C -3.348423 3.956393 -3.279371
H -4.008746 3.137991 -3.580557
H -3.959649 4.857036 -3.148164
H -2.636432 4.141710 -4.088304
C -3.647946 3.368521 -0.855083
H -3.160097 3.123412 0.092792
H -4.263026 4.262045 -0.690009
H -4.308151 2.539214 -1.121513
C 3.814991 2.611562 2.347775
H 3.836769 3.303742 3.198807
H 4.859779 2.484408 2.038049

59
9b-TS(D->17b)
C -0.380895 -0.254627 0.223018
C 1.117587 -0.409961 -0.116087
C 0.558940 2.103118 0.154847
C -0.739679 1.260685 0.311881
H -0.996766 -0.746486 -0.528588
H -0.586795 -0.753719 1.175853
H -1.493195 1.532057 -0.428024
C 1.349824 0.325261 -1.468144
C 1.309627 1.836339 -1.127261
H 0.349444 3.174337 0.218061
H 1.396532 -1.460379 -0.230304
C 1.371576 1.624161 1.404991
C 1.927641 0.226344 1.037234
C -1.128265 1.605952 1.746419
H -1.500096 2.635204 1.835105
H -1.862333 0.941275 2.208016
C 0.214286 1.519830 2.466491
O 0.345052 1.408782 3.663730
O 1.825549 2.702439 -1.799810
O 2.521099 -0.162009 -2.023319
O 0.254278 0.191500 -2.373730
C 3.043603 0.513271 -3.176339
H 3.690140 1.336589 -2.871971
H 3.626017 -0.235719 -3.719041
H 2.237140 0.887533 -3.810355
C 0.094005 -1.107302 -2.938438
H 0.996891 -1.429952 -3.463609
H -0.156159 -1.860423 -2.180056

H -0.733970 -1.027667 -3.644777
C 2.281127 -0.744343 2.149647
H 1.364179 -1.289323 2.426508
H 2.931672 -1.505656 1.704882
C 2.438595 2.639016 1.861631
H 3.171617 2.735340 1.055441
H 1.945379 3.616273 1.938096
C 2.921654 -0.209047 3.452452
H 2.128377 0.031331 4.162273
H 3.483183 -1.043908 3.888900
C 3.156925 2.364699 3.192918
H 3.912308 3.152285 3.304737
H 2.446359 2.494459 4.013534
S 4.719823 0.827939 -0.062693
H 3.286452 0.483849 0.466757
C 5.596173 -0.821586 -0.076934
C 4.742892 -1.920878 -0.716267
H 5.323502 -2.850958 -0.755998
H 3.838135 -2.117303 -0.135948
H 4.432597 -1.652090 -1.725180
C 6.017836 -1.228916 1.340468
H 6.604422 -2.155439 1.301800
H 6.630173 -0.453144 1.806778
H 5.153051 -1.408706 1.982788
C 6.847316 -0.572722 -0.935883
H 7.466328 0.226202 -0.517100
H 7.452588 -1.486472 -0.971351
H 6.575188 -0.298928 -1.958526
C 3.856951 1.006827 3.355438
H 4.424397 1.050001 4.293561
H 4.590144 0.874719 2.558096

41
9c-C
C -0.119017 -0.344993 0.116588
C 1.425210 -0.266924 0.095452
C 0.437381 2.126269 0.121395
C -0.724605 1.086746 0.108637
H -0.478599 -0.910422 -0.745842
H -0.427434 -0.887362 1.015591
H -1.298238 1.236428 -0.812182
C 1.861438 0.460190 -1.197395
C 1.281582 1.905506 -1.126448
H 0.052243 3.147331 0.129564
H 1.875094 -1.262355 0.123447
C 1.366005 1.823597 1.282272
C 1.893132 0.587540 1.255372
C -1.657613 1.299923 1.304172
H -2.465329 0.556765 1.325033
H -1.087615 1.187613 2.236999
C -2.280031 2.688607 1.365593
O -3.258282 3.014223 1.957671
O 1.512739 2.749975 -1.963953
O 3.267600 0.424436 -1.202906
O 1.307423 -0.074790 -2.381403
C 3.924236 1.147368 -2.249257
H 3.987079 2.214058 -2.017540
H 4.928966 0.724349 -2.318454
H 3.400862 1.025856 -3.200801
C 1.661503 -1.427719 -2.655500
H 2.738246 -1.586572 -2.543600
H 1.126827 -2.129341 -2.002229
H 1.369381 -1.616970 -3.689776
C 2.904368 0.091285 2.248319
H 2.442613 -0.663034 2.904052
H 3.704375 -0.428656 1.707292
C 1.679882 2.849369 2.335008
H 2.312114 3.637841 1.900331
H 0.756540 3.353922 2.649120
C 3.484230 1.243159 3.082495
H 4.033292 0.847837 3.943495
H 4.207405 1.794851 2.468567
C 2.382508 2.207721 3.540288
H 2.795372 2.983920 4.192932
H 1.643699 1.653491 4.134320

41
9c-D
C -0.530620 -0.218927 0.245126
C 1.013846 -0.280843 0.181275
C 0.201654 2.202438 0.234020
C -0.987627 1.237742 0.539154
H -0.940176 -0.555395 -0.711283
H -0.899102 -0.896109 1.020964
H -1.864528 1.487257 -0.061719
C 1.445060 0.427314 -1.132143
C 0.887745 1.875995 -1.070483
H -0.117435 3.248193 0.217067
H 1.376131 -1.311745 0.172757
C 1.138678 1.917458 1.439632
C 1.619529 0.491377 1.311175
C -1.234275 1.492131 2.035821
H -1.862970 2.379137 2.186166
H -1.711228 0.663167 2.565968
C 0.143399 1.811181 2.616657
O 0.418027 1.920333 3.794092
O 1.037760 2.667578 -1.978106
O 2.857130 0.418633 -1.133389
O 0.907044 -0.132442 -2.304811
C 3.498179 1.085882 -2.226181
H 3.505348 2.170062 -2.082958
H 4.522561 0.708455 -2.243612
H 2.999111 0.861630 -3.171928
C 1.243221 -1.499120 -2.534290
H 2.305695 -1.683305 -2.348387
H 0.645168 -2.172935 -1.907946
H 1.016445 -1.698266 -3.582972
C 2.721645 0.019775 2.193892
H 2.347949 -0.113744 3.223278
H 3.083443 -0.956505 1.855102
C 2.278455 2.927743 1.642718
H 2.744642 3.105489 0.665393
H 1.858059 3.883422 1.976413
C 3.872083 1.048122 2.238192
H 4.638089 0.717455 2.948513
H 4.336878 1.092427 1.246080
C 3.344005 2.432655 2.629837
H 4.167130 3.155486 2.663721
H 2.907142 2.388164 3.632319

41
9c-E
C -0.561583 -0.294445 0.130056
C 0.977324 -0.362902 0.193243
C 0.172825 2.114646 0.285978
C -0.939437 1.089310 0.699894
H -0.891955 -0.419582 -0.903256
H -1.027269 -1.085762 0.725835
H -1.915741 1.426700 0.344213
C 1.456794 0.369063 -1.085384
C 0.808912 1.790430 -1.071603
H -0.174944 3.149700 0.286365
H 1.354759 -1.387333 0.211274
C 1.375094 1.871449 1.147717
C 1.494892 0.406084 1.472058
C -0.909188 0.928900 2.224558
H -1.012256 1.884729 2.753425
H -1.714252 0.276739 2.577554
C 0.435342 0.306257 2.610466
O 0.677740 -0.128798 3.717612
O 0.946839 2.587284 -1.976746
O 2.864299 0.438358 -1.047264
O 1.003756 -0.228284 -2.279093
C 3.499256 1.162288 -2.106698
H 3.435725 2.241740 -1.943528
H 4.544535 0.846950 -2.095651
H 3.044602 0.926045 -3.071400
C 1.429344 -1.572467 -2.488404
H 2.487222 -1.697715 -2.238080
H 0.832510 -2.281352 -1.900667
H 1.279052 -1.780122 -3.549271
C 2.875936 -0.046279 1.957957

H 2.749382 -0.943858 2.570723
H 3.477085 -0.306322 1.084755
C 2.296022 2.915973 1.669603
H 2.344461 3.752359 0.959883
H 1.883658 3.344190 2.602015
C 3.597351 1.043299 2.761857
H 3.061947 1.221615 3.702426
H 4.599144 0.691247 3.031299
C 3.695022 2.348936 1.963628
H 4.207398 2.153472 1.014043
H 4.286623 3.093545 2.506717

41
9c-F
C -0.850100 0.012413 0.235966
C 0.672118 -0.289543 0.186408
C -0.102902 2.329475 0.751235
C -1.110246 1.279301 1.095226
H -1.232573 0.169300 -0.771919
H -1.389896 -0.834812 0.673191
H -2.134027 1.636046 0.957676
C 1.331480 0.332864 -1.076871
C 1.712948 1.767062 -0.623265
H -0.359515 3.307662 0.360683
H 0.869469 -1.362912 0.211625
C 1.338876 1.921050 0.861303
C 1.395740 0.445684 1.382576
C -0.852400 0.872969 2.559210
H -0.917186 1.731104 3.238117
H -1.567659 0.122501 2.914311
C 0.549575 0.289751 2.652908
O 0.951781 -0.271052 3.653755
O 2.216104 2.616033 -1.325901
O 2.486681 -0.431662 -1.356858
O 0.511408 0.451753 -2.211296
C 3.358444 0.094205 -2.365472
H 4.004677 0.879445 -1.962458
H 3.966863 -0.747892 -2.701089
H 2.789467 0.505780 -3.202426
C 0.156516 -0.788573 -2.822607
H 1.040655 -1.340539 -3.152933
H -0.421762 -1.427961 -2.144092
H -0.461022 -0.532683 -3.684788
C 2.835650 -0.019942 1.643703
H 2.793420 -0.978196 2.168557
H 3.337938 -0.192605 0.690678
C 2.232165 2.919159 1.604820
H 2.244766 3.855972 1.038289
H 1.781858 3.132555 2.581966
C 3.634113 0.998537 2.468386
H 3.196297 1.082578 3.469366
H 4.657559 0.630916 2.602082
C 3.652443 2.374583 1.793399
H 4.141186 2.300665 0.813550
H 4.240513 3.085082 2.384617

42
18c
C -0.842985 0.016454 0.231322
C 0.679458 -0.294693 0.187448
C -0.170007 2.391120 0.744867
C -1.118594 1.243822 1.119271
H -1.215122 0.200473 -0.776893
H -1.386525 -0.846825 0.629595
H -2.158247 1.566592 1.002429
C 1.356756 0.309739 -1.075172
C 1.771082 1.736000 -0.610388
H -0.394596 2.762780 -0.260326
H 0.870555 -1.368832 0.224586
C 1.330631 1.923130 0.837215
C 1.398742 0.452099 1.376266
C -0.836861 0.847752 2.568352
H -0.908344 1.706038 3.248762
H -1.533701 0.088910 2.940386
C 0.571904 0.287445 2.654127
O 0.993054 -0.263673 3.653128

O 2.353674 2.544867 -1.296797
O 2.480068 -0.493194 -1.365199
O 0.537144 0.473860 -2.206143
C 3.372814 0.008902 -2.367625
H 4.058206 0.753010 -1.951794
H 3.937963 -0.855387 -2.722292
H 2.822071 0.464468 -3.193829
C 0.142261 -0.744647 -2.837407
H 1.007178 -1.305633 -3.201530
H -0.432423 -1.388401 -2.160429
H -0.489533 -0.455917 -3.678657
C 2.847550 0.002790 1.627074
H 2.820161 -0.963427 2.138169
H 3.351832 -0.153092 0.671769
C 2.204238 2.919259 1.599472
H 2.209423 3.866422 1.049170
H 1.739594 3.113179 2.575209
C 3.634421 1.020696 2.463264
H 3.196939 1.085088 3.465478
H 4.662981 0.665128 2.590638
C 3.632896 2.404797 1.805104
H 4.138642 2.354637 0.832922
H 4.195876 3.120240 2.414580
H -0.314510 3.234696 1.428206

42
17c
C -0.587907 -0.199861 0.214021
C 0.950136 -0.303384 0.235550
C 0.206942 2.187508 0.271247
C -1.025350 1.264939 0.497355
H -0.960655 -0.514679 -0.763227
H -1.015085 -0.875950 0.961949
H -1.855236 1.541054 -0.156330
C 1.485046 0.402890 -1.021961
C 0.990213 1.869189 -0.972669
H -0.077885 3.243675 0.242078
H 1.268242 -1.349937 0.219207
C 1.051256 1.894493 1.547447
C 1.458138 0.350670 1.542200
C -1.363447 1.542825 1.976313
H -2.059177 2.383432 2.079152
H -1.808692 0.693797 2.503509
C -0.036724 1.967973 2.621759
O 0.118013 2.314898 3.771517
O 1.267400 2.683119 -1.830187
O 2.890445 0.320086 -0.982972
O 0.962805 -0.113071 -2.229061
C 3.604332 0.967563 -2.042844
H 3.690010 2.042966 -1.865504
H 4.597249 0.513213 -2.051097
H 3.109227 0.808477 -3.003175
C 1.233177 -1.492115 -2.468554
H 2.271765 -1.740808 -2.229527
H 0.564392 -2.143213 -1.891461
H 1.055107 -1.658650 -3.532381
C 2.918414 0.013300 1.954799
H 2.889368 -0.354107 2.987510
H 3.270390 -0.816356 1.335550
C 2.176341 2.919806 1.749468
H 2.554674 3.224104 0.765703
H 1.740504 3.812907 2.209815
C 3.928837 1.160422 1.910508
H 4.850374 0.839132 2.409073
H 4.187650 1.392035 0.872212
C 3.351582 2.406885 2.577781
H 4.109820 3.193508 2.658616
H 3.015159 2.176272 3.595921
H 0.842248 -0.128104 2.313460

41
9c-TS(C->D)
C -0.399248 -0.187110 0.377389
C 1.130065 -0.242513 0.181555
C 0.344073 2.232320 0.203998
C -0.865693 1.291538 0.508475

H -0.891693 -0.661761 -0.474487
H -0.669495 -0.752975 1.273354
H -1.675172 1.495715 -0.197181
C 1.449784 0.392587 -1.195836
C 0.952868 1.867615 -1.140096
H 0.046094 3.283116 0.194717
H 1.499881 -1.270052 0.205399
C 1.397034 1.926813 1.262674
C 1.832881 0.618308 1.205098
C -1.333085 1.604732 1.935825
H -1.824231 2.585314 1.970252
H -2.033735 0.854523 2.321243
C -0.101402 1.698743 2.823551
O 0.094022 1.211876 3.899468
O 1.081761 2.639358 -2.066433
O 2.846628 0.297683 -1.349656
O 0.753710 -0.177554 -2.281407
C 3.406495 0.922275 -2.509168
H 3.514603 2.001333 -2.368028
H 4.390052 0.467737 -2.646225
H 2.785857 0.745210 -3.391059
C 1.011860 -1.561889 -2.503137
H 2.084299 -1.775911 -2.468643
H 0.496052 -2.193671 -1.768691
H 0.625253 -1.790092 -3.497794
C 2.923388 0.101297 2.088771
H 2.468519 -0.319194 3.001772
H 3.444800 -0.723991 1.591248
C 2.257929 3.021615 1.852180
H 2.867015 3.450358 1.041634
H 1.630266 3.832520 2.236087
C 3.911432 1.212683 2.478725
H 4.592472 0.852199 3.257125
H 4.524611 1.459491 1.602577
C 3.173357 2.471059 2.953650
H 3.889730 3.241153 3.259628
H 2.564900 2.225231 3.831006

41
9c-TS(C->E)
C -0.439806 -0.344001 0.221443
C 1.100813 -0.375718 0.151527
C 0.275929 2.082651 0.266484
C -0.860341 1.074563 0.665221
H -0.844435 -0.586796 -0.763655
H -0.809120 -1.088292 0.931705
H -1.801024 1.373236 0.195636
C 1.470560 0.335135 -1.178072
C 0.838092 1.762108 -1.116873
H -0.062869 3.119903 0.295994
H 1.492880 -1.394885 0.155068
C 1.447461 1.803508 1.176624
C 1.728369 0.450697 1.278104
C -0.996694 1.075498 2.189224
H -1.179159 2.075144 2.605913
H -1.838360 0.437140 2.485867
C 0.251811 0.470345 2.823722
O 0.625445 0.603259 3.958160
O 0.878562 2.540467 -2.044897
O 2.878169 0.374369 -1.243135
O 0.912881 -0.257655 -2.327683
C 3.443324 1.108950 -2.333106
H 3.421621 2.185909 -2.141441
H 4.478315 0.770899 -2.415239
H 2.907286 0.907406 -3.263699
C 1.302032 -1.608670 -2.561953
H 2.378890 -1.741361 -2.420457
H 0.763463 -2.304324 -1.905817
H 1.040229 -1.827759 -3.598490
C 2.980828 -0.047246 1.955417
H 2.763255 -0.968583 2.507327
H 3.680752 -0.313308 1.152758
C 2.189125 2.856080 1.937983
H 2.190139 3.792377 1.367068
H 1.661761 3.071546 2.882536
C 3.614353 0.998517 2.886801

H 3.048271 1.034002 3.822044
H 4.635188 0.690092 3.137099
C 3.621631 2.393603 2.249054
H 4.196833 2.369110 1.314814
H 4.111518 3.113985 2.912703

41
9c-TS(D->E)
C -0.682623 -0.101510 0.122500
C 0.844376 -0.234424 0.236517
C 0.167538 2.283938 0.183164
C -1.040749 1.340977 0.534440
H -0.984537 -0.317357 -0.905338
H -1.192383 -0.813222 0.777381
H -1.958173 1.687081 0.054596
C 1.405904 0.414835 -1.055119
C 0.840496 1.868053 -1.115523
H -0.124029 3.335046 0.132584
H 1.165163 -1.275743 0.305201
C 1.203664 2.010507 1.270071
C 1.412895 0.569222 1.421075
C -1.100724 1.375865 2.058667
H -1.487273 2.327594 2.442061
H -1.721220 0.573058 2.469358
C 0.349839 1.254039 2.501795
O 0.720846 1.351193 3.701600
O 1.001388 2.593866 -2.072367
O 2.812287 0.417287 -0.945281
O 0.975608 -0.200111 -2.244222
C 3.531565 1.055129 -2.006453
H 3.505772 2.144408 -1.908455
H 4.560852 0.702138 -1.920257
H 3.119417 0.781146 -2.980353
C 1.332183 -1.574073 -2.381965
H 2.369398 -1.747283 -2.079329
H 0.670738 -2.223348 -1.794867
H 1.215741 -1.815824 -3.439680
C 2.689139 0.074404 2.090583
H 2.460136 -0.187985 3.127638
H 3.016291 -0.834610 1.578572
C 2.249793 3.037045 1.615446
H 2.705662 3.354124 0.665303
H 1.775136 3.928608 2.041902
C 3.821159 1.114014 2.070093
H 4.644879 0.763894 2.701754
H 4.206908 1.196612 1.048070
C 3.339517 2.488381 2.546327
H 4.174015 3.197336 2.578430
H 2.936697 2.403055 3.559207

41
9c-TS(E->F)
C -0.472866 -0.370201 0.141010
C 1.063946 -0.429053 0.255759
C 0.075546 2.075203 0.419984
C -0.930700 0.982190 0.727428
H -0.772490 -0.459279 -0.902410
H -0.942415 -1.185521 0.700951
H -1.930351 1.253661 0.381384
C 1.661004 0.306885 -0.967582
C 1.511654 1.784385 -0.595528
H -0.253550 3.082280 0.191336
H 1.442160 -1.450444 0.324087
C 1.418989 1.873427 0.966488
C 1.543680 0.425723 1.499192
C -0.879217 0.813268 2.260705
H -1.049247 1.761213 2.785498
H -1.628423 0.101918 2.622434
C 0.508378 0.292496 2.631246
O 0.763766 -0.175908 3.722082
O 1.830963 2.756374 -1.321422
O 3.026380 -0.054822 -1.047639
O 1.009575 0.082215 -2.194000
C 3.814835 0.678308 -1.991433
H 4.056815 1.677741 -1.618568
H 4.730842 0.099558 -2.125975

H 3.291430 0.781150 -2.945374
C 1.109450 -1.251160 -2.687802
H 2.150471 -1.581839 -2.740574
H 0.546726 -1.958833 -2.064456
H 0.675417 -1.238738 -3.688836
C 2.953041 0.076106 1.991889
H 2.882848 -0.825334 2.607285
H 3.576244 -0.150972 1.126140
C 2.193363 2.977140 1.654294
H 2.198339 3.853551 0.998895
H 1.666010 3.258352 2.575994
C 3.593301 1.214394 2.797050
H 3.037305 1.364448 3.730507
H 4.611627 0.926559 3.080314
C 3.615898 2.521500 1.995486
H 4.179195 2.372004 1.065071
H 4.129999 3.310041 2.555566

56
9c-TS(F->18c)
C -0.767956 -0.118317 0.194477
C 0.761995 -0.380277 0.174354
C -0.146062 2.257002 0.625922
C -1.085553 1.147024 1.035783
H -1.141002 0.012249 -0.820956
H -1.292297 -0.972029 0.637288
H -2.127973 1.441892 0.887262
C 1.421489 0.202187 -1.107867
C 1.761727 1.663823 -0.704745
H -0.405232 2.827929 -0.266063
H 0.987913 -1.445339 0.251598
C 1.337303 1.881819 0.756624
C 1.449413 0.430521 1.341943
C -0.809874 0.797308 2.502929
H -0.909471 1.674130 3.152896
H -1.494105 0.030068 2.879749
C 0.611419 0.277999 2.617140
O 1.037792 -0.237451 3.631812
O 2.272560 2.486242 -1.429317
O 2.585097 -0.554268 -1.357310
O 0.603644 0.276900 -2.249251
C 3.460267 -0.046665 -2.373029
H 4.096794 0.753544 -1.984421
H 4.078340 -0.891773 -2.681961
H 2.894305 0.338352 -3.224449
C 0.271122 -0.986140 -2.827390
H 1.165123 -1.526683 -3.149381
H -0.288871 -1.620217 -2.129105
H -0.356604 -0.764551 -3.691603
C 2.906955 0.028073 1.617297
H 2.896201 -0.910337 2.178169
H 3.415695 -0.165811 0.671776
C 2.202733 2.931927 1.466442
H 2.173210 3.856816 0.882946
H 1.757165 3.148203 2.443670
C 3.671810 1.103014 2.399080
H 3.231427 1.214445 3.396150
H 4.706562 0.774301 2.545689
C 3.641541 2.447931 1.665976
H 4.133846 2.352341 0.689961
H 4.201107 3.204435 2.226630
H -0.423012 3.396772 1.601281
S -0.676400 4.550548 2.522553
C -2.486837 4.817717 2.157416
C -2.816844 6.193574 2.755464
H -3.880153 6.412533 2.602881
H -2.618084 6.217351 3.830967
H -2.230439 6.983014 2.278298
C -2.728763 4.831631 0.643879
H -2.522646 3.855557 0.195529
H -3.778273 5.074547 0.438012
H -2.098681 5.575911 0.150881
C -3.338172 3.736383 2.833179
H -3.166241 3.720506 3.912224
H -4.403127 3.931121 2.655262
H -3.110121 2.742890 2.439100

56

9c-TS(D->17c)

C -0.529827 0.098886 0.519150
C 0.949808 -0.206421 0.199388
C 0.567098 2.367386 0.327541
C -0.716364 1.618557 0.791309
H -1.147014 -0.202554 -0.328297
H -0.839210 -0.486837 1.386726
H -1.602472 1.991720 0.274246
C 1.245315 0.426054 -1.191281
C 1.021343 1.954060 -1.048336
H 0.432093 3.452992 0.341278
H 1.131108 -1.283627 0.148069
C 1.581505 1.930964 1.431862
C 1.851992 0.417003 1.258674
C -0.753060 1.953974 2.290808
H -1.247418 2.914300 2.481540
H -1.249886 1.201877 2.908211
C 0.711241 2.109145 2.692079
O 1.119407 2.357735 3.806378
O 1.228160 2.732224 -1.956529
O 2.573131 0.114210 -1.532771
O 0.341217 0.020146 -2.194713
C 3.064381 0.640387 -2.772878
H 3.375078 1.682746 -2.666494
H 3.923837 0.020362 -3.035860
H 2.303157 0.578592 -3.553325
C 0.320397 -1.382011 -2.461964
H 1.334000 -1.788620 -2.524761
H -0.241875 -1.930412 -1.696326
H -0.181635 -1.500998 -3.423425
C 3.308431 0.012949 1.330260
H 3.371713 -1.077004 1.417075
H 3.786233 0.273843 0.378950
C 2.881484 2.742865 1.449773
H 3.300073 2.708335 0.437122
H 2.644465 3.793220 1.650834
C 4.029037 0.688287 2.500095
H 3.593174 0.314623 3.428917
H 5.078040 0.376748 2.505089
C 3.924249 2.234473 2.460123
H 4.891705 2.674555 2.196411
H 3.665154 2.606643 3.452928
S 0.949039 -1.583429 3.278807
H 1.375458 -0.327420 2.450606
C 0.828944 -1.111474 5.086246
C -0.409253 -0.249709 5.357825
H -0.540180 -0.127924 6.440440
H -0.296021 0.747396 4.929868
H -1.311209 -0.719197 4.955962
C 2.093900 -0.395411 5.566286
H 2.029212 -0.227751 6.648620
H 2.986007 -0.994417 5.365785
H 2.199509 0.579286 5.086137
C 0.687625 -2.468291 5.797579
H 1.560819 -3.102359 5.622657
H 0.595263 -2.298915 6.876917
H -0.204195 -3.005542 5.461801

38

9d-C

C -0.095610 -0.338318 0.162647
C 1.452240 -0.277336 0.120709
C 0.454064 2.150662 0.142826
C -0.699748 1.096800 0.144658
H -0.469608 -0.910767 -0.688984
H -0.396691 -0.867611 1.071570
H -1.273963 1.236170 -0.777277
C 1.867467 0.456031 -1.179830
C 1.287988 1.906902 -1.113790
H 0.061632 3.168613 0.139030
H 1.896550 -1.274831 0.141036
C 1.398747 1.834778 1.274558
C 1.913479 0.596774 1.255499
C -1.633213 1.321728 1.336340
H -2.425869 0.563414 1.378606

H -1.062430 1.247116 2.272340
C -2.293901 2.694836 1.351869
O -3.227940 3.035329 2.003982
O 1.512901 2.738999 -1.964480
O 3.273837 0.426282 -1.201793
O 1.300630 -0.082845 -2.355069
C 3.916645 1.138045 -2.264652
H 3.981837 2.207288 -2.046039
H 4.920619 0.714564 -2.341464
H 3.381435 1.005614 -3.208050
C 1.654535 -1.435404 -2.631937
H 2.732565 -1.592464 -2.530545
H 1.127248 -2.137309 -1.973062
H 1.352567 -1.625667 -3.663163
C 2.964522 0.398960 2.310863
H 2.862958 -0.540829 2.866298
H 3.957298 0.381465 1.840316
C 2.008716 2.698200 2.345983
H 2.694288 3.434648 1.903406
H 1.271613 3.268549 2.922990
C 2.765854 1.650507 3.215034
H 2.140095 1.377865 4.070796
H 3.708050 2.037011 3.610964

38
9d-D
C -0.403162 -0.251981 0.286834
C 1.131965 -0.265165 0.102868
C 0.231124 2.208158 0.171155
C -0.905108 1.199763 0.548830
H -0.874933 -0.651698 -0.613798
H -0.679635 -0.901736 1.121959
H -1.811525 1.393967 -0.027976
C 1.426227 0.438267 -1.256845
C 0.857879 1.882304 -1.162160
H -0.131939 3.239381 0.152959
H 1.528746 -1.283425 0.072228
C 1.220237 1.953518 1.339208
C 1.776036 0.552418 1.176844
C -1.110048 1.490068 2.045121
H -1.772254 2.351480 2.197482
H -1.525839 0.655773 2.616635
C 0.274331 1.886965 2.557472
O 0.578705 2.113708 3.707769
O 0.948665 2.671988 -2.079351
O 2.826850 0.441573 -1.410988
O 0.772518 -0.141168 -2.361890
C 3.346086 1.097979 -2.573706
H 3.379633 2.182208 -2.437329
H 4.357971 0.709304 -2.705396
H 2.740056 0.874643 -3.454620
C 1.102089 -1.505384 -2.614950
H 2.181243 -1.672533 -2.546460
H 0.587750 -2.181101 -1.920078
H 0.763451 -1.718694 -3.630168
C 3.185779 0.483808 1.679251
H 3.349220 -0.325346 2.403831
H 3.856831 0.289293 0.831734
C 2.463024 2.824984 1.555330
H 2.868912 3.117437 0.580133
H 2.242185 3.733172 2.122700
C 3.439856 1.883764 2.293083
H 3.184528 1.876717 3.355314
H 4.481617 2.200681 2.195654

38
9d-E
C -0.552165 -0.271943 0.142496
C 0.986873 -0.393877 0.153876
C 0.249628 2.129213 0.284506
C -0.870884 1.119207 0.738222
H -0.920999 -0.378975 -0.879742
H -1.023969 -1.051966 0.748001
H -1.851063 1.482018 0.420823
C 1.432395 0.338007 -1.137309
C 0.807421 1.772071 -1.102998

H -0.078795 3.170547 0.284593
H 1.332831 -1.429585 0.147833
C 1.448662 1.831071 1.122398
C 1.544535 0.358872 1.418020
C -0.798815 0.928256 2.260473
H -0.859268 1.873683 2.812745
H -1.613598 0.291174 2.618897
C 0.541439 0.261735 2.595839
O 0.815942 -0.187283 3.689267
O 0.914103 2.553834 -2.023687
O 2.841596 0.397190 -1.145137
O 0.934938 -0.250786 -2.316168
C 3.448307 1.089508 -2.241844
H 3.392355 2.173648 -2.108117
H 4.492479 0.770907 -2.251267
H 2.965812 0.828825 -3.186433
C 1.333331 -1.601212 -2.540357
H 2.393761 -1.745959 -2.312128
H 0.736058 -2.302379 -1.944209
H 1.158517 -1.800733 -3.599012
C 2.996339 0.124210 1.853870
H 3.052227 -0.612408 2.658556
H 3.578373 -0.225070 1.000891
C 2.729554 2.537335 1.408375
H 3.289504 2.712508 0.475427
H 2.596302 3.518842 1.879888
C 3.480203 1.521753 2.310523
H 3.188555 1.683082 3.353397
H 4.565944 1.632702 2.249879

38
9d-F
C -0.916773 0.127420 0.208513
C 0.595292 -0.249778 0.262118
C -0.176242 2.438272 0.855498
C -1.207907 1.373504 1.093796
H -1.207766 0.330808 -0.822278
H -1.526121 -0.708557 0.568316
H -2.220354 1.740976 0.908423
C 1.335917 0.341125 -0.963697
C 1.624972 1.801875 -0.534668
H -0.409530 3.420862 0.461865
H 0.742652 -1.330335 0.306777
C 1.233190 1.959874 0.952846
C 1.258155 0.491021 1.476766
C -1.042353 0.906677 2.551094
H -1.178144 1.725516 3.265957
H -1.760718 0.121977 2.816108
C 0.366131 0.356444 2.720967
O 0.742459 -0.135174 3.766466
O 2.104816 2.663085 -1.236897
O 2.551001 -0.371362 -1.092583
O 0.625725 0.368967 -2.172683
C 3.474918 0.135243 -2.063069
H 4.000876 1.019352 -1.691032
H 4.188933 -0.670776 -2.241329
H 2.964272 0.397753 -2.992923
C 0.315560 -0.917493 -2.708045
H 1.213667 -1.532489 -2.813392
H -0.408365 -1.452939 -2.080902
H -0.126596 -0.739219 -3.689239
C 2.738468 0.231681 1.834306
H 2.774313 -0.213640 2.829777
H 3.202774 -0.458271 1.132509
C 2.318654 2.685738 1.746527
H 2.631251 3.611148 1.256127
H 1.942384 2.931791 2.745371
C 3.439551 1.622900 1.809630
H 4.094696 1.769467 2.672002
H 4.064450 1.711802 0.914810

39
18d
C -0.848792 0.188635 0.136452
C 0.639721 -0.292137 0.109722
C -0.035330 2.507096 0.834189

C -1.047379 1.372639 1.105816
H -1.156677 0.473147 -0.871398
H -1.497664 -0.634710 0.451609
H -2.069854 1.753411 1.016964
C 1.330138 0.241766 -1.167627
C 1.675175 1.701089 -0.770119
H -0.287802 3.057805 -0.077239
H 0.720139 -1.379350 0.164166
C 1.389989 1.896647 0.725033
C 1.417228 0.427832 1.265633
C -0.786510 0.848126 2.515864
H -0.895849 1.625648 3.281039
H -1.472025 0.036911 2.787970
C 0.635677 0.317635 2.581943
O 1.104915 -0.136379 3.607369
O 2.128291 2.536979 -1.517869
O 2.506918 -0.515420 -1.364210
O 0.550913 0.285596 -2.333782
C 3.384201 -0.047908 -2.395278
H 3.953645 0.827621 -2.069219
H 4.066331 -0.874468 -2.601642
H 2.826013 0.213577 -3.297644
C 0.131786 -0.992528 -2.811362
H 0.980630 -1.673291 -2.921560
H -0.606913 -1.450375 -2.141460
H -0.329686 -0.818071 -3.784348
C 2.915496 0.126536 1.504645
H 3.017890 -0.300080 2.503450
H 3.300113 -0.591275 0.783528
C 2.562126 2.583830 1.418364
H 2.862561 3.500669 0.903963
H 2.278426 2.841794 2.445466
C 3.654915 1.492591 1.398729
H 4.387797 1.628231 2.198128
H 4.201714 1.552982 0.451677
H -0.065142 3.230357 1.657173

39
17d
C -0.562293 0.110547 0.389485
C 0.917138 -0.216384 0.100194
C 0.576548 2.368245 0.198101
C -0.752760 1.647418 0.575906
H -1.190140 -0.242004 -0.429514
H -0.878457 -0.421255 1.293113
H -1.586302 2.003516 -0.032302
C 1.272841 0.400359 -1.268405
C 1.141281 1.938202 -1.128466
H 0.454425 3.455377 0.186635
H 1.076556 -1.297612 0.048281
C 1.474220 1.927676 1.387509
C 1.756882 0.368695 1.259078
C -0.908291 2.046300 2.053508
H -1.353320 3.044123 2.154407
H -1.512579 1.358837 2.651581
C 0.529031 2.125730 2.574148
O 0.867718 2.300899 3.723608
O 1.489572 2.710061 -1.998750
O 2.570980 -0.020949 -1.601092
O 0.344787 0.073510 -2.288749
C 3.125059 0.478792 -2.824782
H 3.554175 1.474507 -2.687784
H 3.909035 -0.227961 -3.105422
H 2.365580 0.528124 -3.607720
C 0.234602 -1.316865 -2.585837
H 1.220251 -1.784355 -2.668418
H -0.352779 -1.850666 -1.828023
H -0.282727 -1.383579 -3.544446
C 3.301303 0.241825 1.205818
H 3.661055 -0.026645 2.205003
H 3.624282 -0.531766 0.508536
C 2.846103 2.591874 1.526958
H 2.855821 3.603583 1.109943
H 3.072776 2.668091 2.596501
C 3.827368 1.635843 0.834580

H 4.863016 1.794770 1.149538
H 3.790568 1.772848 -0.249846
H 1.398851 -0.136145 2.163016

38
9d-TS(C->D)
C -0.300585 -0.201656 0.434993
C 1.219566 -0.235156 0.157368
C 0.361168 2.249391 0.189200
C -0.807563 1.269852 0.542363
H -0.828575 -0.718169 -0.369799
H -0.505378 -0.740985 1.363898
H -1.633735 1.428112 -0.155657
C 1.449028 0.403663 -1.239654
C 0.922707 1.872010 -1.175235
H 0.028562 3.289330 0.175663
H 1.610844 -1.254891 0.166392
C 1.453302 1.957976 1.198168
C 1.920992 0.666901 1.132005
C -1.266044 1.609449 1.966505
H -1.820501 2.556602 1.972833
H -1.910178 0.834417 2.398737
C -0.033478 1.831504 2.829150
O 0.177635 1.491064 3.954157
O 0.991624 2.629690 -2.118876
O 2.837415 0.340916 -1.468540
O 0.708597 -0.189528 -2.282541
C 3.319979 0.962027 -2.664693
H 3.414537 2.044503 -2.542187
H 4.303060 0.524183 -2.850754
H 2.654857 0.762211 -3.508283
C 0.985791 -1.569055 -2.511461
H 2.063071 -1.759023 -2.533846
H 0.524547 -2.207845 -1.747470
H 0.551577 -1.811524 -3.482817
C 3.214272 0.498760 1.870929
H 3.229161 -0.361830 2.551267
H 4.025032 0.344162 1.144049
C 2.505460 2.863578 1.800388
H 3.098894 3.321590 0.996088
H 2.097332 3.671324 2.413869
C 3.353603 1.854382 2.618263
H 2.915731 1.759380 3.615358
H 4.393404 2.170397 2.732502

38
9d-TS(C->E)
C -0.392003 -0.323979 0.253479
C 1.148061 -0.387656 0.134298
C 0.341506 2.110925 0.244223
C -0.786889 1.106867 0.691782
H -0.829936 -0.574289 -0.715276
H -0.750212 -1.052357 0.985279
H -1.738838 1.403644 0.244363
C 1.477126 0.313219 -1.213731
C 0.857274 1.749740 -1.152315
H 0.006579 3.149288 0.259881
H 1.523070 -1.412736 0.132276
C 1.521639 1.806043 1.118542
C 1.801379 0.460812 1.214835
C -0.885530 1.123599 2.220480
H -1.034712 2.130074 2.633370
H -1.736793 0.508298 2.538500
C 0.358224 0.482596 2.830520
O 0.767397 0.594227 3.953854
O 0.873358 2.508441 -2.096657
O 2.882421 0.344937 -1.321947
O 0.880348 -0.285886 -2.339368
C 3.417747 1.053042 -2.444188
H 3.402404 2.134374 -2.278290
H 4.449695 0.711399 -2.546836
H 2.856056 0.830685 -3.354728
C 1.254298 -1.641112 -2.575019
H 2.333966 -1.779774 -2.464030
H 0.730741 -2.328401 -1.898349
H 0.960938 -1.865864 -3.601831

C 3.117925 0.241095 1.914616
H 3.078172 -0.569207 2.648950
H 3.871785 -0.018170 1.162609
C 2.500877 2.664302 1.857470
H 3.069659 3.284910 1.150314
H 2.022318 3.357048 2.561905
C 3.405845 1.618716 2.580781
H 3.128267 1.571641 3.634472
H 4.463279 1.887538 2.524578

38
9d-TS(D->E)
C -0.662673 -0.097277 0.147403
C 0.866025 -0.254495 0.241757
C 0.201368 2.298505 0.211443
C -1.007776 1.349687 0.569387
H -0.979577 -0.306919 -0.876979
H -1.173013 -0.804510 0.806469
H -1.927404 1.699169 0.096630
C 1.409634 0.417505 -1.053546
C 0.838203 1.870836 -1.101062
H -0.091815 3.348938 0.163274
H 1.178420 -1.299787 0.288756
C 1.230022 1.986088 1.288248
C 1.431187 0.547384 1.414262
C -1.057832 1.378119 2.094484
H -1.447375 2.326455 2.483270
H -1.668541 0.569510 2.507537
C 0.399322 1.270386 2.520081
O 0.786024 1.325970 3.719677
O 0.959557 2.583977 -2.072953
O 2.818217 0.438193 -0.967247
O 0.967770 -0.193647 -2.239560
C 3.512606 1.084966 -2.039897
H 3.473246 2.173897 -1.942922
H 4.547699 0.746074 -1.968511
H 3.089833 0.804529 -3.007311
C 1.339644 -1.561529 -2.396194
H 2.381874 -1.725610 -2.105905
H 0.691899 -2.225435 -1.810332
H 1.215757 -1.792793 -3.455416
C 2.796659 0.292088 2.027119
H 2.738237 -0.364212 2.899334
H 3.415540 -0.189927 1.267950
C 2.493541 2.734733 1.632504
H 2.977554 3.028096 0.692424
H 2.314443 3.647070 2.209019
C 3.351514 1.695468 2.404317
H 3.232277 1.855170 3.475358
H 4.412856 1.791943 2.159907

38
9d-TS(E->F)
C -0.578884 -0.349699 0.119702
C 0.952133 -0.408913 0.328220
C -0.100703 2.113797 0.437300
C -1.092819 0.989545 0.697338
H -0.808065 -0.424698 -0.942937
H -1.079098 -1.176315 0.634457
H -2.081396 1.237769 0.304952
C 1.609969 0.326913 -0.858700
C 1.383549 1.806402 -0.524953
H -0.433813 3.106541 0.159973
H 1.326439 -1.429418 0.421997
C 1.208275 1.902921 1.033153
C 1.341775 0.468302 1.575174
C -1.114642 0.794616 2.226796
H -1.342557 1.725209 2.760218
H -1.859146 0.053421 2.534557
C 0.268766 0.316826 2.668084
O 0.490777 -0.104744 3.784275
O 1.740435 2.779255 -1.228641
O 2.991141 0.020448 -0.809134
O 1.072835 0.053308 -2.127770
C 3.825731 0.732649 -1.728584
H 3.972997 1.769488 -1.413533

H 4.781180 0.204348 -1.731974
H 3.394233 0.733422 -2.733023
C 1.234946 -1.293401 -2.566460
H 2.276520 -1.617411 -2.486438
H 0.604578 -1.985105 -1.991918
H 0.921896 -1.314674 -3.611517
C 2.773371 0.397238 2.143923
H 2.711976 0.089653 3.190326
H 3.378465 -0.331373 1.605853
C 2.165852 2.784427 1.797966
H 2.418773 3.690125 1.241835
H 1.721088 3.078665 2.756793
C 3.364703 1.829090 2.002772
H 3.976259 2.112047 2.863424
H 4.004427 1.873365 1.116647

53
9d-TS(F->18d)
C -0.809957 -0.131440 0.141674
C 0.723042 -0.399451 0.269628
C -0.316632 2.288095 0.631155
C -1.233169 1.123761 0.959067
H -1.073734 0.000336 -0.908481
H -1.375021 -0.989932 0.519208
H -2.275018 1.371638 0.738071
C 1.452064 0.123041 -0.991576
C 1.583578 1.640727 -0.704283
H -0.573841 2.908966 -0.226792
H 0.940063 -1.457565 0.425666
C 1.147362 1.909336 0.755779
C 1.295979 0.503055 1.418849
C -1.048173 0.762729 2.436784
H -1.276338 1.603546 3.099697
H -1.688842 -0.074643 2.734153
C 0.401128 0.371663 2.661433
O 0.807172 -0.012368 3.739758
O 1.984688 2.472433 -1.483554
O 2.725929 -0.485328 -1.023192
O 0.779261 -0.026520 -2.213361
C 3.628922 0.005731 -2.021416
H 4.050098 0.974690 -1.737537
H 4.426158 -0.736201 -2.089706
H 3.128358 0.110218 -2.987210
C 0.576554 -1.381952 -2.614273
H 1.516070 -1.941547 -2.612856
H -0.142027 -1.892480 -1.960841
H 0.172184 -1.342228 -3.626571
C 2.788961 0.411544 1.811053
H 2.841511 0.101581 2.855884
H 3.318399 -0.324325 1.209337
C 2.171770 2.792353 1.472365
H 2.397647 3.698562 0.905825
H 1.782567 3.086164 2.452192
C 3.376903 1.838596 1.615628
H 4.034084 2.130181 2.438582
H 3.975859 1.877958 0.699476
H -0.564186 3.367468 1.686240
S -0.749804 4.458231 2.689946
C -2.508386 4.911525 2.262495
C -2.799221 6.190884 3.060878
H -3.830214 6.512299 2.872507
H -2.687859 6.022016 4.135856
H -2.127752 7.001927 2.766825
C -2.635650 5.187425 0.760187
H -2.448408 4.285099 0.170974
H -3.652121 5.528746 0.529003
H -1.930358 5.957995 0.440044
C -3.469344 3.796283 2.690116
H -3.376189 3.586070 3.758450
H -4.505156 4.093888 2.484756
H -3.274437 2.870640 2.142051

53
9d-TS(D->17d)
C -0.641236 0.155491 0.360997
C 0.845513 -0.208514 0.128553

C 0.534044 2.392620 0.237780
C -0.785649 1.675283 0.661213
H -1.209339 -0.096821 -0.535616
H -1.041667 -0.433039 1.187932
H -1.645894 2.082308 0.126495
C 1.239608 0.421862 -1.240002
C 1.034996 1.955360 -1.114463
H 0.427654 3.481251 0.239456
H 0.991027 -1.290962 0.082043
C 1.481783 1.920091 1.376016
C 1.689670 0.393317 1.239964
C -0.860213 1.992524 2.168696
H -1.379524 2.938129 2.362767
H -1.348982 1.216973 2.764690
C 0.591575 2.160927 2.604360
O 0.986054 2.457227 3.711273
O 1.297086 2.721282 -2.018779
O 2.581616 0.088740 -1.498143
O 0.394947 0.030454 -2.298506
C 3.159628 0.609891 -2.702574
H 3.482443 1.645799 -2.571521
H 4.022328 -0.025123 -2.913869
H 2.447830 0.565345 -3.529282
C 0.364639 -1.371300 -2.567009
H 1.371503 -1.798845 -2.556214
H -0.263498 -1.906568 -1.844546
H -0.068447 -1.481092 -3.562472
C 3.168598 0.095577 1.435785
H 3.326684 -0.797063 2.048985
H 3.616376 -0.094394 0.456316
C 2.892481 2.513923 1.478209
H 3.241827 2.779639 0.474089
H 2.905959 3.416624 2.094356
C 3.749751 1.377540 2.062720
H 3.634711 1.358014 3.149035
H 4.814190 1.501377 1.845625
S 0.469968 -1.457456 3.241779
H 1.100829 -0.331478 2.392546
C 0.906597 -1.032704 5.010564
C -0.152192 -0.099488 5.610681
H 0.065373 0.069978 6.672904
H -0.151610 0.871681 5.112351
H -1.149747 -0.540436 5.533209
C 2.294458 -0.391205 5.089558
H 2.572538 -0.253919 6.141623
H 3.051865 -1.021491 4.615715
H 2.294486 0.592374 4.617140
C 0.901550 -2.381583 5.746822
H 1.665101 -3.053977 5.347400
H 1.107725 -2.213502 6.810618
H -0.070727 -2.877319 5.667327

6. X-Ray Crystal Data

The sample of compound **5** for X-ray analysis was obtained via slow evaporation in ether. The thermal ellipsoids are shown at the 30% probability level.

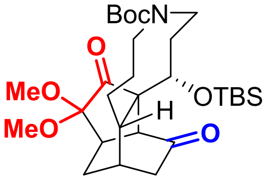

5

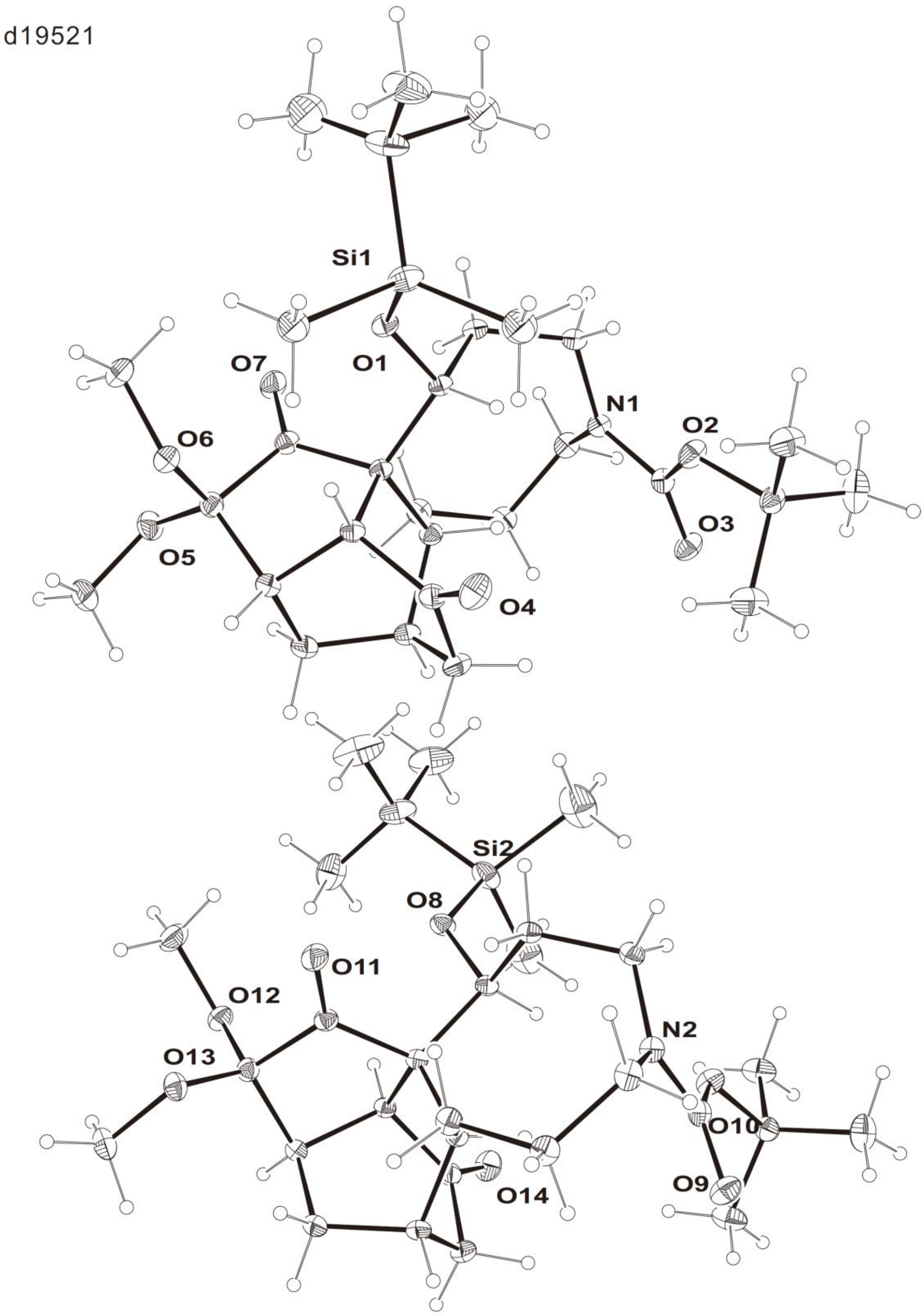

Table S2. Crystal data and structure refinement for compound **5** (CCDC 2217732).

Identification code	d19521	
Empirical formula	C29 H49 N O7 Si	
Formula weight	551.78	
Temperature	200(2) K	
Wavelength	0.71073 Å	
Crystal system	Triclinic	
Space group	P -1	
Unit cell dimensions	a = 10.0691(4) Å	α= 94.6330(10)°.
	b = 15.0308(6) Å	β= 98.2990(10)°.
	c = 20.8925(8) Å	γ = 97.4360(10)°.
Volume	3086.6(2) $Å^3$	
Z	4	
Density (calculated)	1.187 Mg/m^3	
Absorption coefficient	0.119 mm^{-1}	
F(000)	1200	
Crystal size	0.52 x 0.47 x 0.22 mm^3	
Theta range for data collection	2.15 to 25.11°.	
Index ranges	-11<=h<=12, -17<=k<=17, -24<=l<=24	
Reflections collected	111397	
Independent reflections	10880 [R(int) = 0.0321]	
Completeness to theta = 25.11°	99.1 %	
Absorption correction	multi-scan	
Max. and min. transmission	0.9742 and 0.9405	
Refinement method	Full-matrix least-squares on F^2	
Data / restraints / parameters	10880 / 0 / 693	
Goodness-of-fit on F^2	1.015	
Final R indices [I>2sigma(I)]	R1 = 0.0536, wR2 = 0.1310	
R indices (all data)	R1 = 0.0625, wR2 = 0.1406	
Largest diff. peak and hole	0.859 and -0.777 e.$Å^{-3}$	

The sample of compound **8b** for X-ray analysis was obtained via slow evaporation in ether. The thermal ellipsoids are shown at the 30% probability level.

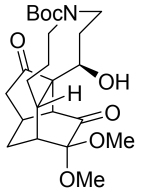

8b

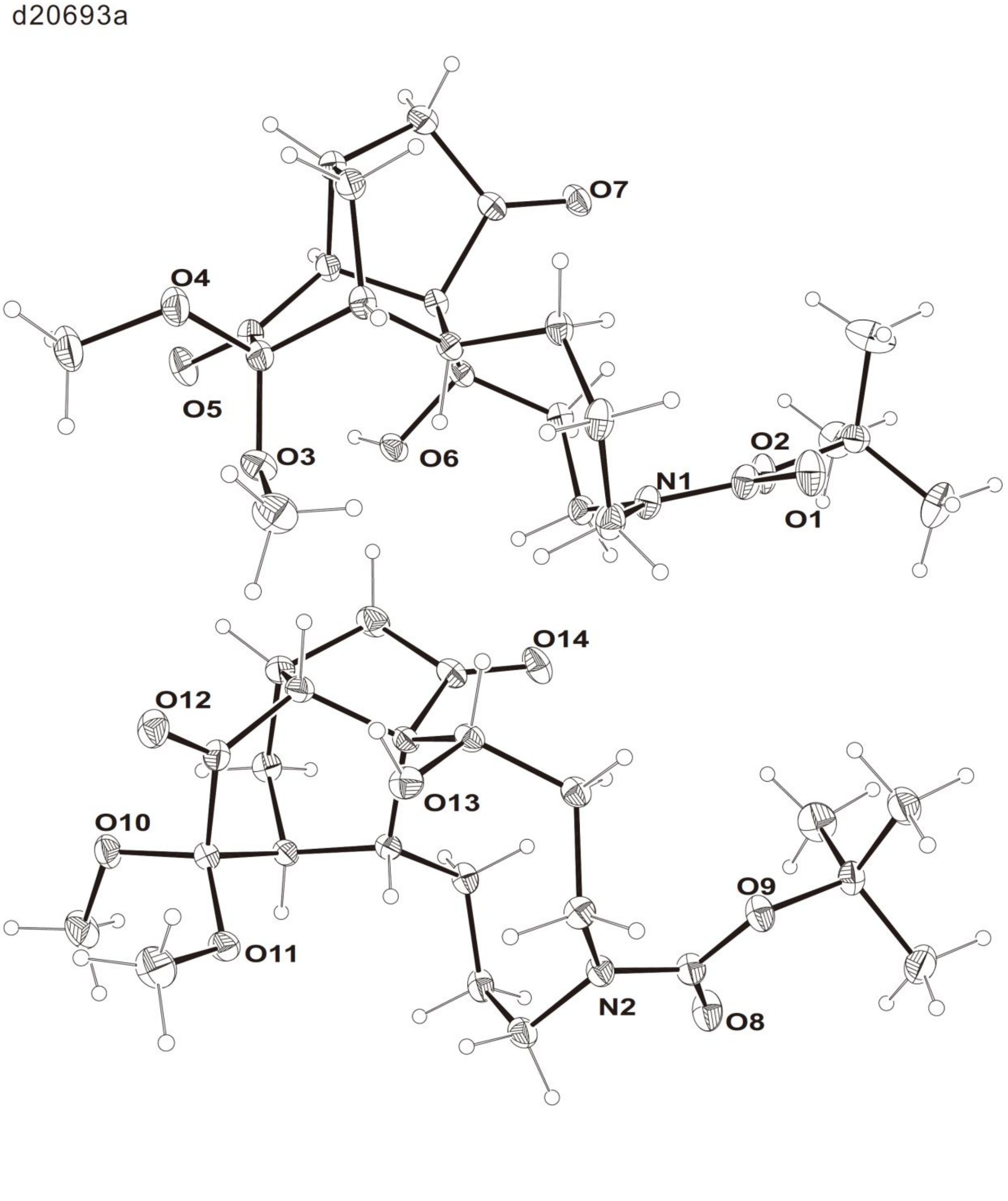

Table S3. Crystal data and structure refinement for compound **8b** (CCDC 2217733).

Identification code	d20693a	
Empirical formula	C23 H35 N O7	
Formula weight	437.52	
Temperature	200(2) K	
Wavelength	0.71073 Å	
Crystal system	Monoclinic	
Space group	P 21/n	
Unit cell dimensions	a = 16.2584(5) Å	α= 90°.
	b = 14.7339(6) Å	β= 92.8220(10)°.
	c = 19.5315(8) Å	γ = 90°.
Volume	4673.1(3) $Å^3$	
Z	8	
Density (calculated)	1.244 Mg/m^3	
Absorption coefficient	0.091 mm^{-1}	
F(000)	1888	
Crystal size	0.17 x 0.09 x 0.04 mm^3	
Theta range for data collection	2.50 to 25.05°.	
Index ranges	-19<=h<=18, -17<=k<=17, -23<=l<=23	
Reflections collected	63997	
Independent reflections	8239 [R(int) = 0.0526]	
Completeness to theta = 25.05°	99.4 %	
Absorption correction	multi-scan	
Max. and min. transmission	0.9964 and 0.9846	
Refinement method	Full-matrix least-squares on F^2	
Data / restraints / parameters	8239 / 0 / 573	
Goodness-of-fit on F^2	1.088	
Final R indices [I>2sigma(I)]	R1 = 0.0561, wR2 = 0.1607	
R indices (all data)	R1 = 0.0815, wR2 = 0.1764	
Largest diff. peak and hole	0.289 and -0.291 e.$Å^{-3}$	

The sample of compound **18c** for X-ray analysis was obtained via slow evaporation in dichloromethane. The thermal ellipsoids are shown at the 30% probability level.

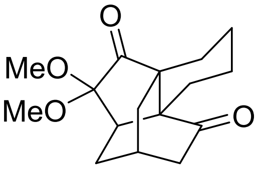

18c

d23302

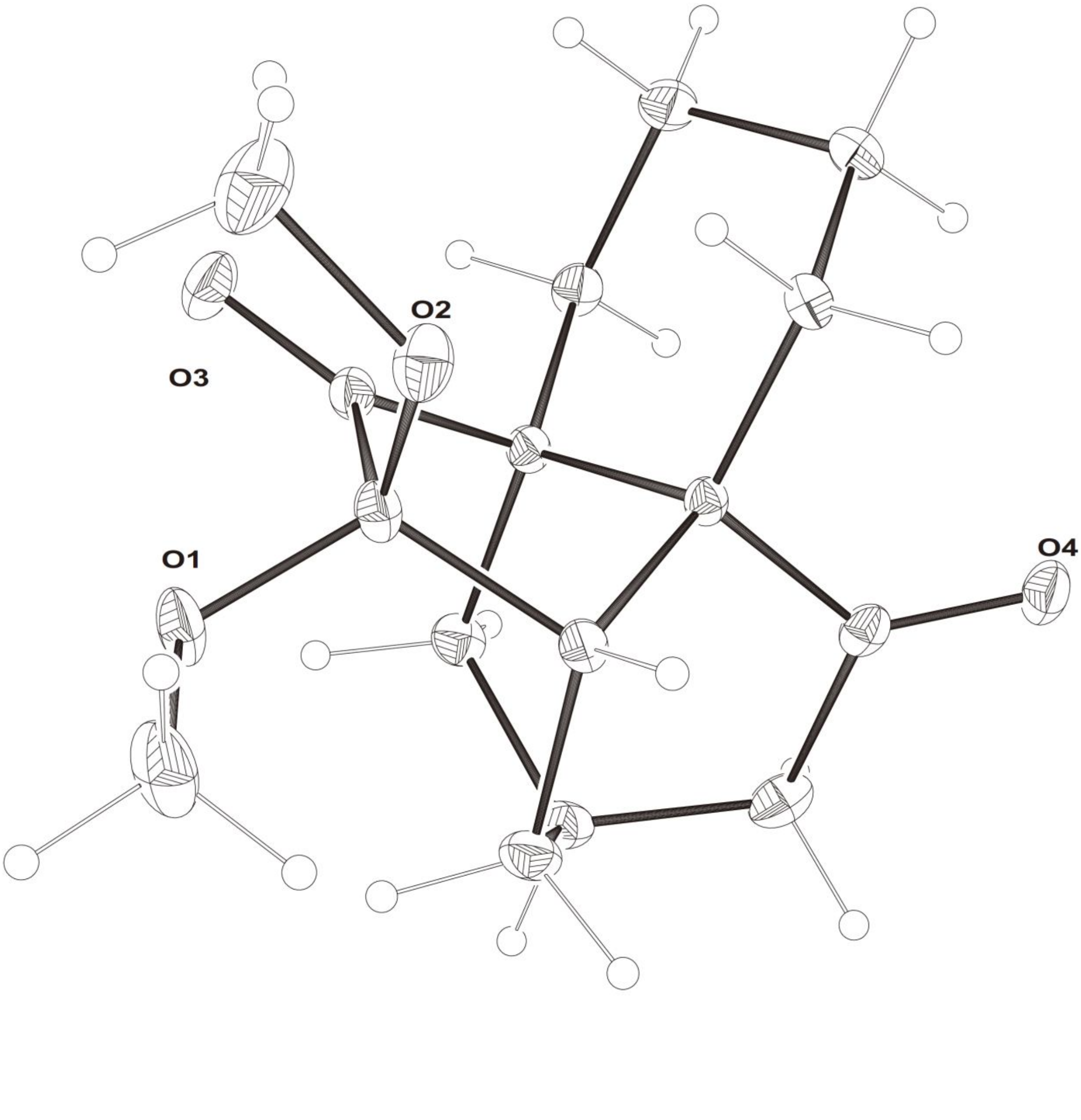

Table S4. Crystal data and structure refinement for compound **18c** (CCDC 2217734).

Identification code	d23302	
Empirical formula	C16 H22 O4	
Formula weight	278.34	
Temperature	200(2) K	
Wavelength	0.71073 Å	
Crystal system	Monoclinic	
Space group	C 2/c	
Unit cell dimensions	a = 22.1369(7) Å	α= 90°.
	b = 11.1599(3) Å	β= 117.9700(10)°.
	c = 12.9852(4) Å	γ = 90°.
Volume	2833.23(15) Å^3	
Z	8	
Density (calculated)	1.305 Mg/m^3	
Absorption coefficient	0.093 mm^{-1}	
F(000)	1200	
Crystal size	0.28 x 0.24 x 0.11 mm^3	
Theta range for data collection	2.08 to 25.10°.	
Index ranges	-25<=h<=26, -13<=k<=13, -15<=l<=15	
Reflections collected	23052	
Independent reflections	2504 [R(int) = 0.0496]	
Completeness to theta = 25.10°	99.0 %	
Absorption correction	multi-scan	
Max. and min. transmission	0.9899 and 0.9746	
Refinement method	Full-matrix least-squares on F^2	
Data / restraints / parameters	2504 / 0 / 183	
Goodness-of-fit on F^2	1.010	
Final R indices [I>2sigma(I)]	R1 = 0.0370, wR2 = 0.1005	
R indices (all data)	R1 = 0.0413, wR2 = 0.1048	
Largest diff. peak and hole	0.268 and -0.164 e.Å^{-3}	

7. ^{1}H- and ^{13}C-NMR Spectra

```
Data acquired by:064030

EXP-19-AA4923-P

exp1  PROTON

      SAMPLE              PRESATURATION
date   Oct  3 2019  satmode            n
solvent      cdcl3  wet                n
file /home/NHRI/vn~        SPECIAL
mrsys/data/064030/~ temp        not used
EXP-19-AA4923-P_20~ gain        not used
190922_01/PROTON_0~ spin              20
                 2  hst            0.008
   ACQUISITION      pw90          10.700
sw          4800.8  alfa          10.000
at           1.706          FLAGS
np           16384  il                 n
fb            2600  in                 n
bs               4  dp                 y
ss               4  hs                nn
d1           1.000        PROCESSING
nt              16  lb              0.20
ct              16  fn             32768
   TRANSMITTER              DISPLAY
tn              H1  sp            -146.5
sfrq       299.993  wp            3299.6
tof          258.1  rfl           2777.8
tpwr            57  rfp           2180.9
pw           5.350  rp            -101.5
    DECOUPLER       lp             -68.3
dn             C13           PLOT
dof              0  wc               268
dm             nnn  sc                 0
decwave          g  vs                18
dpwr            38  th                 3
dmf          12300  ai   cdc  ph
```

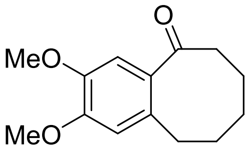

12a

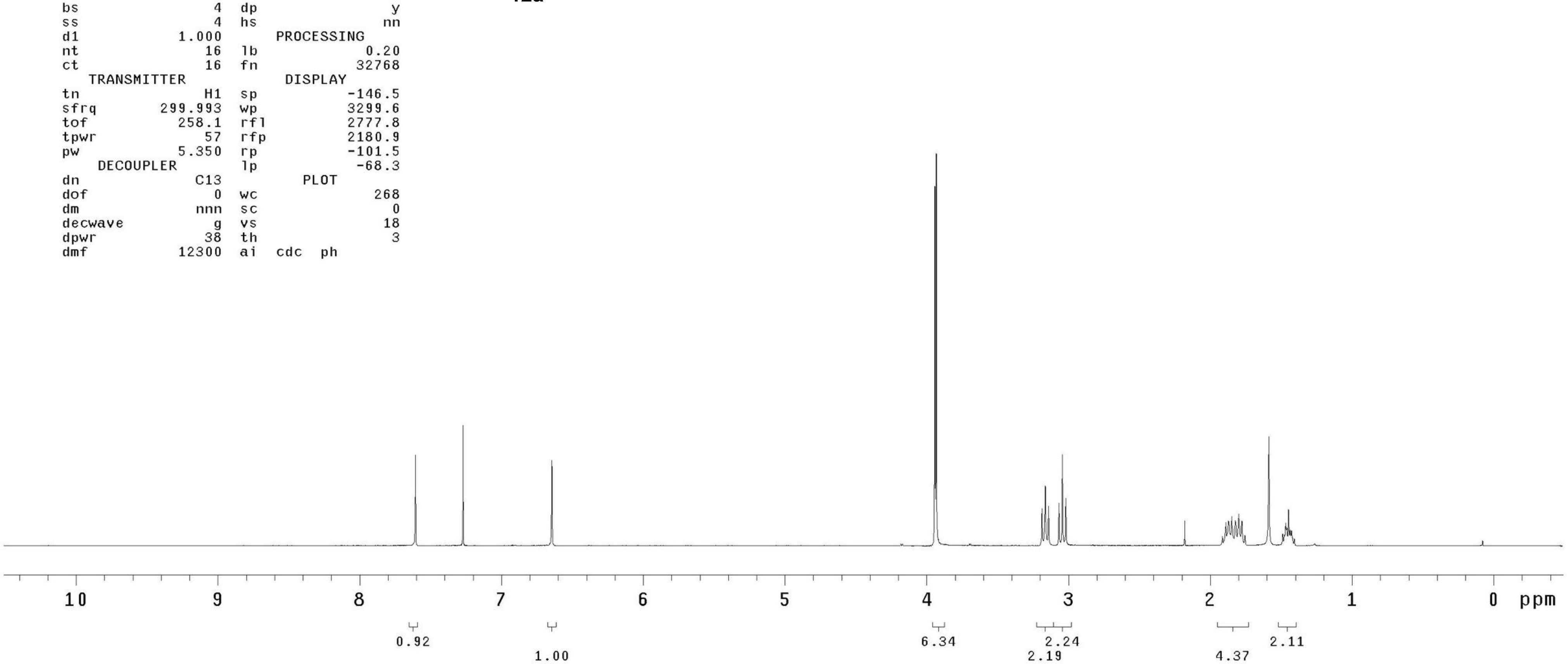

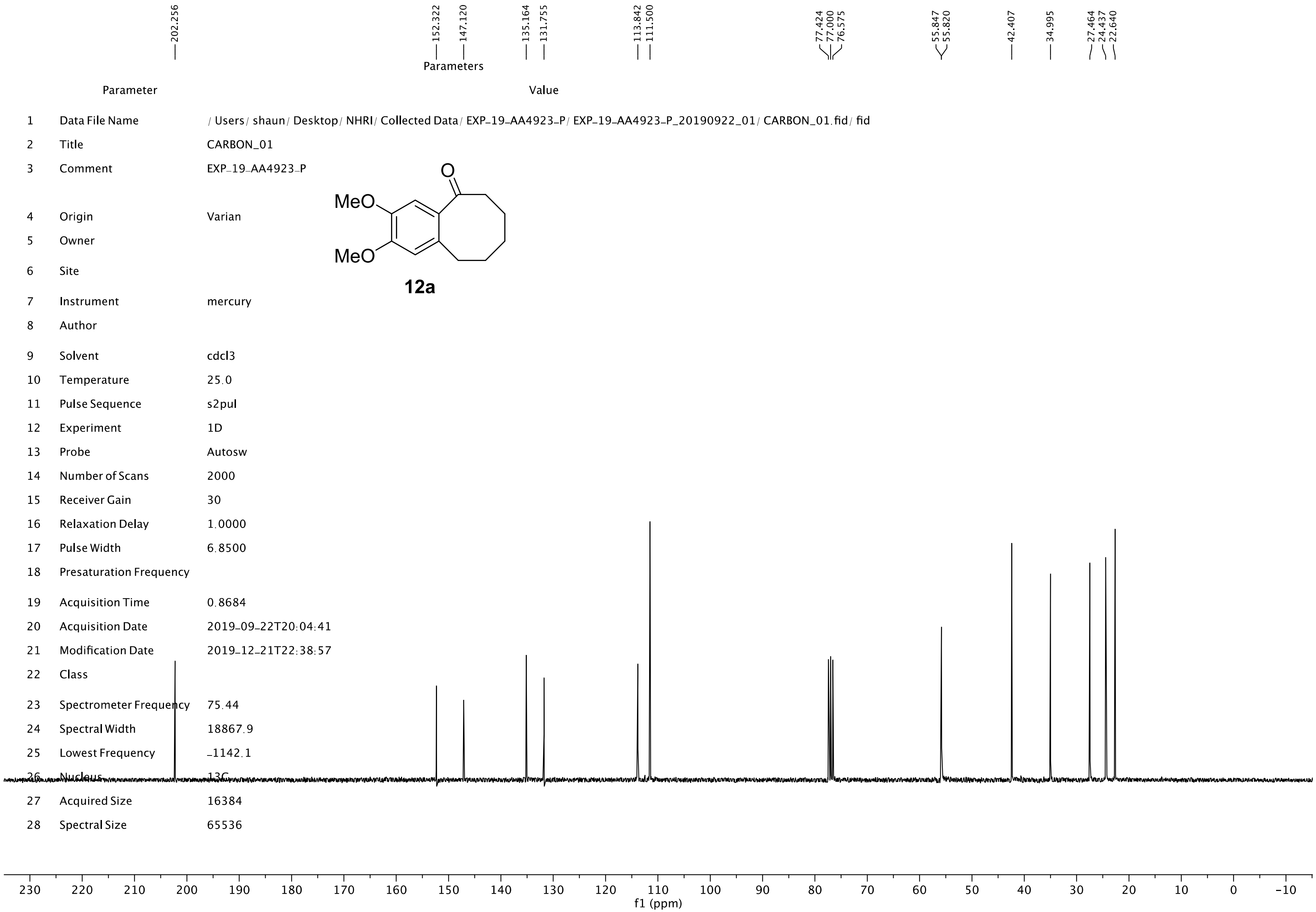

Parameters

	Parameter	Value
1	Data File Name	/ Users / shaun / Desktop / NHRI / Collected Data / EXP_19_AA4923_P / EXP_19_AA4923_P_20190922_01 / CARBON_01.fid / fid
2	Title	CARBON_01
3	Comment	EXP_19_AA4923_P
4	Origin	Varian
5	Owner	
6	Site	
7	Instrument	mercury
8	Author	
9	Solvent	cdcl3
10	Temperature	25.0
11	Pulse Sequence	s2pul
12	Experiment	1D
13	Probe	Autosw
14	Number of Scans	2000
15	Receiver Gain	30
16	Relaxation Delay	1.0000
17	Pulse Width	6.8500
18	Presaturation Frequency	
19	Acquisition Time	0.8684
20	Acquisition Date	2019-09-22T20:04:41
21	Modification Date	2019-12-21T22:38:57
22	Class	
23	Spectrometer Frequency	75.44
24	Spectral Width	18867.9
25	Lowest Frequency	-1142.1
26	Nucleus	13C
27	Acquired Size	16384
28	Spectral Size	65536

12a

Data acquired by:064030

EXP-19-AA5634-A

exp1 PROTON

SAMPLE		PRESATURATION	
date	Oct 7 2019	satmode	n
solvent	cdcl3	wet	n
file /home/NHRI/vn~		SPECIAL	
mrsys/data/064030/~		temp	not used
EXP-19-AA5634-A_20~		gain	not used
191004_01/PROTON_0~		spin	20
	3	hst	0.008
ACQUISITION		pw90	10.700
sw	4800.8	alfa	10.000
at	1.706	FLAGS	
np	16384	il	n
fb	2600	in	n
bs	4	dp	y
ss	4	hs	nn
d1	1.000	PROCESSING	
nt	16	lb	0.20
ct	16	fn	32768
TRANSMITTER		DISPLAY	
tn	H1	sp	-150.0
sfrq	299.993	wp	3299.6
tof	258.1	rfl	2778.1
tpwr	57	rfp	2180.9
pw	5.350	rp	-105.4
DECOUPLER		lp	-64.6
dn	C13	PLOT	
dof	0	wc	268
dm	nnn	sc	0
decwave	g	vs	35
dpwr	38	th	4
dmf	12300	ai	cdc ph

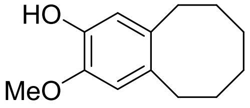

13a

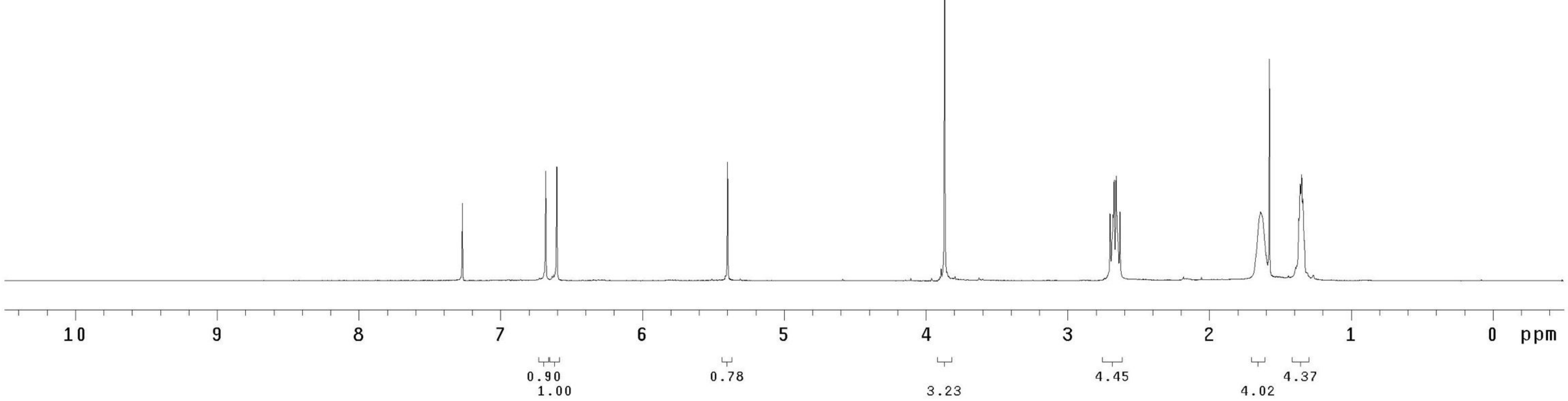

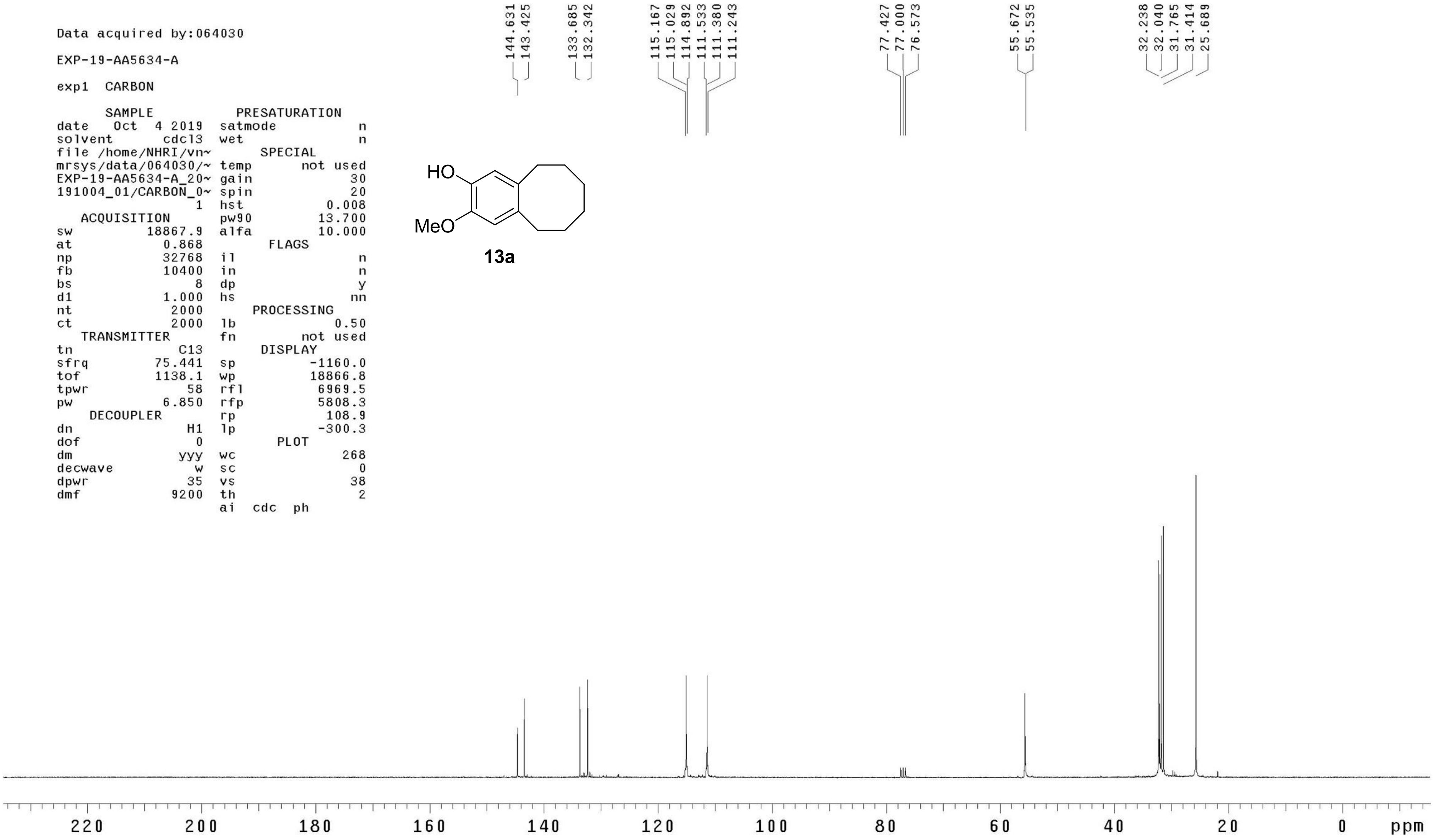

Data acquired by:064030
EXP-19-AA5634-A
exp1 CARBON
SAMPLE
date Oct 4 2019
solvent cdcl3
file /home/NHRI/vn~
mrsys/data/064030/~
EXP-19-AA5634-A_20~
191004_01/CARBON_0~
1
ACQUISITION
sw 18867.9
at 0.868
np 32768
fb 10400
bs 8
d1 1.000
nt 2000
ct 2000
TRANSMITTER
tn C13
sfrq 75.441
tof 1138.1
tpwr 58
pw 6.850
DECOUPLER
dn H1
dof 0
dm yyy
decwave w
dpwr 35
dmf 9200
PRESATURATION
satmode n
wet n
SPECIAL
temp not used
gain 30
spin 20
hst 0.008
pw90 13.700
alfa 10.000
FLAGS
il n
in n
dp y
hs nn
PROCESSING
lb 0.50
fn not used
DISPLAY
sp -1160.0
wp 18866.8
rfl 6969.5
rfp 5808.3
rp 108.9
lp -300.3
PLOT
wc 268
sc 0
vs 38
th 2
ai cdc ph
144.631
143.425
133.685
132.342
115.167
115.029
114.892
111.533
111.380
111.243
77.427
77.000
76.573
55.672
55.535
32.238
32.040
31.765
31.414
25.689
HO
MeO
13a
220
200
180
160
140
120
100
80
60
40
20
0
ppm

064030-EXP-22-AB0558-A2

Parameters

	Parameter	Value
1	Sample Name	0I+OSIyxSMa9k6gOve1XeA.3.fid
2	Origin	Bruker BioSpin GmbH
3	Owner	nmrsu
4	Site	
5	Spectrometer	Avance
6	Author	
7	Solvent	CDCl3
8	Temperature	294.7
9	Pulse Sequence	zg30
10	Experiment	1D
11	Number of Scans	32
12	Receiver Gain	101
13	Relaxation Delay	1.0000
14	Pulse Width	10.6000
15	Acquisition Time	2.7525
16	Acquisition Date	2022-10-04T21:33:05
17	Modification Date	2022-10-07T09:02:02
18	Spectrometer Frequency	600.14
19	Spectral Width	11904.8
20	Lowest Frequency	-2254.4
21	Nucleus	1H
22	Acquired Size	32768
23	Spectral Size	131072

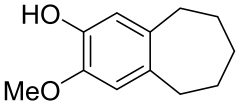

12b

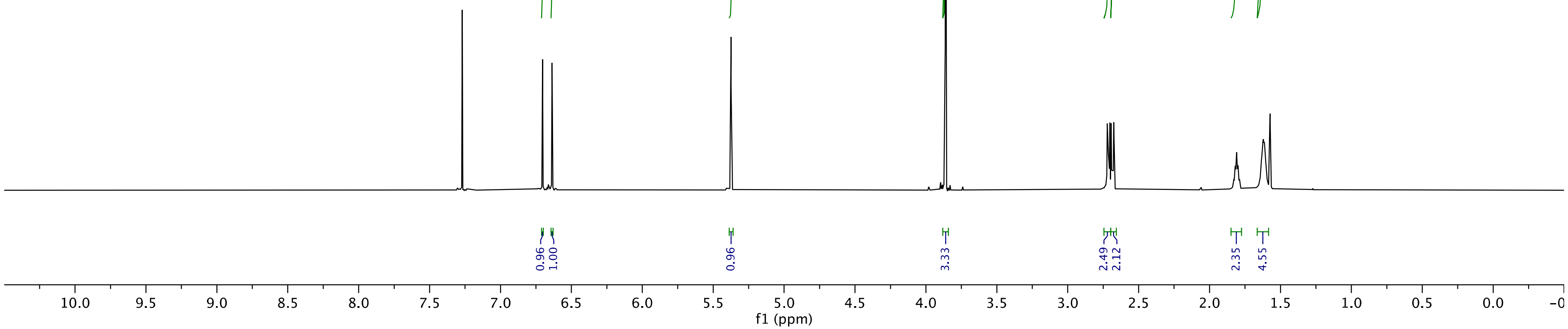

064030-EXP-22-AB0558-A2

Parameters

	Parameter	Value
1	Sample Name	0I+OSIyxSMa9k6gOve1XeA.2.fid
2	Origin	Bruker BioSpin GmbH
3	Owner	nmrsu
4	Site	
5	Spectrometer	Avance
6	Author	
7	Solvent	CDCl3
8	Temperature	294.7
9	Pulse Sequence	zgpg30
10	Experiment	1D
11	Number of Scans	1600
12	Receiver Gain	90
13	Relaxation Delay	2.0000
14	Pulse Width	11.5000
15	Acquisition Time	0.9175
16	Acquisition Date	2022-10-04T21:25:51
17	Modification Date	2022-10-07T09:02:02
18	Spectrometer Frequency	150.92
19	Spectral Width	35714.3
20	Lowest Frequency	-1268.1
21	Nucleus	13C
22	Acquired Size	32768
23	Spectral Size	65536

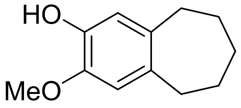

12b

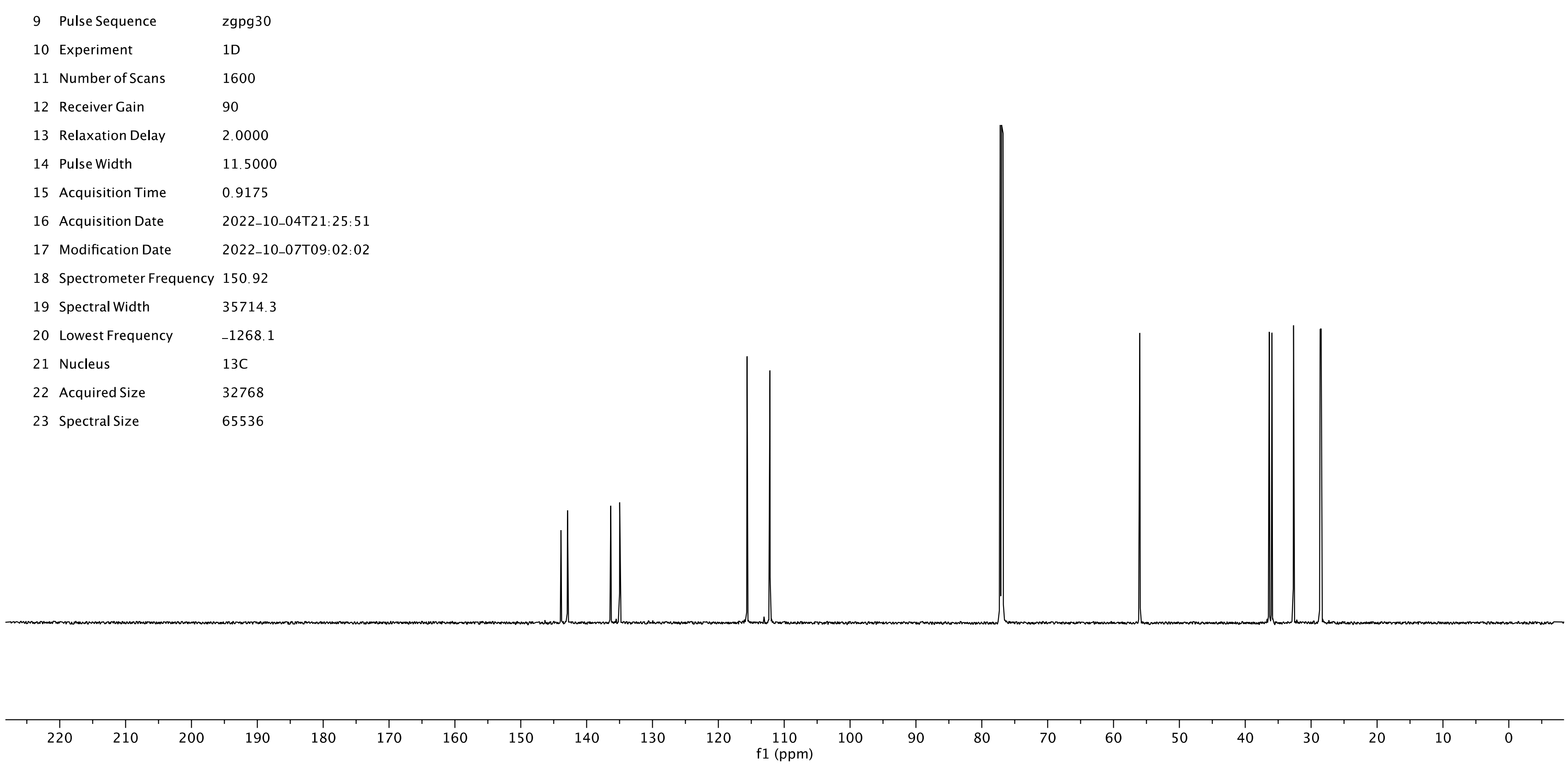

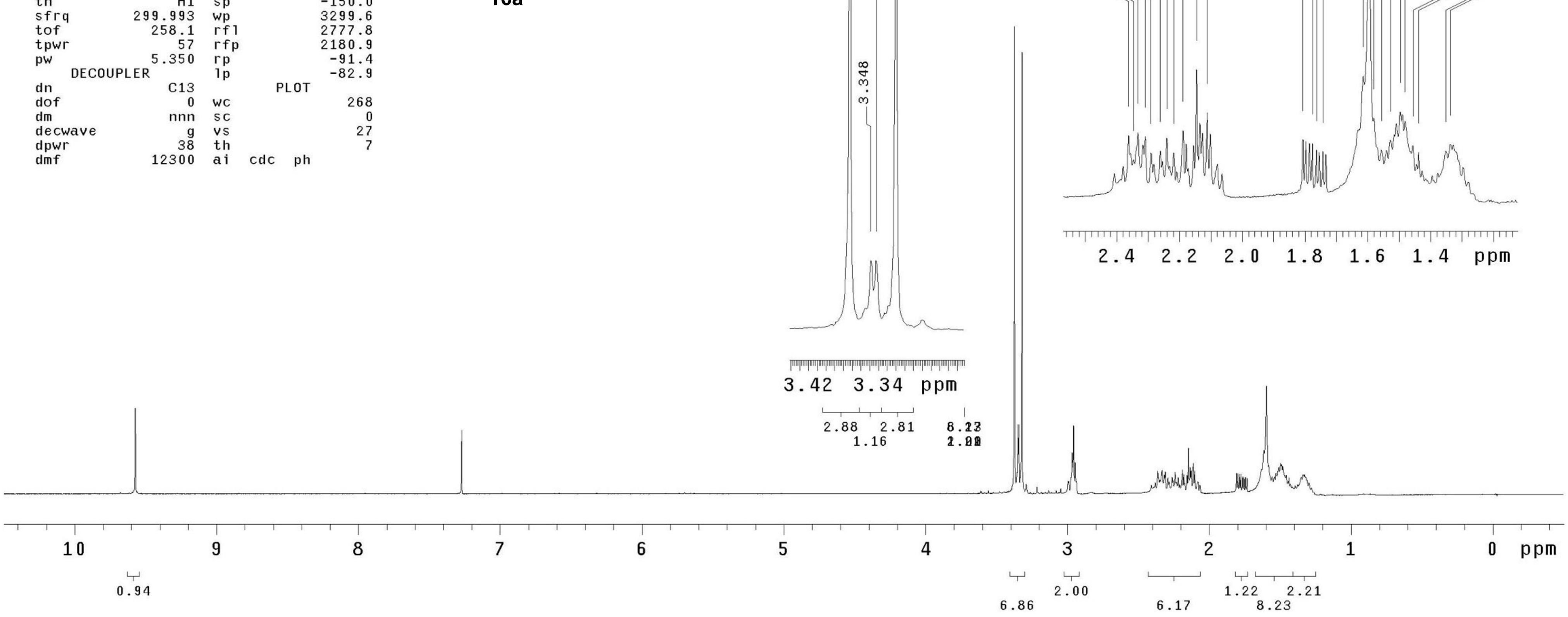
EXP-19-AA5643-A
exp6 PROTON
SAMPLE
date Oct 14 2019
solvent cdcl3
file /home/NHRI/vn~
mrsys/data/064030/~
EXP-19-AA5643-A_20~
191012_01/PROTON_0~
2
ACQUISITION
sw 4800.8
at 1.706
np 16384
fb 2600
bs 4
ss 4
d1 1.000
nt 16
ct 16
TRANSMITTER
tn H1
sfrq 299.993
tof 258.1
tpwr 57
pw 5.350
DECOUPLER
dn C13
dof 0
dm nnn
decwave g
dpwr 38
dmf 12300
PRESATURATION
satmode n
wet n
SPECIAL
temp not used
gain not used
spin 20
hst 0.008
pw90 10.700
alfa 10.000
FLAGS
il n
in n
dp y
hs nn
PROCESSING
lb 0.20
fn 32768
DISPLAY
sp -150.0
wp 3299.6
rfl 2777.8
rfp 2180.9
rp -91.4
lp -82.9
PLOT
wc 268
sc 0
vs 27
th 7
ai cdc ph
9.572
7.270
3.373
3.348
3.342
3.319
2.964
2.956
2.145
1.614
1.595
2.362
2.346
2.333
2.309
2.291
2.261
2.240
2.217
2.188
2.112
1.806
1.776
1.763
1.743
1.580
1.556
1.528
1.496
1.481
1.455
1.437
1.350
1.336
2.4 2.2 2.0 1.8 1.6 1.4 ppm
3.42 3.34 ppm
2.88 2.81
1.16
10 9 8 7 6 5 4 3 2 1 0 ppm
0.94
6.86
2.00
6.17
1.22 2.21
8.23

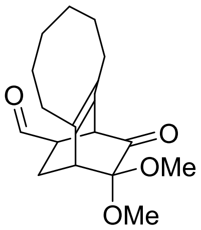
O
O
OMe
OMe

16a

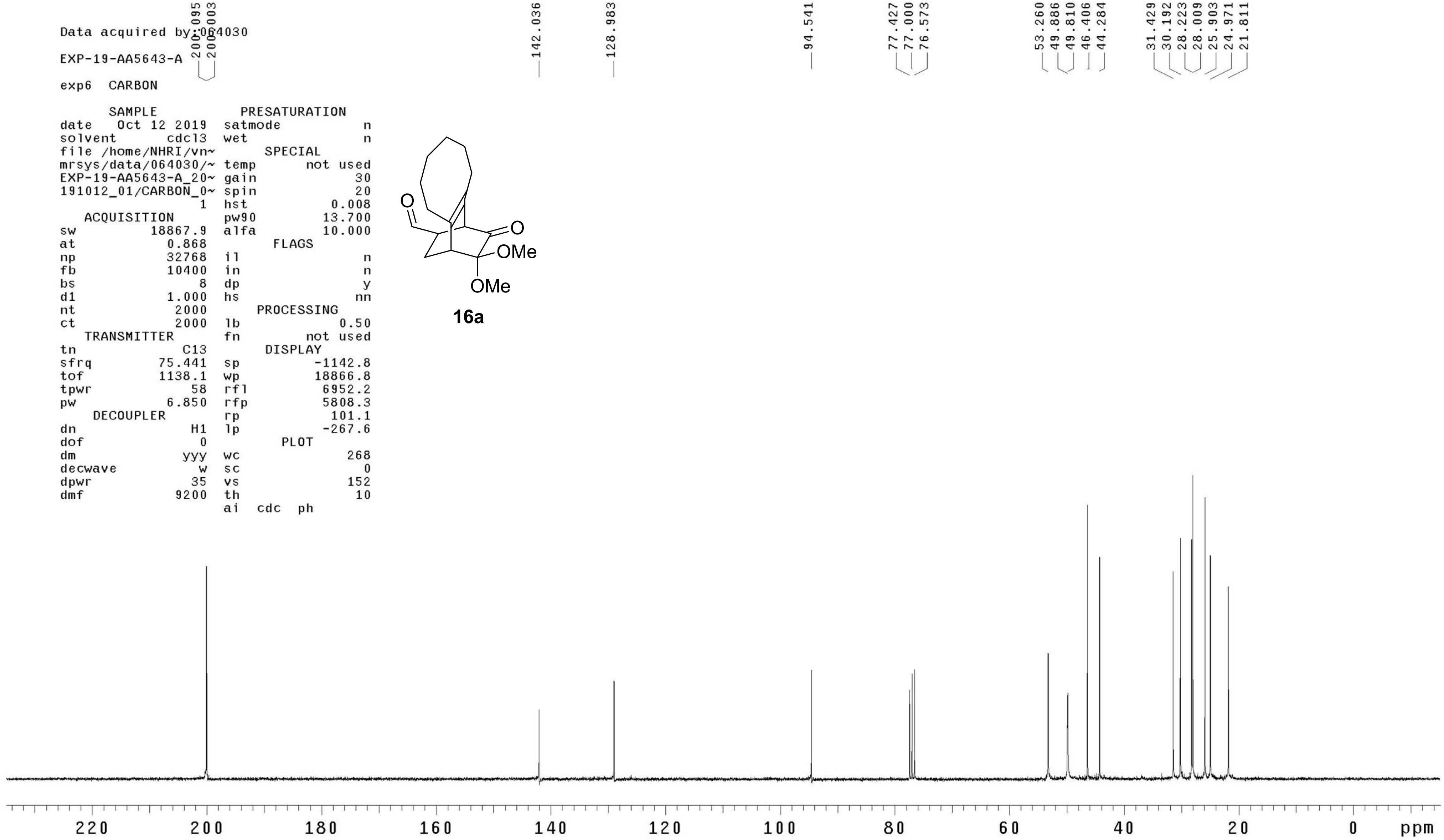

Data acquired by:064030
EXP-19-AA5643-A
exp6 CARBON
SAMPLE
date Oct 12 2019
solvent cdcl3
file /home/NHRI/vn~
mrsys/data/064030/~
EXP-19-AA5643-A_20~
191012_01/CARBON_0~
1
ACQUISITION
sw 18867.9
at 0.868
np 32768
fb 10400
bs 8
d1 1.000
nt 2000
ct 2000
TRANSMITTER
tn C13
sfrq 75.441
tof 1138.1
tpwr 58
pw 6.850
DECOUPLER
dn H1
dof 0
dm yyy
decwave w
dpwr 35
dmf 9200
PRESATURATION
satmode n
wet n
SPECIAL
temp not used
gain 30
spin 20
hst 0.008
pw90 13.700
alfa 10.000
FLAGS
il n
in n
dp y
hs nn
PROCESSING
lb 0.50
fn not used
DISPLAY
sp -1142.8
wp 18866.8
rfl 6952.2
rfp 5808.3
rp 101.1
lp -267.6
PLOT
wc 268
sc 0
vs 152
th 10
ai cdc ph
200.095
200.003
142.036
128.983
94.541
77.427
77.000
76.573
53.260
49.886
49.810
46.406
44.284
31.429
30.192
28.223
28.009
25.903
24.971
21.811
O
O
OMe
OMe
16a
220
200
180
160
140
120
100
80
60
40
20
0
ppm

064030-EXP-22-AB2950-P1

Parameters

	Parameter	Value
1	Sample Name	o6RkeaiJSvKFCwyk4pCa7w.1.fid
2	Origin	Bruker BioSpin GmbH
3	Owner	nmrsu
4	Site	
5	Spectrometer	Avance
6	Author	
7	Solvent	CDCl3
8	Temperature	294.7
9	Pulse Sequence	zg30
10	Experiment	1D
11	Number of Scans	32
12	Receiver Gain	101
13	Relaxation Delay	1.0000
14	Pulse Width	8.0000
15	Acquisition Time	4.1943
16	Acquisition Date	2022-10-08T13:14:29
17	Modification Date	2022-10-08T22:01:16
18	Spectrometer Frequency	400.17
19	Spectral Width	7812.5
20	Lowest Frequency	-1440.8
21	Nucleus	1H
22	Acquired Size	32768
23	Spectral Size	65536

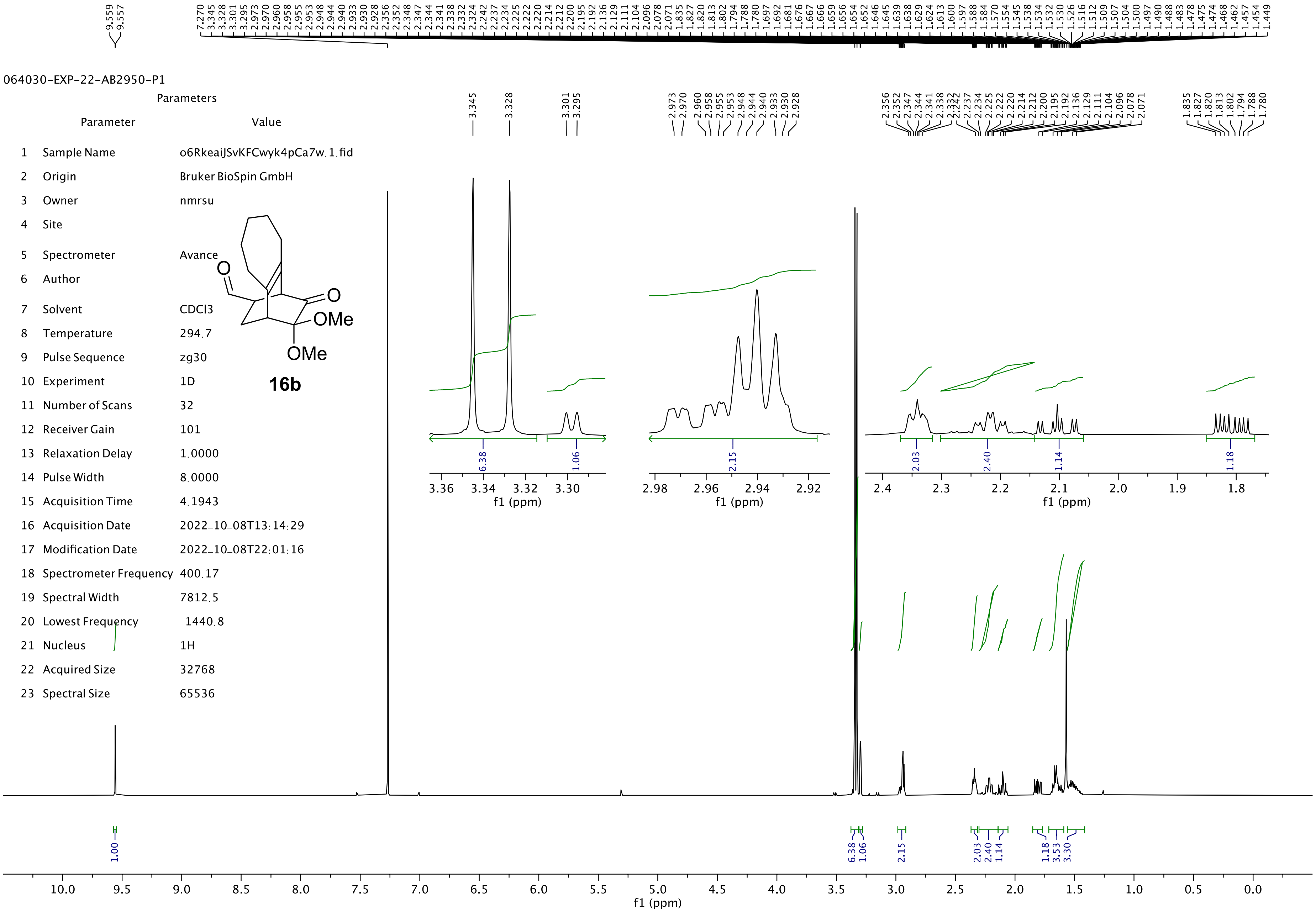

16b

064030-EXP-22-AB2950-P1

Parameters

	Parameter	Value
1	Sample Name	o6RkeaiJSvKFCwyk4pCa7w.3.fid
2	Origin	Bruker BioSpin GmbH
3	Owner	nmrsu
4	Site	
5	Spectrometer	Avance
6	Author	
7	Solvent	CDCl3
8	Temperature	294.7
9	Pulse Sequence	zgpg30
10	Experiment	1D
11	Number of Scans	1600
12	Receiver Gain	101
13	Relaxation Delay	2.0000
14	Pulse Width	8.0000
15	Acquisition Time	1.3763
16	Acquisition Date	2022-10-08T14:54:01
17	Modification Date	2022-10-08T22:01:16
18	Spectrometer Frequency	100.63
19	Spectral Width	23809.5
20	Lowest Frequency	-844.6
21	Nucleus	13C
22	Acquired Size	32768
23	Spectral Size	65536

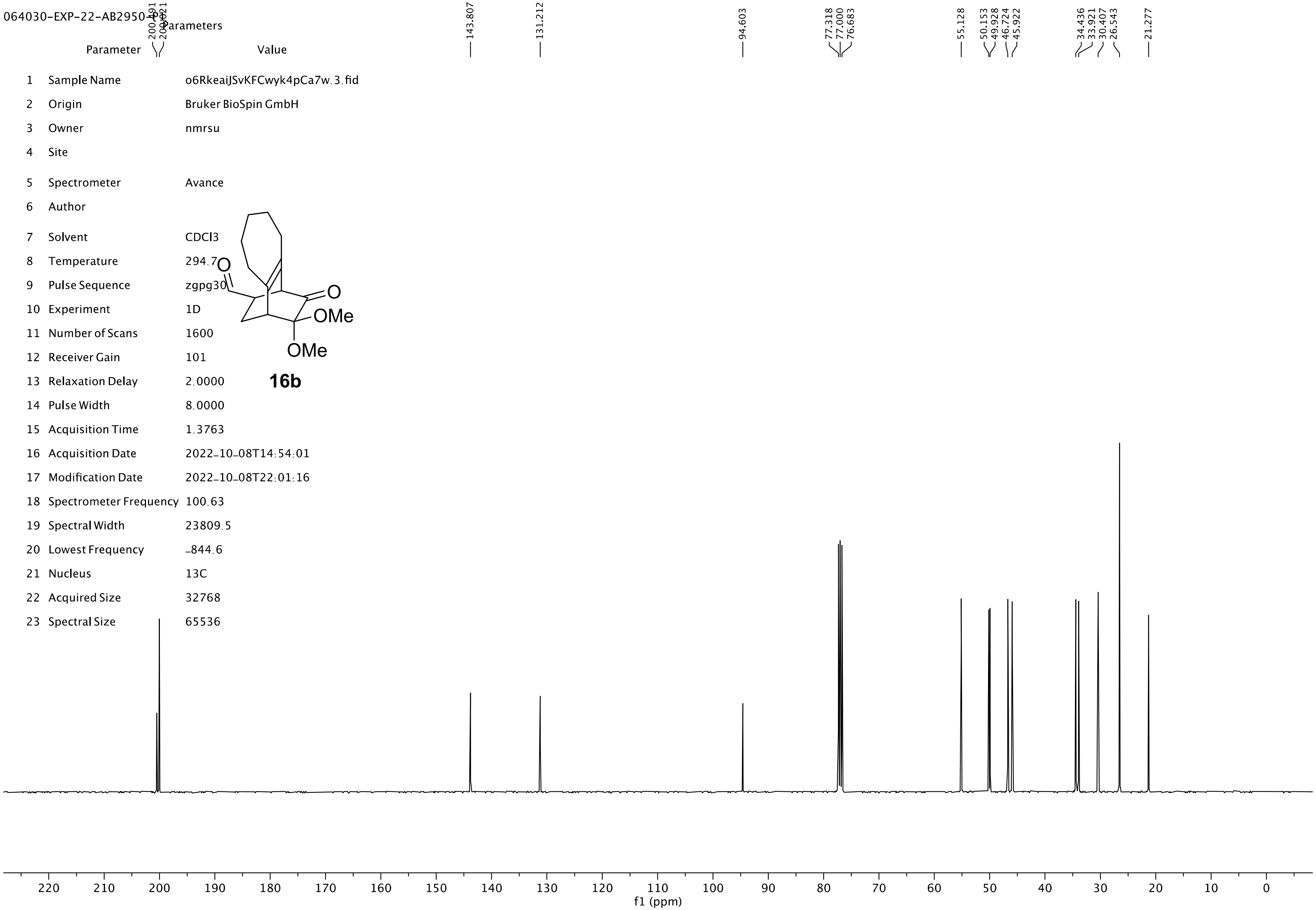

16b

```
EXP-20-AA6402-A

exp2  PROTON

      SAMPLE                 PRESATURATION
date   Jan 21 2020  satmode              n
solvent      cdcl3  wet                  n
file /home/NHRI/vn~       SPECIAL
mrsys/data/064030/~ temp          not used
EXP-20-AA6402-A_20~ gain          not used
200109_01/PROTON_0~ spin          not used
                 7  hst              0.008
   ACQUISITION      pw90            10.700
sw          4800.8  alfa            10.000
at           1.706          FLAGS
np           16384  il                   n
fb            2600  in                   n
bs               4  dp                   y
ss               4  hs                  nn
d1           1.000         PROCESSING
nt              16  lb                0.20
ct              16  fn               32768
   TRANSMITTER              DISPLAY
tn              H1  sp              -150.0
sfrq       299.993  wp              3299.6
tof          258.1  rfl             2777.8
tpwr            57  rfp             2180.9
pw           5.350  rp              -102.4
    DECOUPLER       lp               -71.6
dn             C13           PLOT
dof              0  wc                 268
dm             nnn  sc                   0
decwave          g  vs                  36
dpwr            38  th                   8
dmf          12300  ai   cdc   ph
```

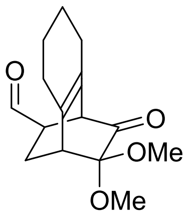

16c

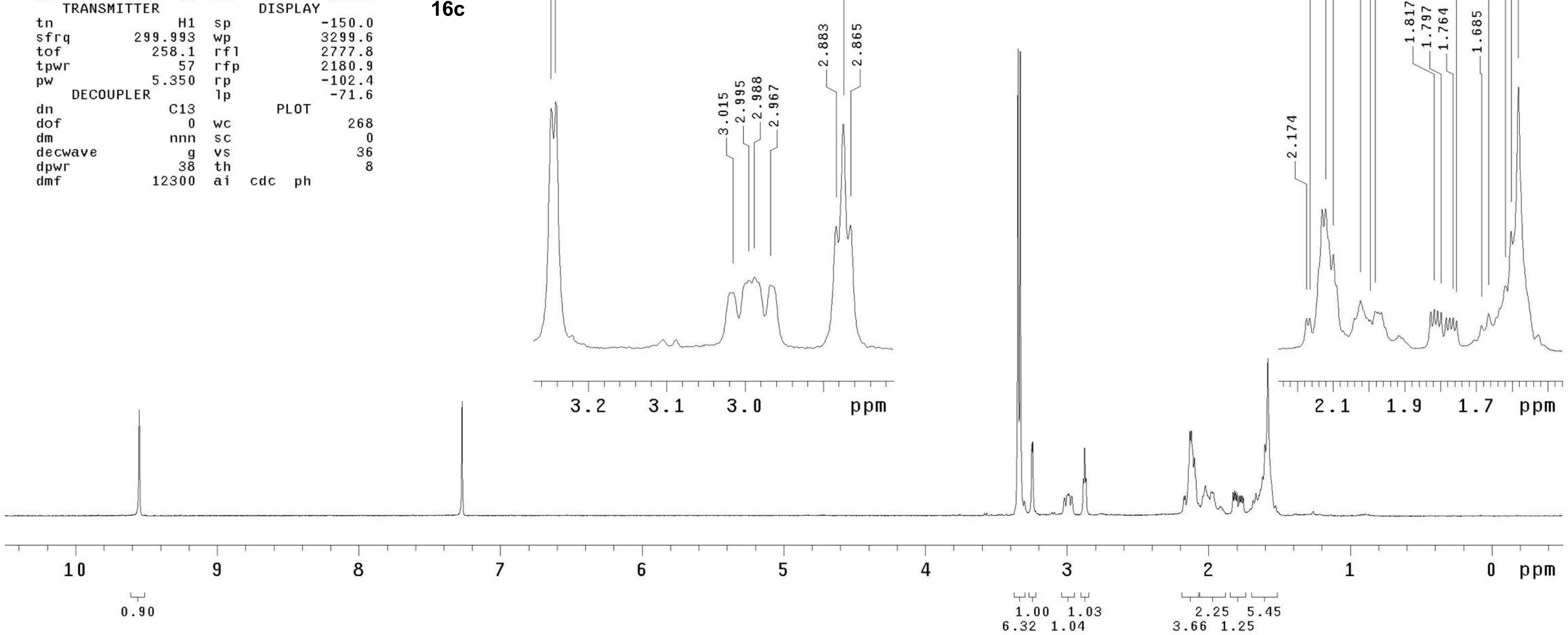

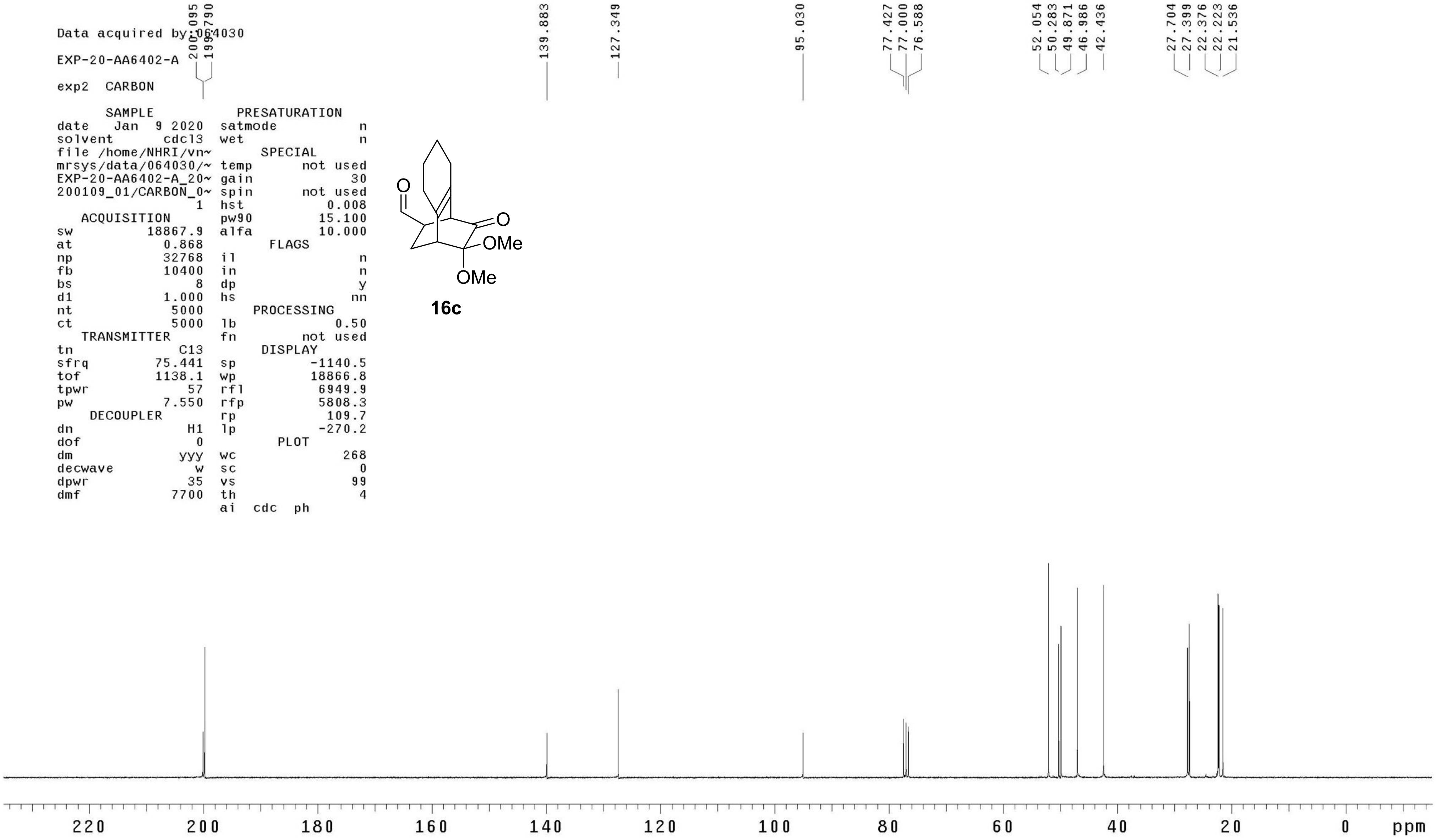

Data acquired by:064030
EXP-20-AA6402-A
exp2 CARBON
SAMPLE
date Jan 9 2020
solvent cdcl3
file /home/NHRI/vn~
mrsys/data/064030/~
EXP-20-AA6402-A_20~
200109_01/CARBON_0~
1
ACQUISITION
sw 18867.9
at 0.868
np 32768
fb 10400
bs 8
d1 1.000
nt 5000
ct 5000
TRANSMITTER
tn C13
sfrq 75.441
tof 1138.1
tpwr 57
pw 7.550
DECOUPLER
dn H1
dof 0
dm yyy
decwave w
dpwr 35
dmf 7700
PRESATURATION
satmode n
wet n
SPECIAL
temp not used
gain 30
spin not used
hst 0.008
pw90 15.100
alfa 10.000
FLAGS
il n
in n
dp y
hs nn
PROCESSING
lb 0.50
fn not used
DISPLAY
sp -1140.5
wp 18866.8
rfl 6949.9
rfp 5808.3
rp 109.7
lp -270.2
PLOT
wc 268
sc 0
vs 99
th 4
ai cdc ph
200.095
199.790
139.883
127.349
95.030
77.427
77.000
76.588
52.054
50.283
49.871
46.986
42.436
27.704
27.399
22.376
22.223
21.536
O
O
OMe
OMe
16c
220
200
180
160
140
120
100
80
60
40
20
0
ppm

064030-EXP-22-AB0550-P

Parameters

	Parameter	Value
1	Sample Name	jd5dysNiQ8eYPPI962mQPA.4.fid
2	Origin	Bruker BioSpin GmbH
3	Owner	nmrsu
4	Site	
5	Spectrometer	Avance
6	Author	
7	Solvent	CDCl3
8	Temperature	294.7
9	Pulse Sequence	zg30
10	Experiment	1D
11	Number of Scans	32
12	Receiver Gain	101
13	Relaxation Delay	1.0000
14	Pulse Width	10.6000
15	Acquisition Time	2.7525
16	Acquisition Date	2022-09-21T16:35:52
17	Modification Date	2022-10-07T08:48:20
18	Spectrometer Frequency	600.14
19	Spectral Width	11904.8
20	Lowest Frequency	-2254.6
21	Nucleus	1H
22	Acquired Size	32768
23	Spectral Size	131072

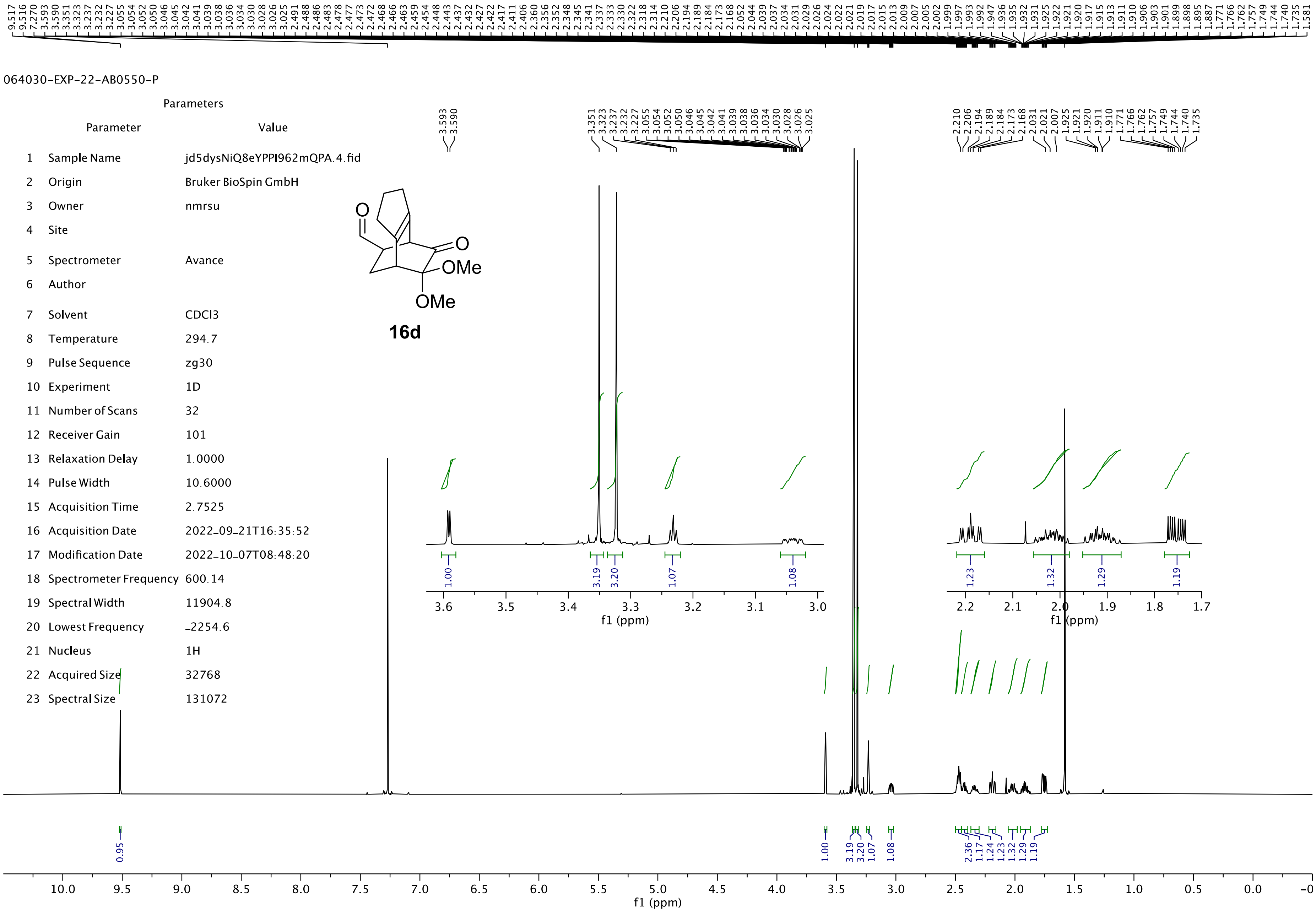

064030-EXP-22-AB0550-P

Parameters

	Parameter	Value
1	Sample Name	jd5dysNiQ8eYPPI962mQPA.2.fid
2	Origin	Bruker BioSpin GmbH
3	Owner	nmrsu
4	Site	
5	Spectrometer	Avance
6	Author	
7	Solvent	CDCl3
8	Temperature	294.7
9	Pulse Sequence	zgpg30
10	Experiment	1D
11	Number of Scans	1200
12	Receiver Gain	101
13	Relaxation Delay	2.0000
14	Pulse Width	11.5000
15	Acquisition Time	0.9175
16	Acquisition Date	2022-09-21T16:11:12
17	Modification Date	2022-10-07T08:48:19
18	Spectrometer Frequency	150.92
19	Spectral Width	35714.3
20	Lowest Frequency	-1277.4
21	Nucleus	13C
22	Acquired Size	32768
23	Spectral Size	65536

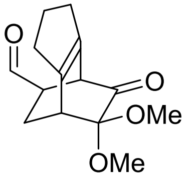

16d

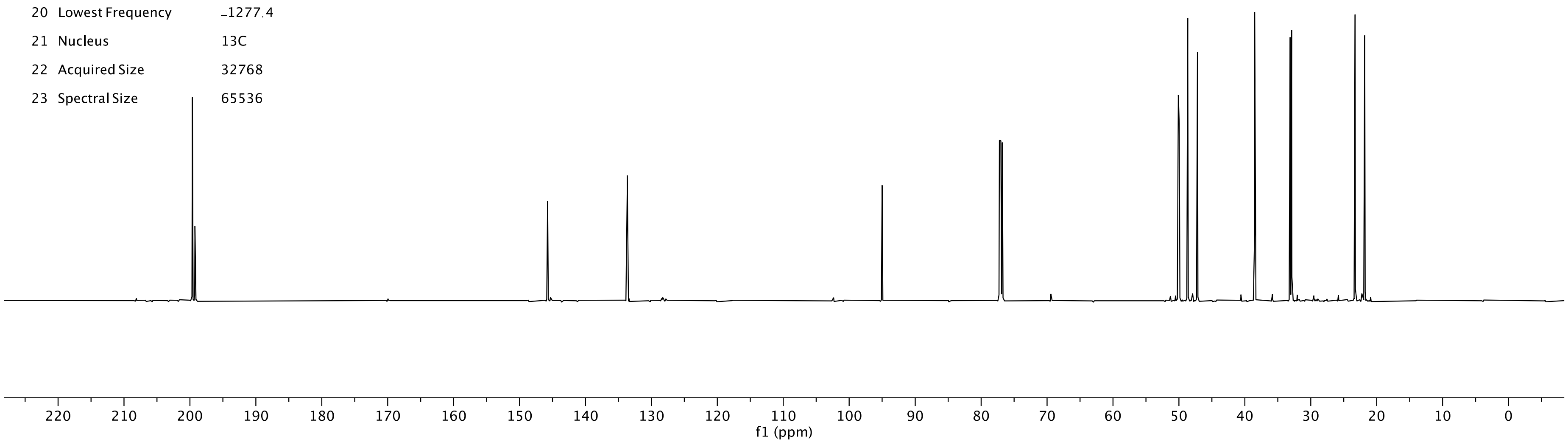

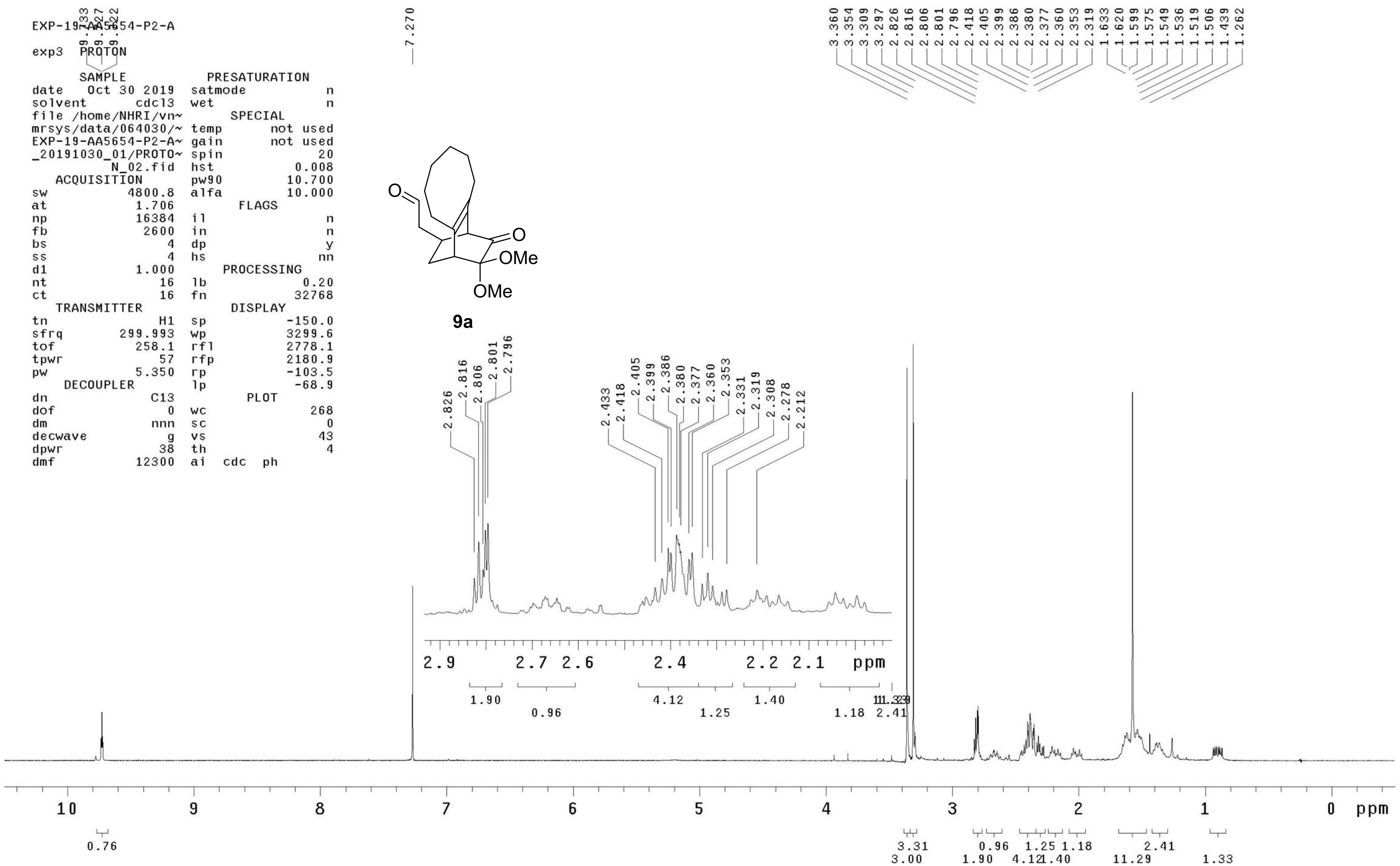

EXP-19-AA5654-P2-A
exp3 PROTON
SAMPLE
date Oct 30 2019
solvent cdcl3
file /home/NHRI/vn~
mrsys/data/064030/~
EXP-19-AA5654-P2-A~
_20191030_01/PROTO~
N_02.fid
ACQUISITION
sw 4800.8
at 1.706
np 16384
fb 2600
bs 4
ss 4
d1 1.000
nt 16
ct 16
TRANSMITTER
tn H1
sfrq 299.993
tof 258.1
tpwr 57
pw 5.350
DECOUPLER
dn C13
dof 0
dm nnn
decwave g
dpwr 38
dmf 12300
PRESATURATION
satmode n
wet n
SPECIAL
temp not used
gain not used
spin 20
hst 0.008
pw90 10.700
alfa 10.000
FLAGS
il n
in n
dp y
hs nn
PROCESSING
lb 0.20
fn 32768
DISPLAY
sp -150.0
wp 3299.6
rfl 2778.1
rfp 2180.9
rp -103.5
lp -68.9
PLOT
wc 268
sc 0
vs 43
th 4
ai cdc ph
9.733 9.727 9.722
7.270
3.360 3.354 3.309 3.297 2.826 2.816 2.806 2.801 2.796 2.418 2.405 2.399 2.386 2.380 2.377 2.360 2.353 2.319 1.633 1.620 1.599 1.575 1.549 1.536 1.519 1.506 1.439 1.262
2.826 2.816 2.806 2.801 2.796
2.433 2.418 2.405 2.399 2.386 2.380 2.377 2.360 2.353 2.331 2.319 2.308 2.278 2.212
O
O
OMe
OMe
9a
2.9 2.7 2.6 2.4 2.2 2.1 ppm
1.90 0.96 4.12 1.25 1.40 1.18 2.41 11.29
10 9 8 7 6 5 4 3 2 1 0 ppm
0.76 3.31 3.00 1.90 0.96 4.12 1.25 1.40 1.18 11.29 2.41 1.33

Data acquired by: 064030

EXP-19-AA5654-P2-A

exp1 CARBON

```
      SAMPLE              PRESATURATION
date   Oct 30 2019   satmode             n
solvent      cdcl3   wet                 n
file /home/NHRI/vn~        SPECIAL
mrsys/data/064030/~  temp         not used
EXP-19-AA5654-P2-A~  gain               30
_20191030_01/CARBO~  spin               20
              N_01   hst             0.008
   ACQUISITION       pw90           13.700
sw         18867.9   alfa           10.000
at           0.868          FLAGS
np           32768   il                  n
fb           10400   in                  n
bs               8   dp                  y
d1           1.000   hs                 nn
nt            2000        PROCESSING
ct            2000   lb               0.50
   TRANSMITTER       fn           not used
tn             C13         DISPLAY
sfrq        75.441   sp            -1142.8
tof         1138.1   wp            18866.8
tpwr            58   rfl            6952.2
pw           6.850   rfp            5808.3
   DECOUPLER         rp              115.9
dn              H1   lp             -294.6
dof              0          PLOT
dm             yyy   wc                268
decwave          w   sc                  0
dpwr            35   vs                154
dmf           9200   th                 10
                     ai   cdc   ph
```

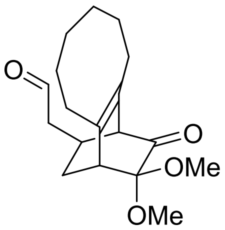

9a

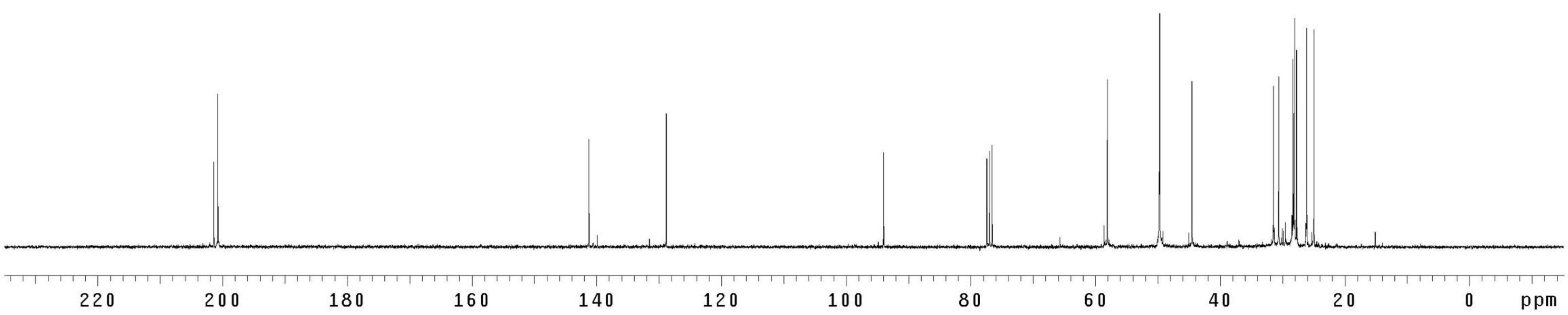

064030-EXP-22-AB2951-P

Parameters

	Parameter	Value
1	Sample Name	SqxPrsWwQAWR0A8tgmyzBg.2.fid
2	Origin	Bruker BioSpin GmbH
3	Owner	nmrsu
4	Site	
5	Spectrometer	Avance
6	Author	
7	Solvent	CDCl3
8	Temperature	294.7
9	Pulse Sequence	zg30
10	Experiment	1D
11	Number of Scans	16
12	Receiver Gain	30
13	Relaxation Delay	1.0000
14	Pulse Width	8.0000
15	Acquisition Time	4.1943
16	Acquisition Date	2022-10-12T04:34:50
17	Modification Date	2022-10-12T09:39:39
18	Spectrometer Frequency	400.17
19	Spectral Width	7812.5
20	Lowest Frequency	-1440.8
21	Nucleus	1H
22	Acquired Size	32768
23	Spectral Size	65536

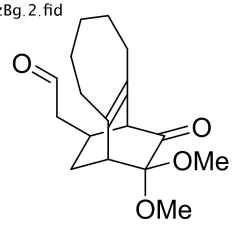

9b

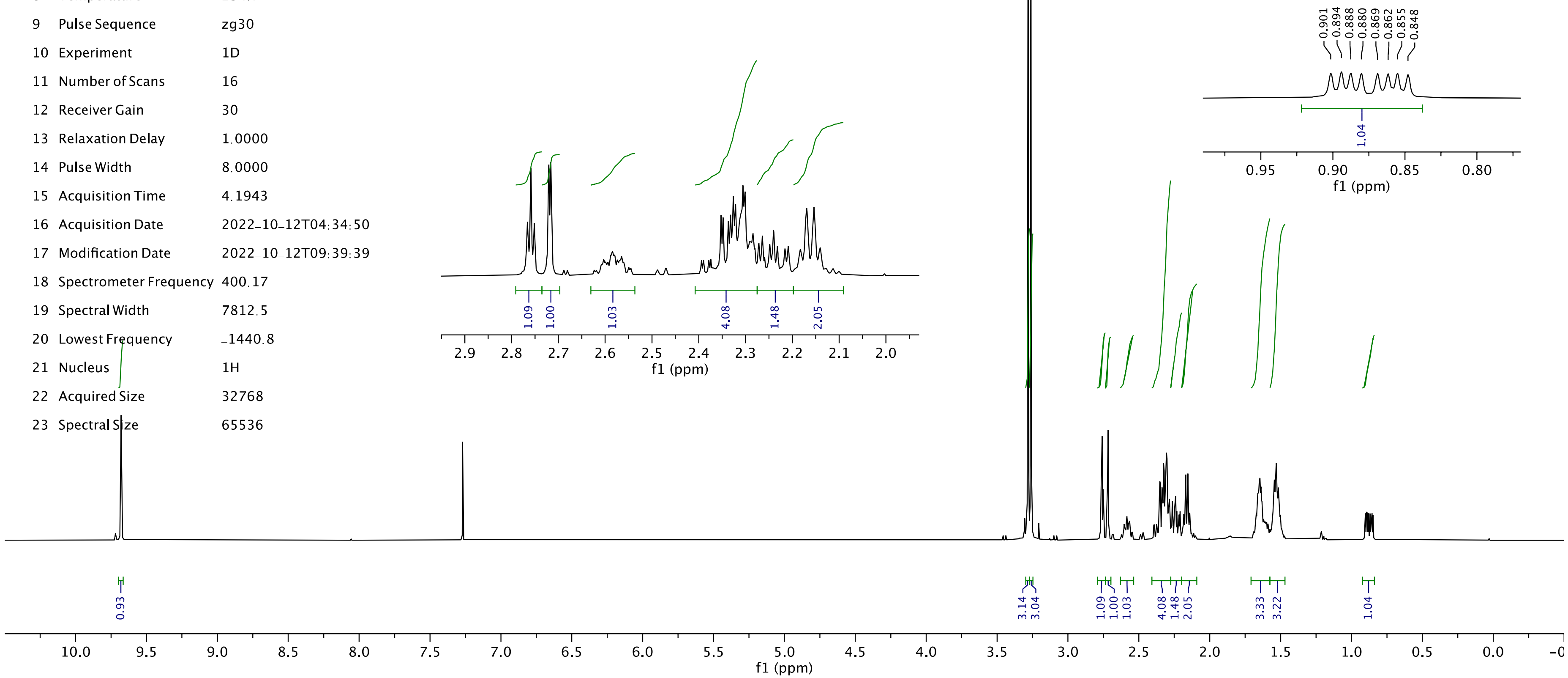

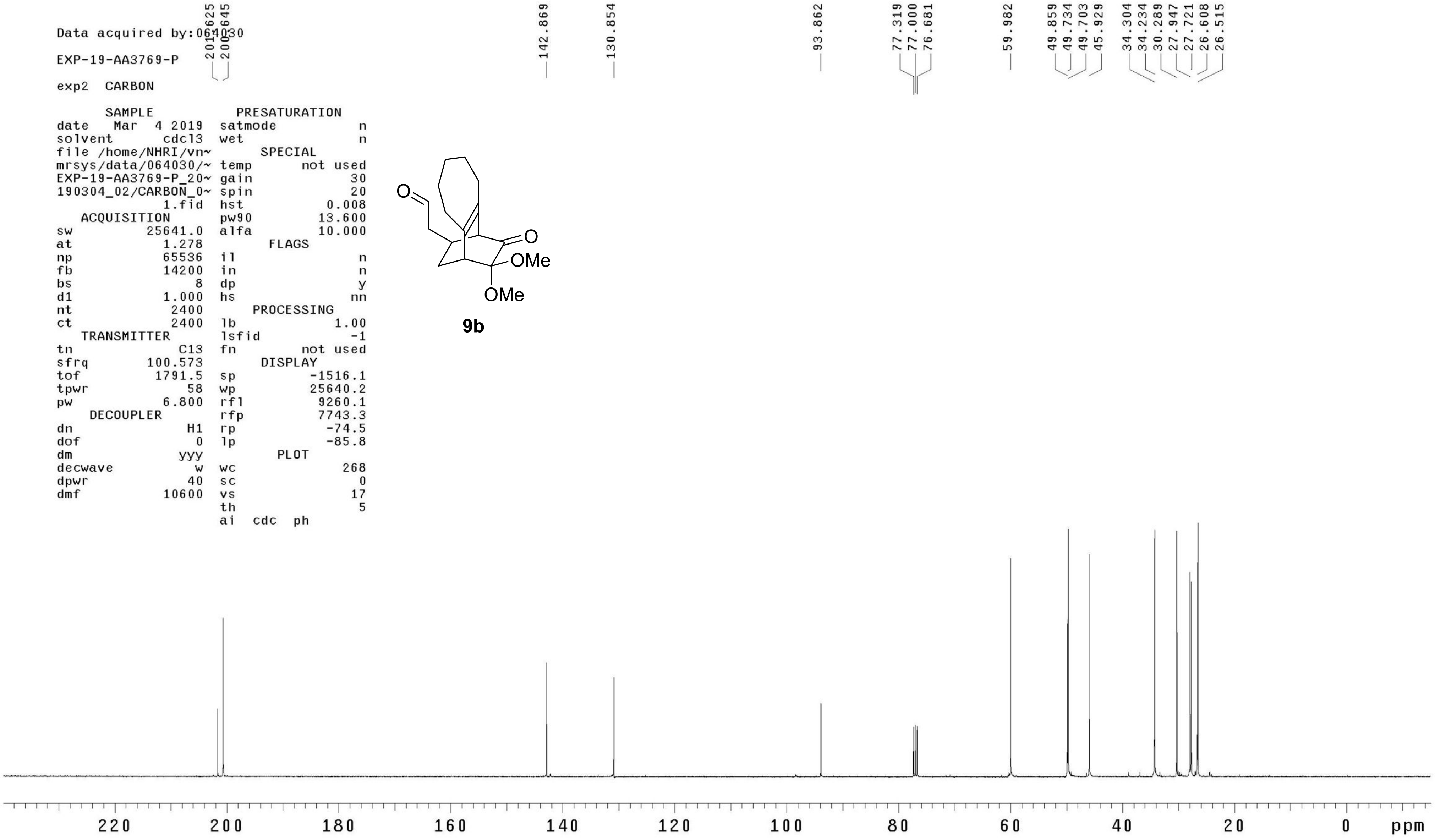

Data acquired by:064030
EXP-19-AA3769-P
exp2 CARBON
SAMPLE
date Mar 4 2019
solvent cdcl3
file /home/NHRI/vn~
mrsys/data/064030/~
EXP-19-AA3769-P_20~
190304_02/CARBON_0~
1.fid
ACQUISITION
sw 25641.0
at 1.278
np 65536
fb 14200
bs 8
d1 1.000
nt 2400
ct 2400
TRANSMITTER
tn C13
sfrq 100.573
tof 1791.5
tpwr 58
pw 6.800
DECOUPLER
dn H1
dof 0
dm yyy
decwave w
dpwr 40
dmf 10600
PRESATURATION
satmode n
wet n
SPECIAL
temp not used
gain 30
spin 20
hst 0.008
pw90 13.600
alfa 10.000
FLAGS
il n
in n
dp y
hs nn
PROCESSING
lb 1.00
lsfid -1
fn not used
DISPLAY
sp -1516.1
wp 25640.2
rfl 9260.1
rfp 7743.3
rp -74.5
lp -85.8
PLOT
wc 268
sc 0
vs 17
th 5
ai cdc ph
201.625
200.645
142.869
130.854
93.862
77.319
77.000
76.681
59.982
49.859
49.734
49.703
45.929
34.304
34.234
30.289
27.947
27.721
26.608
26.515
O
O
OMe
OMe
9b
220
200
180
160
140
120
100
80
60
40
20
0
ppm

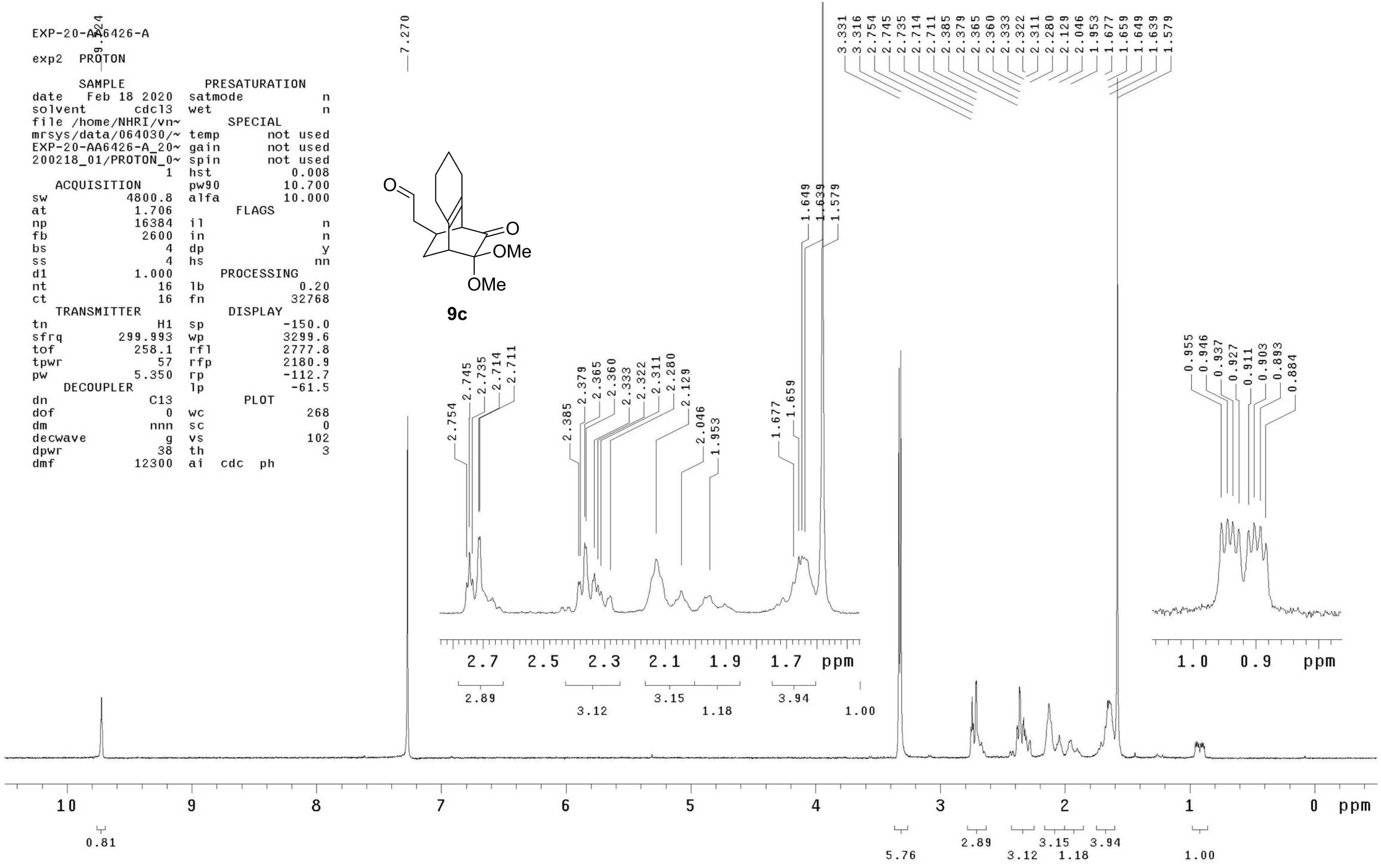
EXP-20-AA6426-A
exp2 PROTON
SAMPLE
date Feb 18 2020
solvent cdcl3
file /home/NHRI/vn~
mrsys/data/064030/~
EXP-20-AA6426-A_20~
200218_01/PROTON_0~
1
ACQUISITION
sw 4800.8
at 1.706
np 16384
fb 2600
bs 4
ss 4
d1 1.000
nt 16
ct 16
TRANSMITTER
tn H1
sfrq 299.993
tof 258.1
tpwr 57
pw 5.350
DECOUPLER
dn C13
dof 0
dm nnn
decwave g
dpwr 38
dmf 12300
PRESATURATION
satmode n
wet n
SPECIAL
temp not used
gain not used
spin not used
hst 0.008
pw90 10.700
alfa 10.000
FLAGS
il n
in n
dp y
hs nn
PROCESSING
lb 0.20
fn 32768
DISPLAY
sp -150.0
wp 3299.6
rfl 2777.8
rfp 2180.9
rp -112.7
lp -61.5
PLOT
wc 268
sc 0
vs 102
th 3
ai cdc ph
OMe
OMe
9c
ppm

Data acquired by: 064030

EXP-20-AA6426-A

exp2 CARBON

SAMPLE		PRESATURATION	
date	Mar 2 2020	satmode	n
solvent	cdcl3	wet	n
file /home/NHRI/vn~		SPECIAL	
mrsys/data/064030/~		temp	not used
EXP-20-AA6426-A_20~		gain	30
200302_01/CARBON_0~		spin	not used
	1.fid	hst	0.008
ACQUISITION		pw90	15.100
sw	18867.9	alfa	10.000
at	0.868	FLAGS	
np	32768	il	n
fb	10400	in	n
bs	8	dp	y
d1	1.000	hs	nn
nt	6000	PROCESSING	
ct	6000	lb	0.50
TRANSMITTER		fn	not used
tn	C13	DISPLAY	
sfrq	75.441	sp	-1137.0
tof	1138.1	wp	18866.8
tpwr	57	rfl	6946.5
pw	7.550	rfp	5808.3
DECOUPLER		rp	104.5
dn	H1	lp	-290.7
dof	0	PLOT	
dm	yyy	wc	268
decwave	w	sc	0
dpwr	35	vs	435
dmf	7700	th	6
		ai cdc ph	

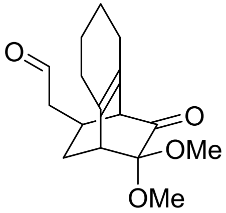

9c

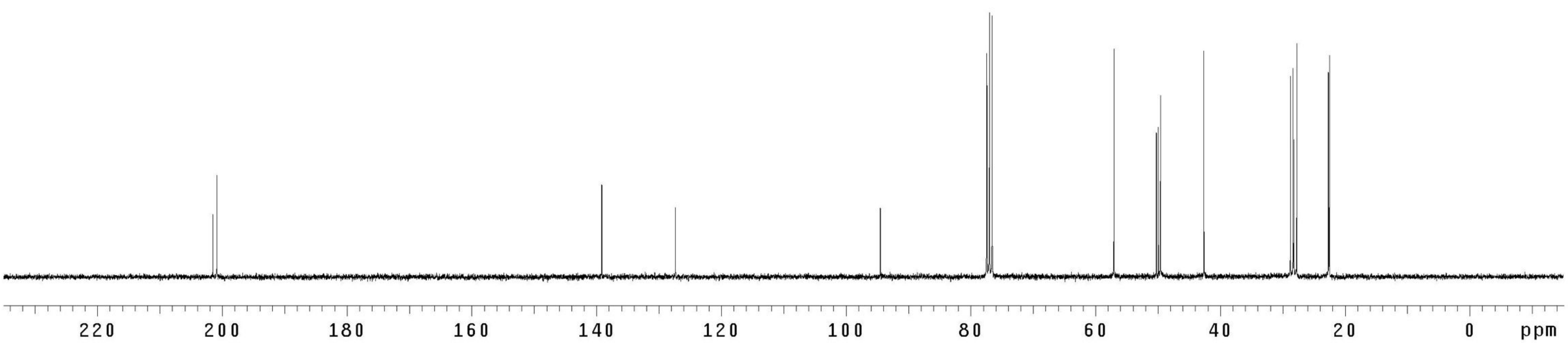

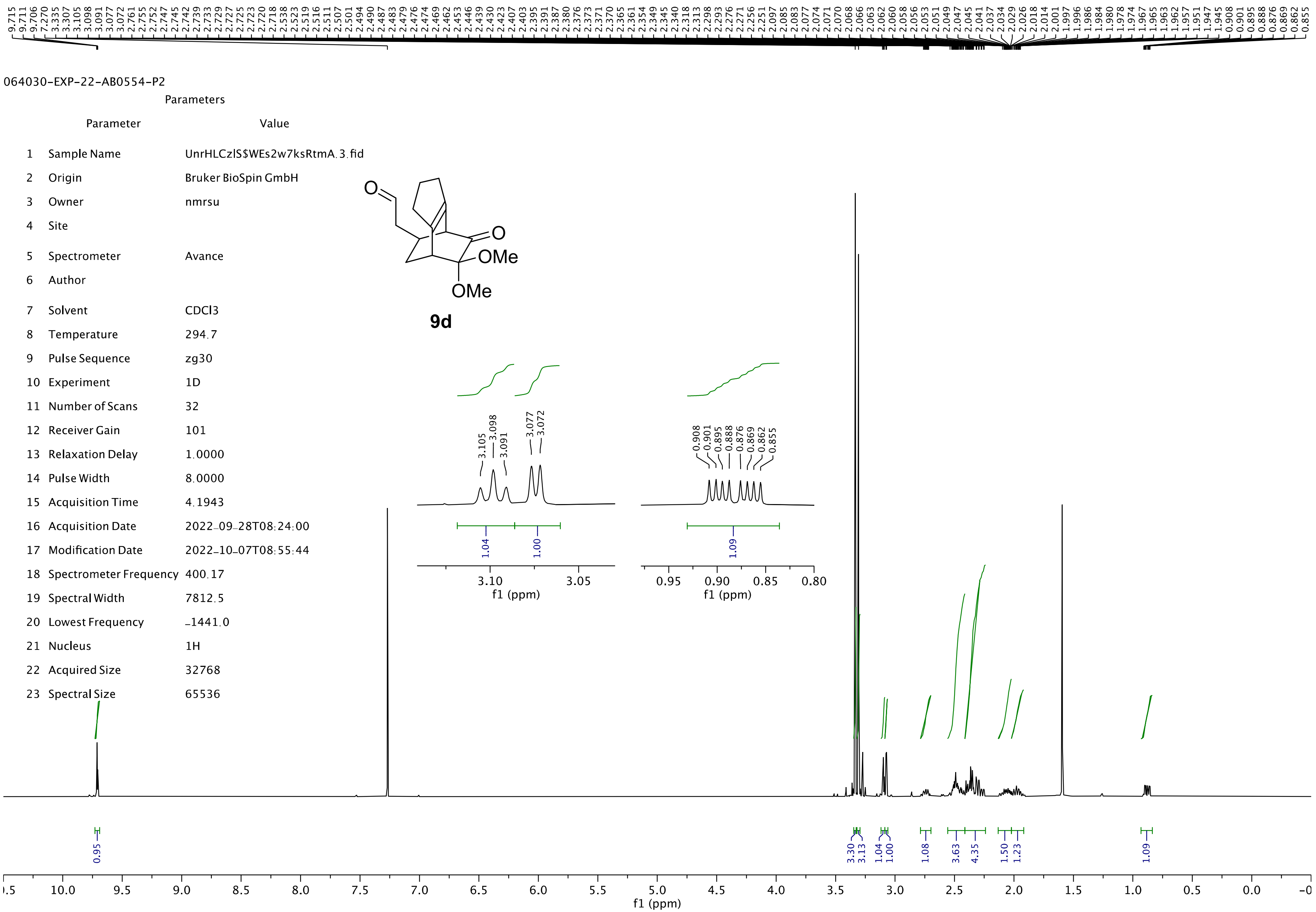

9.715 9.711 9.706 7.270 3.335 3.307 3.105 3.098 3.091 3.077 3.072 2.761 2.755 2.752 2.747 2.745 2.742 2.739 2.733 2.729 2.727 2.725 2.723 2.720 2.718 2.538 2.523 2.519 2.516 2.511 2.507 2.501 2.494 2.490 2.487 2.483 2.479 2.476 2.474 2.469 2.462 2.453 2.446 2.439 2.430 2.423 2.407 2.403 2.395 2.391 2.387 2.380 2.376 2.373 2.371 2.370 2.365 2.361 2.354 2.349 2.345 2.340 2.318 2.313 2.298 2.293 2.276 2.271 2.256 2.251 2.097 2.085 2.083 2.077 2.074 2.071 2.070 2.068 2.066 2.063 2.062 2.060 2.058 2.056 2.053 2.051 2.049 2.047 2.045 2.041 2.037 2.034 2.029 2.026 2.018 2.014 2.001 1.997 1.996 1.986 1.984 1.980 1.978 1.974 1.967 1.965 1.963 1.962 1.957 1.951 1.947 1.945 0.908 0.901 0.895 0.888 0.876 0.869 0.862 0.855
064030-EXP-22-AB0554-P2
Parameters
Parameter
Value
1 Sample Name UnrHLCzlS$WEs2w7ksRtmA.3.fid
2 Origin Bruker BioSpin GmbH
3 Owner nmrsu
4 Site
5 Spectrometer Avance
6 Author
7 Solvent CDCl3
8 Temperature 294.7
9 Pulse Sequence zg30
10 Experiment 1D
11 Number of Scans 32
12 Receiver Gain 101
13 Relaxation Delay 1.0000
14 Pulse Width 8.0000
15 Acquisition Time 4.1943
16 Acquisition Date 2022-09-28T08:24:00
17 Modification Date 2022-10-07T08:55:44
18 Spectrometer Frequency 400.17
19 Spectral Width 7812.5
20 Lowest Frequency -1441.0
21 Nucleus 1H
22 Acquired Size 32768
23 Spectral Size 65536
O
O
OMe
OMe
9d
3.105 3.098 3.091 3.077 3.072
1.04 1.00
3.10 3.05
f1 (ppm)
0.908 0.901 0.895 0.888 0.876 0.869 0.862 0.855
1.09
0.95 0.90 0.85 0.80
f1 (ppm)
0.95 3.30 3.13 1.04 1.00 1.08 3.63 4.35 1.50 1.23 1.09
10.0 9.5 9.0 8.5 8.0 7.5 7.0 6.5 6.0 5.5 5.0 4.5 4.0 3.5 3.0 2.5 2.0 1.5 1.0 0.5 0.0
f1 (ppm)

064030-EXP-22-AB0554-P2

Parameters

	Parameter	Value
1	Sample Name	UnrHLCzlS$WEs2w7ksRtmA.2.fid
2	Origin	Bruker BioSpin GmbH
3	Owner	nmrsu
4	Site	
5	Spectrometer	Avance
6	Author	
7	Solvent	CDCl3
8	Temperature	294.7
9	Pulse Sequence	zgpg30
10	Experiment	1D
11	Number of Scans	1040
12	Receiver Gain	101
13	Relaxation Delay	2.0000
14	Pulse Width	8.0000
15	Acquisition Time	1.3763
16	Acquisition Date	2022-09-28T08:12:40
17	Modification Date	2022-10-07T08:55:44
18	Spectrometer Frequency	100.63
19	Spectral Width	23809.5
20	Lowest Frequency	-846.6
21	Nucleus	13C
22	Acquired Size	32768
23	Spectral Size	65536

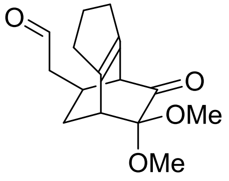

9d

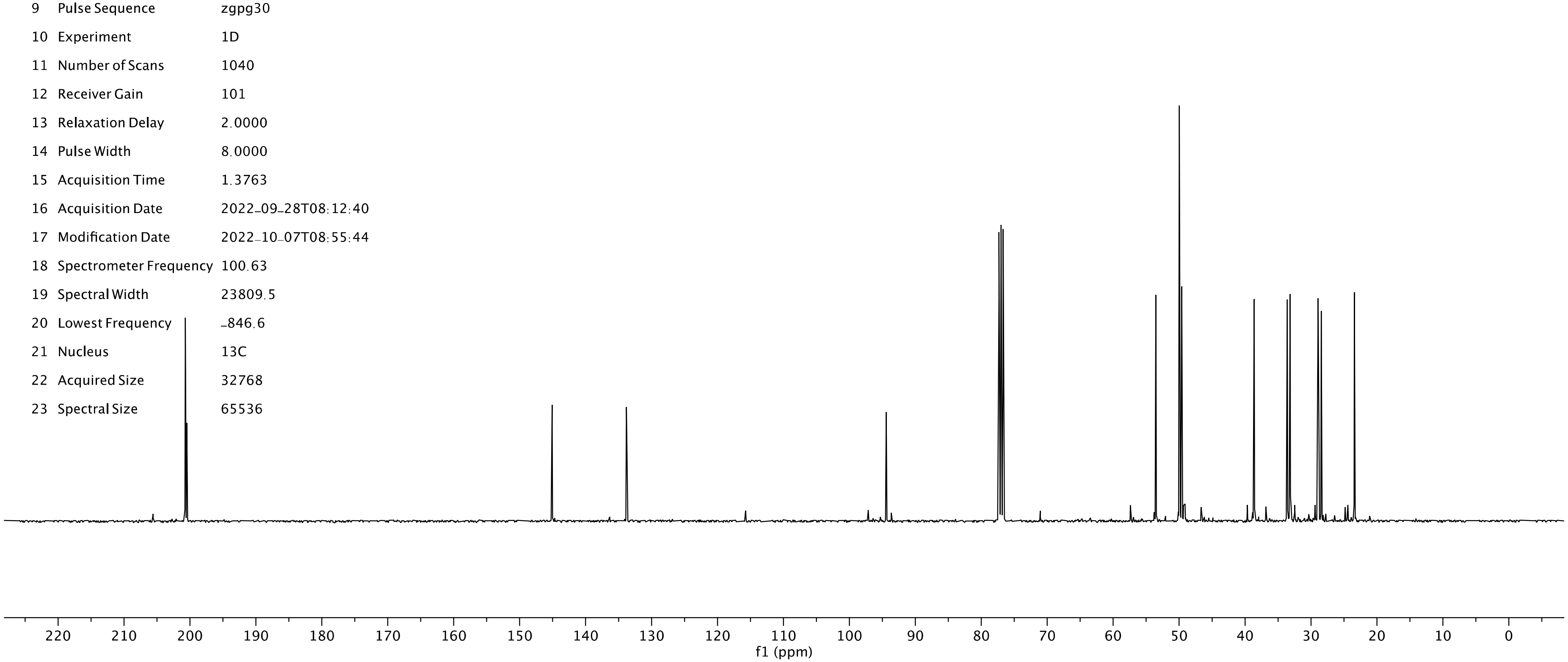

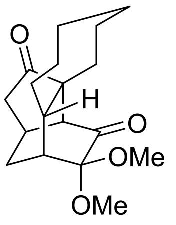

17a

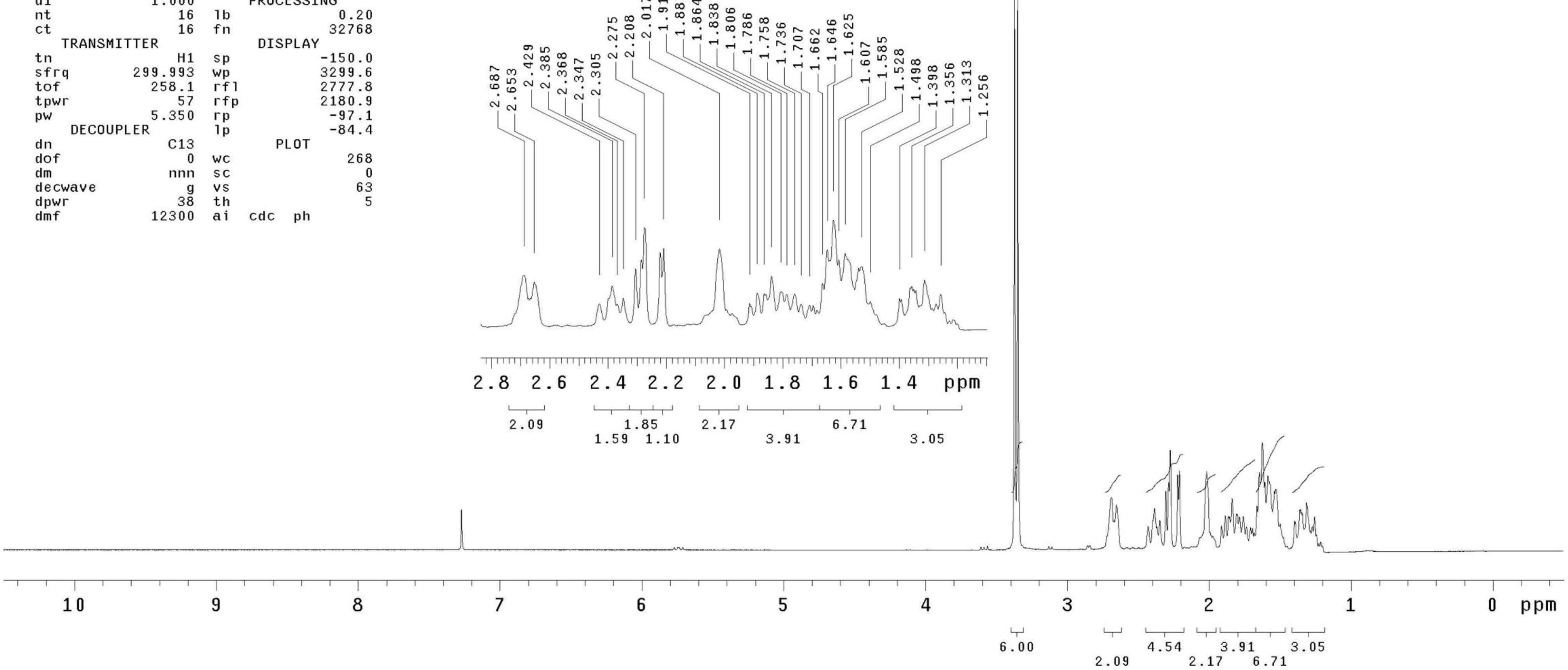
EXP-19-AA5661-42
exp4 PROTON
SAMPLE
date Dec 2 2019
solvent cdcl3
file /home/NHRI/vn~
mrsys/data/064030/~
EXP-19-AA5661-42_2~
0191202_01/PROTON_~
01
ACQUISITION
sw 4800.8
at 1.706
np 16384
fb 2600
bs 4
ss 4
d1 1.000
nt 16
ct 16
TRANSMITTER
tn H1
sfrq 299.993
tof 258.1
tpwr 57
pw 5.350
DECOUPLER
dn C13
dof 0
dm nnn
decwave g
dpwr 38
dmf 12300
PRESATURATION
satmode n
wet n
SPECIAL
temp not used
gain not used
spin not used
hst 0.008
pw90 10.700
alfa 10.000
FLAGS
il n
in n
dp y
hs nn
PROCESSING
lb 0.20
fn 32768
DISPLAY
sp -150.0
wp 3299.6
rfl 2777.8
rfp 2180.9
rp -97.1
lp -84.4
PLOT
wc 268
sc 0
vs 63
th 5
ai cdc ph
7.270
3.372 3.349 2.687 2.672 2.667 2.653 2.398 2.385 2.347 2.305 2.286 2.275 2.221 2.208 2.017 1.887 1.864 1.857 1.838 1.806 1.801 1.786 1.758 1.662 1.646 1.638 1.625 1.607 1.585 1.539 1.528 1.398 1.362 1.356 1.349 1.342 1.313 1.256
2.687 2.653 2.429 2.385 2.368 2.347 2.305 2.275 2.208 2.017 1.914 1.887 1.864 1.838 1.806 1.786 1.758 1.736 1.707 1.662 1.646 1.625 1.607 1.585 1.528 1.498 1.398 1.356 1.313 1.256
2.8 2.6 2.4 2.2 2.0 1.8 1.6 1.4 ppm
2.09 1.85 1.59 1.10 2.17 3.91 6.71 3.05
10 9 8 7 6 5 4 3 2 1 0 ppm
6.00 2.09 4.54 2.17 3.91 6.71 3.05

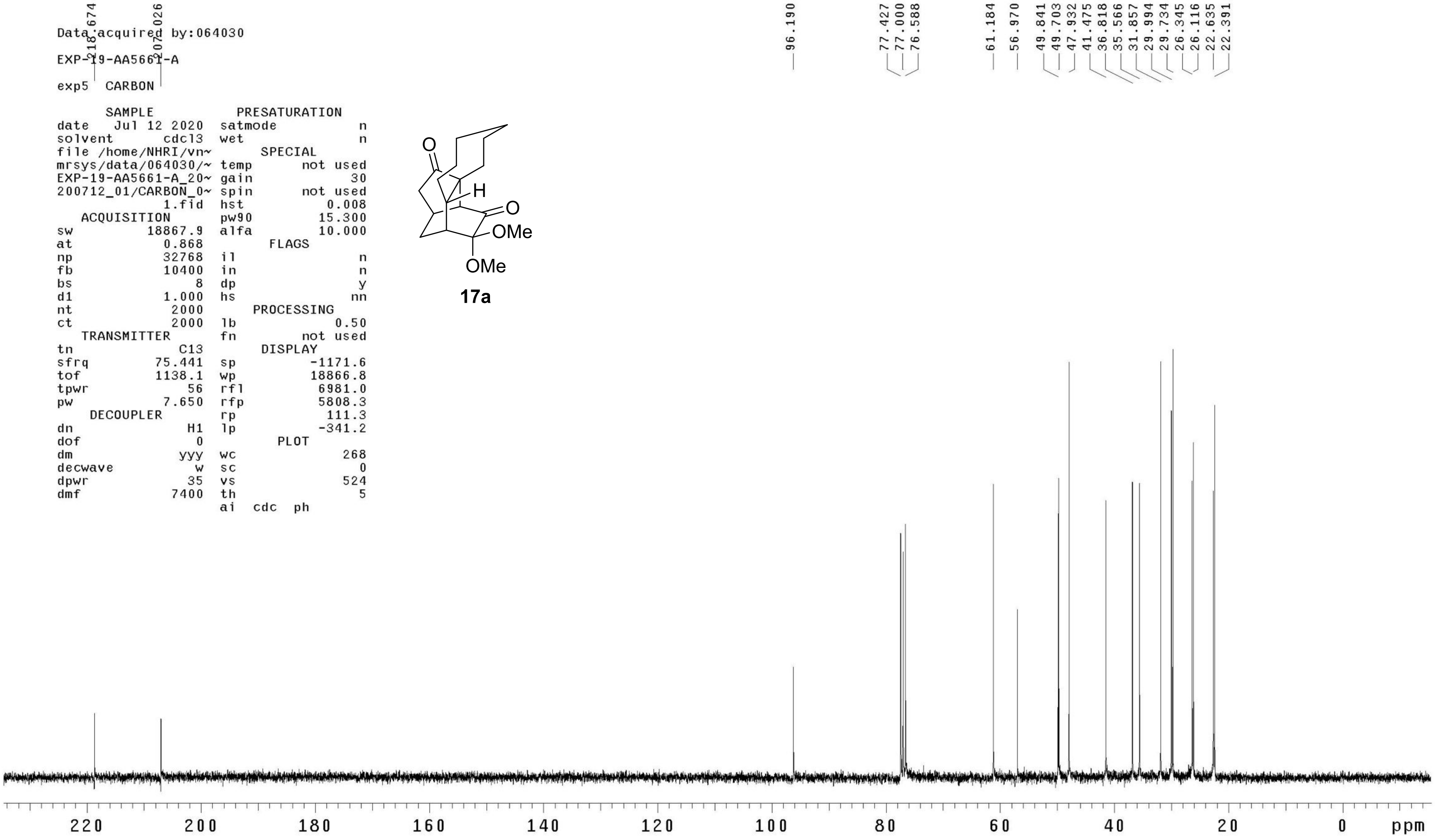

218.674
207.026
96.190
77.427
77.000
76.588
61.184
56.970
49.841
49.703
47.932
41.475
36.818
35.566
31.857
29.994
29.734
26.345
26.116
22.635
22.391
Data acquired by:064030
EXP-19-AA5661-A
exp5 CARBON
SAMPLE
date Jul 12 2020
solvent cdcl3
file /home/NHRI/vn~
mrsys/data/064030/~
EXP-19-AA5661-A_20~
200712_01/CARBON_0~
1.fid
ACQUISITION
sw 18867.9
at 0.868
np 32768
fb 10400
bs 8
d1 1.000
nt 2000
ct 2000
TRANSMITTER
tn C13
sfrq 75.441
tof 1138.1
tpwr 56
pw 7.650
DECOUPLER
dn H1
dof 0
dm yyy
decwave w
dpwr 35
dmf 7400
PRESATURATION
satmode n
wet n
SPECIAL
temp not used
gain 30
spin not used
hst 0.008
pw90 15.300
alfa 10.000
FLAGS
il n
in n
dp y
hs nn
PROCESSING
lb 0.50
fn not used
DISPLAY
sp -1171.6
wp 18866.8
rfl 6981.0
rfp 5808.3
rp 111.3
lp -341.2
PLOT
wc 268
sc 0
vs 524
th 5
ai cdc ph
O
H
O
OMe
OMe
17a
220 200 180 160 140 120 100 80 60 40 20 0 ppm

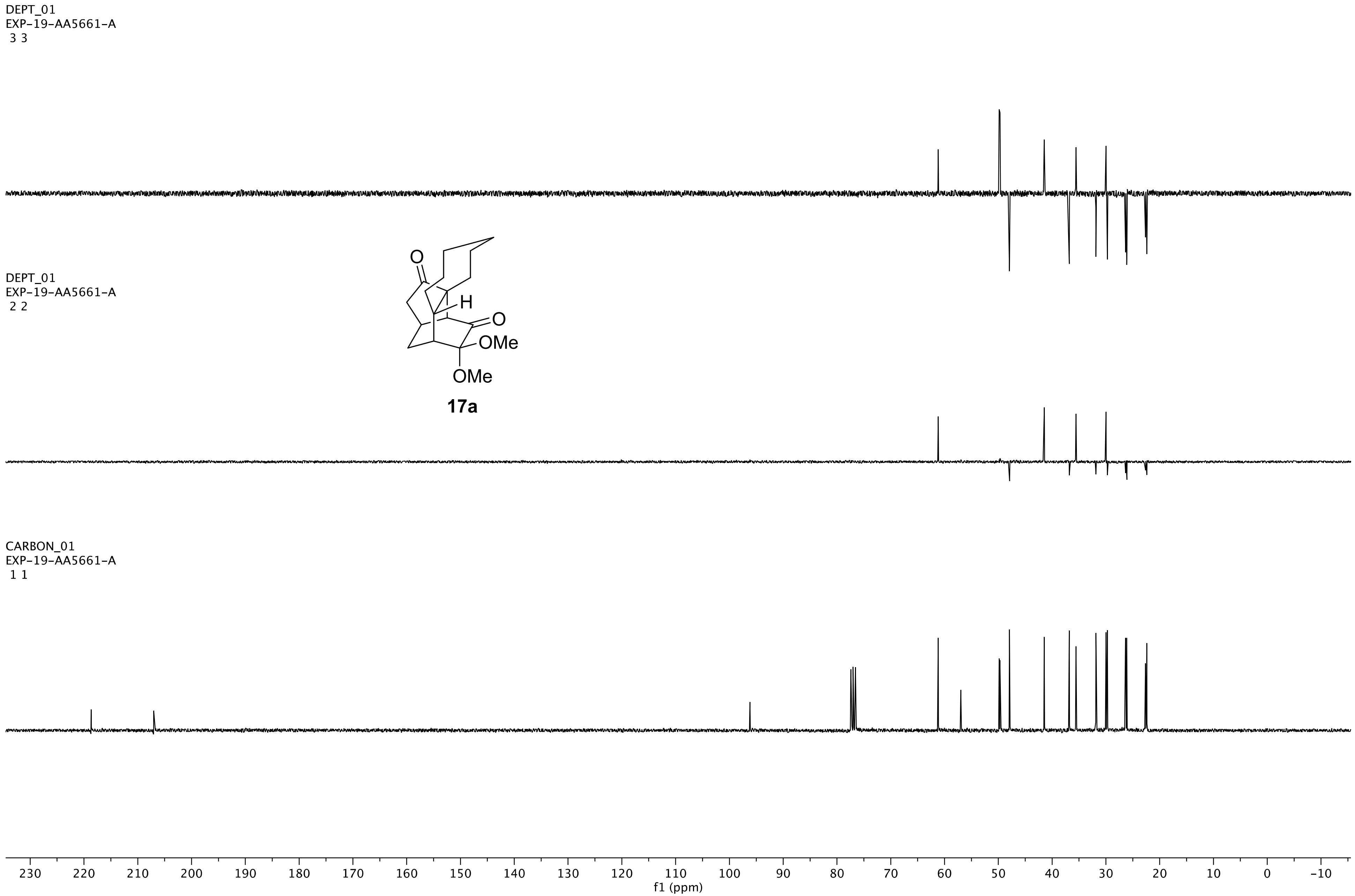
DEPT_01
EXP-19-AA5661-A
3 3
DEPT_01
EXP-19-AA5661-A
2 2
CARBON_01
EXP-19-AA5661-A
1 1
O
H
O
OMe
OMe
17a
230 220 210 200 190 180 170 160 150 140 130 120 110 100 90 80 70 60 50 40 30 20 10 0 −10
f1 (ppm)

PROTON_01
EXP-19-AA3770-ABC-aB-5456

Parameters

	Parameter	Value
1	2	/ Users / shaun / Desktop / NHRI / nmr backup / 300MHz / 064030-Lin_Sheng-Kuo / EXP-18-AA37 / EXP-19-AA3770-ABC-aB-5456_20200718_01 / PROTON_01.fid / fid
2	Title	PROTON_01
3	Comment	EXP-19-AA3770-ABC-aB-5456
4	Origin	Varian
5	Owner	
6	Site	
7	Instrument	mercury
8	Author	
9	Solvent	cdcl3
10	Temperature	25.0
11	Pulse Sequence	s2pul
12	Experiment	1D
13	Probe	Autosw
14	Number of Scans	16
15	Receiver Gain	18
16	Relaxation Delay	1.0000
17	Pulse Width	5.5000
18	Presaturation Frequency	
19	Acquisition Time	1.7064
20	Acquisition Date	2020-07-18T18:36:03
21	Modification Date	2020-12-11T20:25:55
22	Class	
23	mixN	
24	Spectrometer Frequency	299.99
25	Spectral Width	4800.8
26	Lowest Frequency	-742.2
27	Nucleus	1H
28	Acquired Size	8192
29	Spectral Size	65536

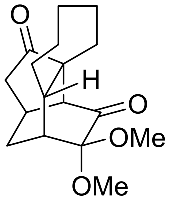

17b

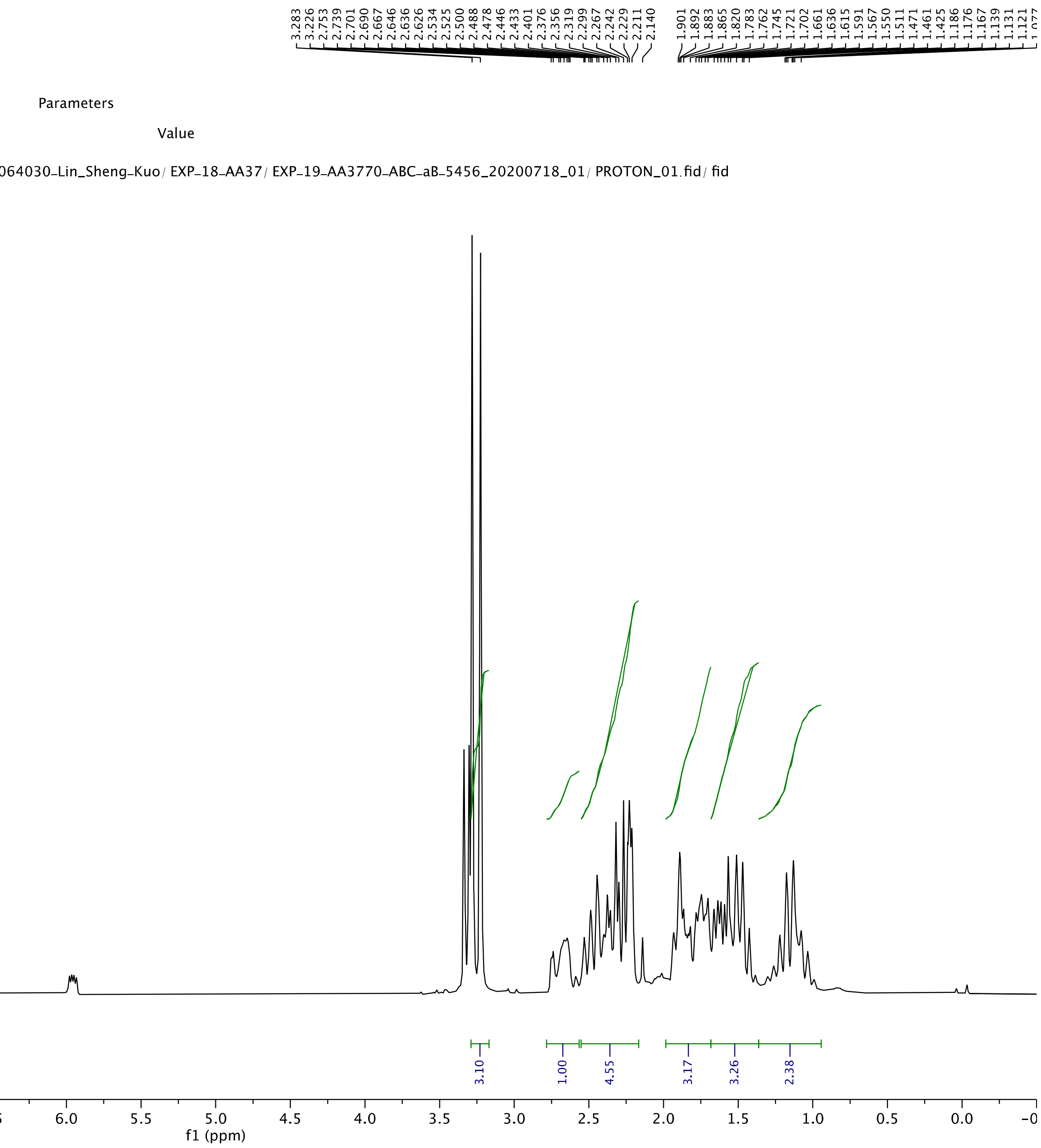

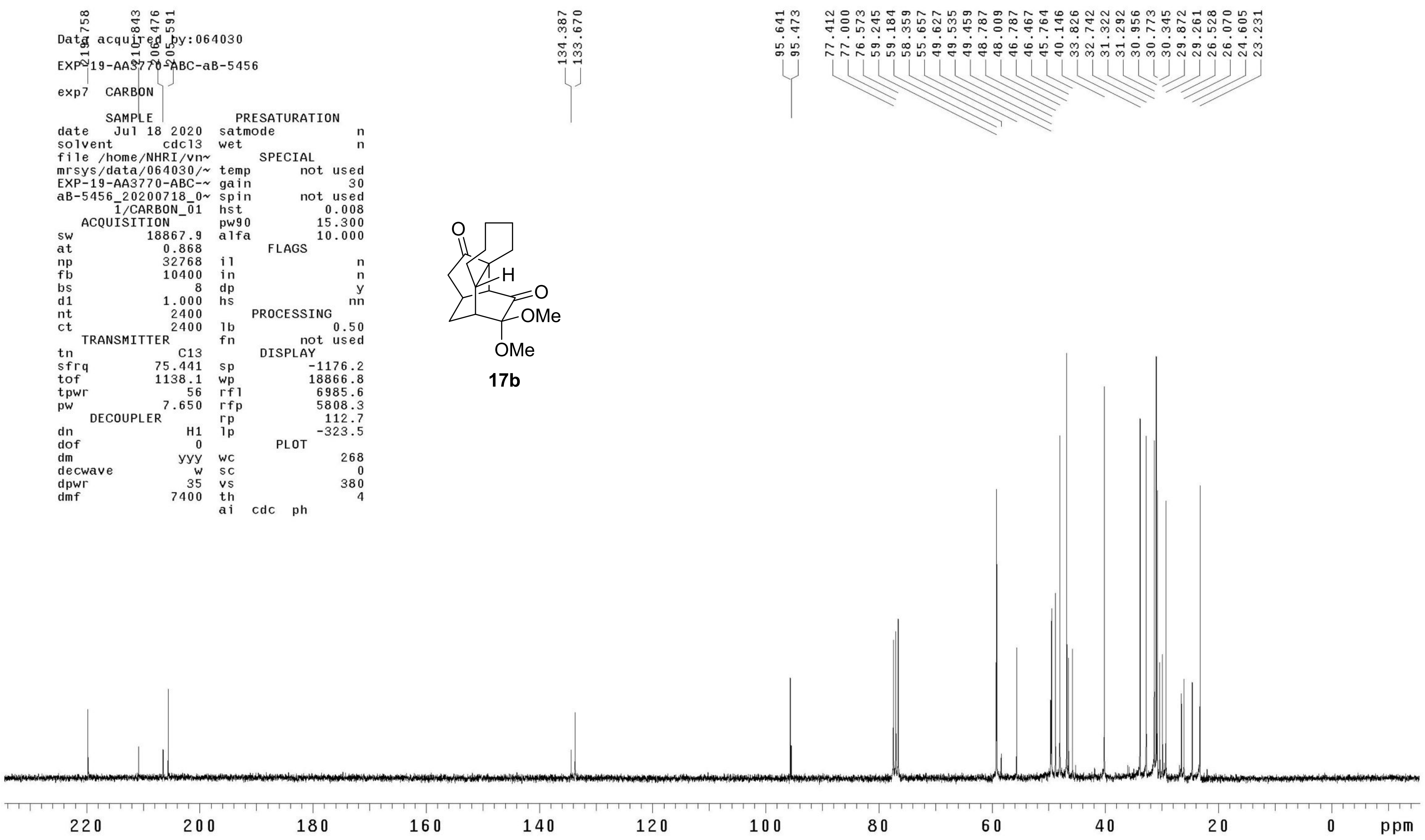

219.758
210.843
206.476
205.591
134.387
133.670
95.641
95.473
77.412
77.000
76.573
59.245
59.184
58.359
55.657
49.627
49.535
49.459
48.787
48.009
46.787
46.467
45.764
40.146
33.826
32.742
31.322
31.292
30.956
30.773
30.345
29.872
29.261
26.528
26.070
24.605
23.231
Data acquired by:064030
EXP-19-AA3770-ABC-aB-5456
exp7 CARBON
SAMPLE
date Jul 18 2020
solvent cdcl3
file /home/NHRI/vn~
mrsys/data/064030/~
EXP-19-AA3770-ABC-~
aB-5456_20200718_0~
1/CARBON_01
ACQUISITION
sw 18867.9
at 0.868
np 32768
fb 10400
bs 8
d1 1.000
nt 2400
ct 2400
TRANSMITTER
tn C13
sfrq 75.441
tof 1138.1
tpwr 56
pw 7.650
DECOUPLER
dn H1
dof 0
dm yyy
decwave w
dpwr 35
dmf 7400
PRESATURATION
satmode n
wet n
SPECIAL
temp not used
gain 30
spin not used
hst 0.008
pw90 15.300
alfa 10.000
FLAGS
il n
in n
dp y
hs nn
PROCESSING
lb 0.50
fn not used
DISPLAY
sp -1176.2
wp 18866.8
rfl 6985.6
rfp 5808.3
rp 112.7
lp -323.5
PLOT
wc 268
sc 0
vs 380
th 4
ai cdc ph
220
200
180
160
140
120
100
80
60
40
20
0
ppm
O
H
O
OMe
OMe

17b

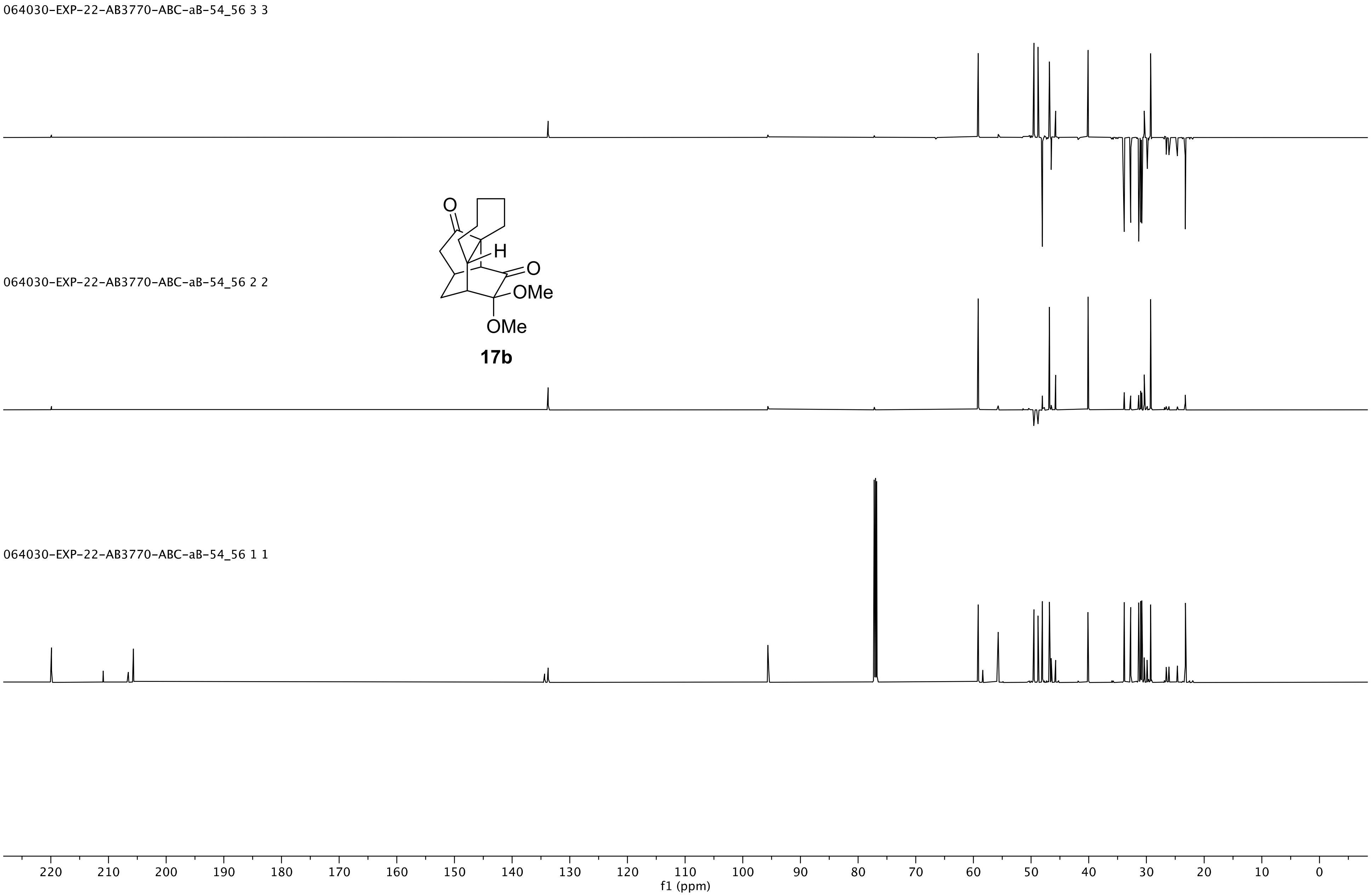
064030-EXP-22-AB3770-ABC-aB-54_56 3 3
064030-EXP-22-AB3770-ABC-aB-54_56 2 2
064030-EXP-22-AB3770-ABC-aB-54_56 1 1
O
H
O
OMe
OMe
17b
220 210 200 190 180 170 160 150 140 130 120 110 100 90 80 70 60 50 40 30 20 10 0
f1 (ppm)

PROTON_01
EXP-19-AA3770-ABC-Ab-4652

	Parameter	Value
1	2	/ Users / shaun / Desktop / NHRI / Collected Data / 7_Membered_Ring / 19. 137g(137i,138g)-EXP-19-AA3770 / 300MHz / EXP-19-AA3770-ABC-Ab-4652_20200717_01 / PROTON_01.fid / fid
2	Title	PROTON_01
3	Comment	EXP-19-AA3770-ABC-Ab-4652
4	Origin	Varian
5	Owner	
6	Site	
7	Instrument	mercury
8	Author	
9	Solvent	cdcl3
10	Temperature	25.0
11	Pulse Sequence	s2pul
12	Experiment	1D
13	Probe	Autosw
14	Number of Scans	16
15	Receiver Gain	16
16	Relaxation Delay	1.0000
17	Pulse Width	5.5000
18	Presaturation Frequency	
19	Acquisition Time	1.7064
20	Acquisition Date	2020-07-17T01:10:02
21	Modification Date	2020-09-02T21:08:23
22	Class	
23	mixN	
24	Spectrometer Frequency	299.99
25	Spectral Width	4800.8
26	Lowest Frequency	-767.7
27	Nucleus	1H
28	Acquired Size	8192
29	Spectral Size	65536

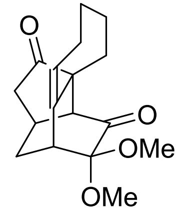

17b'

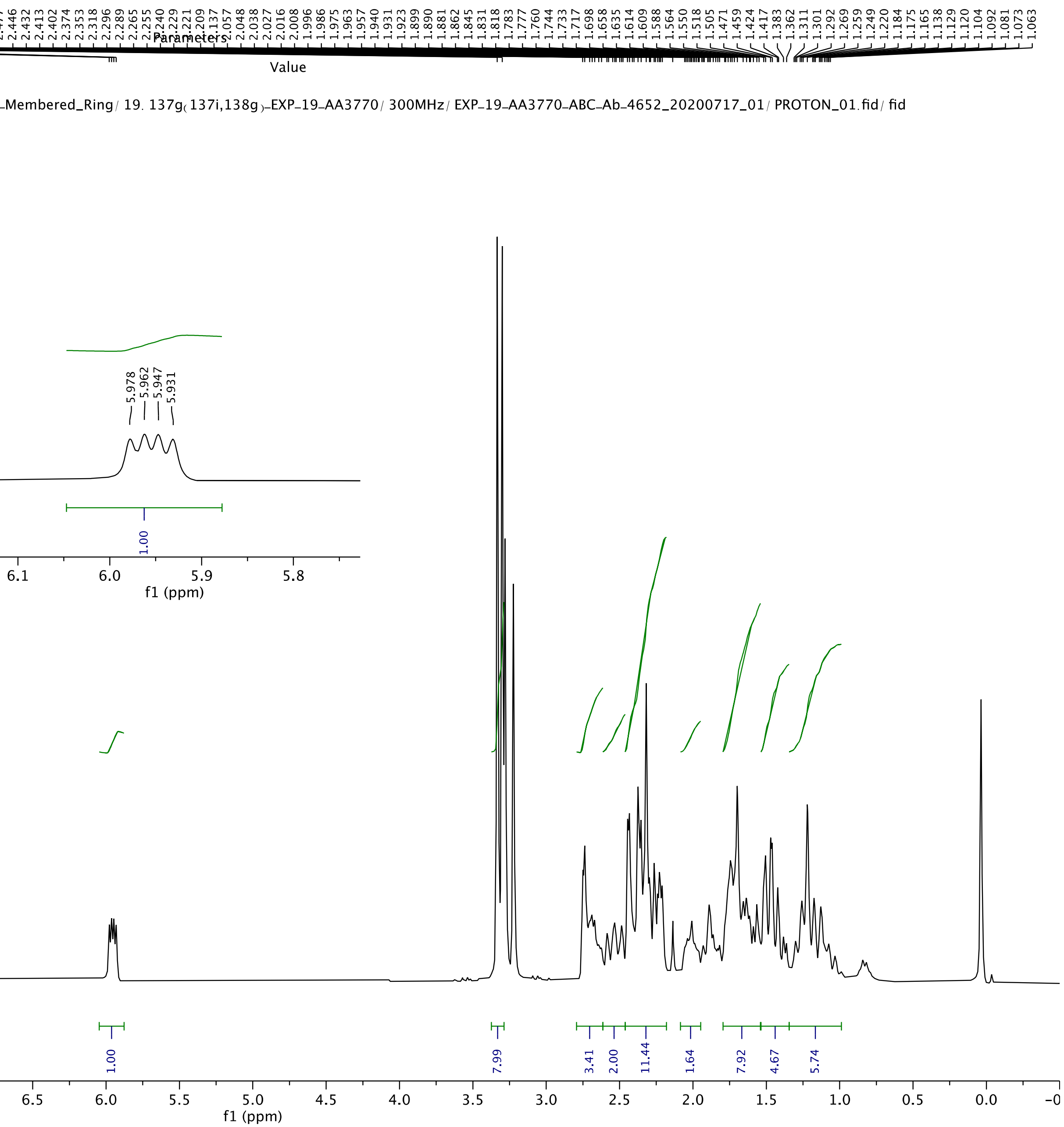

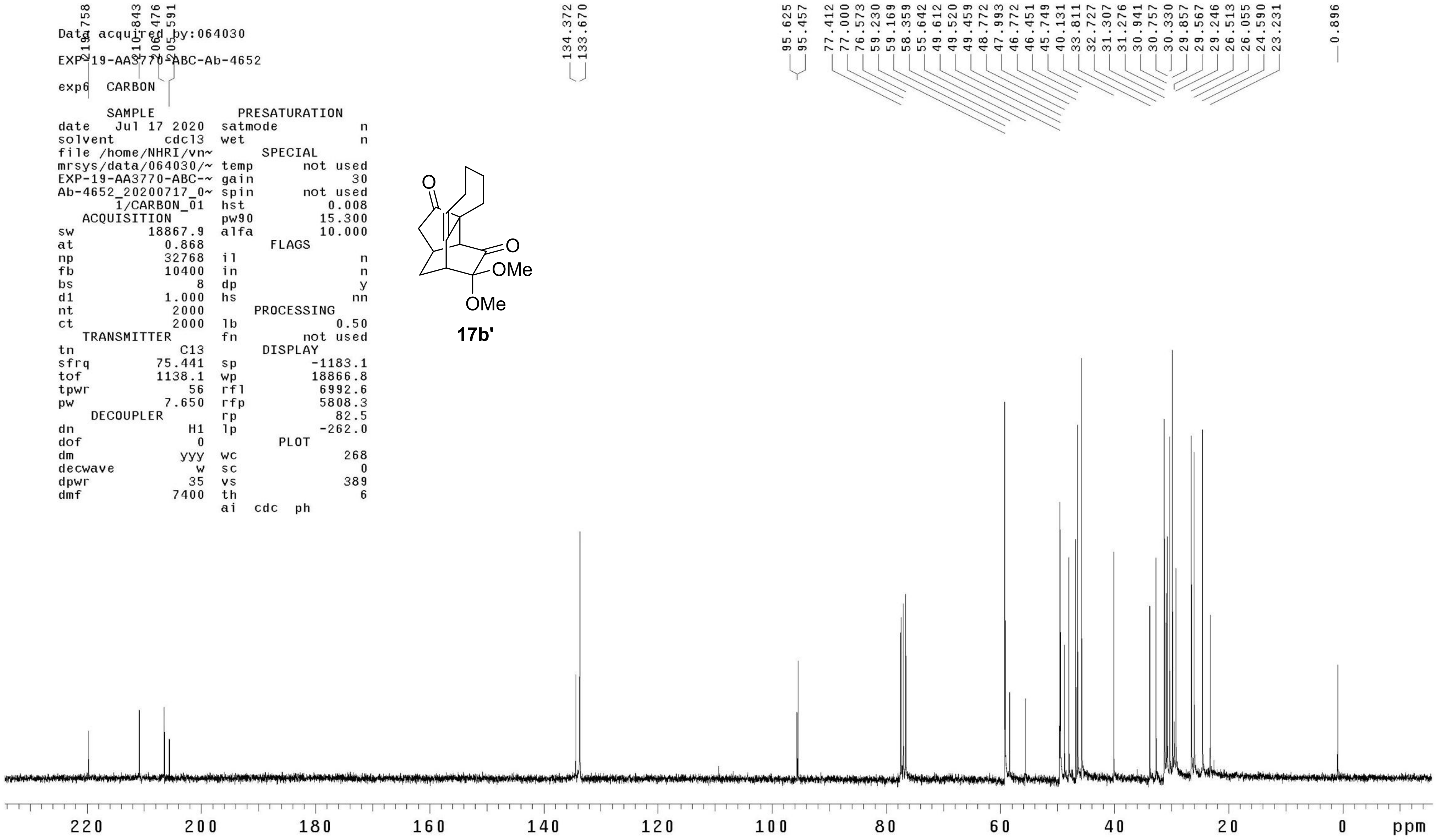
Data acquired by:064030
EXP-19-AA3770-ABC-Ab-4652
exp6 CARBON
SAMPLE
date Jul 17 2020
solvent cdcl3
file /home/NHRI/vn~
mrsys/data/064030/~
EXP-19-AA3770-ABC-~
Ab-4652_20200717_0~
1/CARBON_01
ACQUISITION
sw 18867.9
at 0.868
np 32768
fb 10400
bs 8
d1 1.000
nt 2000
ct 2000
TRANSMITTER
tn C13
sfrq 75.441
tof 1138.1
tpwr 56
pw 7.650
DECOUPLER
dn H1
dof 0
dm yyy
decwave w
dpwr 35
dmf 7400
PRESATURATION
satmode n
wet n
SPECIAL
temp not used
gain 30
spin not used
hst 0.008
pw90 15.300
alfa 10.000
FLAGS
il n
in n
dp y
hs nn
PROCESSING
lb 0.50
fn not used
DISPLAY
sp -1183.1
wp 18866.8
rfl 6992.6
rfp 5808.3
rp 82.5
lp -262.0
PLOT
wc 268
sc 0
vs 389
th 6
ai cdc ph
210.843
206.476
205.591
134.372
133.670
95.625
95.457
77.412
77.000
76.573
59.230
59.169
58.359
55.642
49.612
49.520
49.459
48.772
47.993
46.772
46.451
45.749
40.131
33.811
32.727
31.307
31.276
30.941
30.757
30.330
29.857
29.567
29.246
26.513
26.055
24.590
23.231
0.896
O
O
OMe
OMe
17b'
220
200
180
160
140
120
100
80
60
40
20
0
ppm

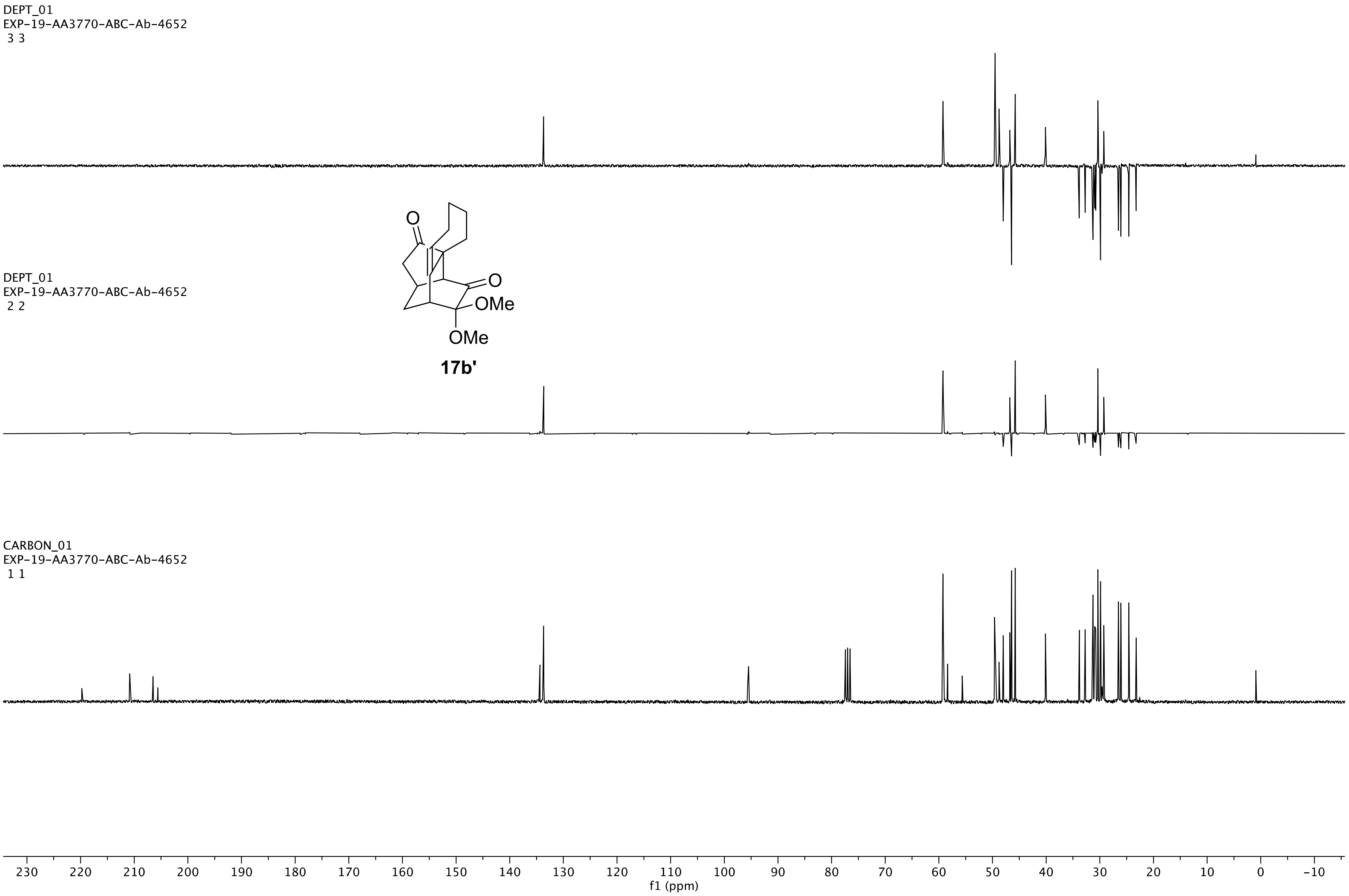
DEPT_01
EXP-19-AA3770-ABC-Ab-4652
3 3
O
O
OMe
OMe
17b'
DEPT_01
EXP-19-AA3770-ABC-Ab-4652
2 2
CARBON_01
EXP-19-AA3770-ABC-Ab-4652
1 1
230 220 210 200 190 180 170 160 150 140 130 120 110 100 90 80 70 60 50 40 30 20 10 0 -10
f1 (ppm)

PROTON_01
EXP-19-AA4439-A

	Parameter	Value
1	2	/ Users / shaun / Desktop / NHRI / Collected Data / 5_Membered_Ring / 13. 137e_EXP_19_AA4439 / 300MHz / EXP_19_AA4439_A_20190725_01 / PROTON_01.fid / fid
2	Title	PROTON_01
3	Comment	EXP_19_AA4439_A
4	Origin	Varian
5	Owner	
6	Site	
7	Instrument	mercury
8	Author	
9	Solvent	cdcl3
10	Temperature	25.0
11	Pulse Sequence	s2pul
12	Experiment	1D
13	Probe	Autosw
14	Number of Scans	16
15	Receiver Gain	24
16	Relaxation Delay	1.0000
17	Pulse Width	5.3500
18	Presaturation Frequency	
19	Acquisition Time	1.7064
20	Acquisition Date	2019_07_25T11:48:53
21	Modification Date	2020_07_11T21:14:44
22	Class	
23	mixN	
24	Spectrometer Frequency	299.99
25	Spectral Width	4800.8
26	Lowest Frequency	_596.7
27	Nucleus	1H
28	Acquired Size	8192
29	Spectral Size	65536

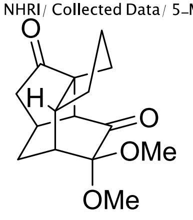

17d

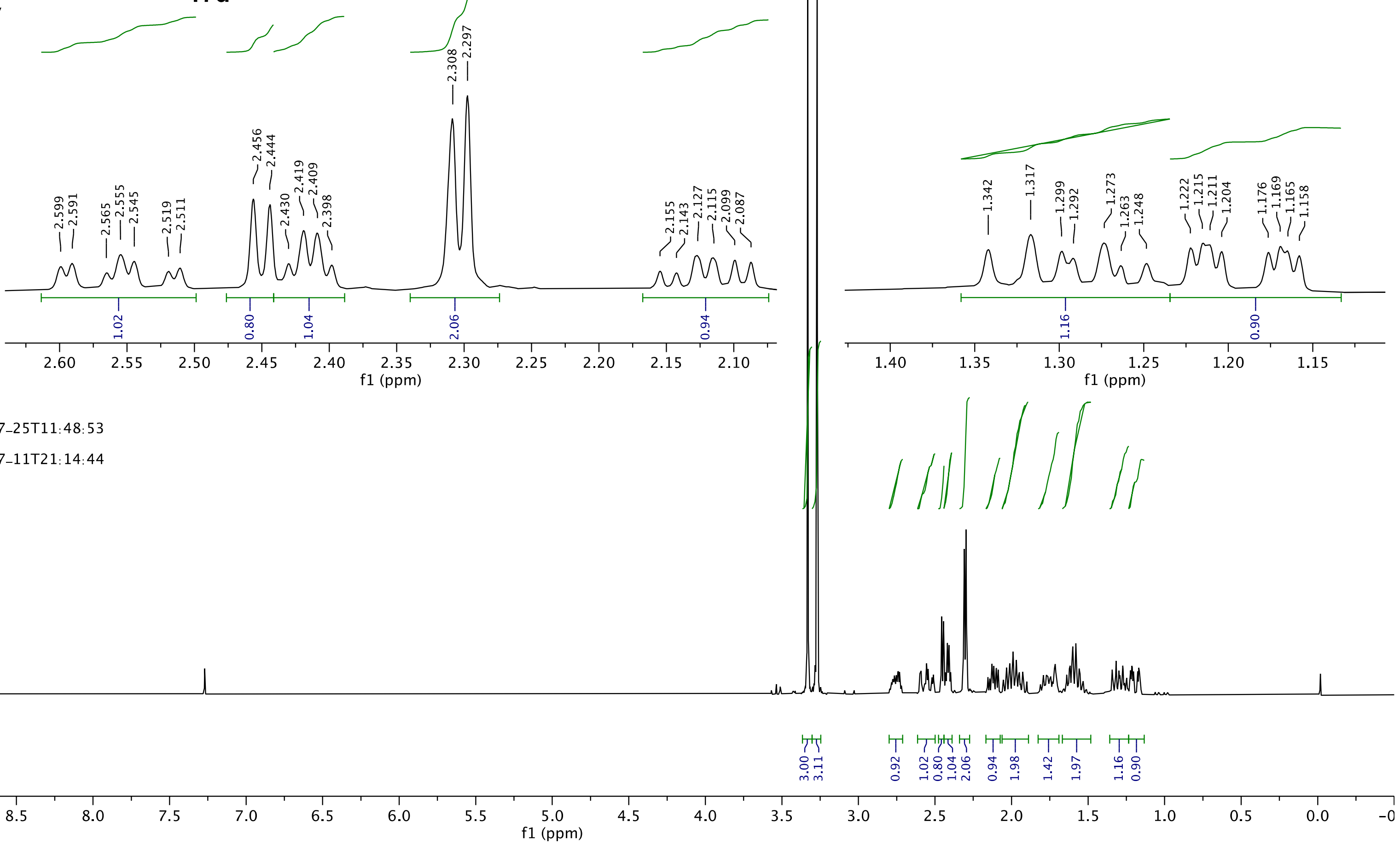

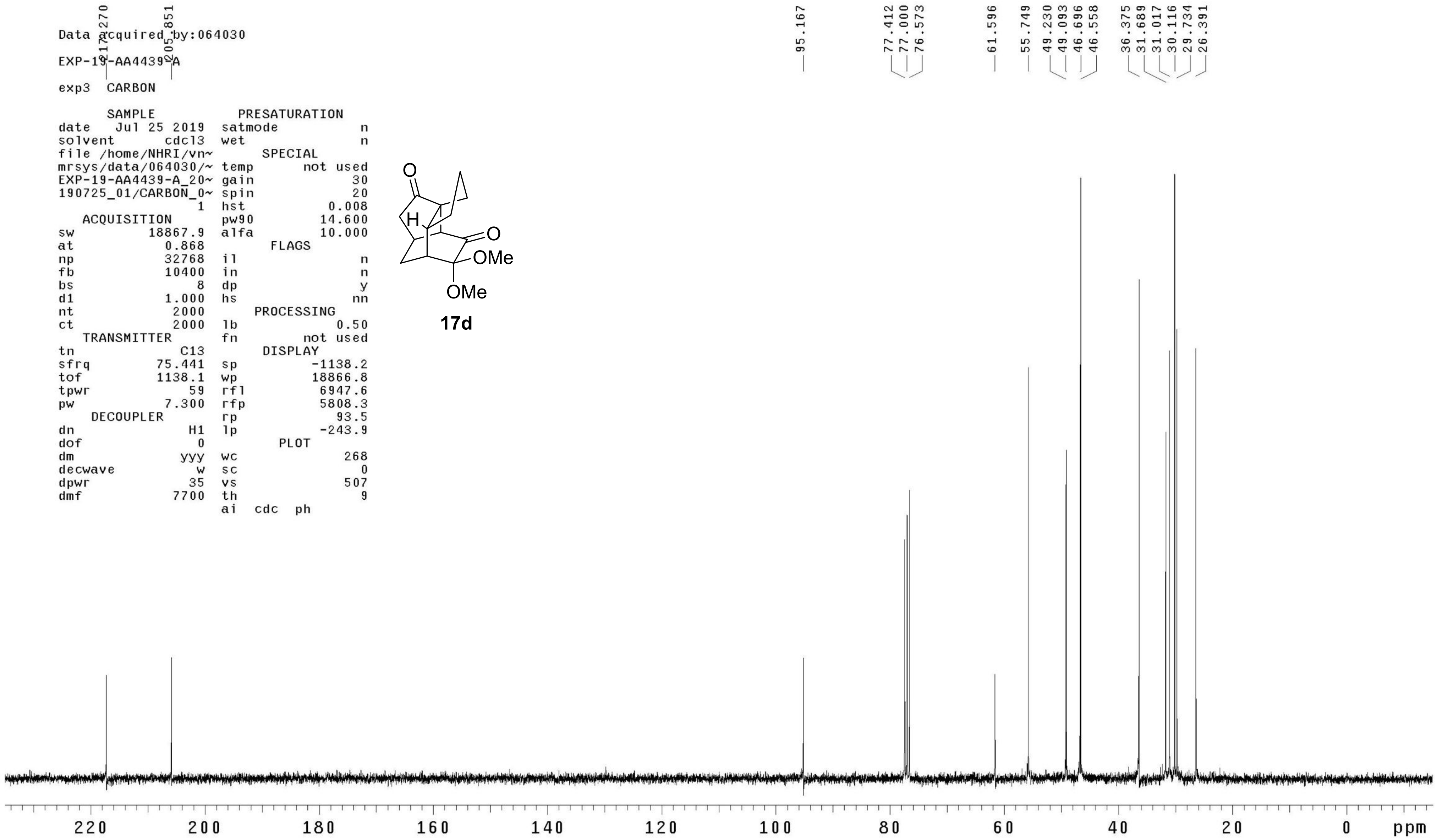
Data acquired by:064030
EXP-19-AA4439-A
exp3 CARBON
SAMPLE
date Jul 25 2019
solvent cdcl3
file /home/NHRI/vn~
mrsys/data/064030/~
EXP-19-AA4439-A_20~
190725_01/CARBON_0~
1
ACQUISITION
sw 18867.9
at 0.868
np 32768
fb 10400
bs 8
d1 1.000
nt 2000
ct 2000
TRANSMITTER
tn C13
sfrq 75.441
tof 1138.1
tpwr 59
pw 7.300
DECOUPLER
dn H1
dof 0
dm yyy
decwave w
dpwr 35
dmf 7700
PRESATURATION
satmode n
wet n
SPECIAL
temp not used
gain 30
spin 20
hst 0.008
pw90 14.600
alfa 10.000
FLAGS
il n
in n
dp y
hs nn
PROCESSING
lb 0.50
fn not used
DISPLAY
sp -1138.2
wp 18866.8
rfl 6947.6
rfp 5808.3
rp 93.5
lp -243.9
PLOT
wc 268
sc 0
vs 507
th 9
ai cdc ph
217.270
205.851
95.167
77.412
77.000
76.573
61.596
55.749
49.230
49.093
46.696
46.558
36.375
31.689
31.017
30.116
29.734
26.391
220
200
180
160
140
120
100
80
60
40
20
0
ppm
O
H
O
OMe
OMe

17d

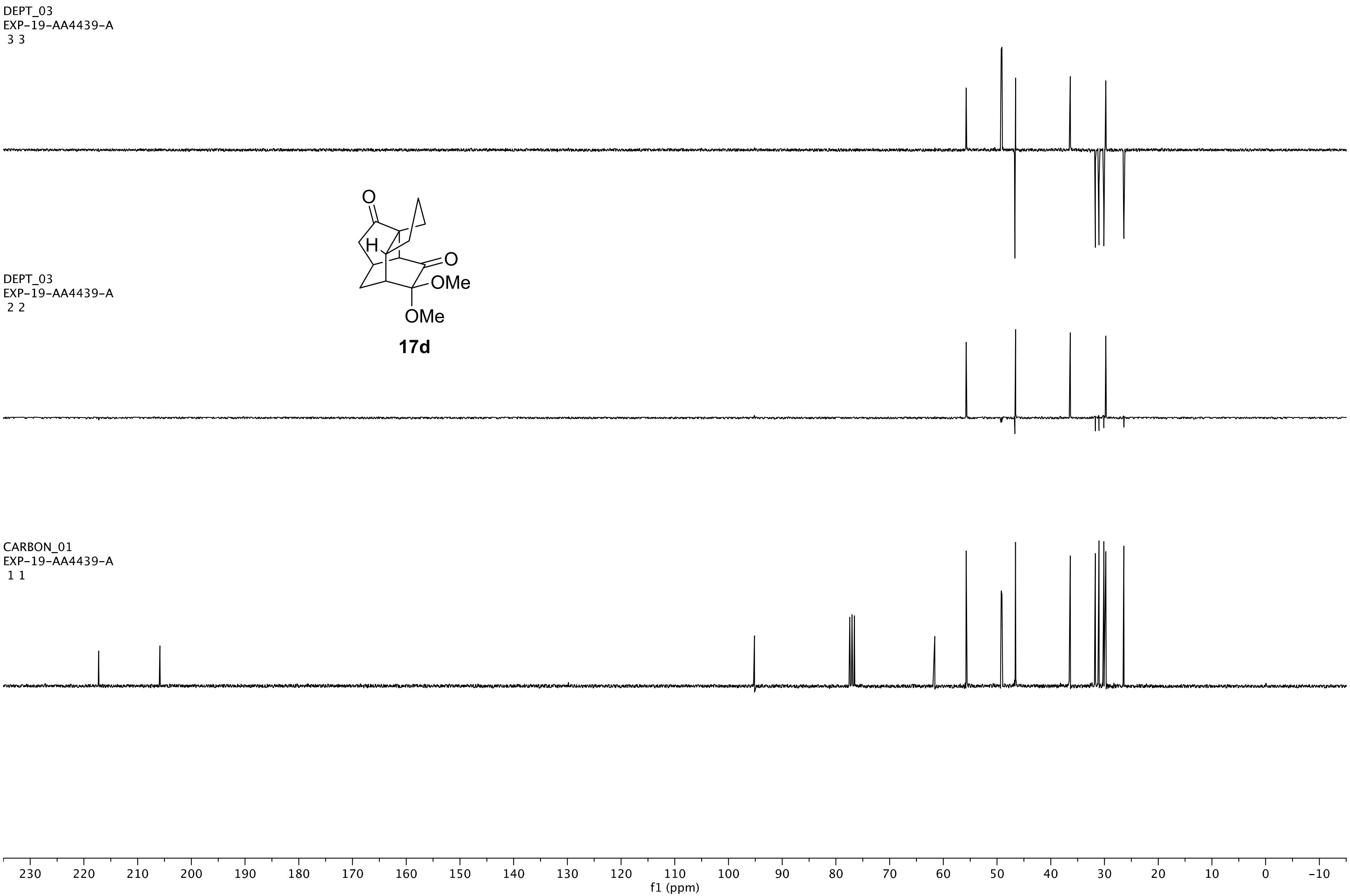
DEPT_03
EXP-19-AA4439-A
3 3
O
H
O
OMe
OMe
DEPT_03
EXP-19-AA4439-A
2 2
CARBON_01
EXP-19-AA4439-A
1 1
230 220 210 200 190 180 170 160 150 140 130 120 110 100 90 80 70 60 50 40 30 20 10 0 -10
f1 (ppm)

17d

PROTON_02
EXP-19-AA3770-ABC-C

	Parameter	Value
1	2	/ Users / shaun / Desktop / NHRI / Collected Data / 7_Membered_Ring / EXP_19_AA3770 / 400MHz / EXP_19_AA3770_ABC_C_20190331_01 / PROTON_02.fid / fid
2	Title	PROTON_02
3	Comment	EXP_19_AA3770_ABC_C
4	Origin	Varian
5	Owner	
6	Site	
7	Instrument	mercury
8	Author	
9	Solvent	cdcl3
10	Temperature	25.0
11	Pulse Sequence	s2pul
12	Experiment	1D
13	Probe	autosw
14	Number of Scans	16
15	Receiver Gain	20
16	Relaxation Delay	1.0000
17	Pulse Width	7.2500
18	Presaturation Frequency	
19	Acquisition Time	2.5608
20	Acquisition Date	2019_03_31T01:59:14
21	Modification Date	2020_07_14T01:24:33
22	Class	
23	mixN	
24	Spectrometer Frequency	399.93
25	Spectral Width	6398.0
26	Lowest Frequency	_794.8
27	Nucleus	1H
28	Acquired Size	16384
29	Spectral Size	65536

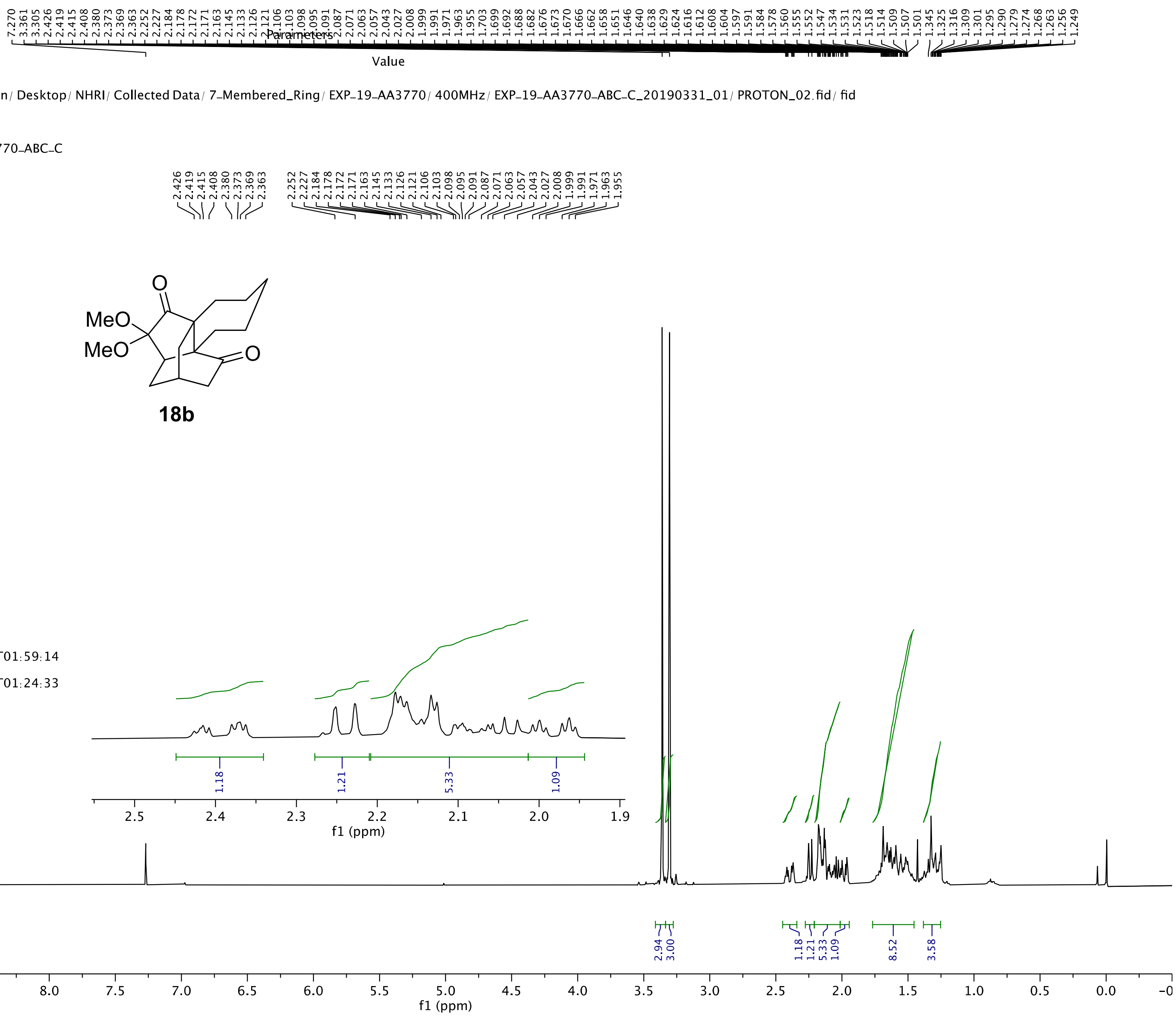

Data acquired by:064030

EXP-19-AA3770-ABC-C

exp2 CARBON

SAMPLE		PRESATURATION	
date	Mar 31 2019	satmode	n
solvent	cdcl3	wet	n
file /home/NHRI/vn~		SPECIAL	
mrsys/data/064030/~		temp	not used
EXP-19-AA3770-ABC-~		gain	30
C_20190331_01/CARB~		spin	20
ON_01.fid		hst	0.008
ACQUISITION		pw90	13.600
sw	25125.6	alfa	10.000
at	1.304	FLAGS	
np	65536	il	n
fb	13800	in	n
bs	8	dp	y
d1	1.000	hs	nn
nt	5000	PROCESSING	
ct	5000	lb	1.00
TRANSMITTER		fn	not used
tn	C13	DISPLAY	
sfrq	100.573	sp	-1501.7
tof	1535.0	wp	25124.9
tpwr	58	rfl	9245.8
pw	6.800	rfp	7743.3
DECOUPLER		rp	39.3
dn	H1	lp	-397.7
dof	0	PLOT	
dm	yyy	wc	268
decwave	w	sc	0
dpwr	40	vs	255
dmf	10600	th	8
		ai cdc ph	

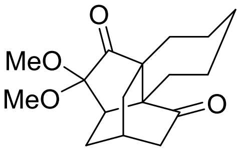

18b

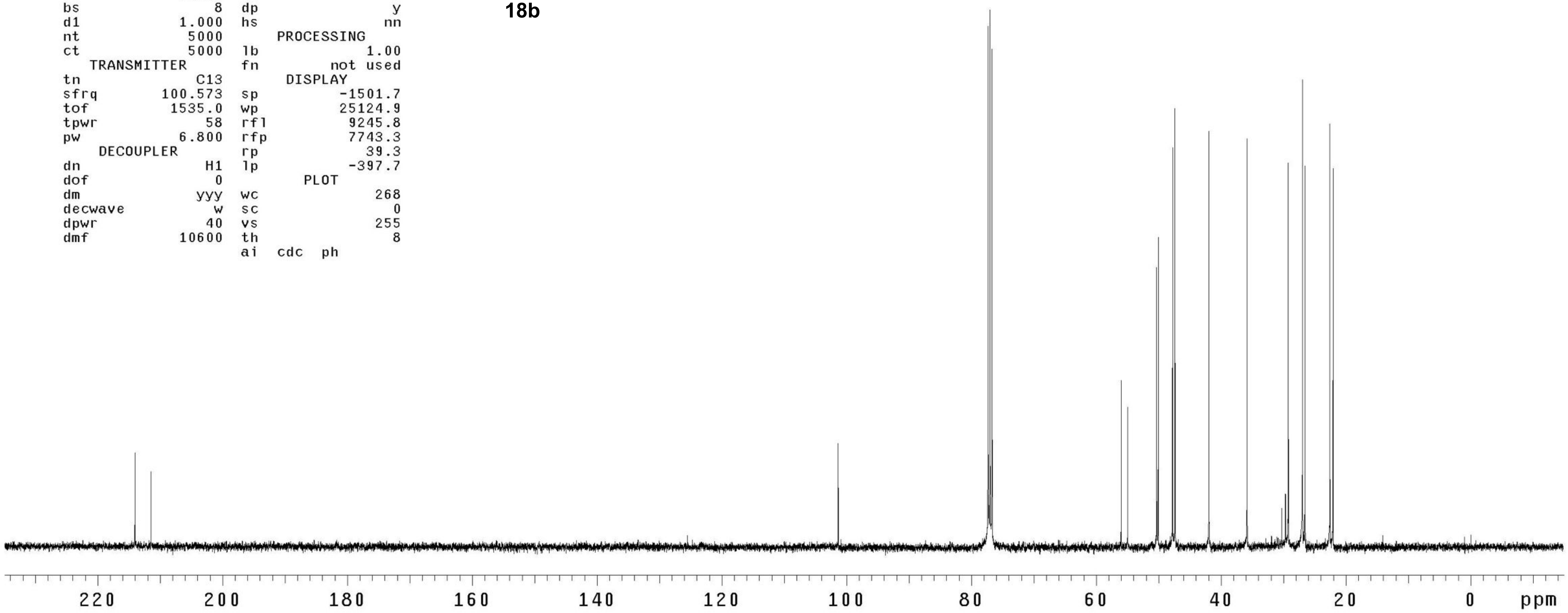

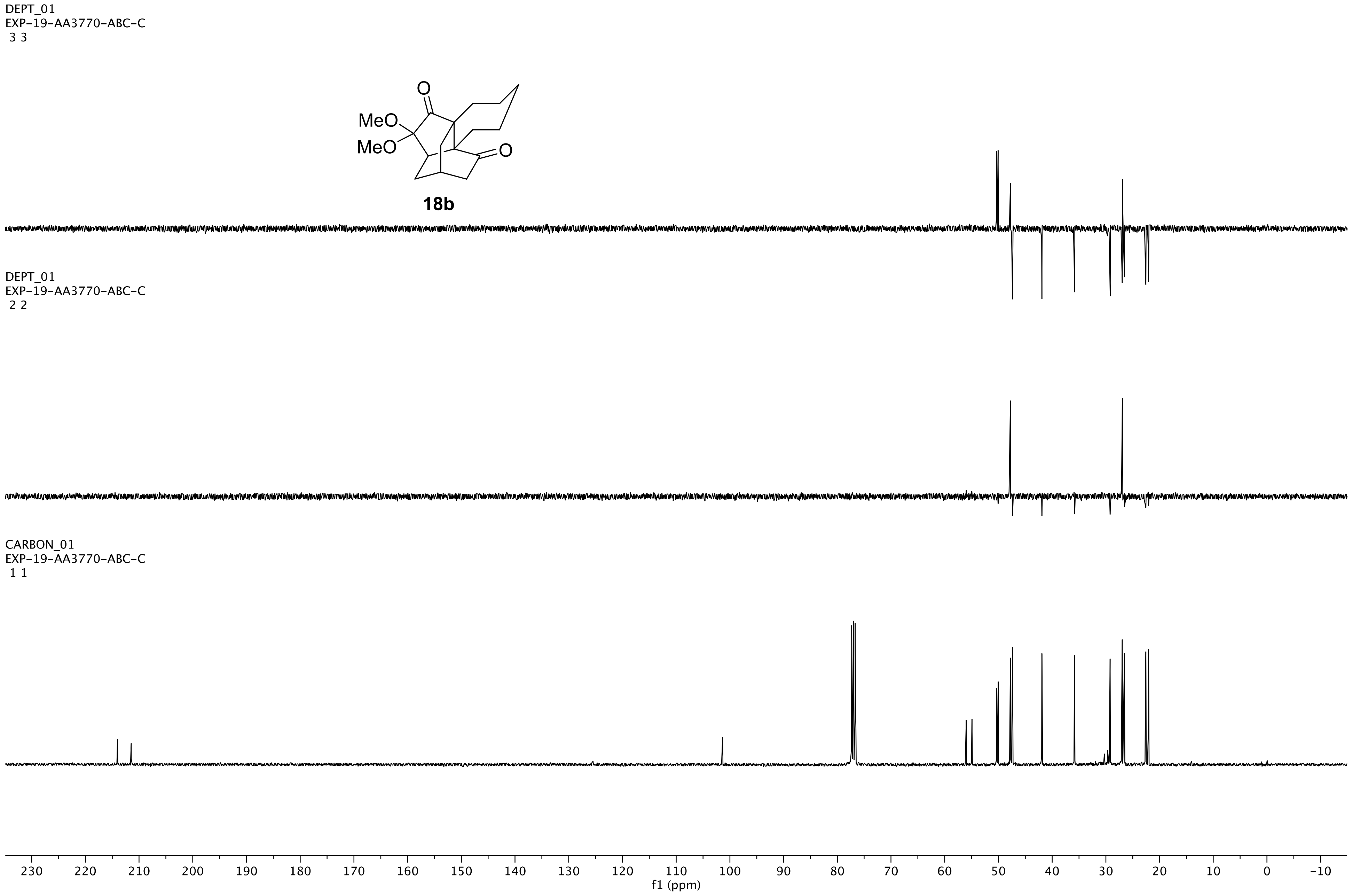
DEPT_01
EXP-19-AA3770-ABC-C
3 3
MeO
MeO
O
O
18b
DEPT_01
EXP-19-AA3770-ABC-C
2 2
CARBON_01
EXP-19-AA3770-ABC-C
1 1
230 220 210 200 190 180 170 160 150 140 130 120 110 100 90 80 70 60 50 40 30 20 10 0 -10
f1 (ppm)

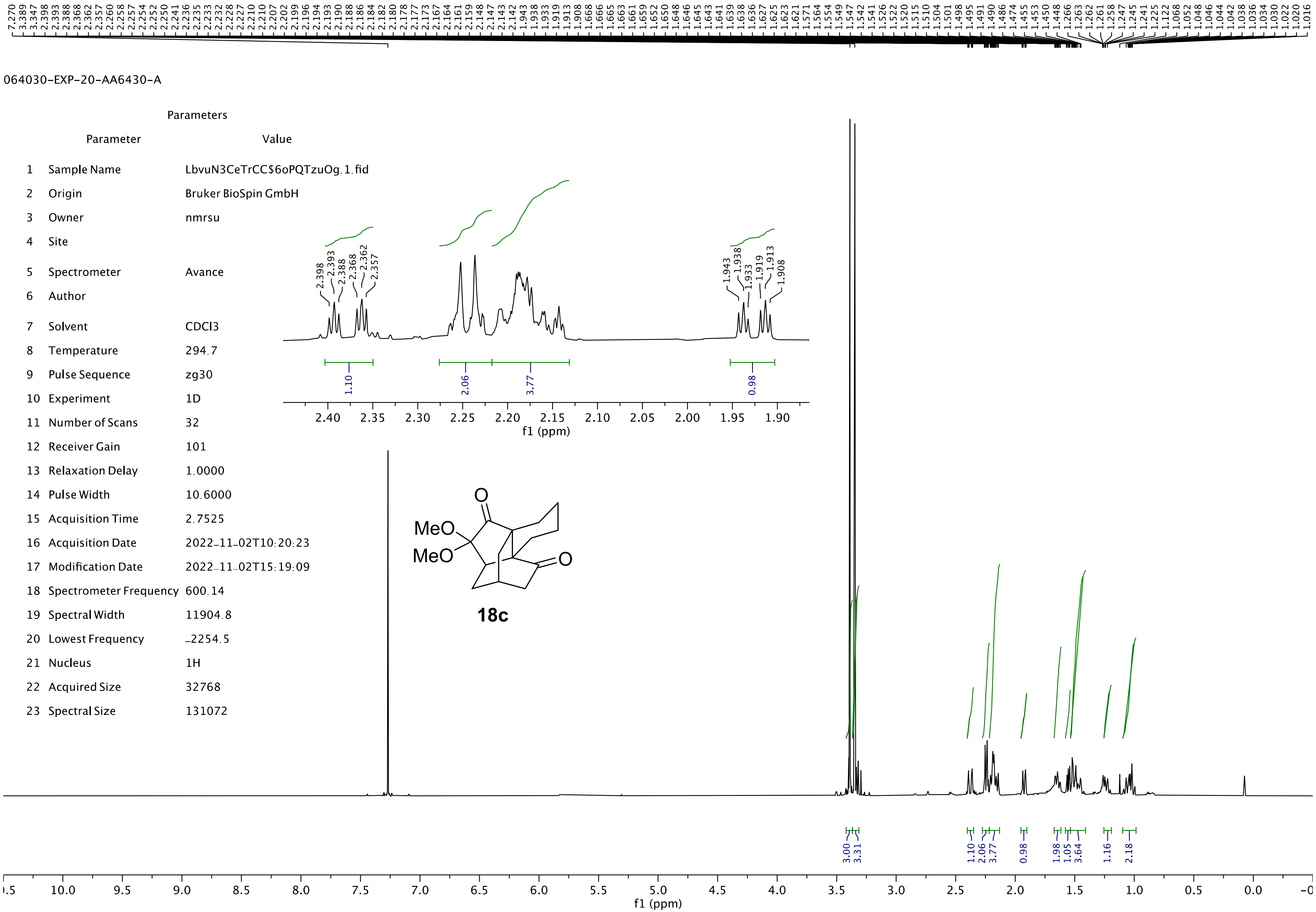

18c

Parameters

	Parameter	Value
1	Sample Name	LbvuN3CeTrCC$6oPQTzuOg.1.fid
2	Origin	Bruker BioSpin GmbH
3	Owner	nmrsu
4	Site	
5	Spectrometer	Avance
6	Author	
7	Solvent	CDCl3
8	Temperature	294.7
9	Pulse Sequence	zg30
10	Experiment	1D
11	Number of Scans	32
12	Receiver Gain	101
13	Relaxation Delay	1.0000
14	Pulse Width	10.6000
15	Acquisition Time	2.7525
16	Acquisition Date	2022-11-02T10:20:23
17	Modification Date	2022-11-02T15:19:09
18	Spectrometer Frequency	600.14
19	Spectral Width	11904.8
20	Lowest Frequency	-2254.5
21	Nucleus	1H
22	Acquired Size	32768
23	Spectral Size	131072

```
Data acquired by:064030

EXP-20-AA6430-A

exp1  CARBON

      SAMPLE                PRESATURATION
date   Mar 15 2020  satmode              n
solvent      cdcl3  wet                  n
file /home/NHRI/vn~       SPECIAL
mrsys/data/064030/~ temp          not used
EXP-20-AA6430-A_20~ gain                30
200315_01/CARBON_0~ spin          not used
                 1  hst              0.008
   ACQUISITION      pw90            15.100
sw         18867.9  alfa            10.000
at           0.868          FLAGS
np           32768  il                   n
fb           10400  in                   n
bs               8  dp                   y
d1           1.000  hs                  nn
nt            2000        PROCESSING
ct            2000  lb                0.50
   TRANSMITTER      fn            not used
tn             C13         DISPLAY
sfrq        75.441  sp             -1138.2
tof         1138.1  wp             18866.8
tpwr            57  rfl             6947.6
pw           7.550  rfp             5808.3
    DECOUPLER       rp               109.7
dn              H1  lp              -323.5
dof              0          PLOT
dm             yyy  wc                 268
decwave          w  sc                   0
dpwr            35  vs                 280
dmf           7700  th                   5
                    ai   cdc   ph
```

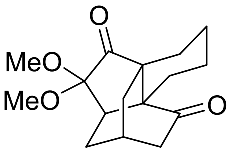

18c

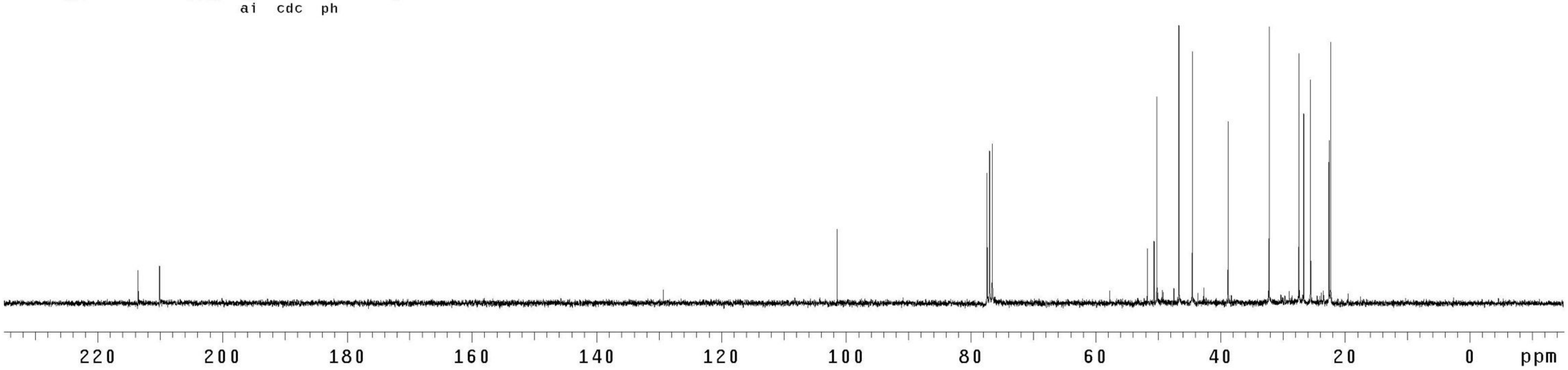

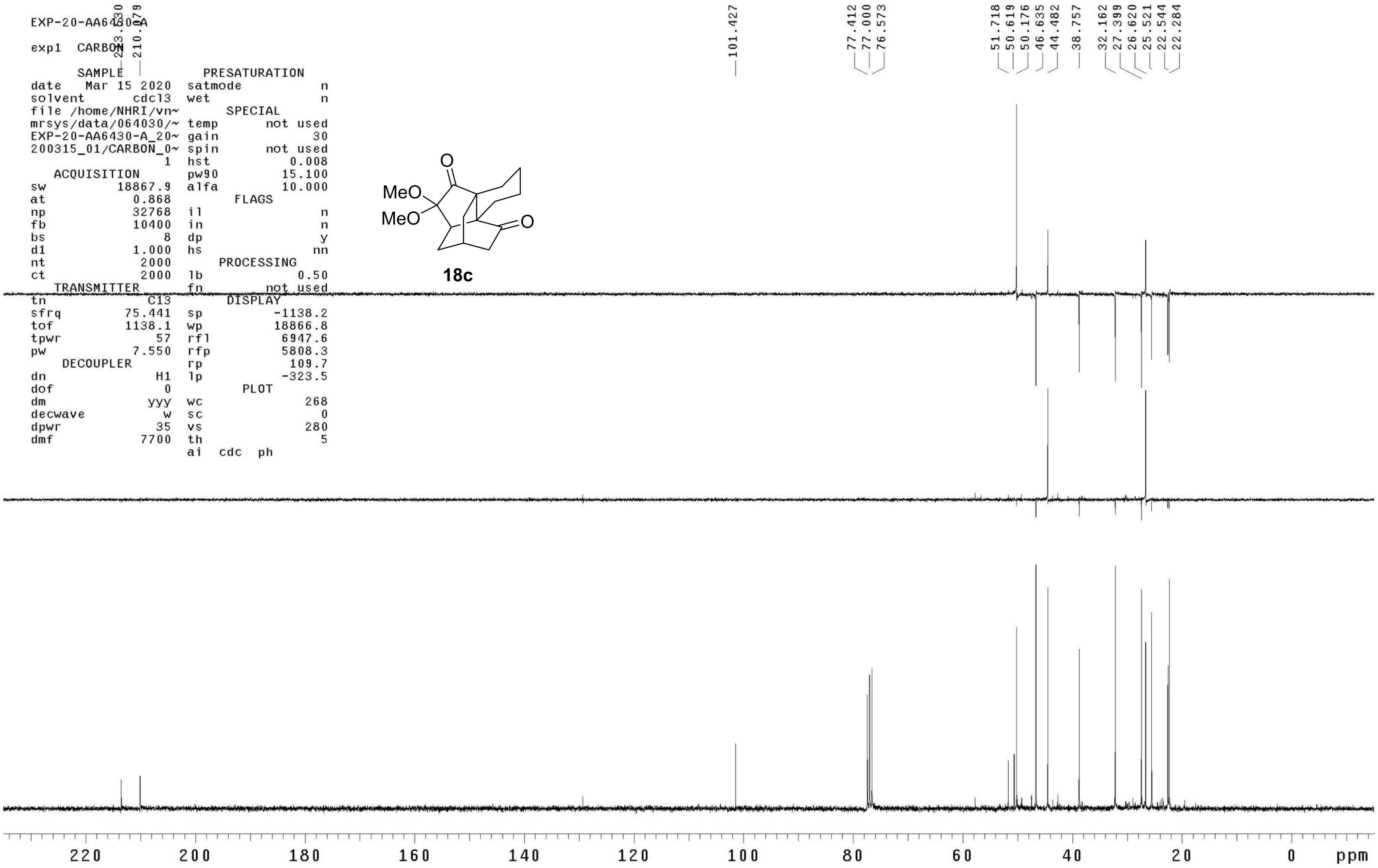

EXP-20-AA6430-A
exp1 CARBON
SAMPLE
date Mar 15 2020
solvent cdcl3
file /home/NHRI/vn~
mrsys/data/064030/~
EXP-20-AA6430-A_20~
200315_01/CARBON_0~
1
ACQUISITION
sw 18867.9
at 0.868
np 32768
fb 10400
bs 8
d1 1.000
nt 2000
ct 2000
TRANSMITTER
tn C13
sfrq 75.441
tof 1138.1
tpwr 57
pw 7.550
DECOUPLER
dn H1
dof 0
dm yyy
decwave w
dpwr 35
dmf 7700
PRESATURATION
satmode n
wet n
SPECIAL
temp not used
gain 30
spin not used
hst 0.008
pw90 15.100
alfa 10.000
FLAGS
il n
in n
dp y
hs nn
PROCESSING
lb 0.50
fn not used
DISPLAY
sp -1138.2
wp 18866.8
rfl 6947.6
rfp 5808.3
rp 109.7
lp -323.5
PLOT
wc 268
sc 0
vs 280
th 5
ai cdc ph
210.179
101.427
77.412
77.000
76.573
51.718
50.619
50.176
46.635
44.482
38.757
32.162
27.399
26.620
25.521
22.544
22.284
MeO
MeO
O
O
18c
220
200
180
160
140
120
100
80
60
40
20
0
ppm

064030-EXP-23-AB0586-A

Parameters

	Parameter	Value
1	Sample Name	VLrzDfxPSGap6drI2J4SXg.1.fid
2	Origin	Bruker BioSpin GmbH
3	Owner	nmrsu
4	Site	
5	Spectrometer	Avance
6	Author	
7	Solvent	CDCl3
8	Temperature	303.0
9	Pulse Sequence	zg30
10	Experiment	1D
11	Number of Scans	32
12	Receiver Gain	101
13	Relaxation Delay	1.0000
14	Pulse Width	8.0000
15	Acquisition Time	4.1943
16	Acquisition Date	2023-04-22T19:06:57
17	Modification Date	2023-06-01T02:12:39
18	Spectrometer Frequency	400.17
19	Spectral Width	7812.5
20	Lowest Frequency	-1440.6
21	Nucleus	1H
22	Acquired Size	32768
23	Spectral Size	65536

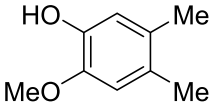

S3

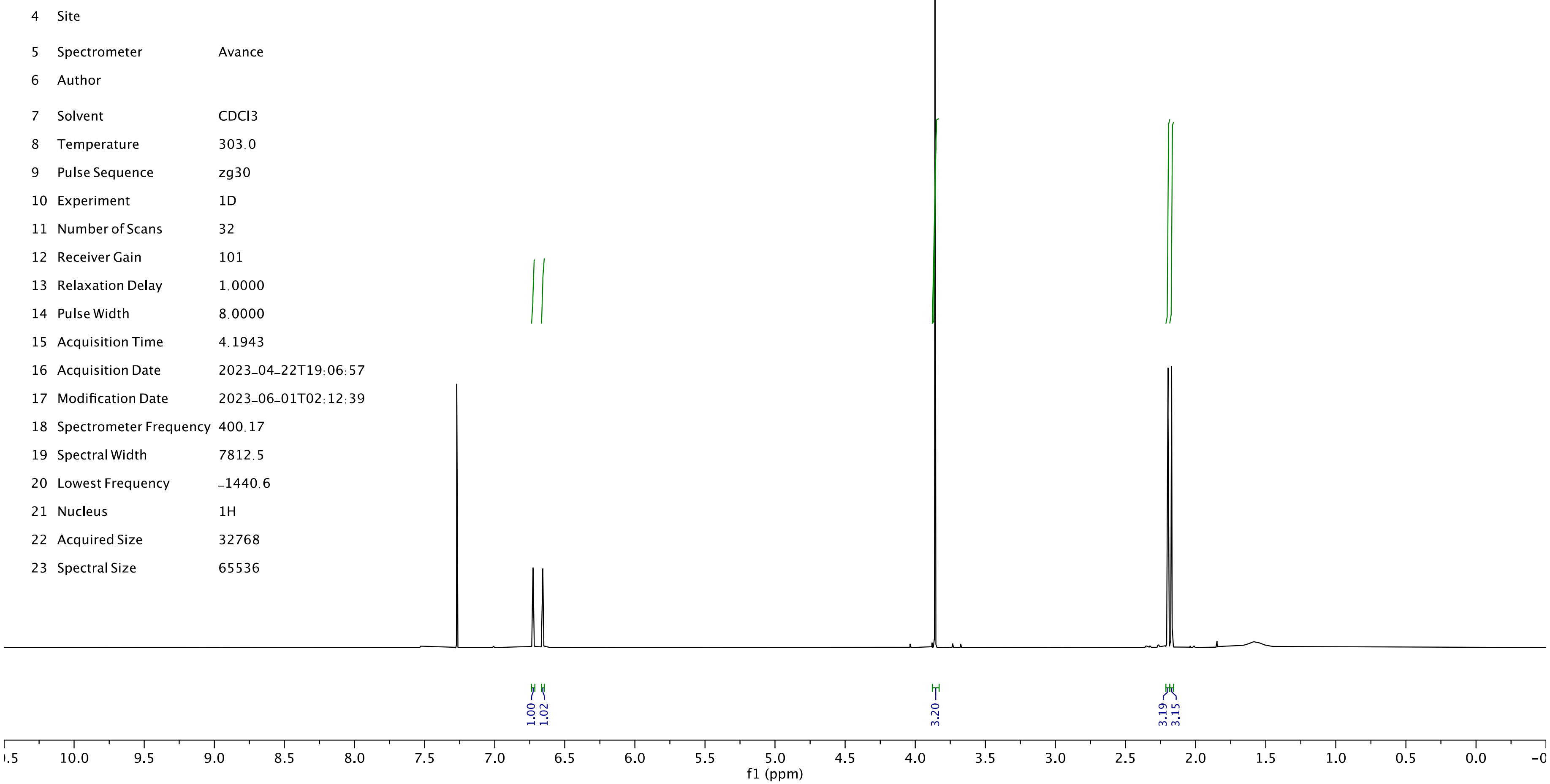

Parameters

	Parameter	Value
1	Sample Name	VLrzDfxPSGap6drI2J4SXg.3.fid
2	Origin	Bruker BioSpin GmbH
3	Owner	nmrsu
4	Site	
5	Spectrometer	Avance
6	Author	
7	Solvent	CDCl3
8	Temperature	303.0
9	Pulse Sequence	zgpg30
10	Experiment	1D
11	Number of Scans	6400
12	Receiver Gain	101
13	Relaxation Delay	2.0000
14	Pulse Width	8.0000
15	Acquisition Time	1.3763
16	Acquisition Date	2023-05-30T08:14:43
17	Modification Date	2023-06-01T02:12:39
18	Spectrometer Frequency	100.63
19	Spectral Width	23809.5
20	Lowest Frequency	-840.7
21	Nucleus	13C
22	Acquired Size	32768
23	Spectral Size	65536

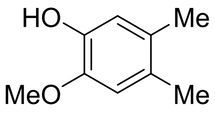

S3

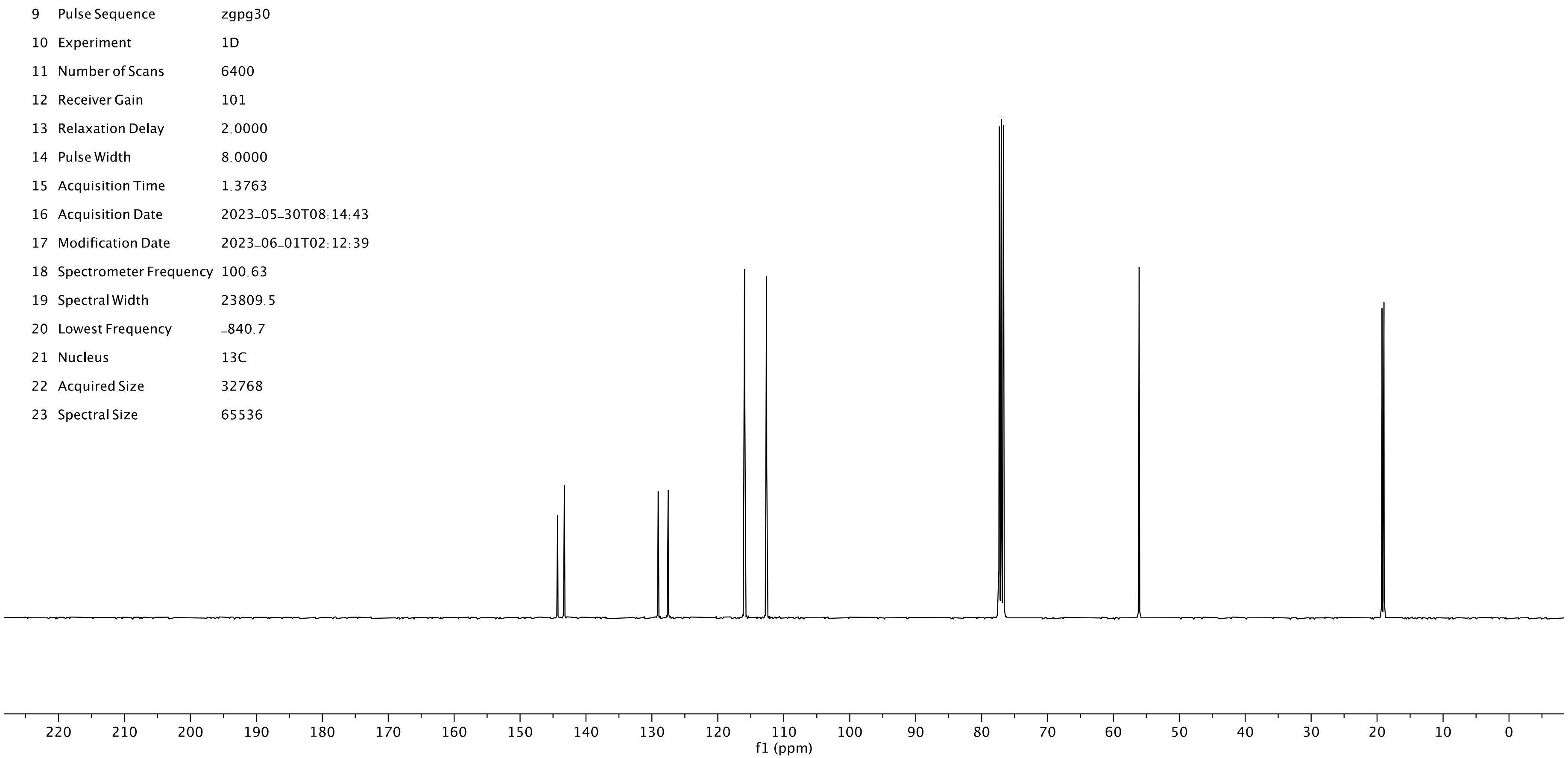

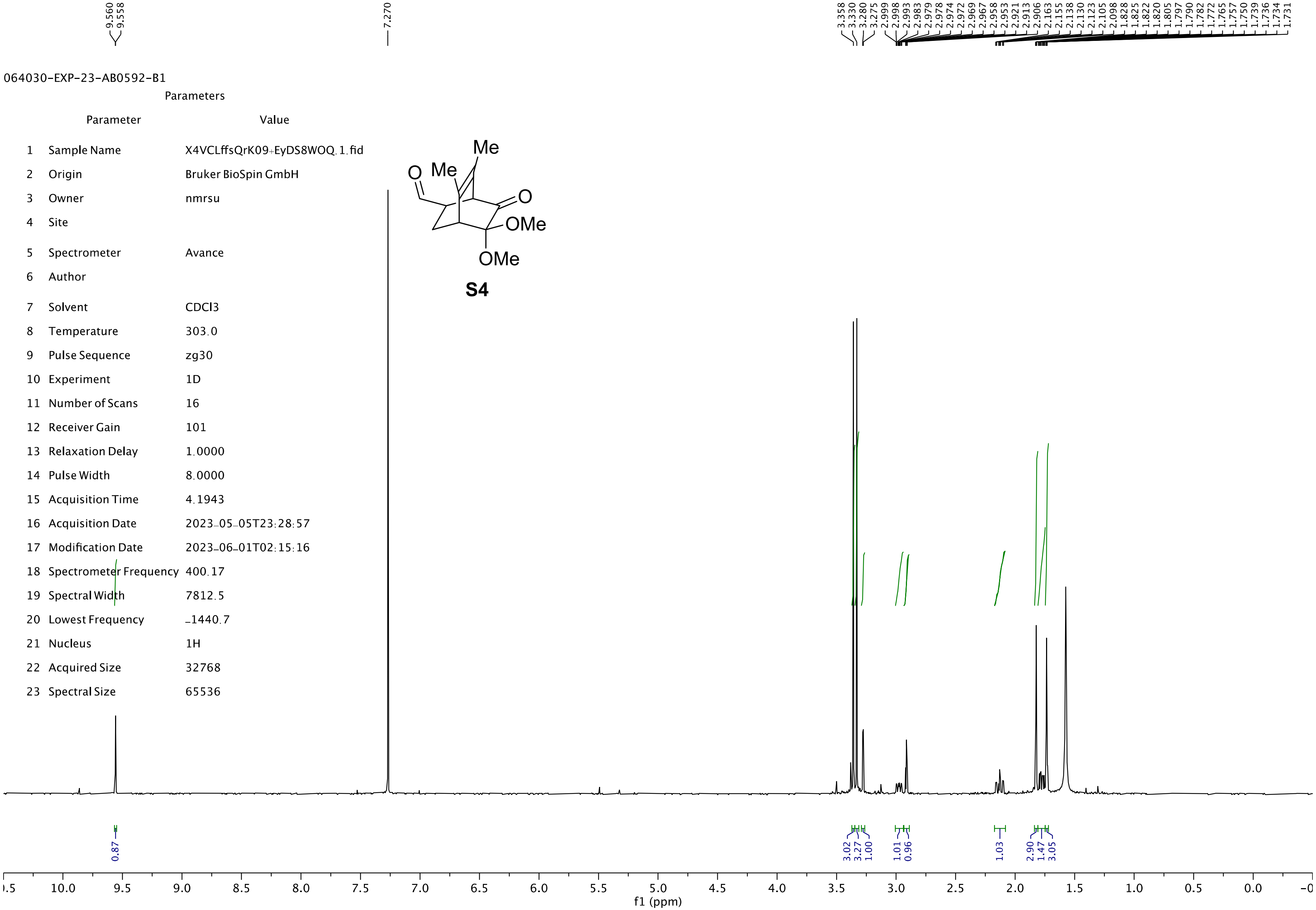

064030-EXP-23-AB0592-B1

Parameters

	Parameter	Value
1	Sample Name	X4VCLffsQrK09+EyDS8WOQ.1.fid
2	Origin	Bruker BioSpin GmbH
3	Owner	nmrsu
4	Site	
5	Spectrometer	Avance
6	Author	
7	Solvent	CDCl3
8	Temperature	303.0
9	Pulse Sequence	zg30
10	Experiment	1D
11	Number of Scans	16
12	Receiver Gain	101
13	Relaxation Delay	1.0000
14	Pulse Width	8.0000
15	Acquisition Time	4.1943
16	Acquisition Date	2023-05-05T23:28:57
17	Modification Date	2023-06-01T02:15:16
18	Spectrometer Frequency	400.17
19	Spectral Width	7812.5
20	Lowest Frequency	-1440.7
21	Nucleus	1H
22	Acquired Size	32768
23	Spectral Size	65536

S4

064030-EXP-23-AB0592-B1

	Parameter	Value
1	Sample Name	X4VCLffsQrK09+EyDS8WOQ.3.fid
2	Origin	Bruker BioSpin GmbH
3	Owner	nmrsu
4	Site	
5	Spectrometer	Avance
6	Author	
7	Solvent	CDCl3
8	Temperature	303.0
9	Pulse Sequence	zgpg30
10	Experiment	1D
11	Number of Scans	5000
12	Receiver Gain	101
13	Relaxation Delay	2.0000
14	Pulse Width	8.0000
15	Acquisition Time	1.3763
16	Acquisition Date	2023-05-06T05:05:24
17	Modification Date	2023-06-01T02:15:16
18	Spectrometer Frequency	100.63
19	Spectral Width	23809.5
20	Lowest Frequency	-849.1
21	Nucleus	13C
22	Acquired Size	32768
23	Spectral Size	65536

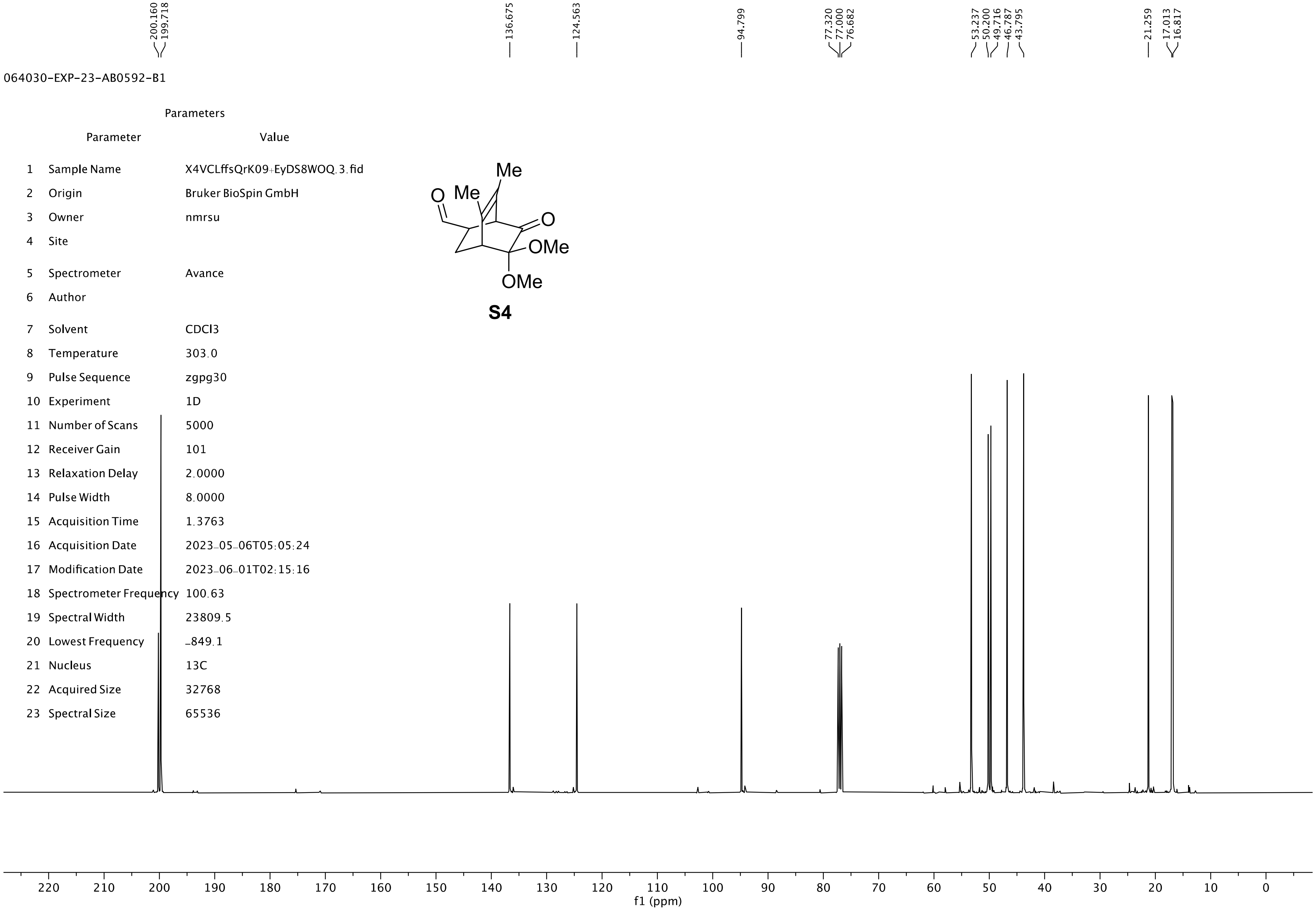

064030-EXP-23-AB0593-B_Combine

Parameters

	Parameter	Value
1	Sample Name	Bp+Gf2gtSW21C5M0XAG$XA.5.fid
2	Origin	Bruker BioSpin GmbH
3	Owner	nmrsu
4	Site	
5	Spectrometer	Avance
6	Author	
7	Solvent	CDCl3
8	Temperature	303.0
9	Pulse Sequence	zg30
10	Experiment	1D
11	Number of Scans	16
12	Receiver Gain	101
13	Relaxation Delay	1.0000
14	Pulse Width	8.0000
15	Acquisition Time	4.1943
16	Acquisition Date	2023-06-04T02:09:13
17	Modification Date	2023-06-04T02:35:50
18	Spectrometer Frequency	400.17
19	Spectral Width	7812.5
20	Lowest Frequency	-1440.6
21	Nucleus	1H
22	Acquired Size	32768
23	Spectral Size	65536

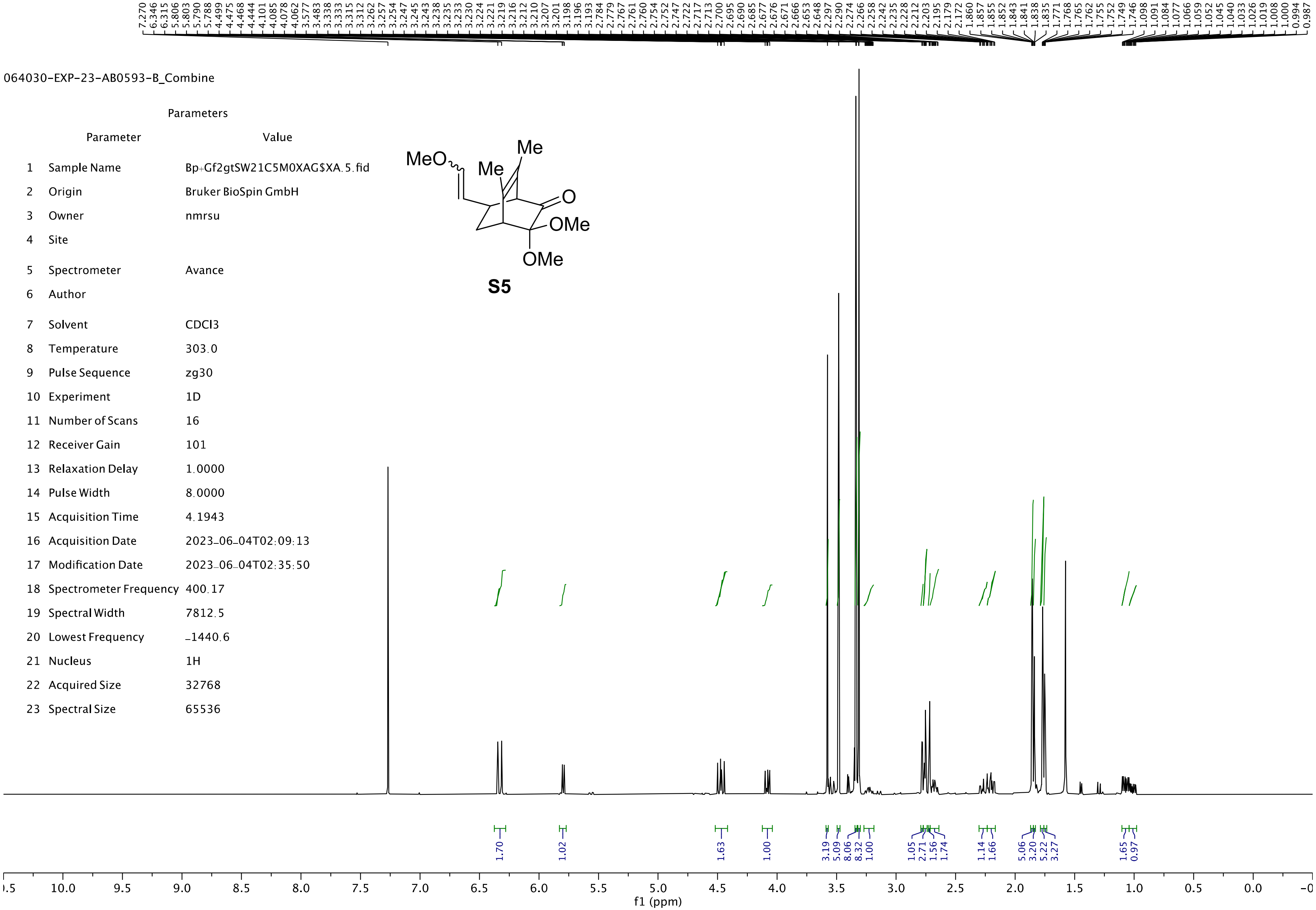

064030-EXP-23-AB0593-B_Combine

Parameters

	Parameter	Value
1	Sample Name	Bp+Gf2gtSW21C5M0XAG$XA.3.fid
2	Origin	Bruker BioSpin GmbH
3	Owner	nmrsu
4	Site	
5	Spectrometer	Avance
6	Author	
7	Solvent	CDCl3
8	Temperature	303.0
9	Pulse Sequence	zgpg30
10	Experiment	1D
11	Number of Scans	5000
12	Receiver Gain	101
13	Relaxation Delay	2.0000
14	Pulse Width	8.0000
15	Acquisition Time	1.3763
16	Acquisition Date	2023-05-15T08:29:56
17	Modification Date	2023-06-04T02:35:50
18	Spectrometer Frequency	100.63
19	Spectral Width	23809.5
20	Lowest Frequency	-842.2
21	Nucleus	13C
22	Acquired Size	32768
23	Spectral Size	65536

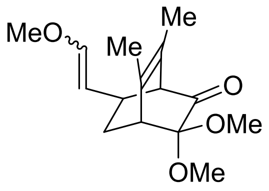

S5

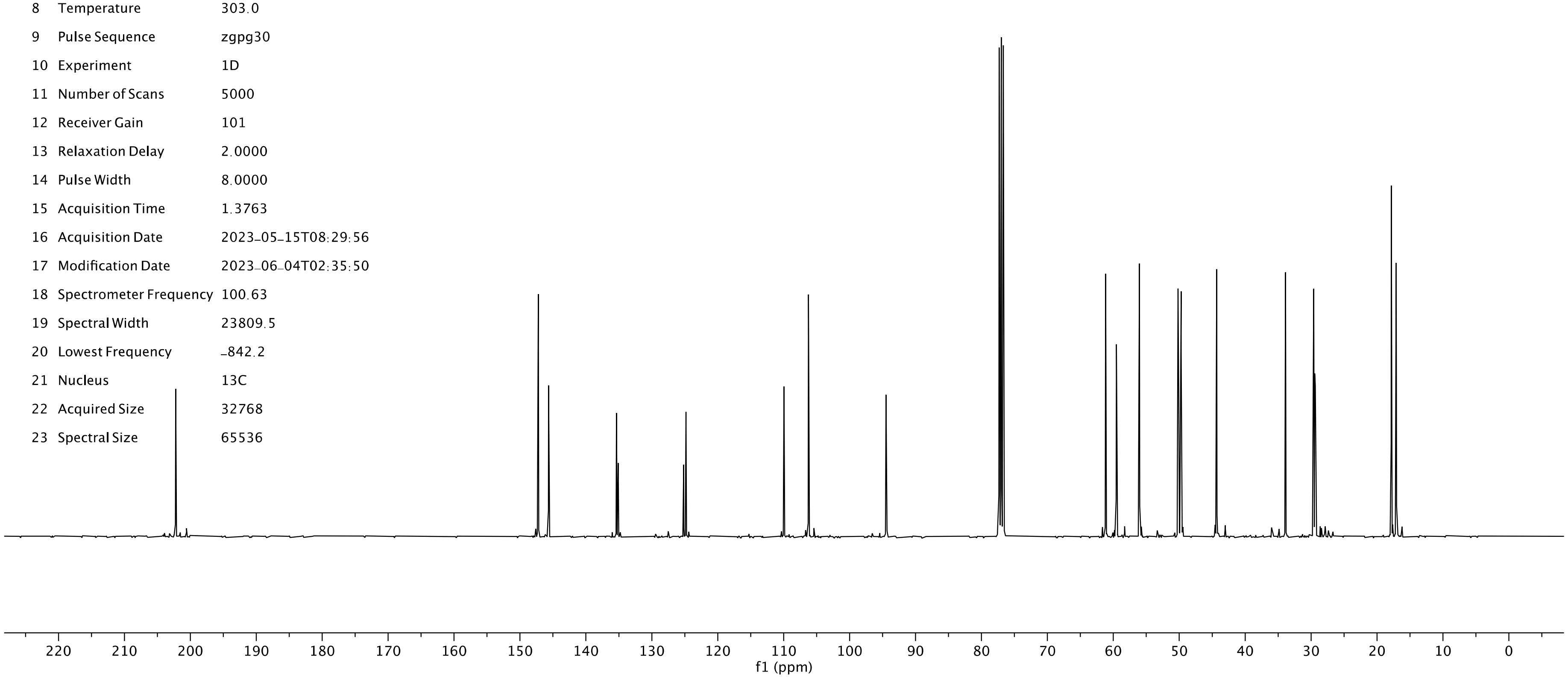

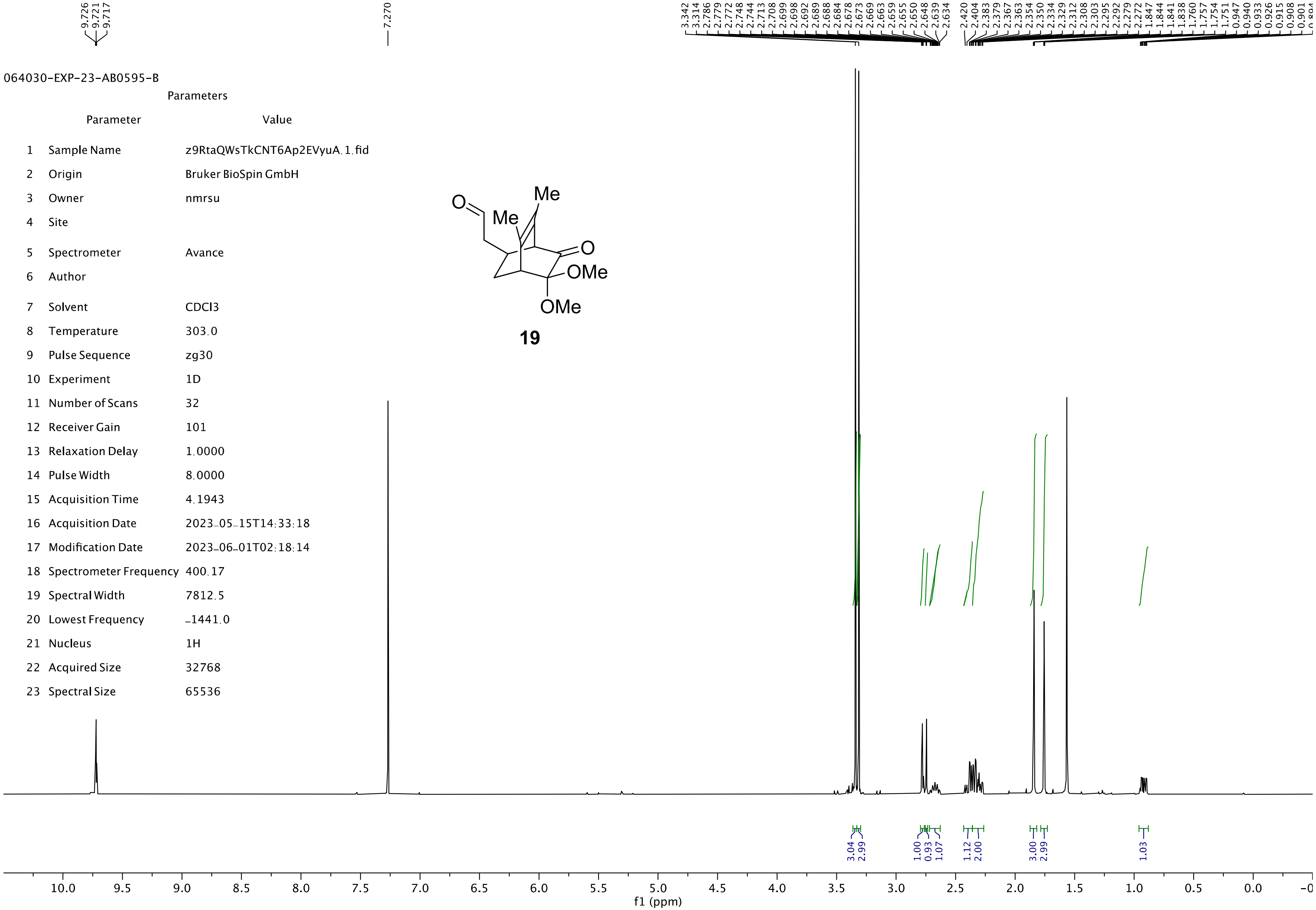

Parameters

	Parameter	Value
1	Sample Name	z9RtaQWsTkCNT6Ap2EVyuA.1.fid
2	Origin	Bruker BioSpin GmbH
3	Owner	nmrsu
4	Site	
5	Spectrometer	Avance
6	Author	
7	Solvent	CDCl3
8	Temperature	303.0
9	Pulse Sequence	zg30
10	Experiment	1D
11	Number of Scans	32
12	Receiver Gain	101
13	Relaxation Delay	1.0000
14	Pulse Width	8.0000
15	Acquisition Time	4.1943
16	Acquisition Date	2023-05-15T14:33:18
17	Modification Date	2023-06-01T02:18:14
18	Spectrometer Frequency	400.17
19	Spectral Width	7812.5
20	Lowest Frequency	-1441.0
21	Nucleus	1H
22	Acquired Size	32768
23	Spectral Size	65536

064030-EXP-23-AB0595-B

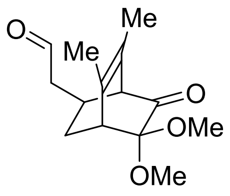

19

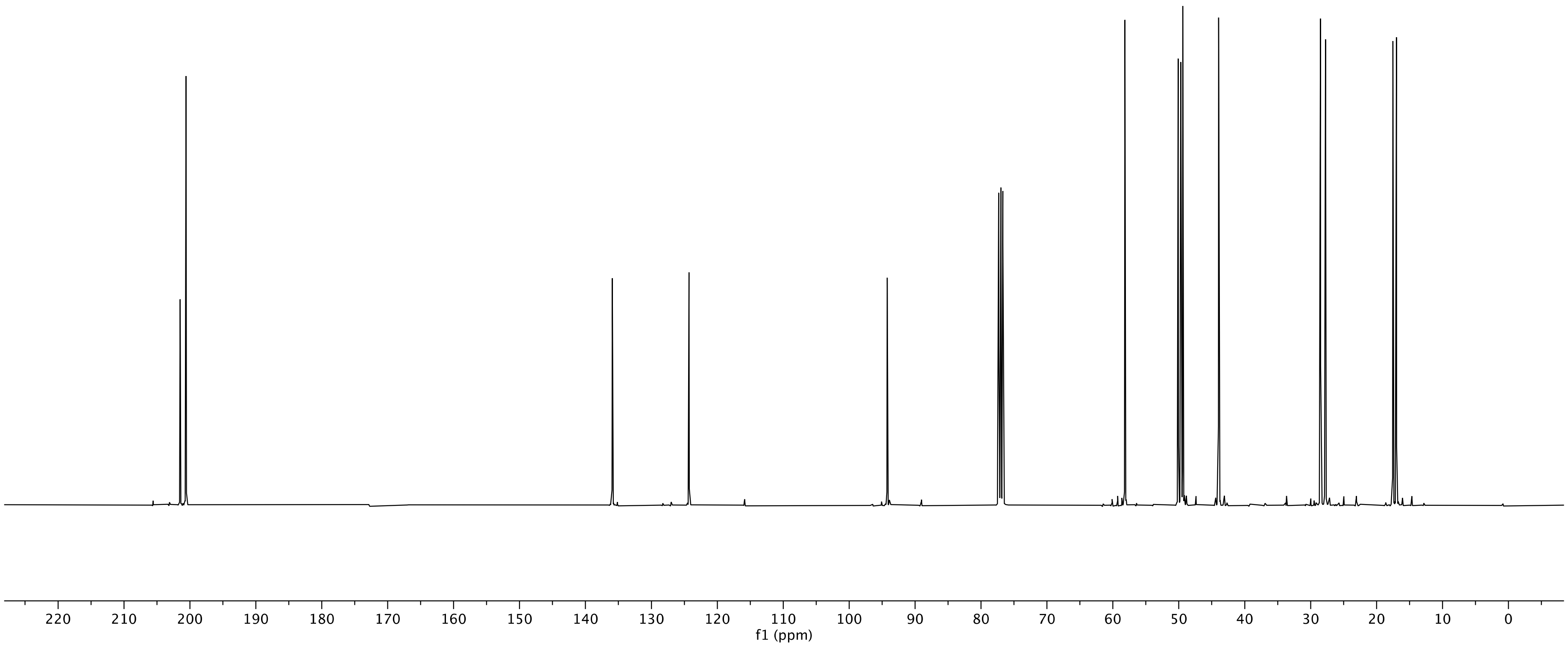

```
Data acquired by:064058

07BP-113-026

exp4  PROTON

     SAMPLE              PRESATURATION
date   Aug 29 2019   satmode              n
solvent      cdcl3   wet                  n
file /home/NHRI/vn~       SPECIAL
mrsys/data/064058/~  temp          not used
07BP-113-026_20190~  gain                34
829_01/PROTON_01.f~  spin                20
                id   hst              0.008
   ACQUISITION       pw90            13.600
sw          6398.0   alfa            10.000
at           2.561          FLAGS
np           32768   il                   n
fb        not used   in                   n
bs               4   dp                   y
d1           1.000   hs                  nn
nt              32         PROCESSING
ct              32   lb                0.20
   TRANSMITTER       fn              131072
tn              H1          DISPLAY
sfrq       399.930   sp              -200.0
tof          415.0   wp              4399.2
tpwr            60   rfl             3702.4
pw           6.800   rfp             2903.5
    DECOUPLER        rp                90.1
dn             C13   lp               -71.7
dof              0            PLOT
dm             nnn   wc                 268
decwave          g   sc                   0
dpwr            43   vs                  66
dmf          17100   th                   2
                     ai   cdc   ph
```

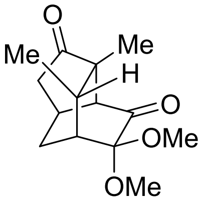

20

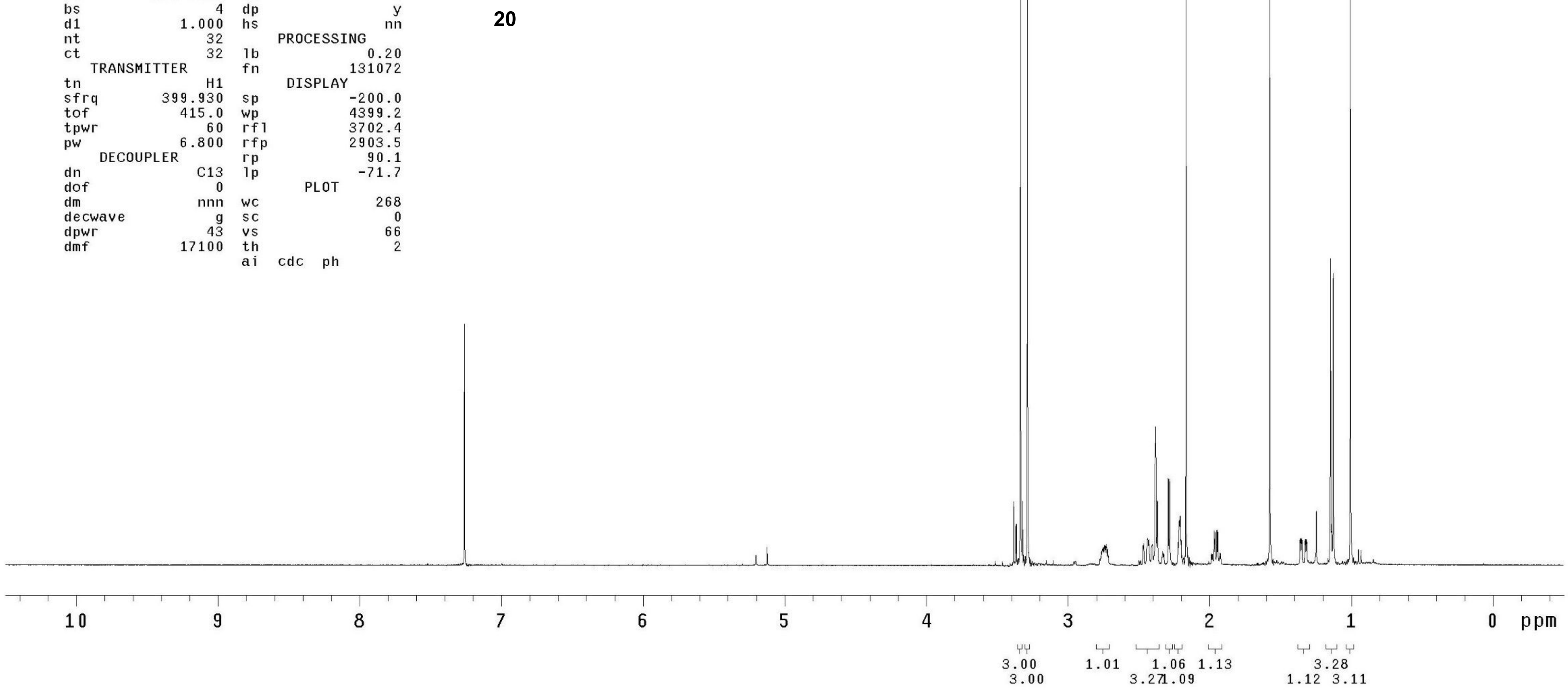

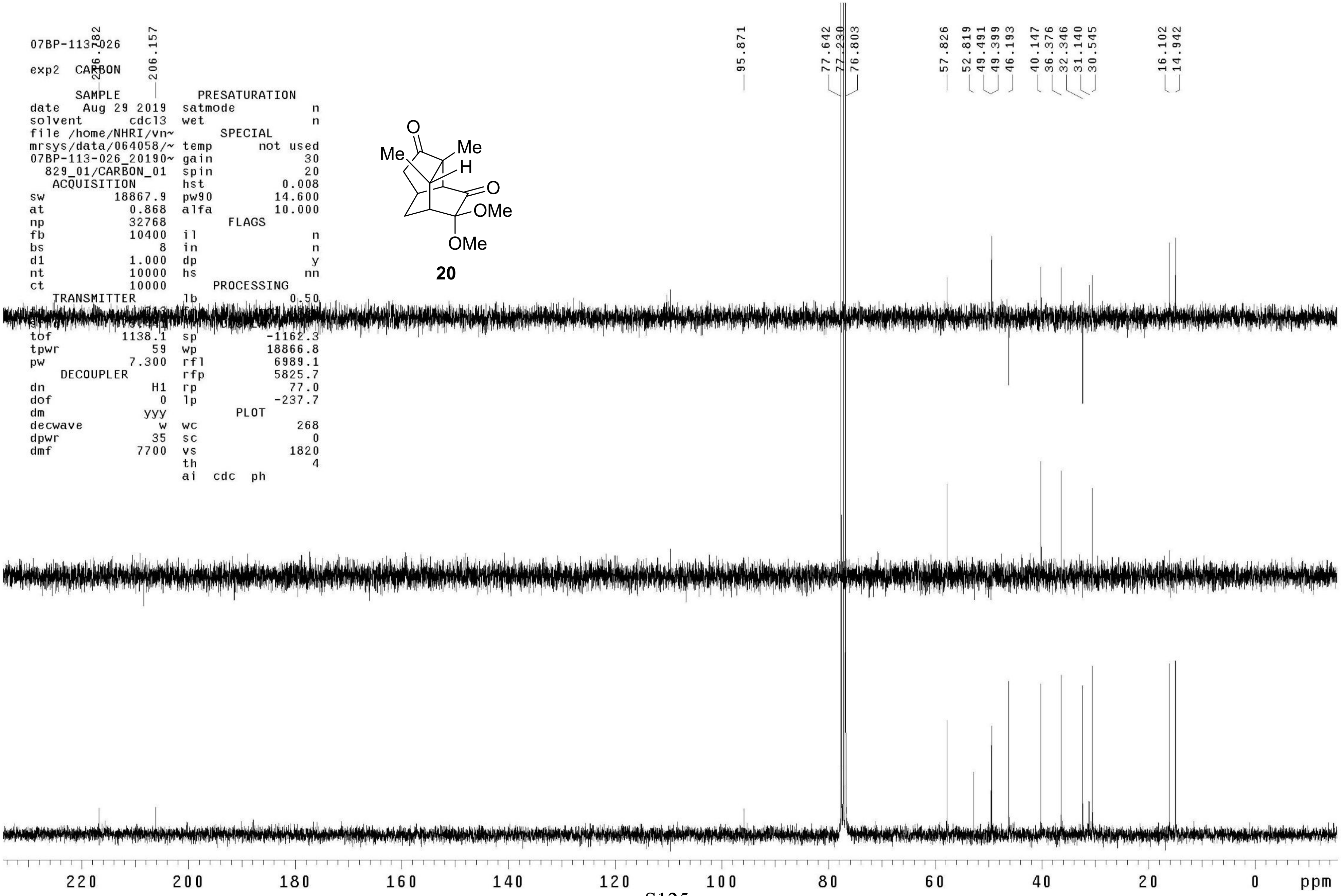
07BP-113-026
exp2 CARBON
SAMPLE
date Aug 29 2019
solvent cdcl3
file /home/NHRI/vn~
mrsys/data/064058/~
07BP-113-026_20190~
829_01/CARBON_01
ACQUISITION
sw 18867.9
at 0.868
np 32768
fb 10400
bs 8
d1 1.000
nt 10000
ct 10000
TRANSMITTER
tof 1138.1
tpwr 59
pw 7.300
DECOUPLER
dn H1
dof 0
dm yyy
decwave w
dpwr 35
dmf 7700
PRESATURATION
satmode n
wet n
SPECIAL
temp not used
gain 30
spin 20
hst 0.008
pw90 14.600
alfa 10.000
FLAGS
il n
in n
dp y
hs nn
PROCESSING
lb 0.50
sp -1162.3
wp 18866.8
rfl 6989.1
rfp 5825.7
rp 77.0
lp -237.7
PLOT
wc 268
sc 0
vs 1820
th 4
ai cdc ph
216.782
206.157
95.871
77.642
77.230
76.803
57.826
52.819
49.491
49.399
46.193
40.147
36.376
32.346
31.140
30.545
16.102
14.942
Me
Me
H
O
O
OMe
OMe
20
220 200 180 160 140 120 100 80 60 40 20 0 ppm

Data acquired by:064058

07BP-113-026-15

exp2 PROTON

SAMPLE		PRESATURATION	
date	Feb 18 2019	satmode	n
solvent	cdcl3	wet	n
file /home/NHRI/vn~		SPECIAL	
mrsys/data/064058/~		temp	not used
07BP-113-026-15_20~		gain	not used
190218_02/PROTON_0~		spin	20
	1.fid	hst	0.008
ACQUISITION		pw90	10.600
sw	4800.8	alfa	10.000
at	1.706	FLAGS	
np	16384	il	n
fb	2600	in	n
bs	4	dp	y
ss	4	hs	nn
d1	1.000	PROCESSING	
nt	16	lb	0.20
ct	16	fn	32768
TRANSMITTER		DISPLAY	
tn	H1	sp	-150.0
sfrq	299.993	wp	3299.6
tof	258.1	rfl	593.6
tpwr	57	rfp	0
pw	5.300	rp	-111.0
DECOUPLER		lp	-11.8
dn	C13	PLOT	
dof	0	wc	268
dm	nnn	sc	0
decwave	g	vs	60
dpwr	38	th	4
dmf	12300	ai cdc ph	

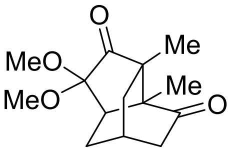

21

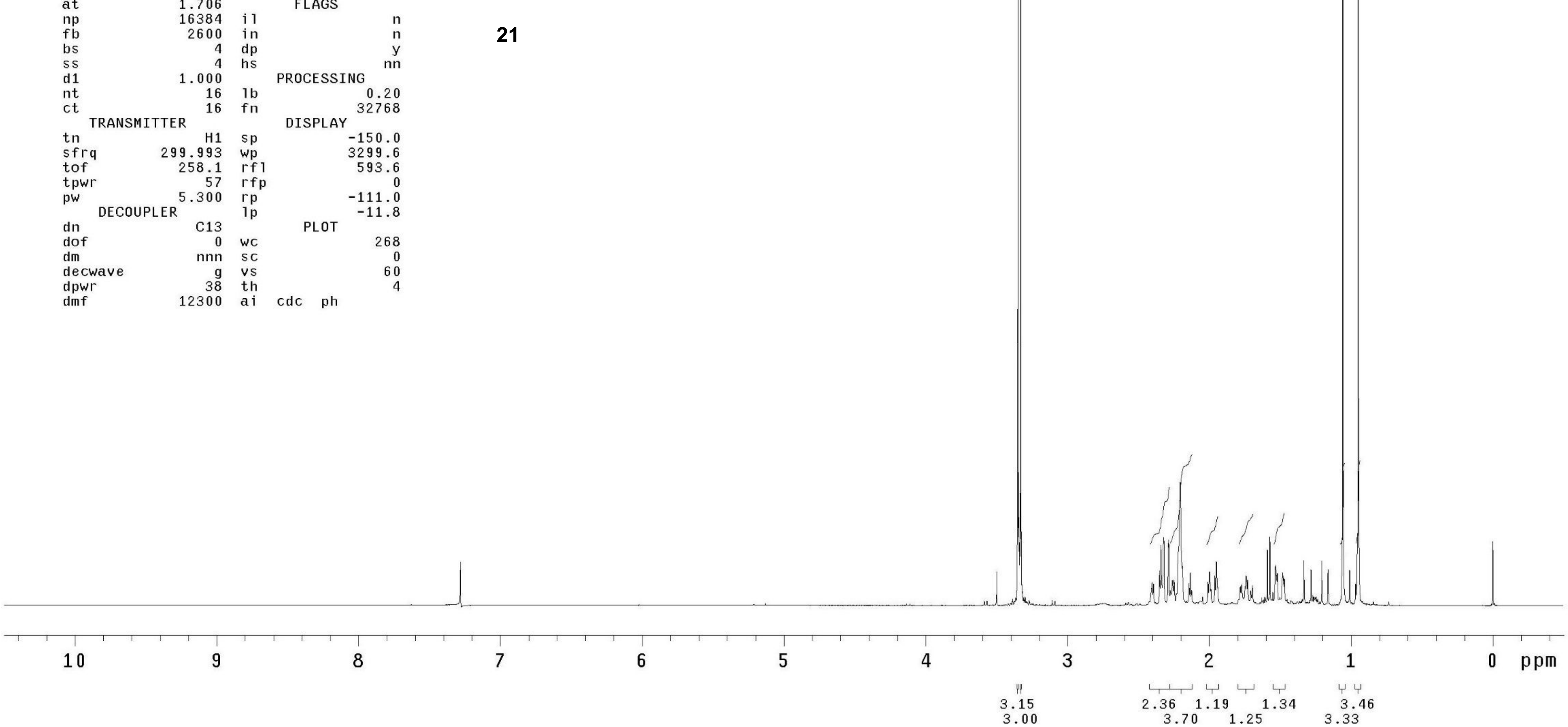

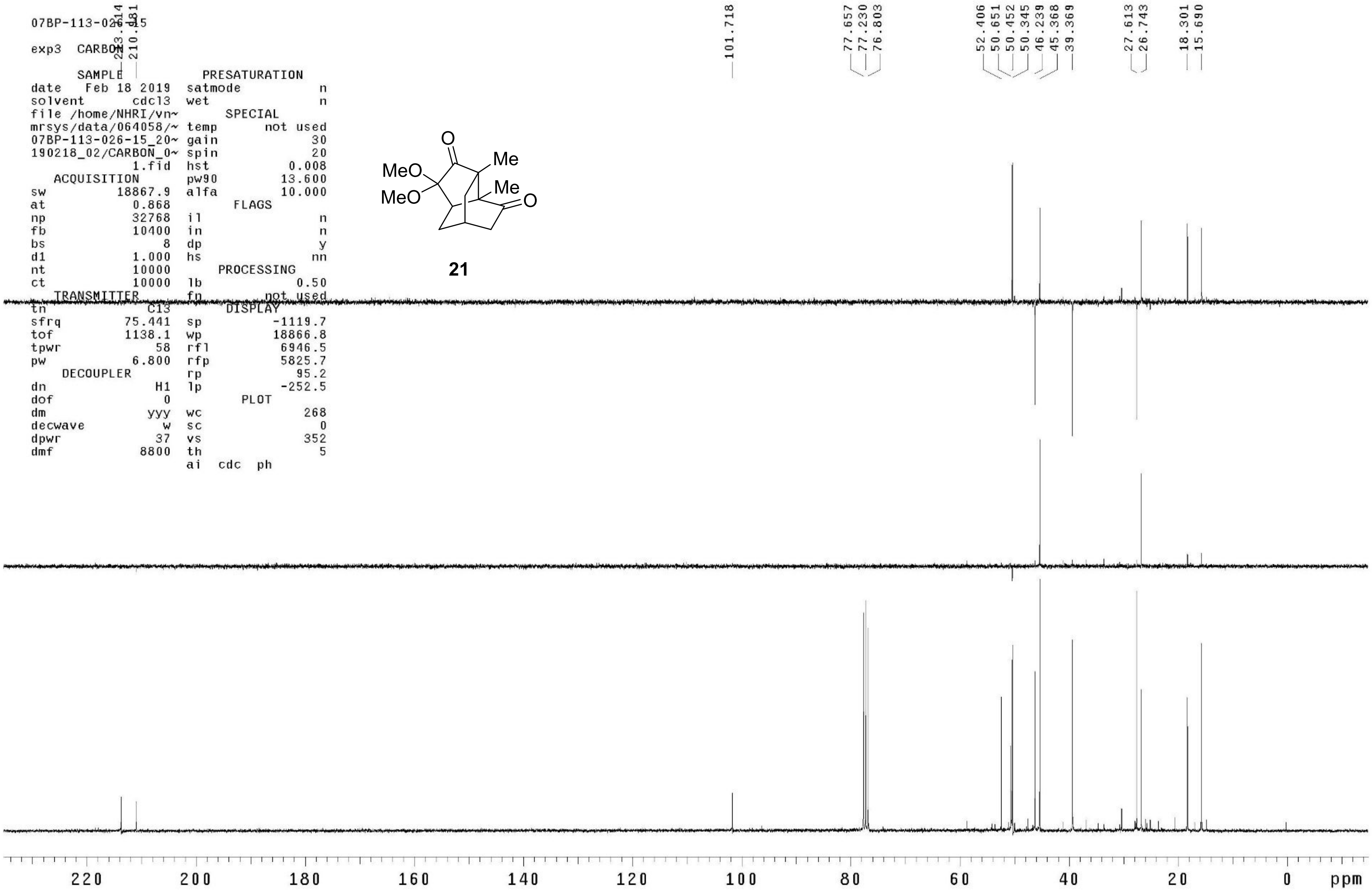
O
Me
MeO
MeO
Me
O
21
07BP-113-026-15
exp3 CARBON
210.981
101.718
77.657
77.230
76.803
52.406
50.651
50.452
50.345
46.239
45.368
39.369
27.613
26.743
18.301
15.690
SAMPLE
date Feb 18 2019
solvent cdcl3
file /home/NHRI/vn~
mrsys/data/064058/~
07BP-113-026-15_20~
190218_02/CARBON_0~
1.fid
ACQUISITION
sw 18867.9
at 0.868
np 32768
fb 10400
bs 8
d1 1.000
nt 10000
ct 10000
TRANSMITTER
tn C13
sfrq 75.441
tof 1138.1
tpwr 58
pw 6.800
DECOUPLER
dn H1
dof 0
dm yyy
decwave w
dpwr 37
dmf 8800
PRESATURATION
satmode n
wet n
SPECIAL
temp not used
gain 30
spin 20
hst 0.008
pw90 13.600
alfa 10.000
FLAGS
il n
in n
dp y
hs nn
PROCESSING
lb 0.50
fn not used
DISPLAY
sp -1119.7
wp 18866.8
rfl 6946.5
rfp 5825.7
rp 95.2
lp -252.5
PLOT
wc 268
sc 0
vs 352
th 5
ai cdc ph
220
200
180
160
140
120
100
80
60
40
20
0
ppm
